# Supplementary material for: PARP7 is a proteotoxic stress sensor that labels proteins for degradation
Source: EMBO J. 2025 Aug 20;44(19):5463–81. doi: 10.1038/s44318-025-00545-7 (PMC12488922; doi:10.1038/s44318-025-00545-7)
Supplement: Supplementary file 1 — Appendix [file 44318_2025_545_MOESM1_ESM.pdf]

Appendix for PARP7 is a proteotoxic stress sensor which labels proteins for degradation

Table of contents

Appendix Figure S1 – page 2
Appendix Figure S2 – page 3
Appendix Figure S3 – page 4

Appendix Figure S1

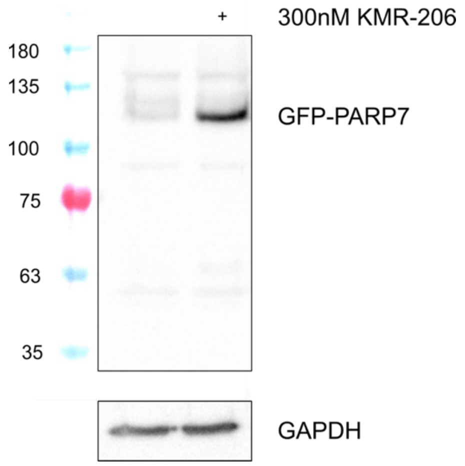

Appendix Figure S1. HEK293 cells were transfected with GFP-PARP7, 24h after transfection followed by addition of 300 nM PARP7 inhibitor KMR-206 overnight. Cells were lysed in RIPA lysis buffer and samples analysed using western blot with indicated antibodies.

Appendix Figure S2

A

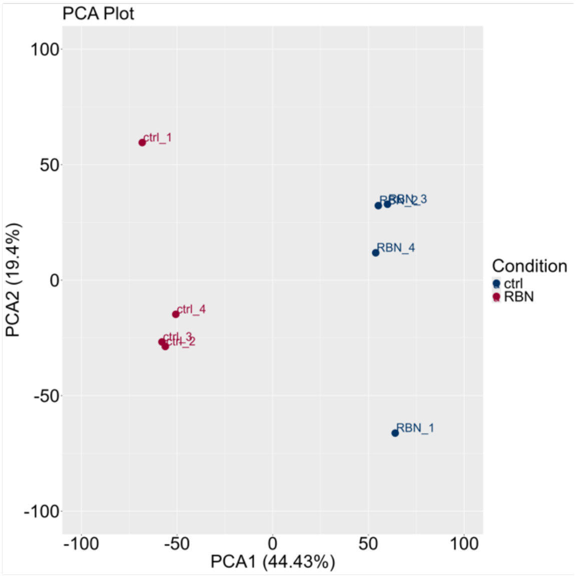

B

Gene	logFC	adj.P.Val	Mass (kDa)
TIPARP	7,217574	2,1513E-12	76,2
SLC16A6	4,36753677	6,04E-10	57,4
TESC	1,71840341	1,2173E-06	24,8
GALNT6	1,64242818	0,02317168	71,2
ZSCAN22	1,40446095	2,491E-05	54,6
DUSP1	1,37466448	4,7904E-09	39,3
PGK2	1,26211449	0,00316055	44,8
WNT7B	1,23965078	4,824E-06	39,3
IL11	1,19990487	1,3388E-06	21,4
CD274	1,196586	1,3234E-05	33,9
CGA	1,17792446	1,3391E-08	13,1
EFNA1	1,09202072	1,1378E-06	23,3
NR4A2	1,09034478	8,0995E-06	67,5
CD83	1,07036443	2,6754E-06	25,9
TUBAL3	1,06447652	0,00072033	38
FAM43A	1,0402974	3,2613E-06	45,2
ZNF557	1,02326752	0,05397961	79,4
NPTX1	0,95960532	2,8842E-06	45,3
MAFF	0,951931	0,00010651	33,7
CRTC2	0,94519222	1,2173E-06	78,2
FOXE1	0,93294192	2,6823E-05	33,5
DMBT1	0,91865539	3,9131E-07	260,7
CYB561A3	0,90609049	0,03079824	24,8
S100P	0,9047333	0,01167438	10,7
RIMKLB	0,87500915	4,0137E-07	39,4
OLR1	0,87163564	8,8417E-06	28,5
FOS	0,85759685	3,999E-05	42,5
FEM1C	0,85612898	0,00135032	75,3
MACROH2A2	0,83182592	0,00568558	64,1
AKAP12	0,82043991	8,002E-07	191,5

Appendix Figure S2: Analysis of protein abundance following PARP7 inhibition. (**A**) HeLa cells were incubated with 100 nM RBN2397 (PARP7i) overnight and harvested by trypsinisation. Pellets were washed 3x in PBS before processing for mass spectrometry. Displayed here is a principal component analysis (PCA) analysis of the 4 replicates. (**B**) Overview of the 30 proteins most affected by PARP7 inhibition.

Appendix Figure S3

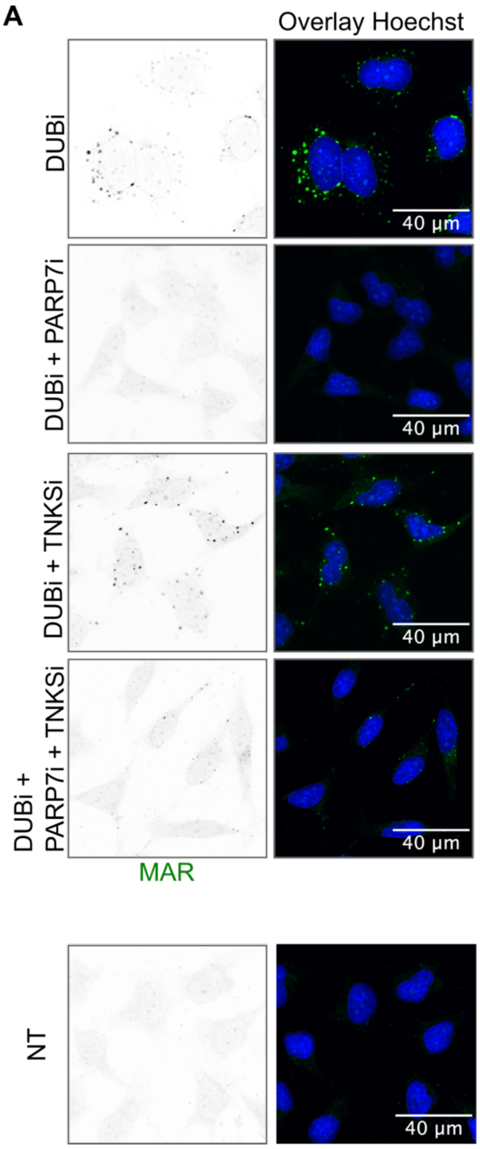

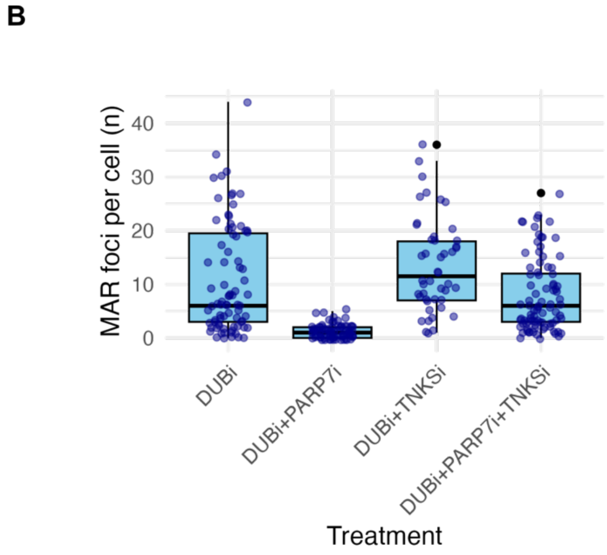

	DUBi	DUBi+RBN	DUBi+XAV
DUBi+RBN	1.0e-15	-	-
DUBi+XAV	0.03552	< 2e-16	-
DUBi+RBN+XAV	0.17348	2.4e-16	0.00021

Appendix Figure S3: influence of PARP inhibition on foci formation. (A) HeLa cells were incubated with 5 µM DUB inhibitor PR-619 for 30 minutes in presence of PARP7 and/or TNKS inhibitors as indicated. Cells were fixed in methanol, stained with an ADP-ribose antibody and analysed using confocal microscopy. **(B)** Analysis of the samples shown in (A), with a bar chart showing the number of MAR foci per cell (top) and a statistical evaluation using a Wilcoxon rank sum test (bottom). Boxplots indicate the median (horizontal line), interquartile range (IQR; box boundaries), whiskers extending to maximum and minimum datapoints within a range of 1.5 x IQR, and individual data points represented by dots.
