# Supplementary figures and images for: PARP7 is a proteotoxic stress sensor that labels proteins for degradation (part 1 of 2)
Source: EMBO J. 2025 Aug 20;44(19):5463–81. doi: 10.1038/s44318-025-00545-7 (PMC12488922; doi:10.1038/s44318-025-00545-7)

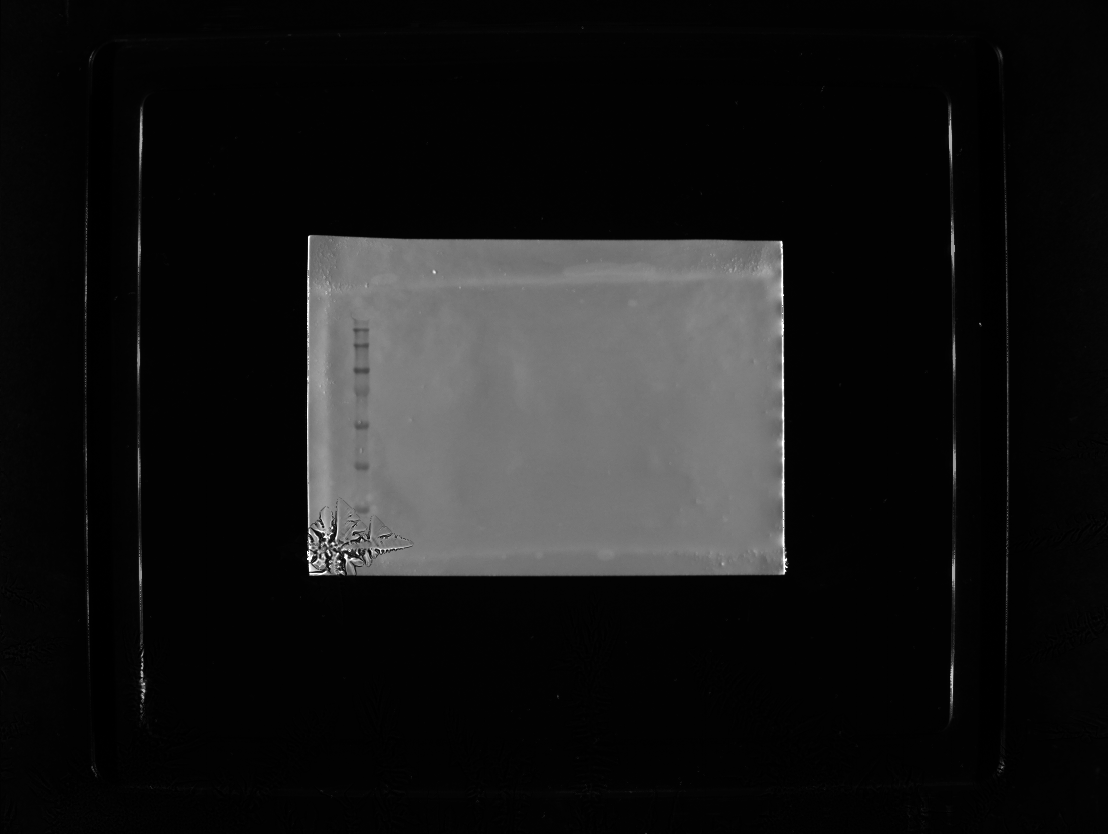

Supplement: Supplementary file 3 — Source data Fig. 2 [file 44318_2025_545_MOESM3_ESM.zip › Fig 2/2F/2F HeLa/31012024 HeLa BTZ TC Ub/2024-0131-105833.tif]

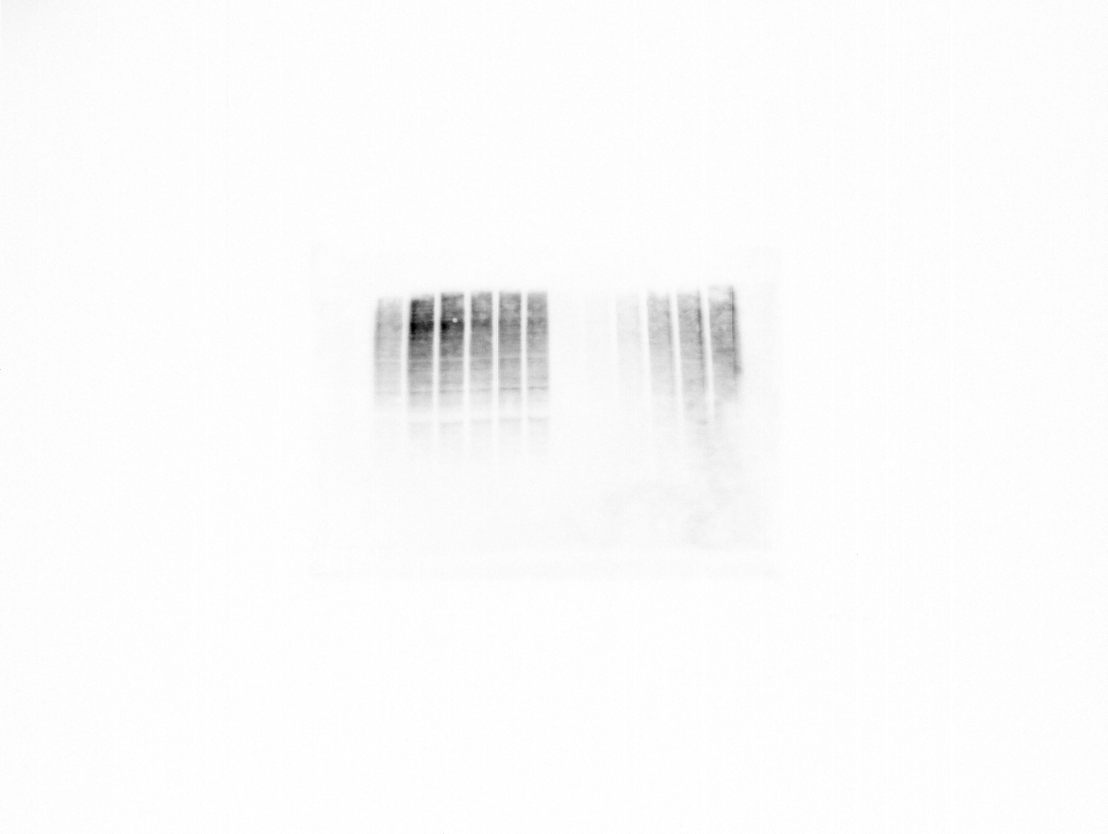

Supplement: Supplementary file 3 — Source data Fig. 2 [file 44318_2025_545_MOESM3_ESM.zip › Fig 2/2F/2F HeLa/31012024 HeLa BTZ TC Ub/2024-0131-105834.tif]

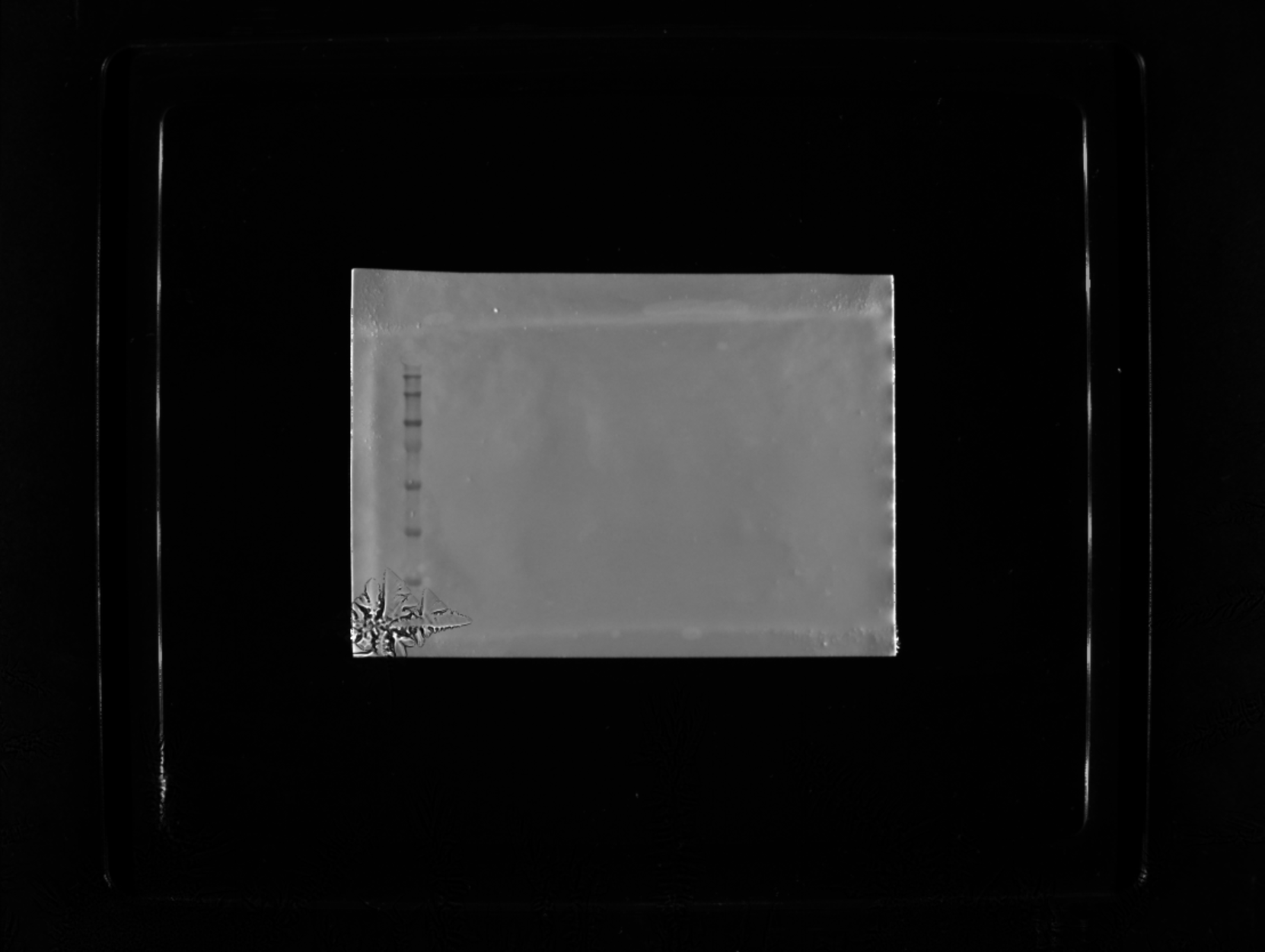

Supplement: Supplementary file 3 — Source data Fig. 2 [file 44318_2025_545_MOESM3_ESM.zip › Fig 2/2F/2F HeLa/31012024 HeLa BTZ TC Ub/2024-0131-105833_pub.tif]

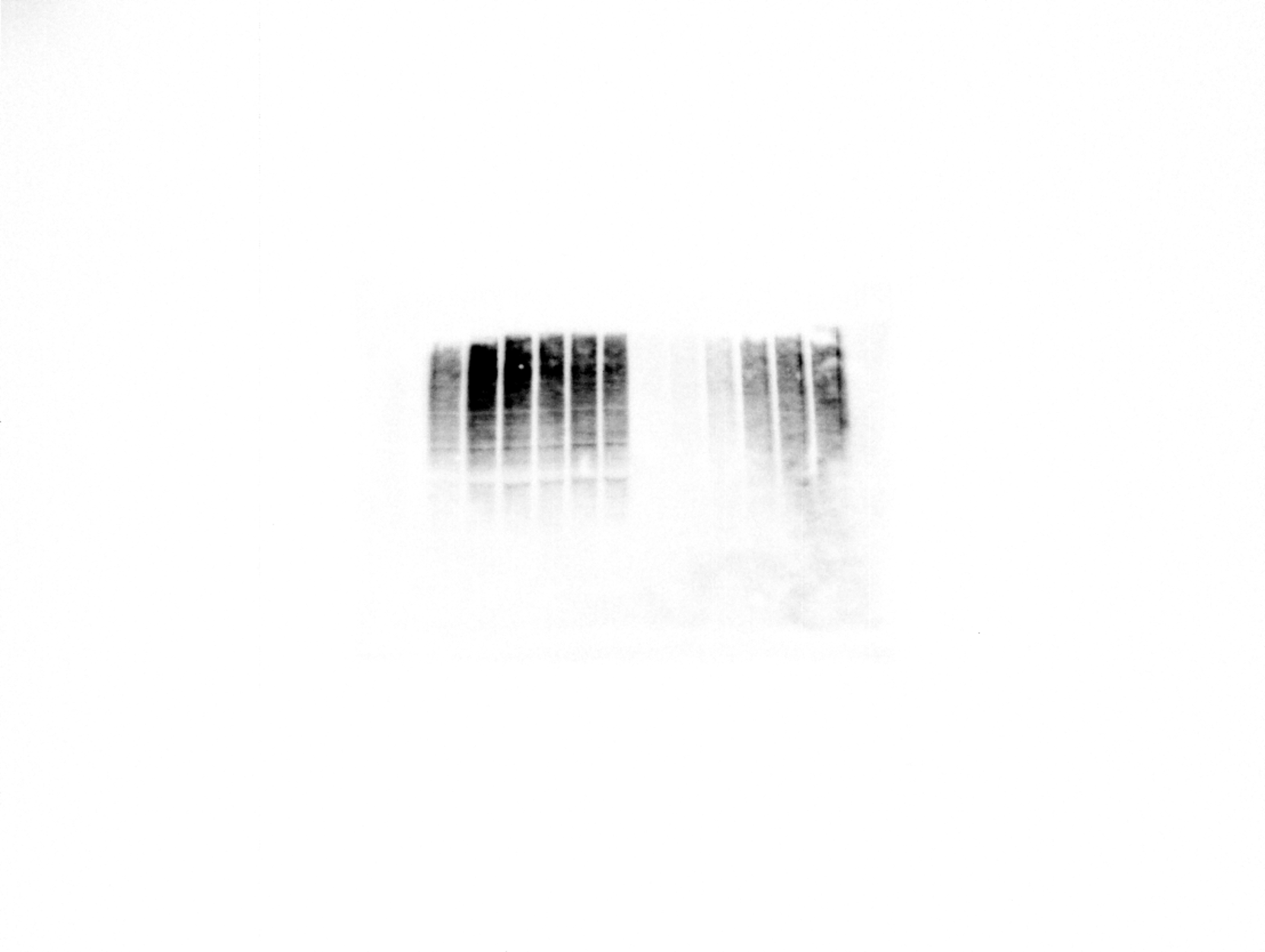

Supplement: Supplementary file 3 — Source data Fig. 2 [file 44318_2025_545_MOESM3_ESM.zip › Fig 2/2F/2F HeLa/31012024 HeLa BTZ TC Ub/S1F1-0131-105836_pub.tif]

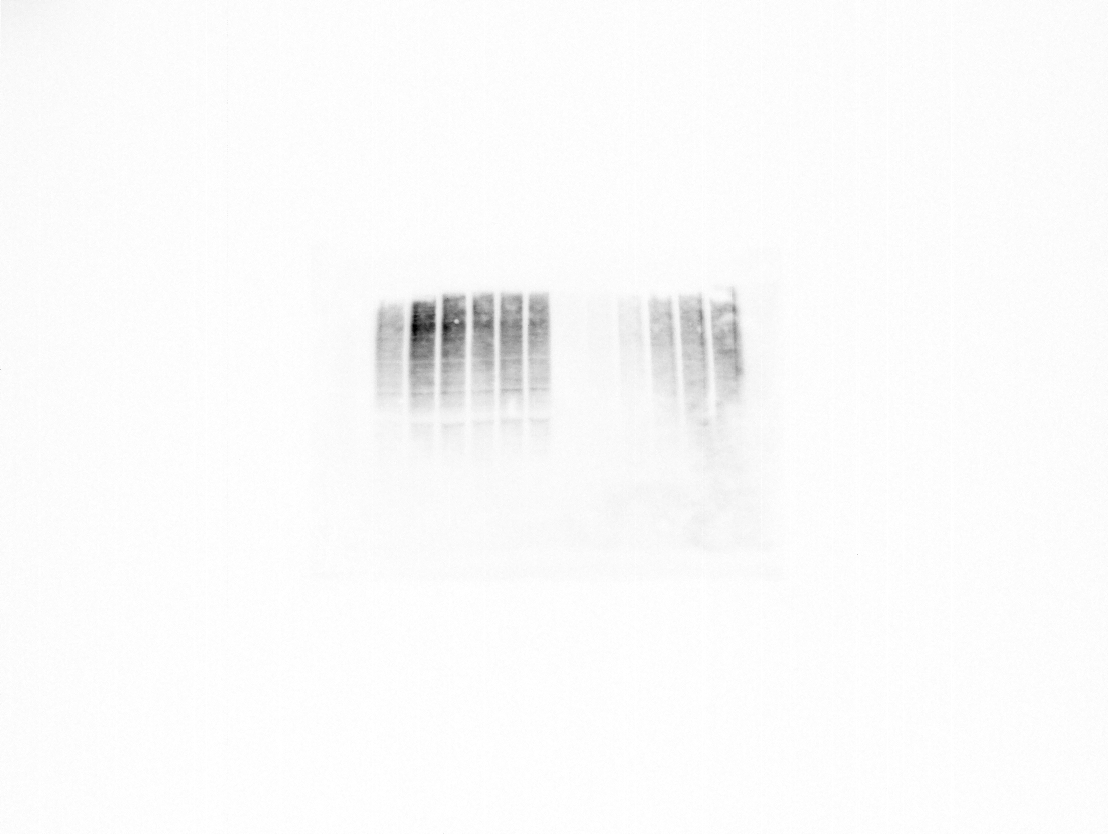

Supplement: Supplementary file 3 — Source data Fig. 2 [file 44318_2025_545_MOESM3_ESM.zip › Fig 2/2F/2F HeLa/31012024 HeLa BTZ TC Ub/S1F1-0131-105836.tif]

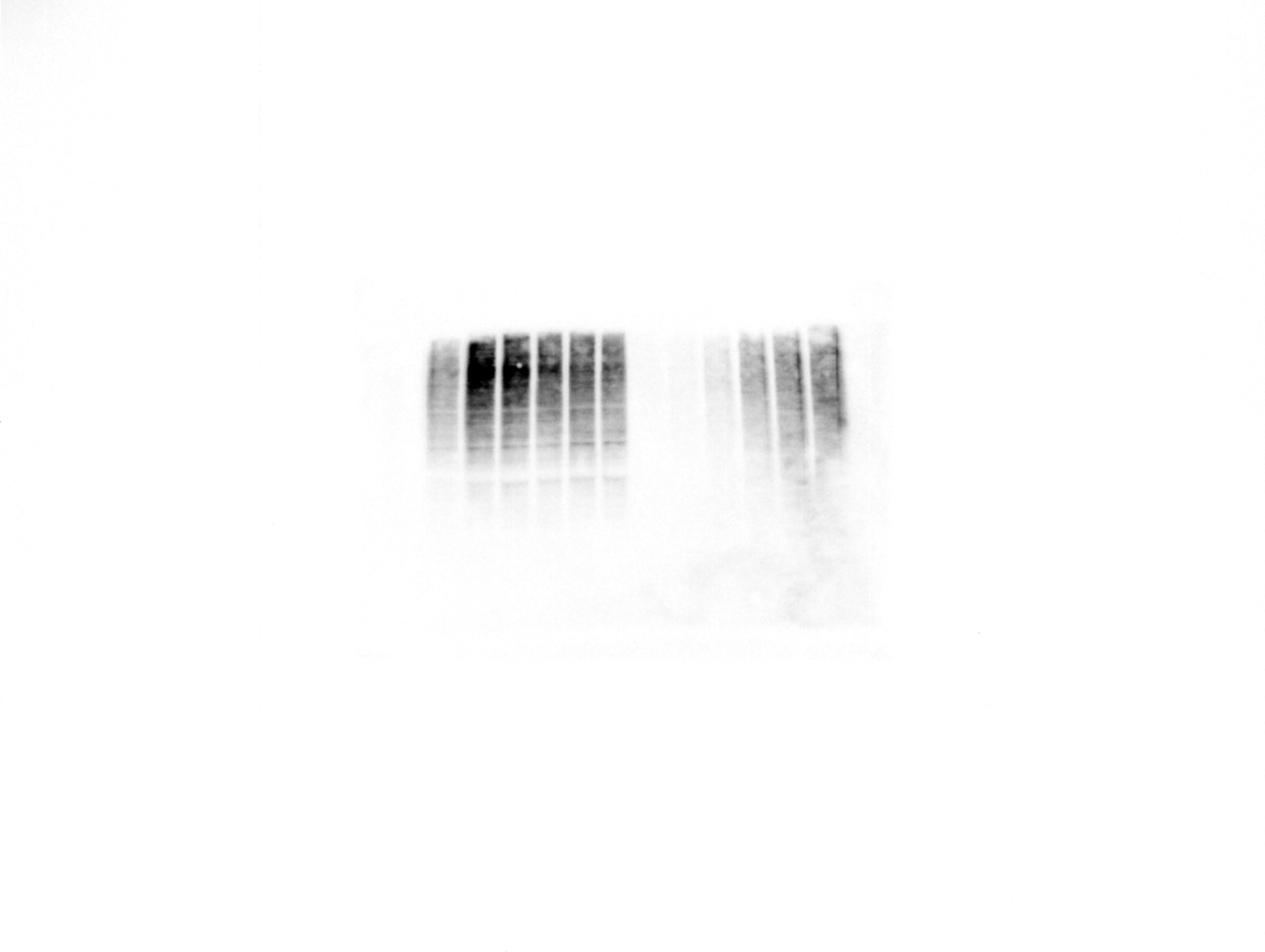

Supplement: Supplementary file 3 — Source data Fig. 2 [file 44318_2025_545_MOESM3_ESM.zip › Fig 2/2F/2F HeLa/31012024 HeLa BTZ TC Ub/2024-0131-105834_pub.tif]

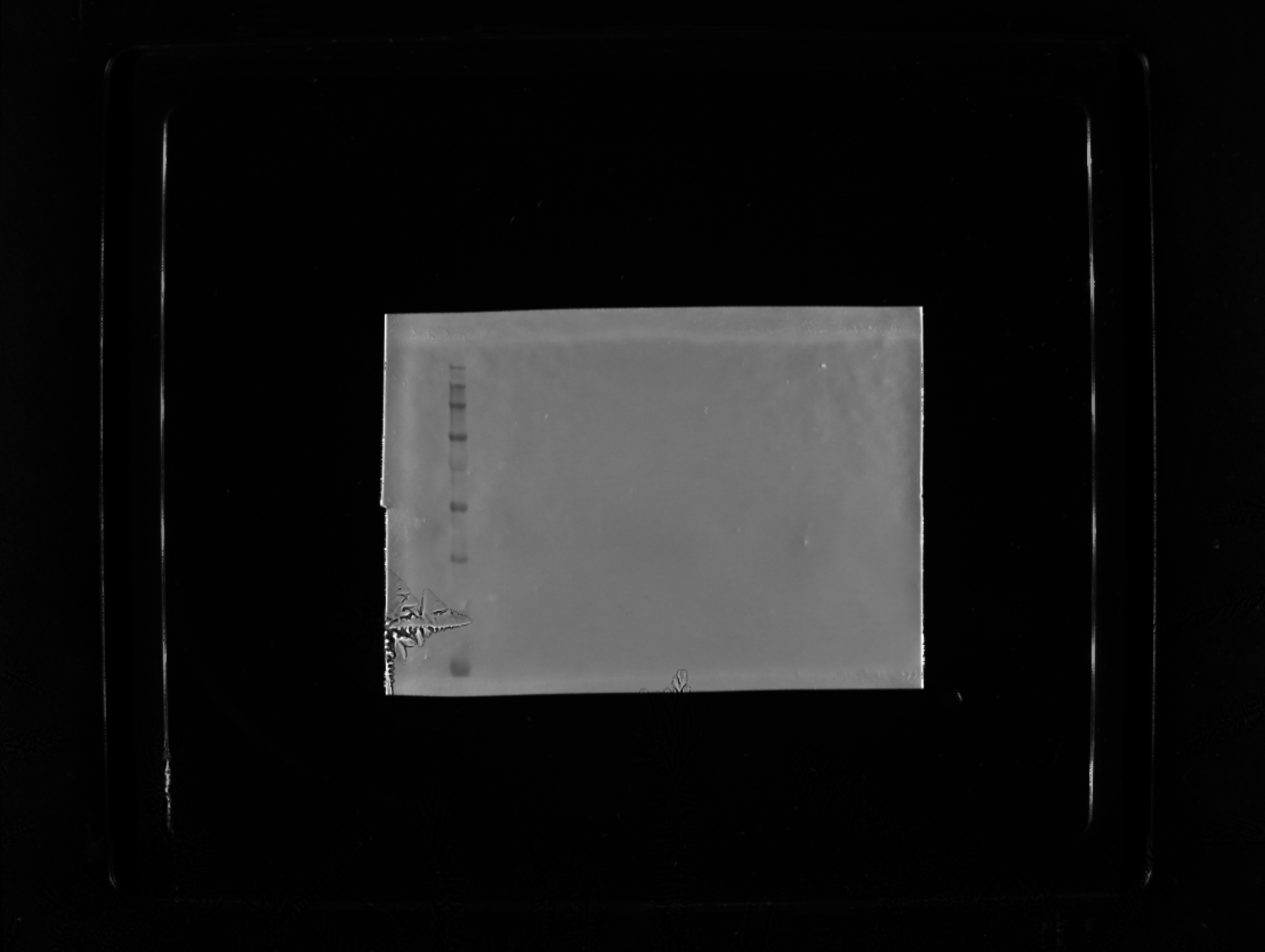

Supplement: Supplementary file 3 — Source data Fig. 2 [file 44318_2025_545_MOESM3_ESM.zip › Fig 2/2F/2F HeLa/31012024 HeLa BTZ TC Tubulin/2024-0131-111253_pub.tif]

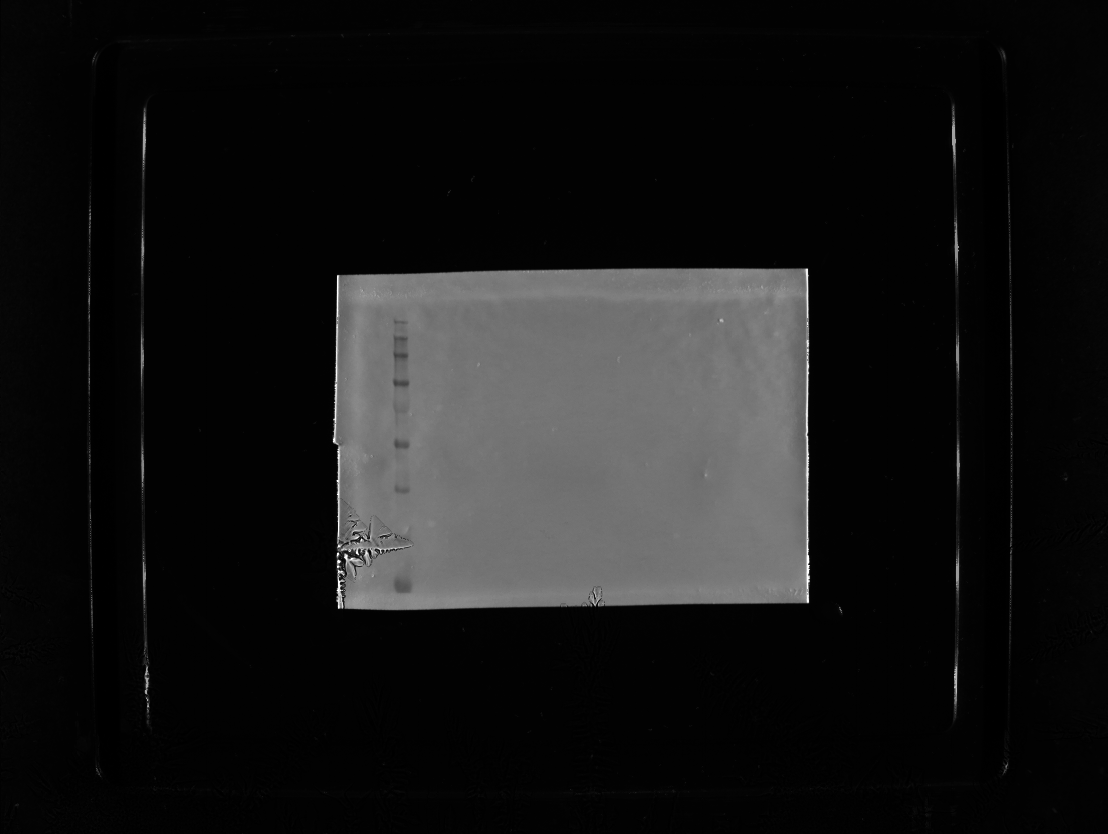

Supplement: Supplementary file 3 — Source data Fig. 2 [file 44318_2025_545_MOESM3_ESM.zip › Fig 2/2F/2F HeLa/31012024 HeLa BTZ TC Tubulin/2024-0131-111253.tif]

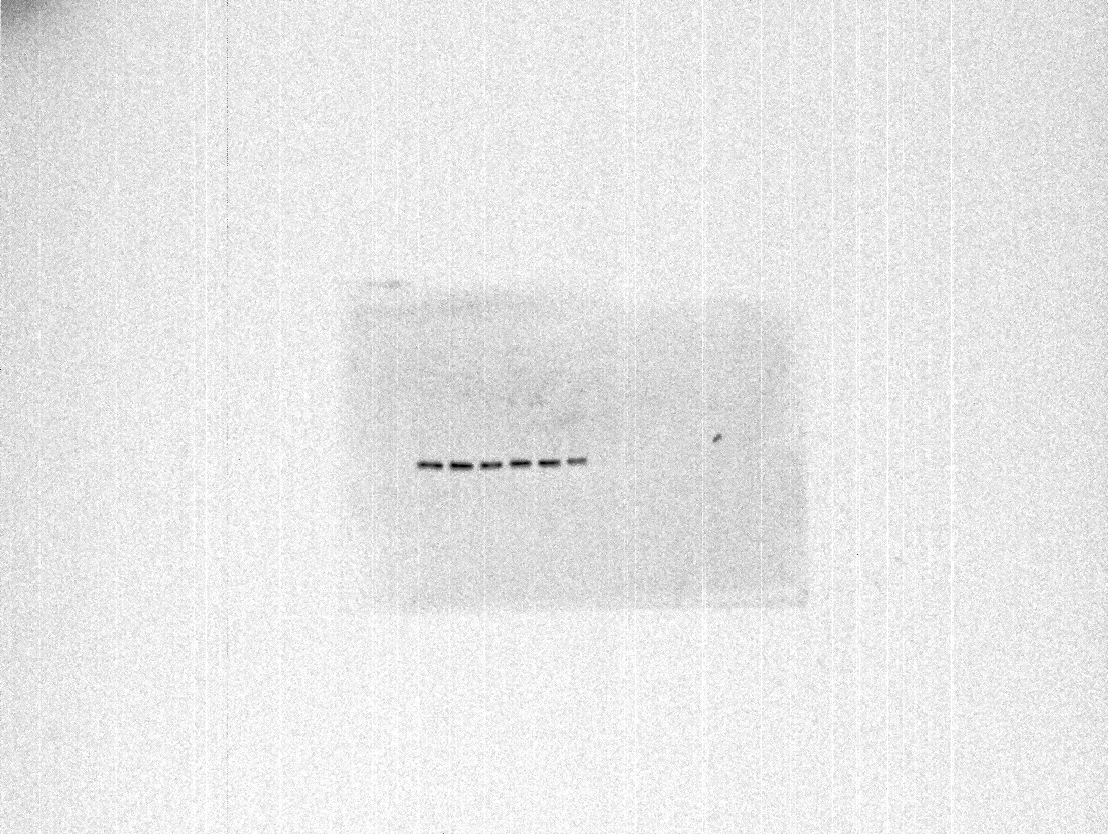

Supplement: Supplementary file 3 — Source data Fig. 2 [file 44318_2025_545_MOESM3_ESM.zip › Fig 2/2F/2F HeLa/31012024 HeLa BTZ TC Tubulin/2024-0131-111255.tif]

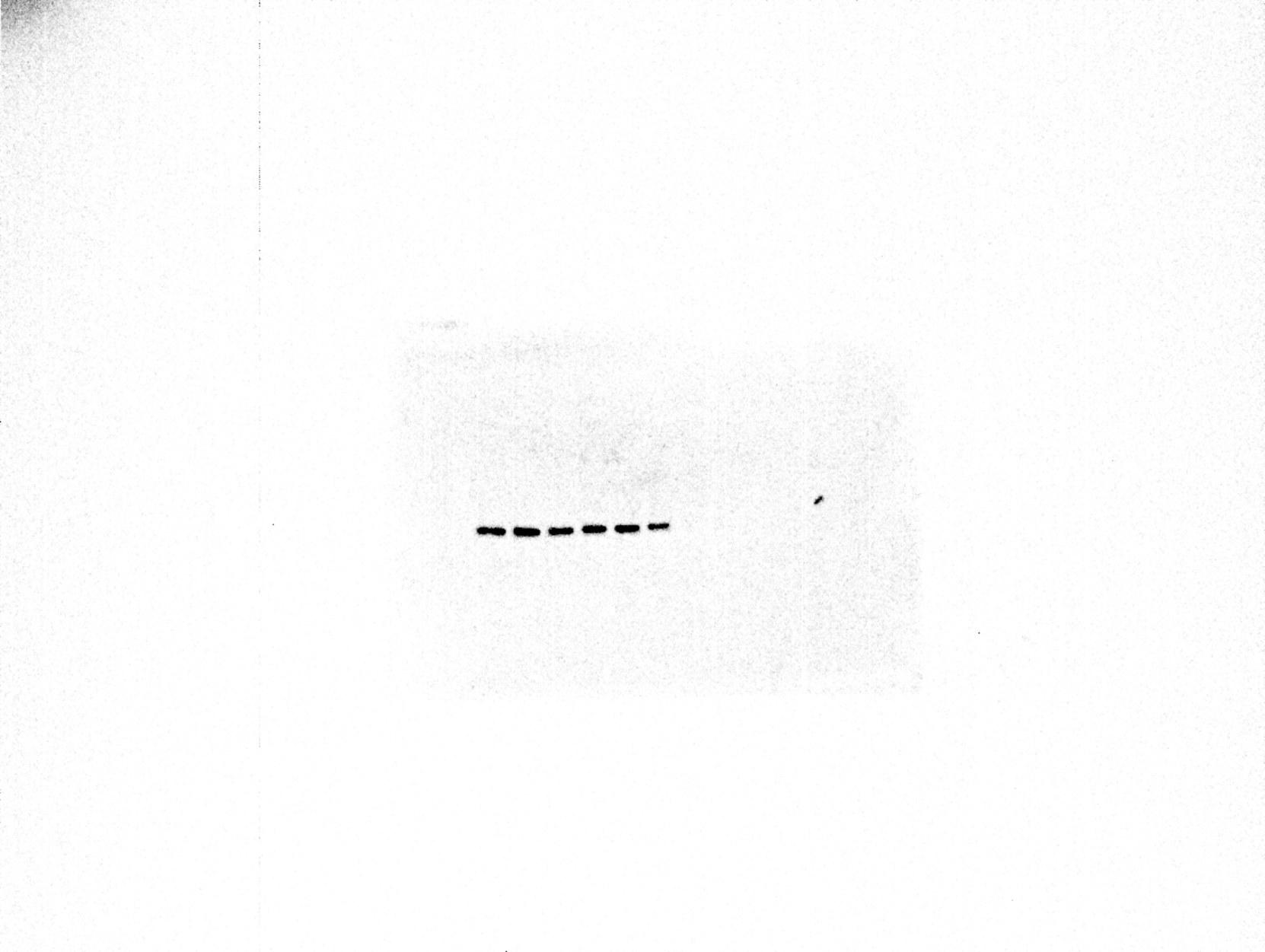

Supplement: Supplementary file 3 — Source data Fig. 2 [file 44318_2025_545_MOESM3_ESM.zip › Fig 2/2F/2F HeLa/31012024 HeLa BTZ TC Tubulin/2024-0131-111255_pub.tif]

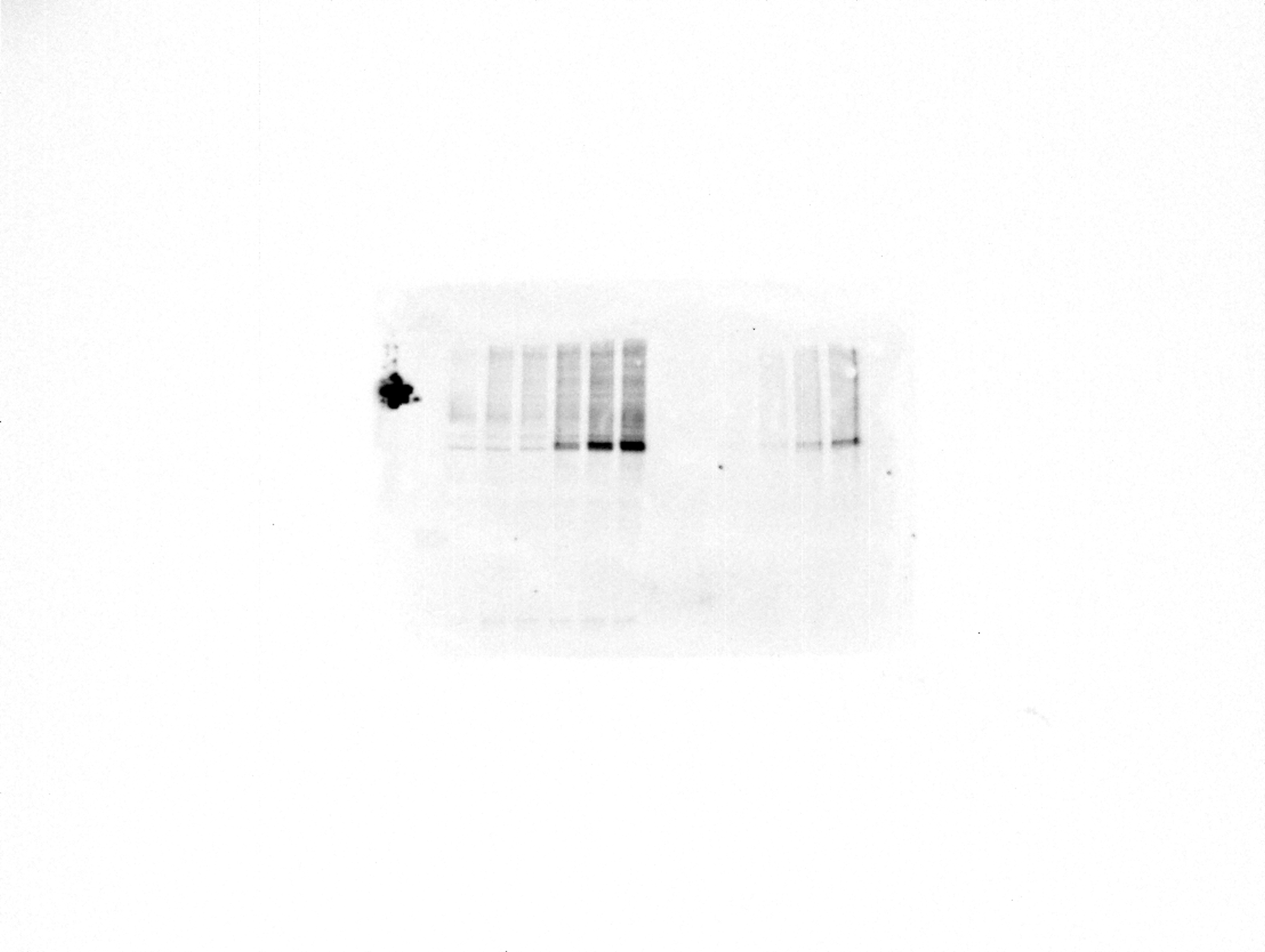

Supplement: Supplementary file 3 — Source data Fig. 2 [file 44318_2025_545_MOESM3_ESM.zip › Fig 2/2F/2F HeLa/31012024 HeLa BTZ TC ADPr/2024-0131-110506_pub.tif]

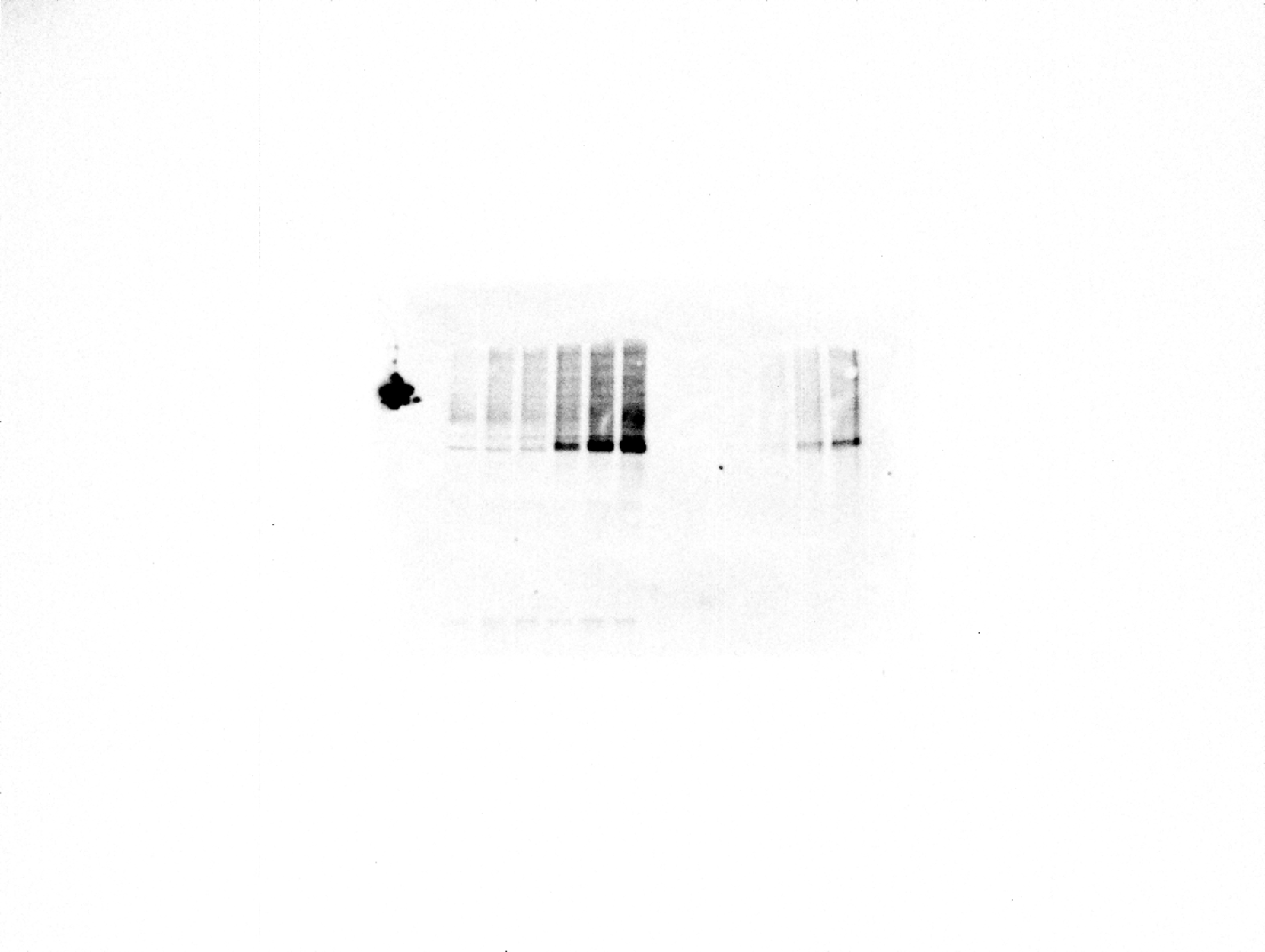

Supplement: Supplementary file 3 — Source data Fig. 2 [file 44318_2025_545_MOESM3_ESM.zip › Fig 2/2F/2F HeLa/31012024 HeLa BTZ TC ADPr/S2F1-0131-110508_pub.tif]

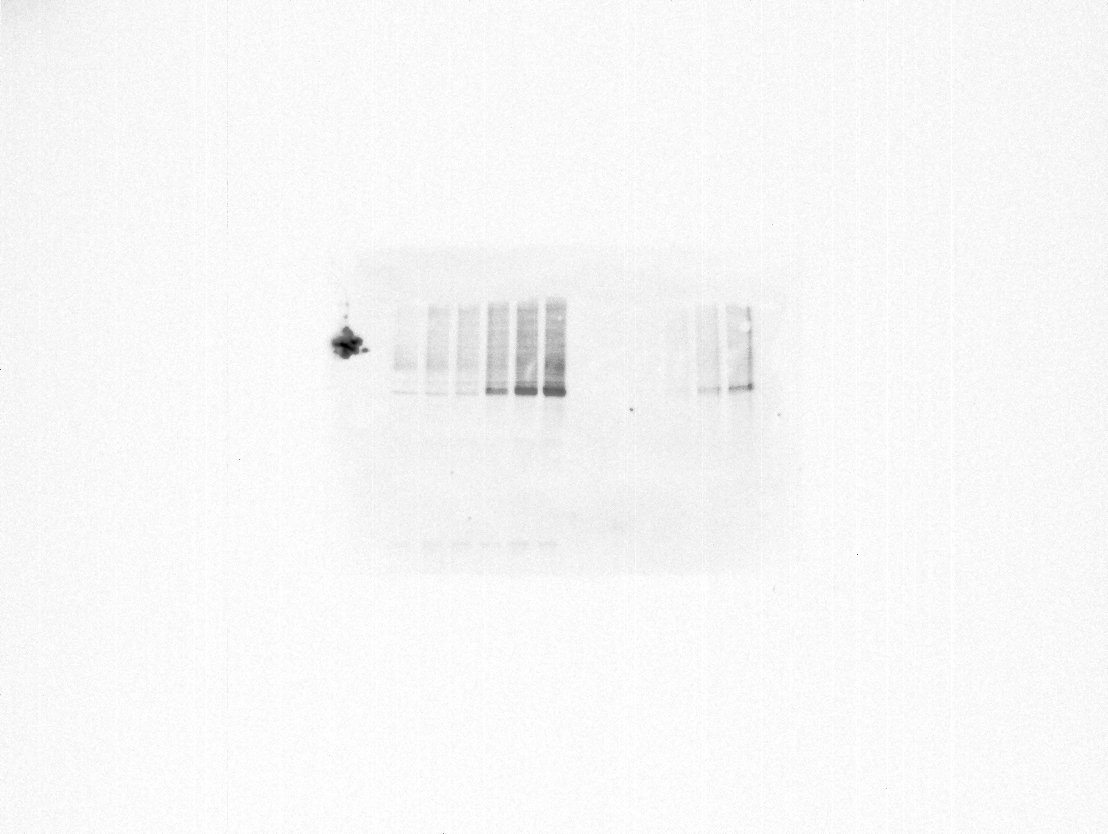

Supplement: Supplementary file 3 — Source data Fig. 2 [file 44318_2025_545_MOESM3_ESM.zip › Fig 2/2F/2F HeLa/31012024 HeLa BTZ TC ADPr/S2F1-0131-110508.tif]

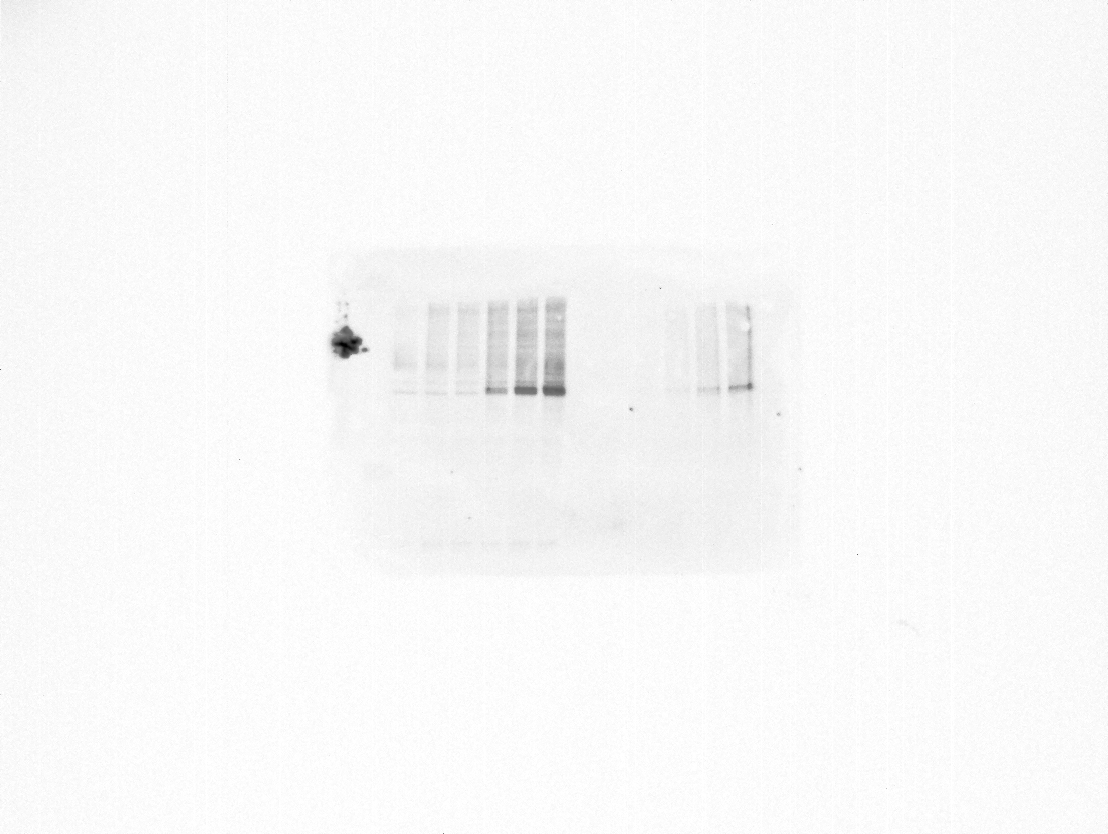

Supplement: Supplementary file 3 — Source data Fig. 2 [file 44318_2025_545_MOESM3_ESM.zip › Fig 2/2F/2F HeLa/31012024 HeLa BTZ TC ADPr/2024-0131-110506.tif]

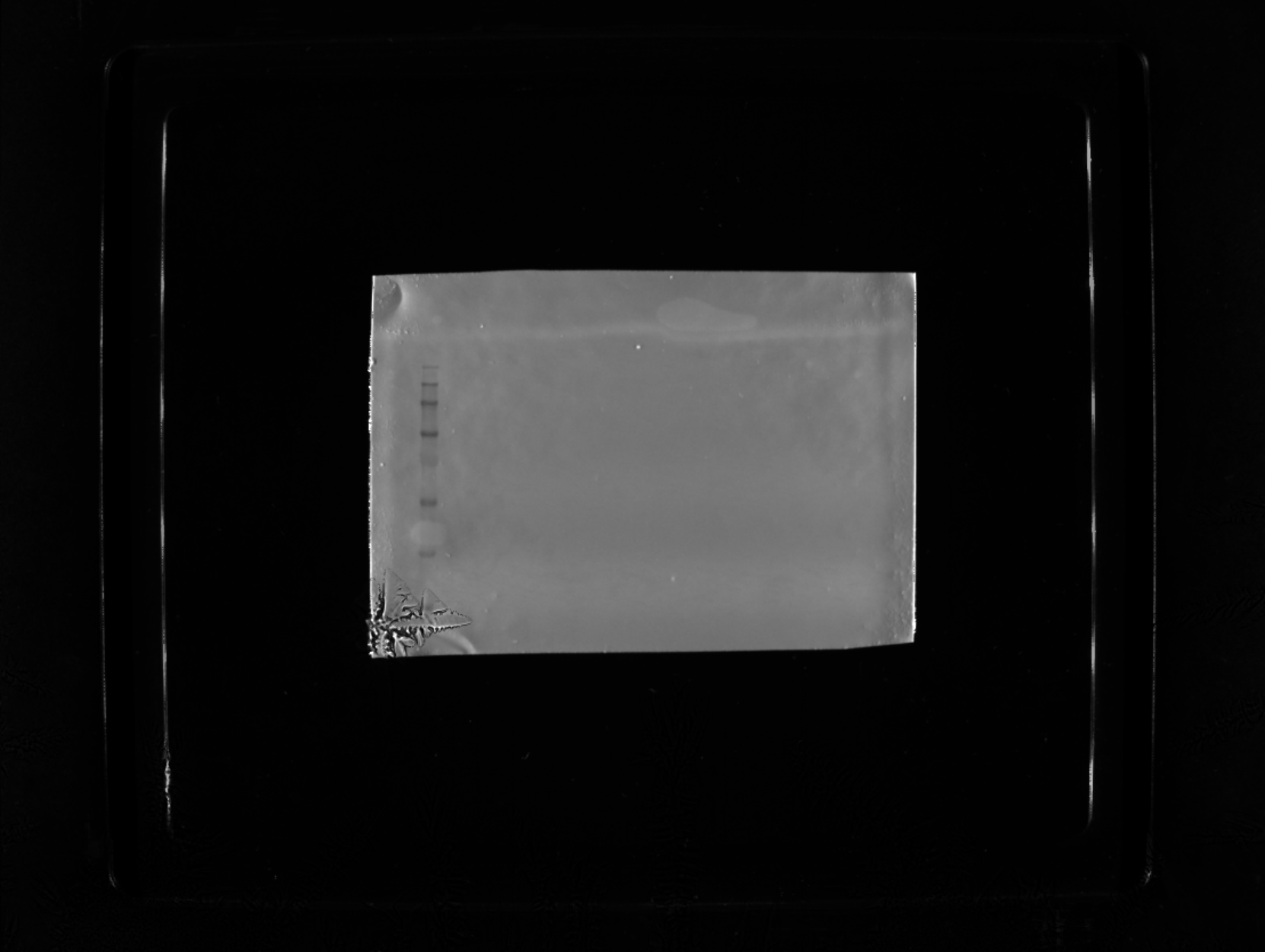

Supplement: Supplementary file 3 — Source data Fig. 2 [file 44318_2025_545_MOESM3_ESM.zip › Fig 2/2F/2F HeLa/31012024 HeLa BTZ TC ADPr/2024-0131-110505_pub.tif]

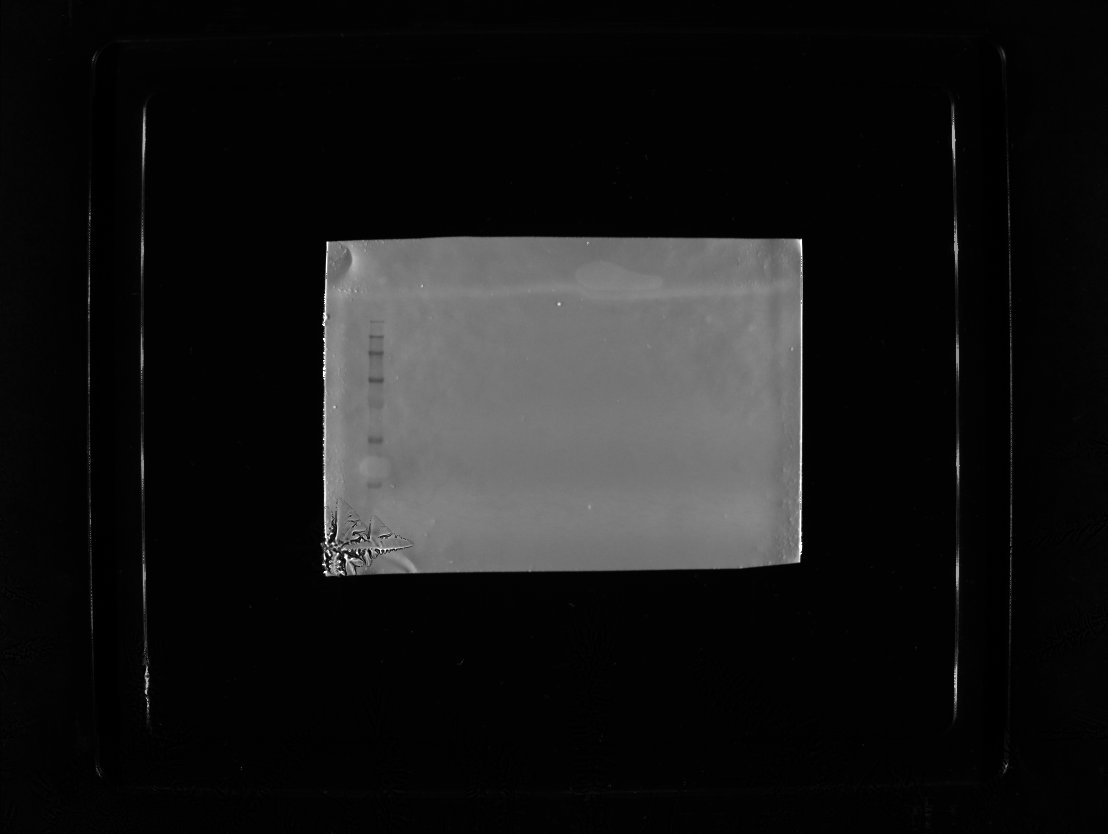

Supplement: Supplementary file 3 — Source data Fig. 2 [file 44318_2025_545_MOESM3_ESM.zip › Fig 2/2F/2F HeLa/31012024 HeLa BTZ TC ADPr/2024-0131-110505.tif]

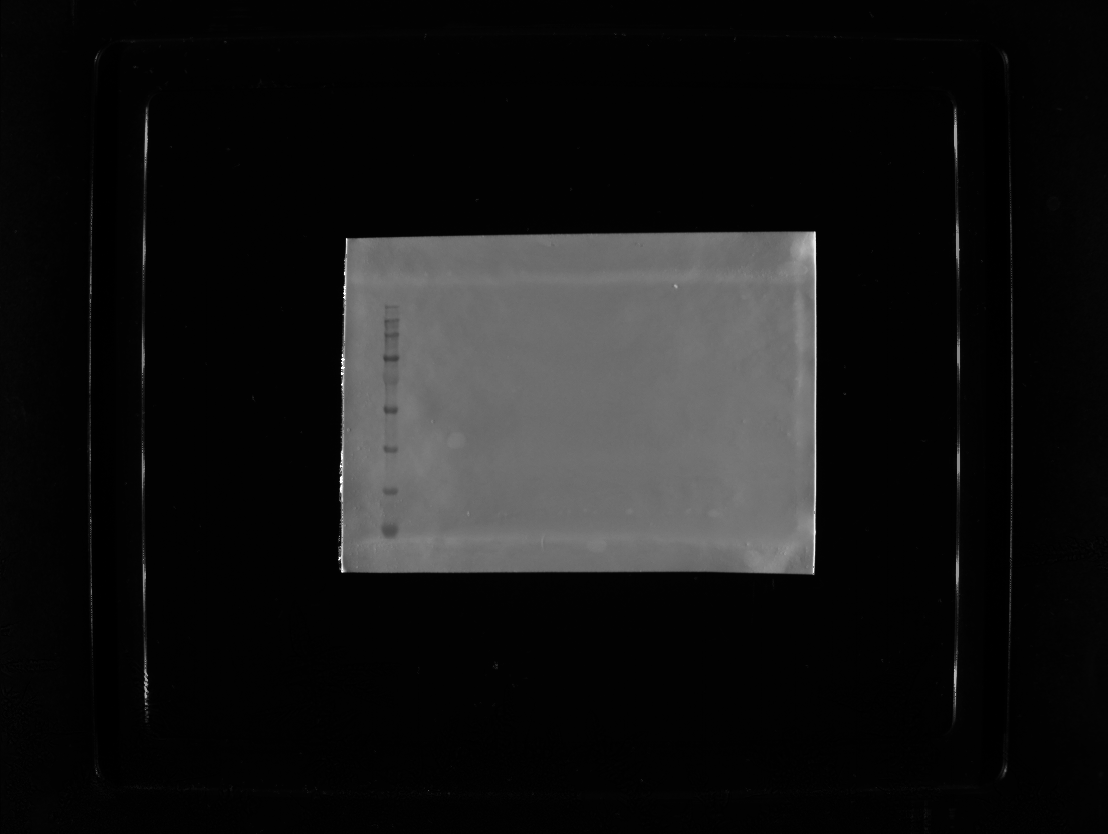

Supplement: Supplementary file 3 — Source data Fig. 2 [file 44318_2025_545_MOESM3_ESM.zip › Fig 2/2F/2F U2OS/24012024 U2OS BTZ TC Ub/2024-0124-112915.tif]

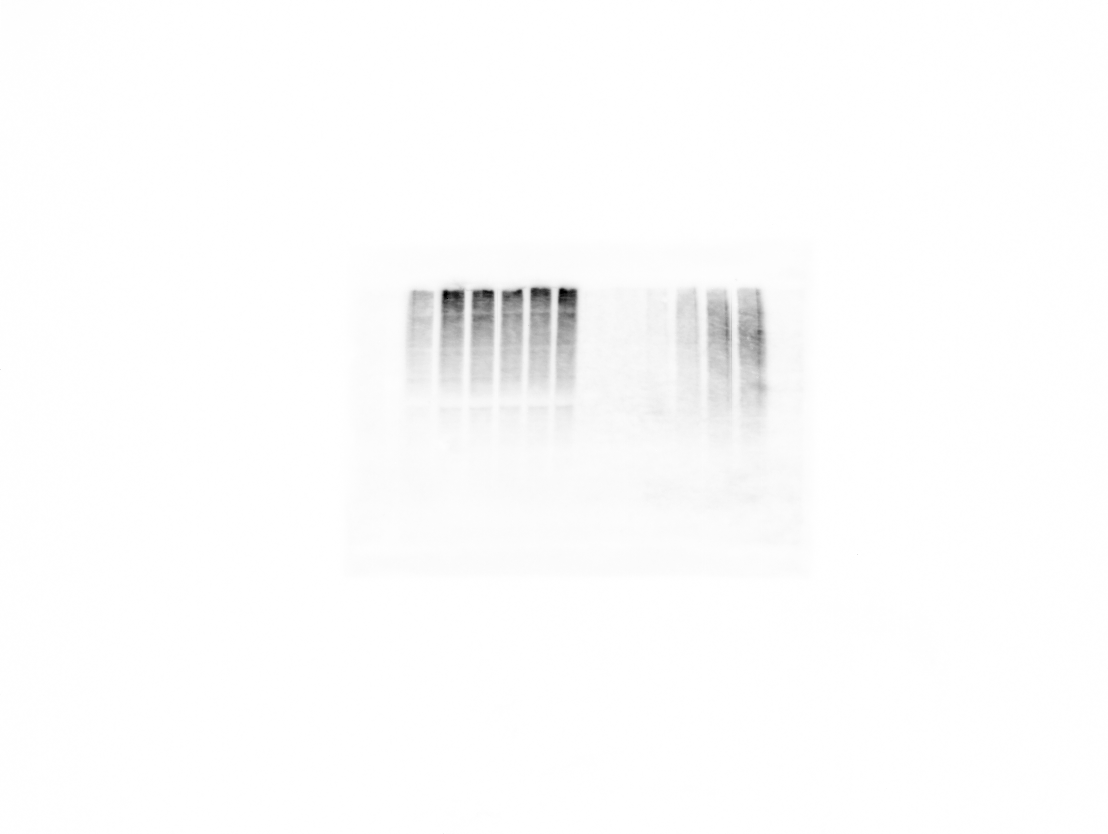

Supplement: Supplementary file 3 — Source data Fig. 2 [file 44318_2025_545_MOESM3_ESM.zip › Fig 2/2F/2F U2OS/24012024 U2OS BTZ TC Ub/2024-0124-112916.tif]

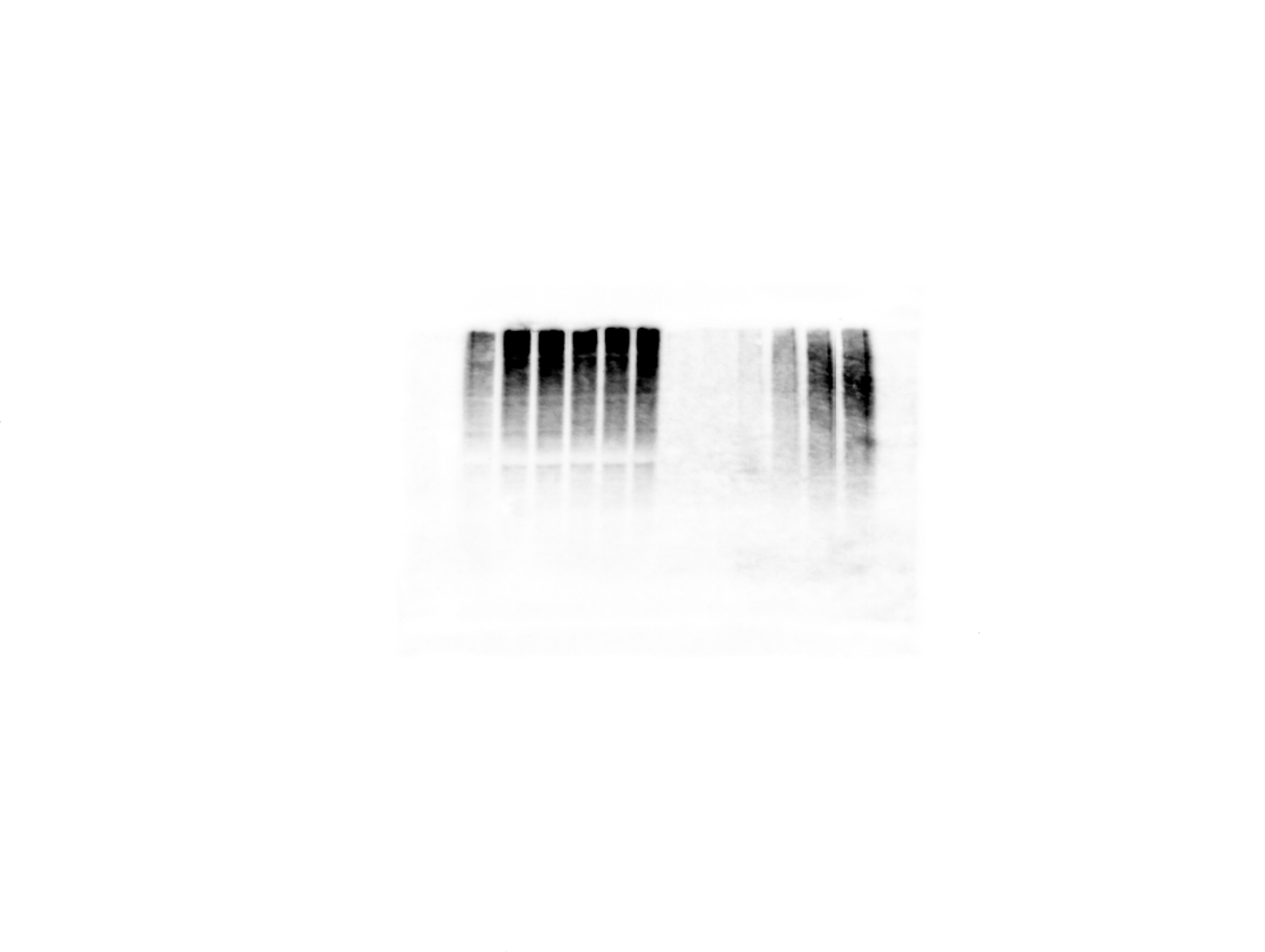

Supplement: Supplementary file 3 — Source data Fig. 2 [file 44318_2025_545_MOESM3_ESM.zip › Fig 2/2F/2F U2OS/24012024 U2OS BTZ TC Ub/2024-0124-112916_pub.tif]

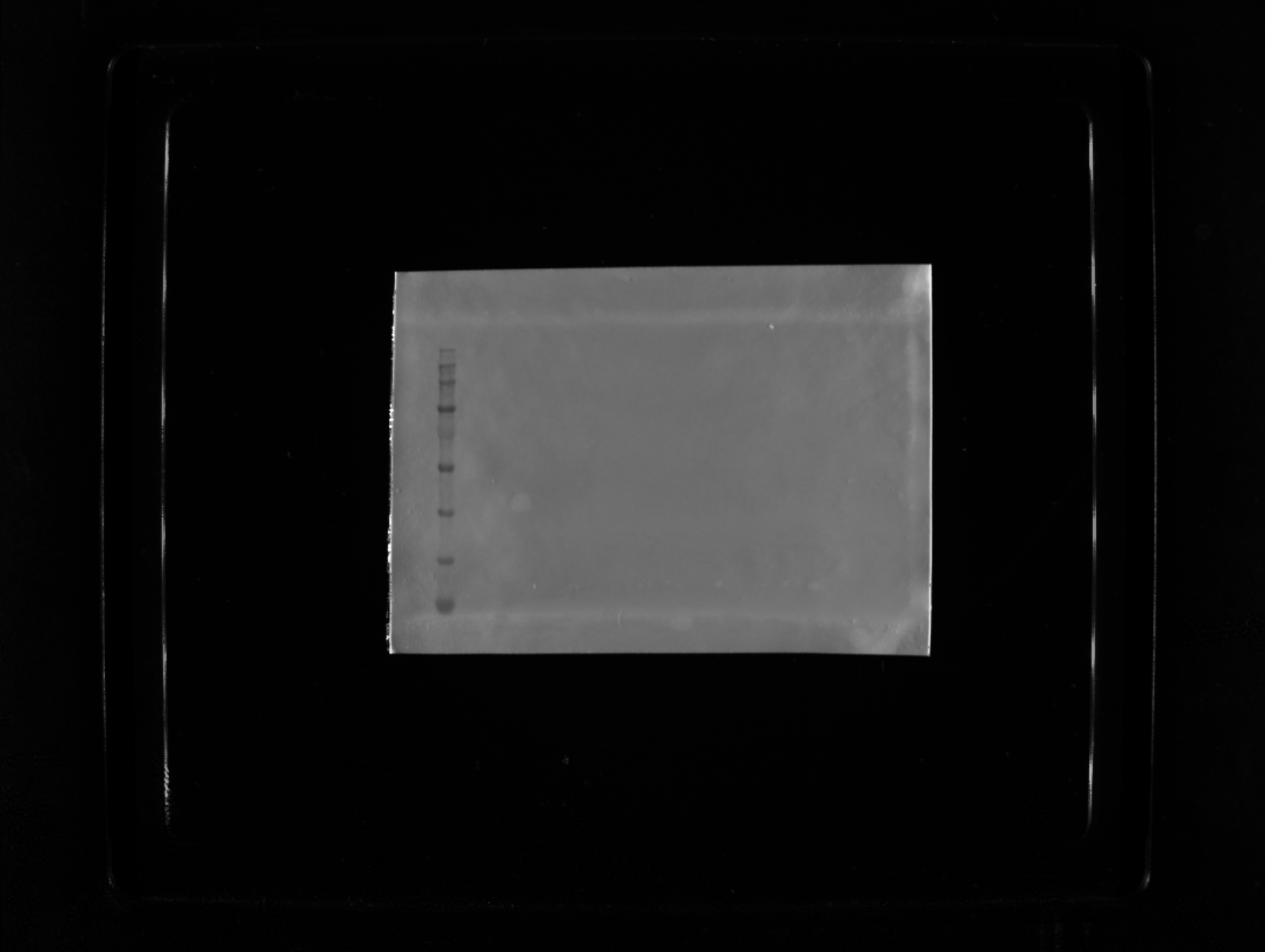

Supplement: Supplementary file 3 — Source data Fig. 2 [file 44318_2025_545_MOESM3_ESM.zip › Fig 2/2F/2F U2OS/24012024 U2OS BTZ TC Ub/2024-0124-112915_pub.tif]

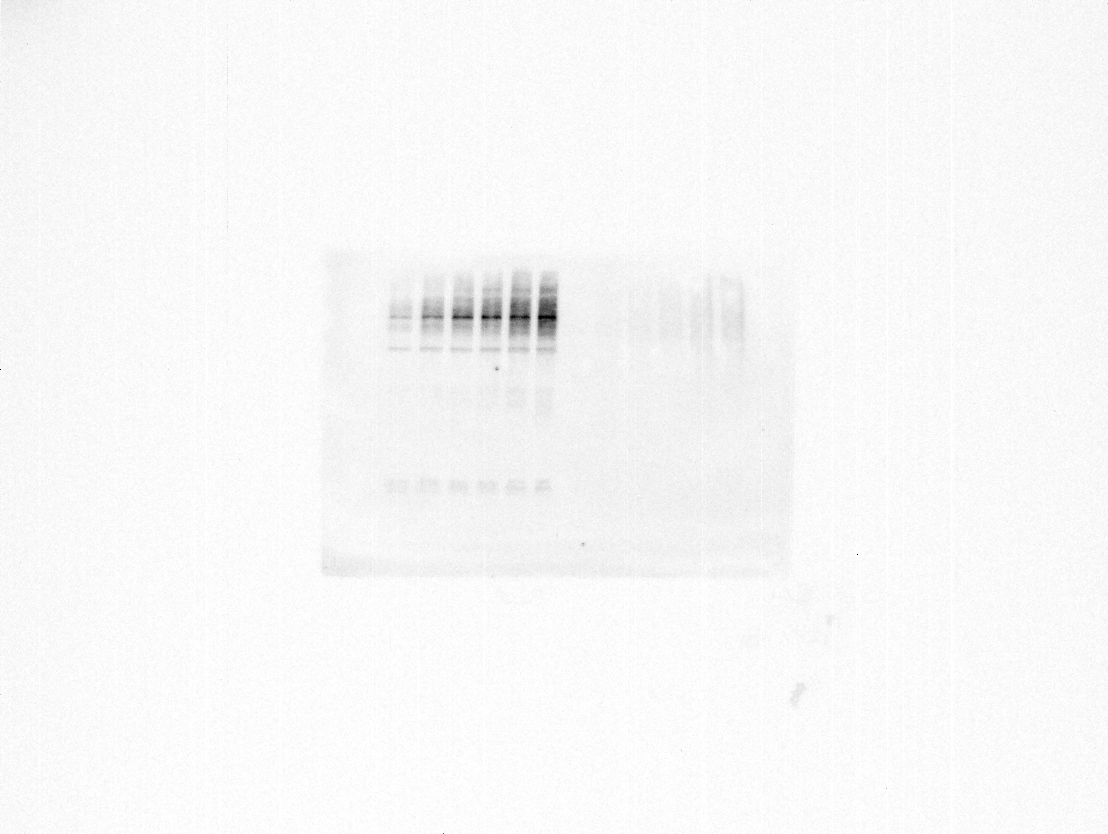

Supplement: Supplementary file 3 — Source data Fig. 2 [file 44318_2025_545_MOESM3_ESM.zip › Fig 2/2F/2F U2OS/24012024 U2OS BTZ TC CST/S1F1-0124-112453.tif]

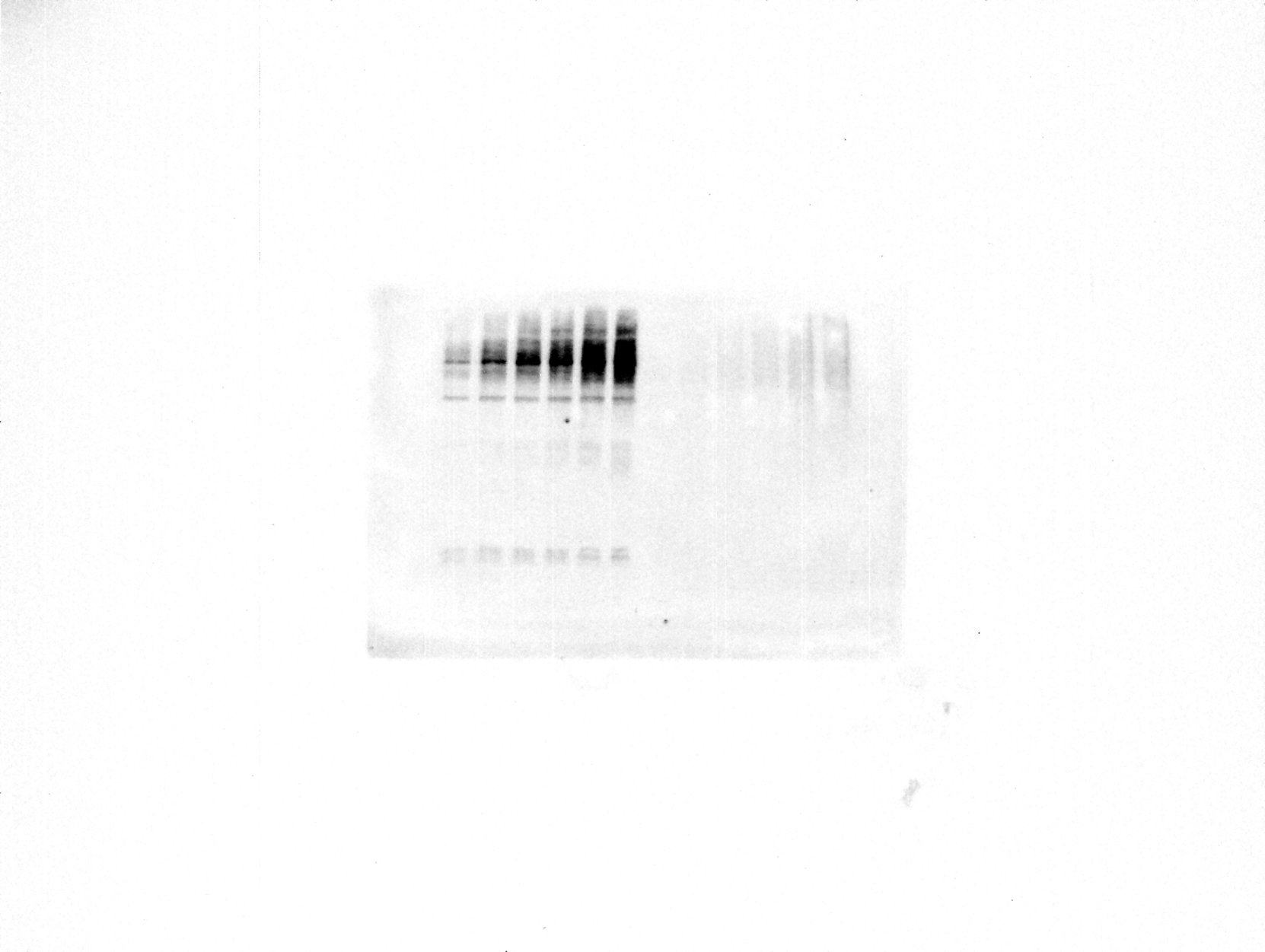

Supplement: Supplementary file 3 — Source data Fig. 2 [file 44318_2025_545_MOESM3_ESM.zip › Fig 2/2F/2F U2OS/24012024 U2OS BTZ TC CST/S1F2-0124-112454_pub.tif]

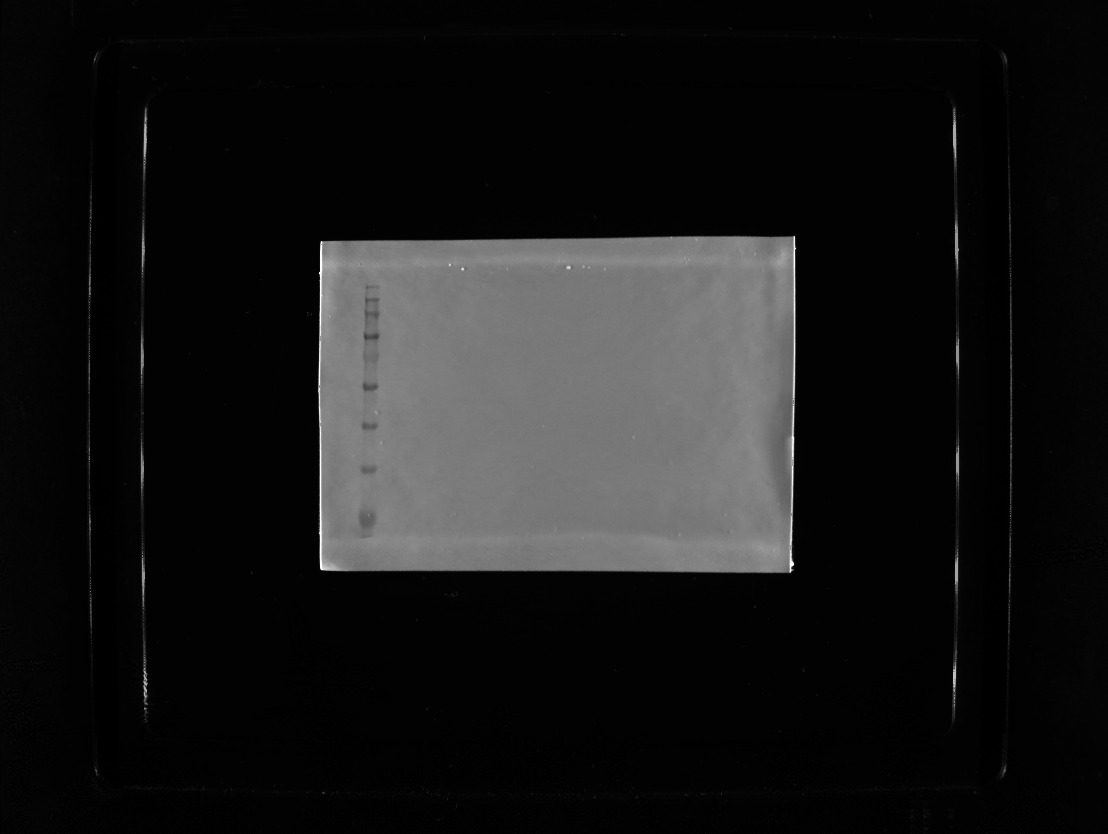

Supplement: Supplementary file 3 — Source data Fig. 2 [file 44318_2025_545_MOESM3_ESM.zip › Fig 2/2F/2F U2OS/24012024 U2OS BTZ TC CST/2024-0124-112451.tif]

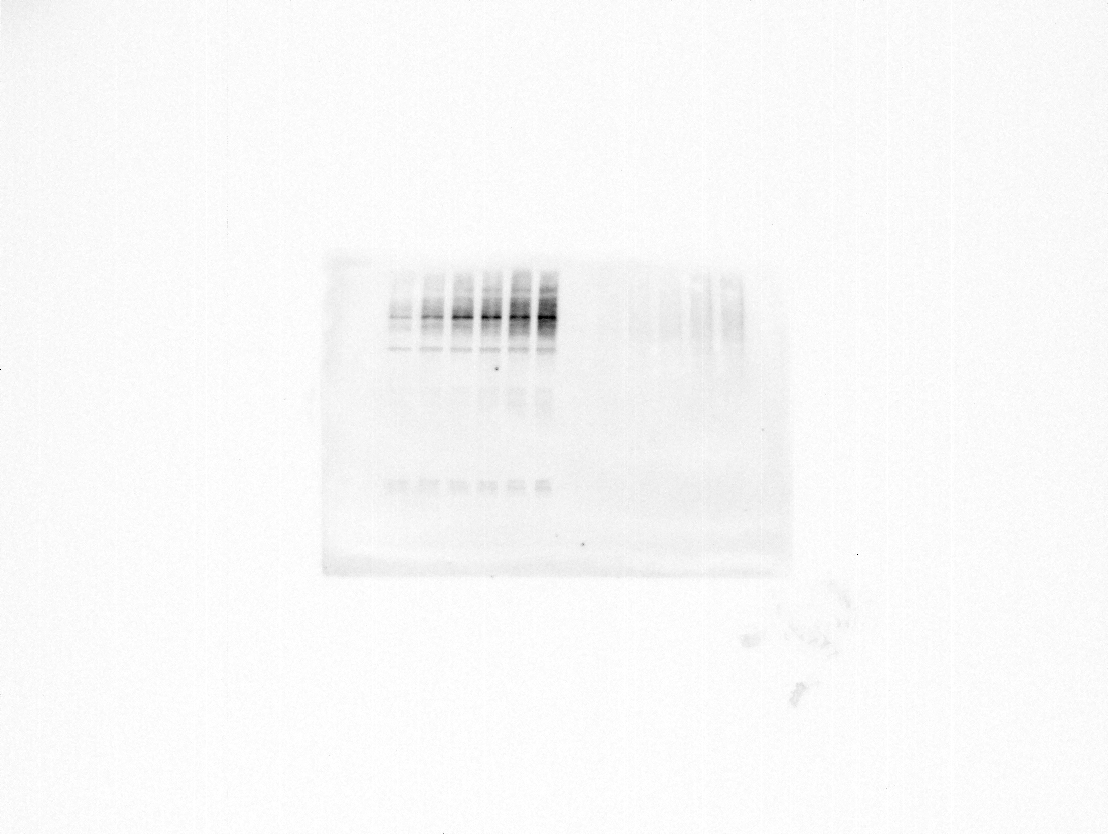

Supplement: Supplementary file 3 — Source data Fig. 2 [file 44318_2025_545_MOESM3_ESM.zip › Fig 2/2F/2F U2OS/24012024 U2OS BTZ TC CST/2024-0124-112452.tif]

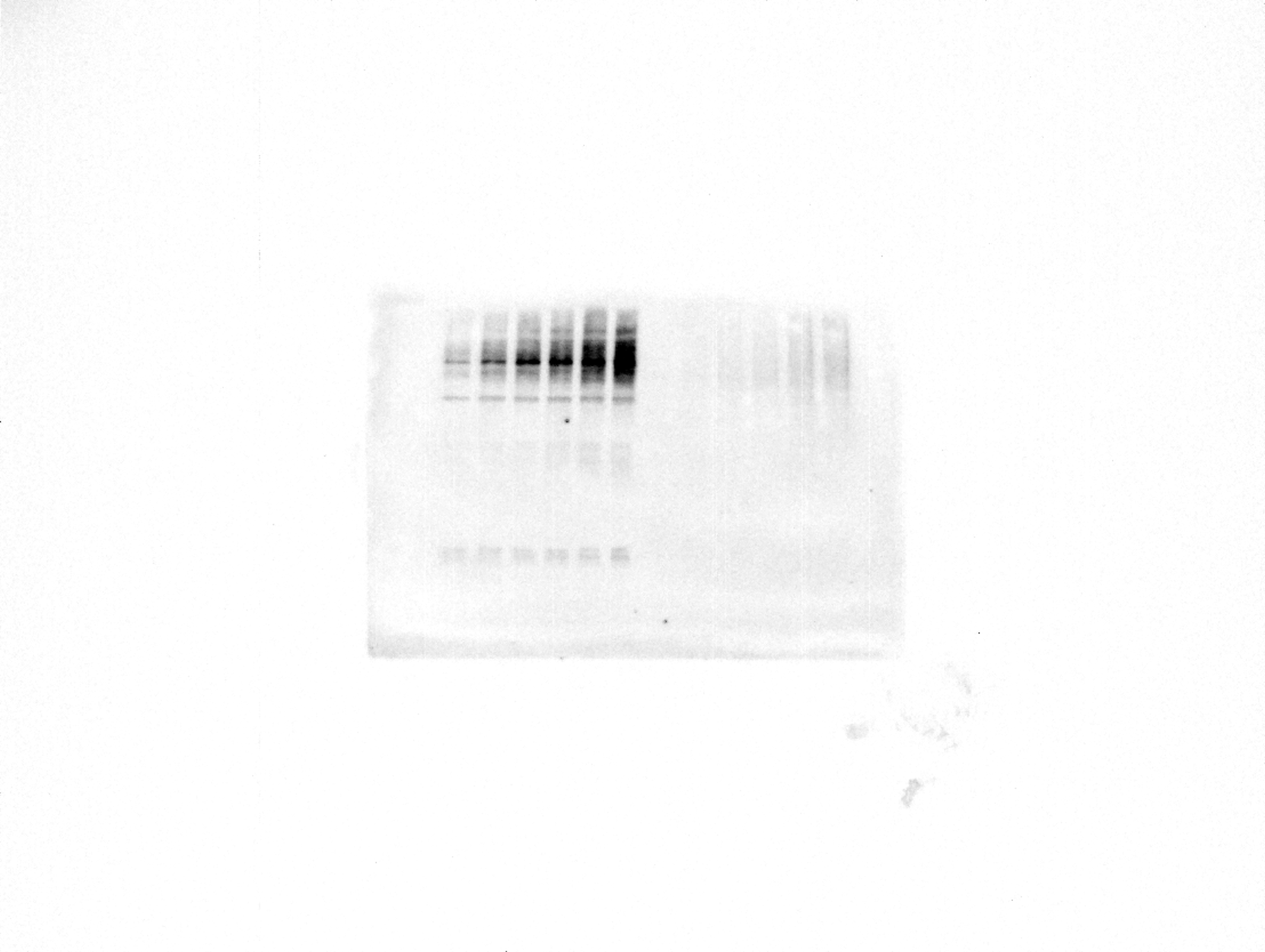

Supplement: Supplementary file 3 — Source data Fig. 2 [file 44318_2025_545_MOESM3_ESM.zip › Fig 2/2F/2F U2OS/24012024 U2OS BTZ TC CST/2024-0124-112452_pub.tif]

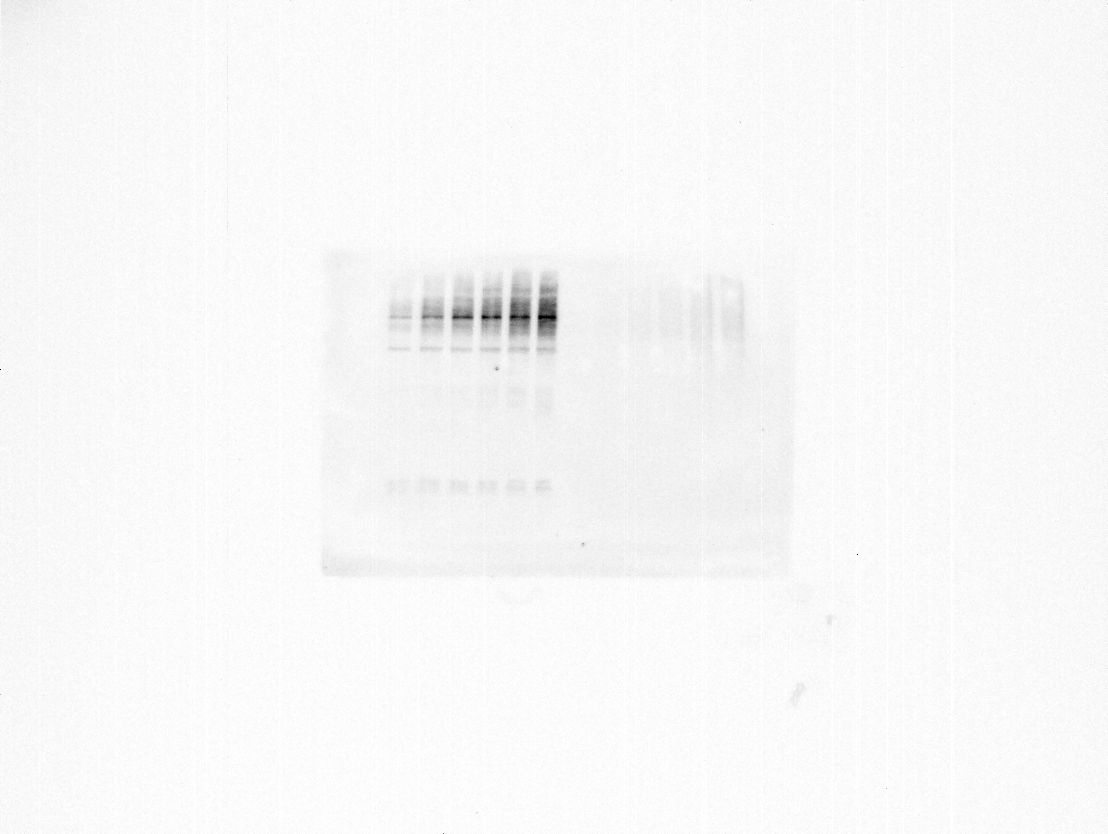

Supplement: Supplementary file 3 — Source data Fig. 2 [file 44318_2025_545_MOESM3_ESM.zip › Fig 2/2F/2F U2OS/24012024 U2OS BTZ TC CST/S1F2-0124-112454.tif]

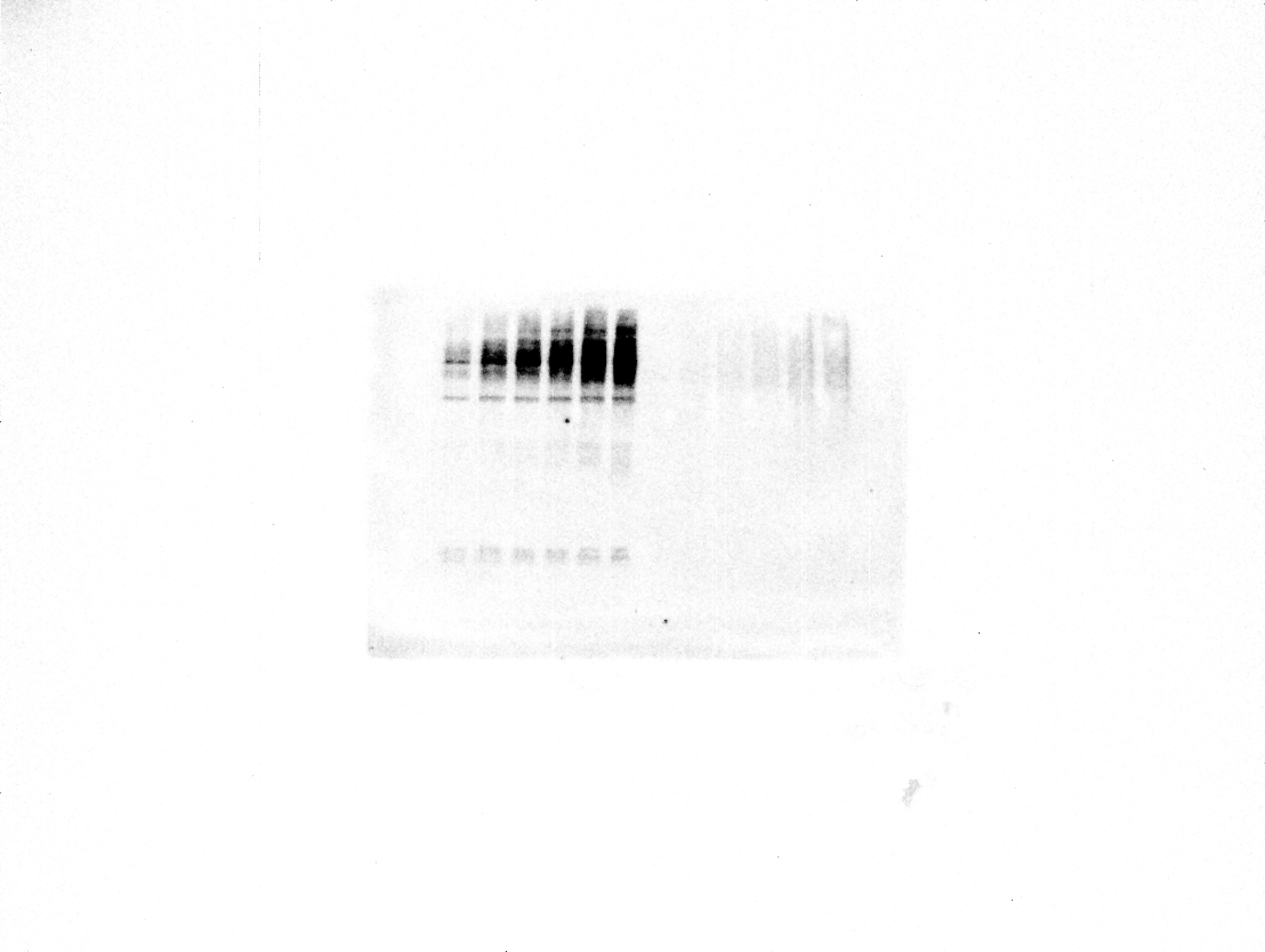

Supplement: Supplementary file 3 — Source data Fig. 2 [file 44318_2025_545_MOESM3_ESM.zip › Fig 2/2F/2F U2OS/24012024 U2OS BTZ TC CST/S1F1-0124-112453_pub.tif]

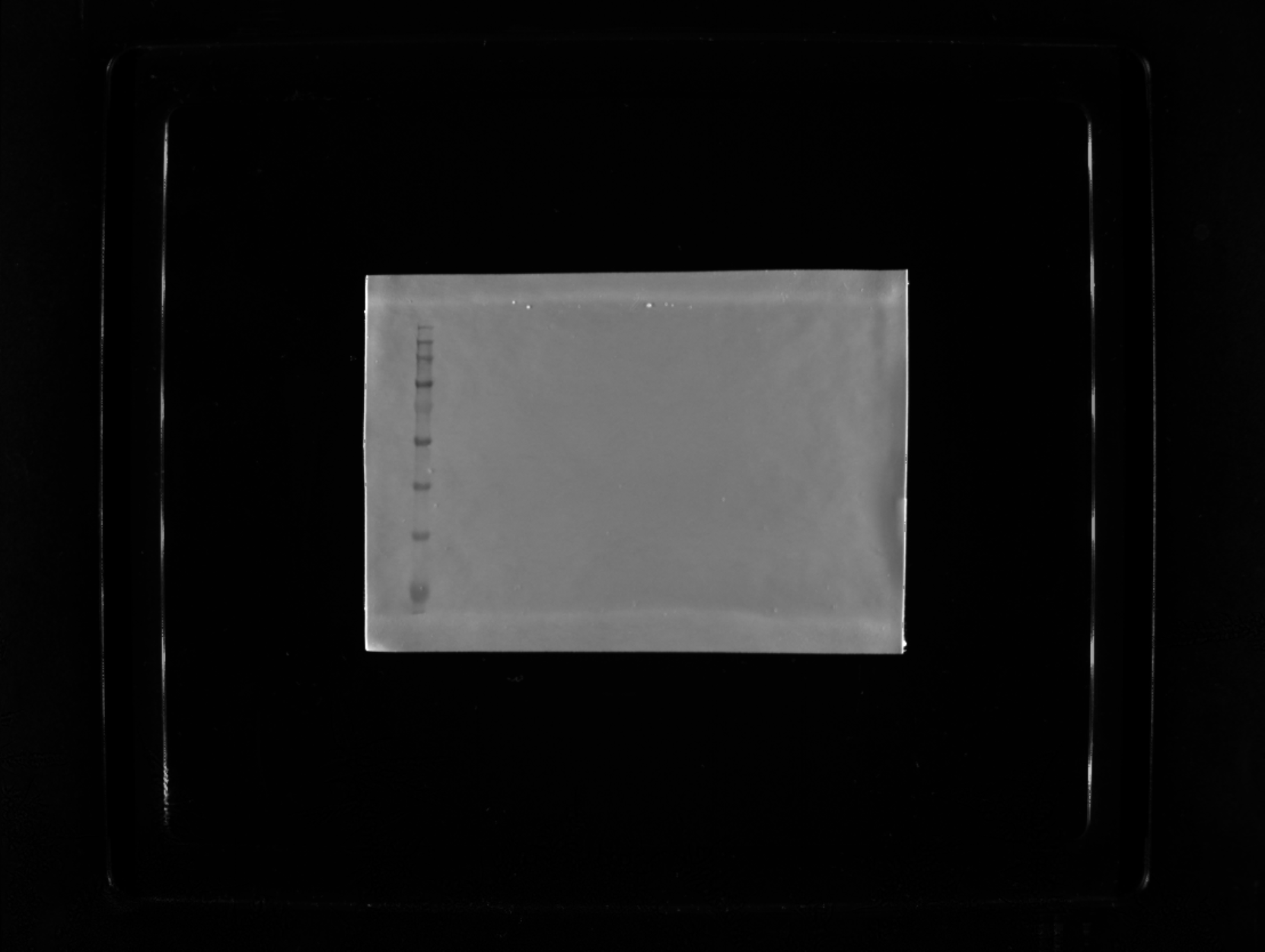

Supplement: Supplementary file 3 — Source data Fig. 2 [file 44318_2025_545_MOESM3_ESM.zip › Fig 2/2F/2F U2OS/24012024 U2OS BTZ TC CST/2024-0124-112451_pub.tif]

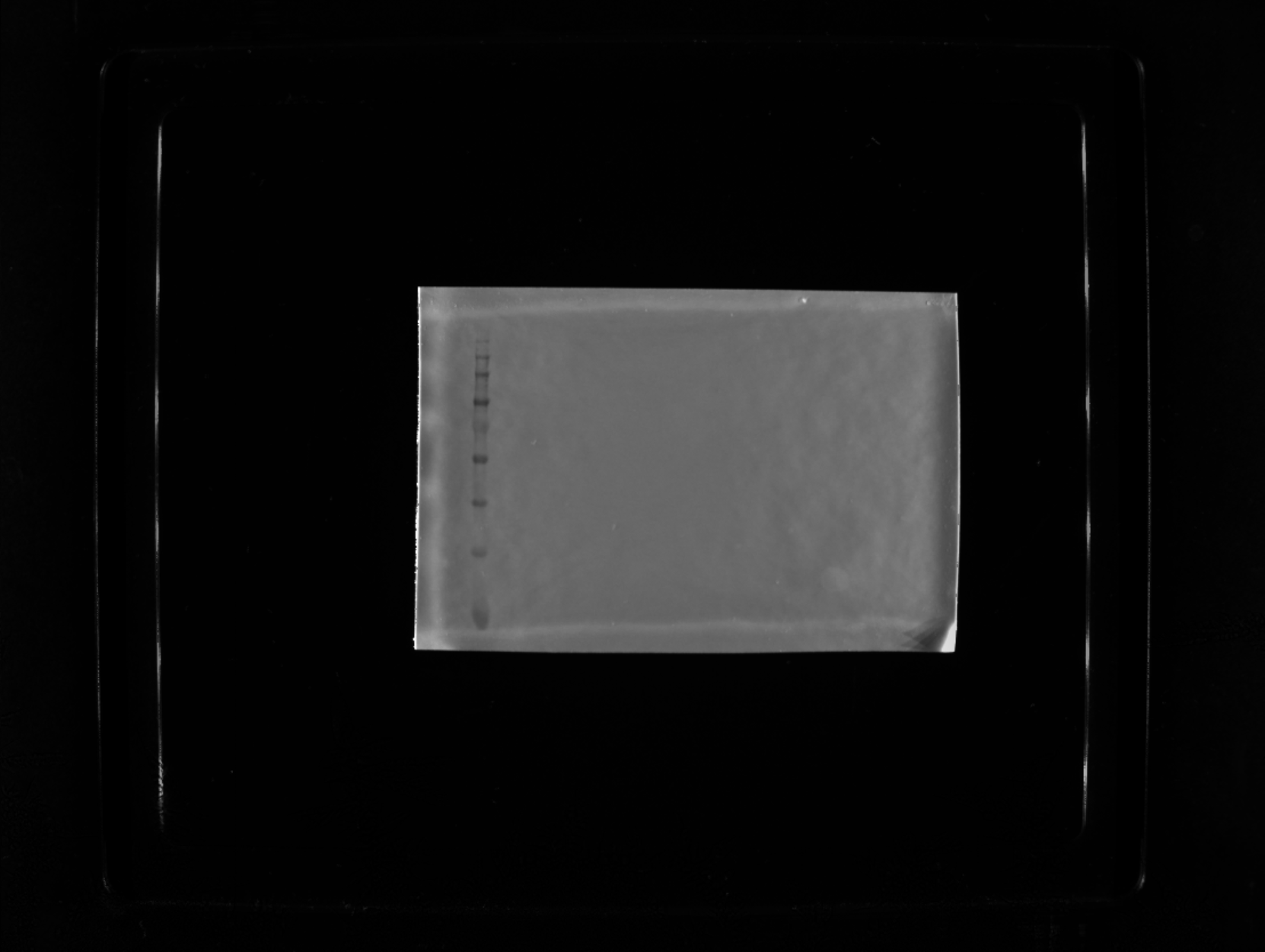

Supplement: Supplementary file 3 — Source data Fig. 2 [file 44318_2025_545_MOESM3_ESM.zip › Fig 2/2F/2F U2OS/24012024 U2OS BTZ TC Tubulin/2024-0124-113314_pub.tif]

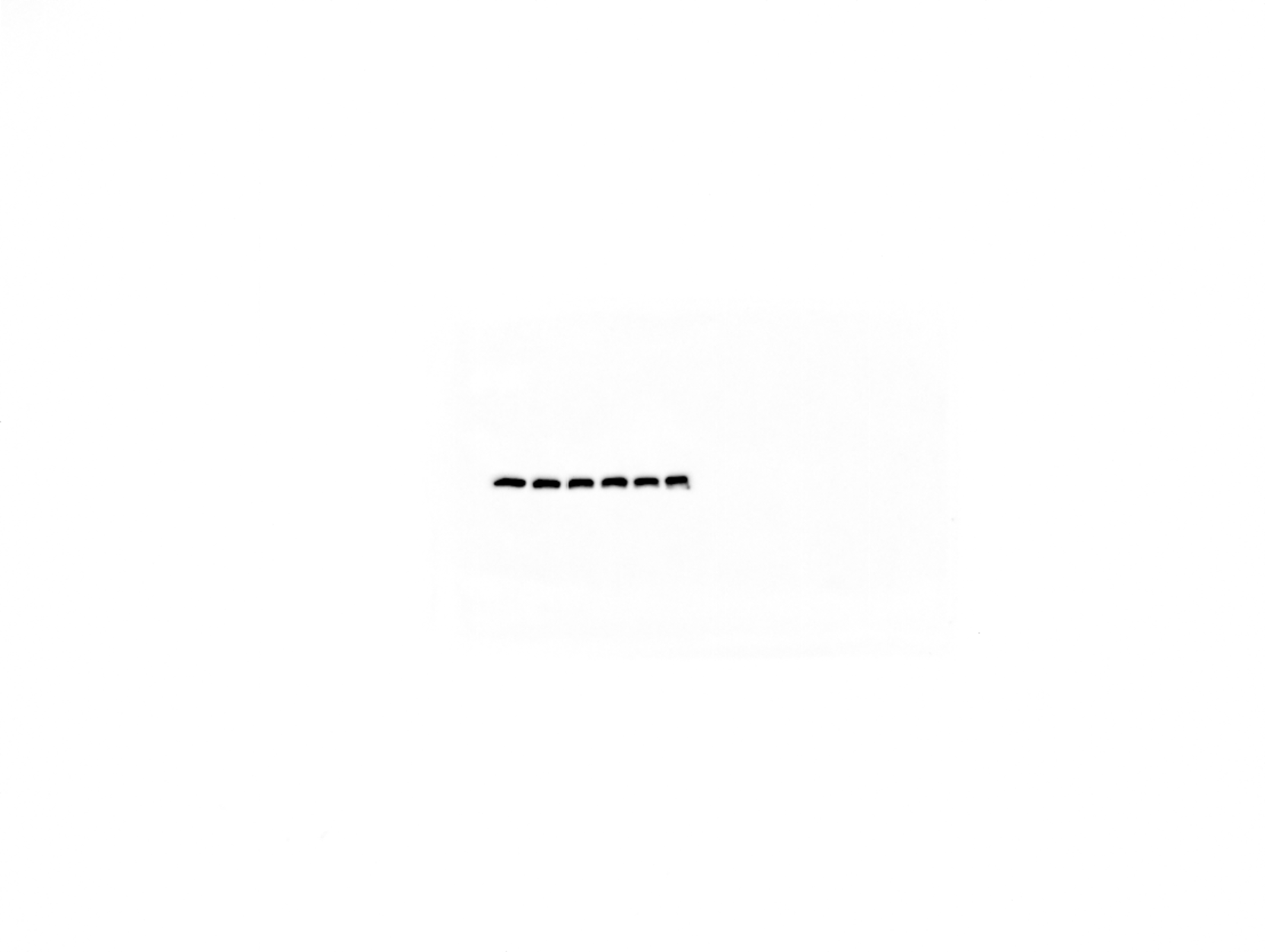

Supplement: Supplementary file 3 — Source data Fig. 2 [file 44318_2025_545_MOESM3_ESM.zip › Fig 2/2F/2F U2OS/24012024 U2OS BTZ TC Tubulin/2024-0124-113316_pub.tif]

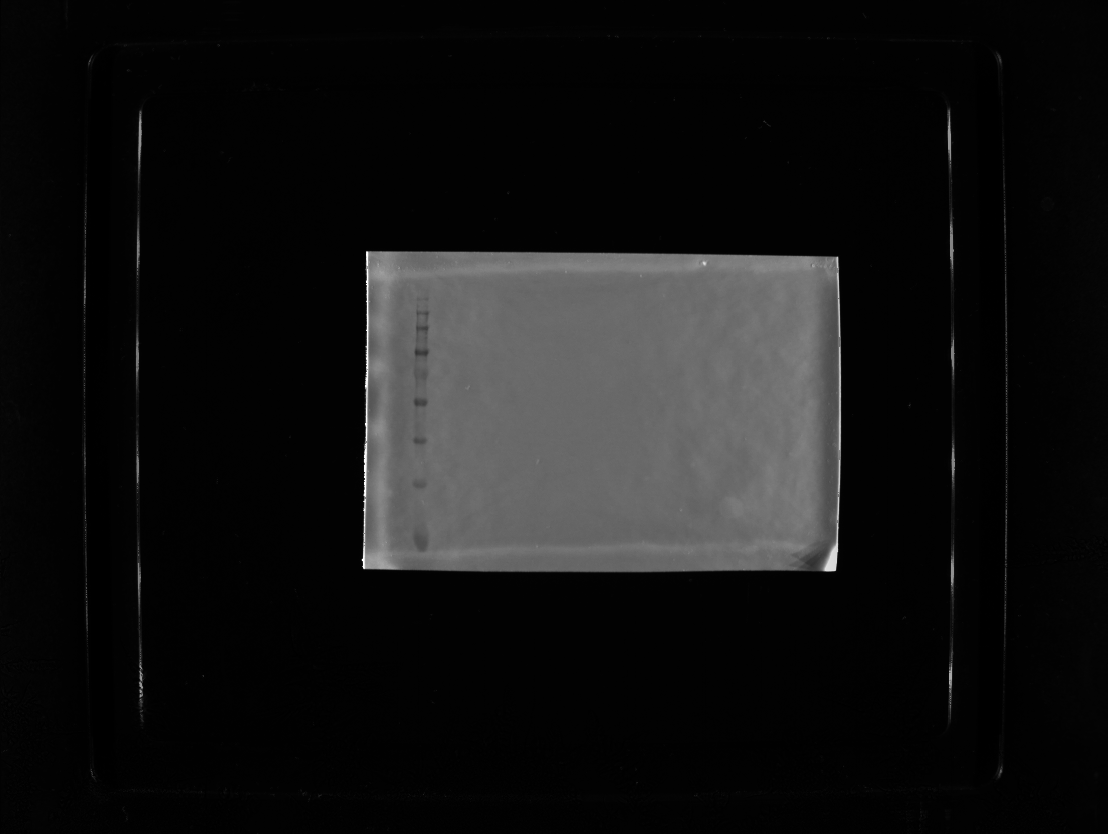

Supplement: Supplementary file 3 — Source data Fig. 2 [file 44318_2025_545_MOESM3_ESM.zip › Fig 2/2F/2F U2OS/24012024 U2OS BTZ TC Tubulin/2024-0124-113314.tif]

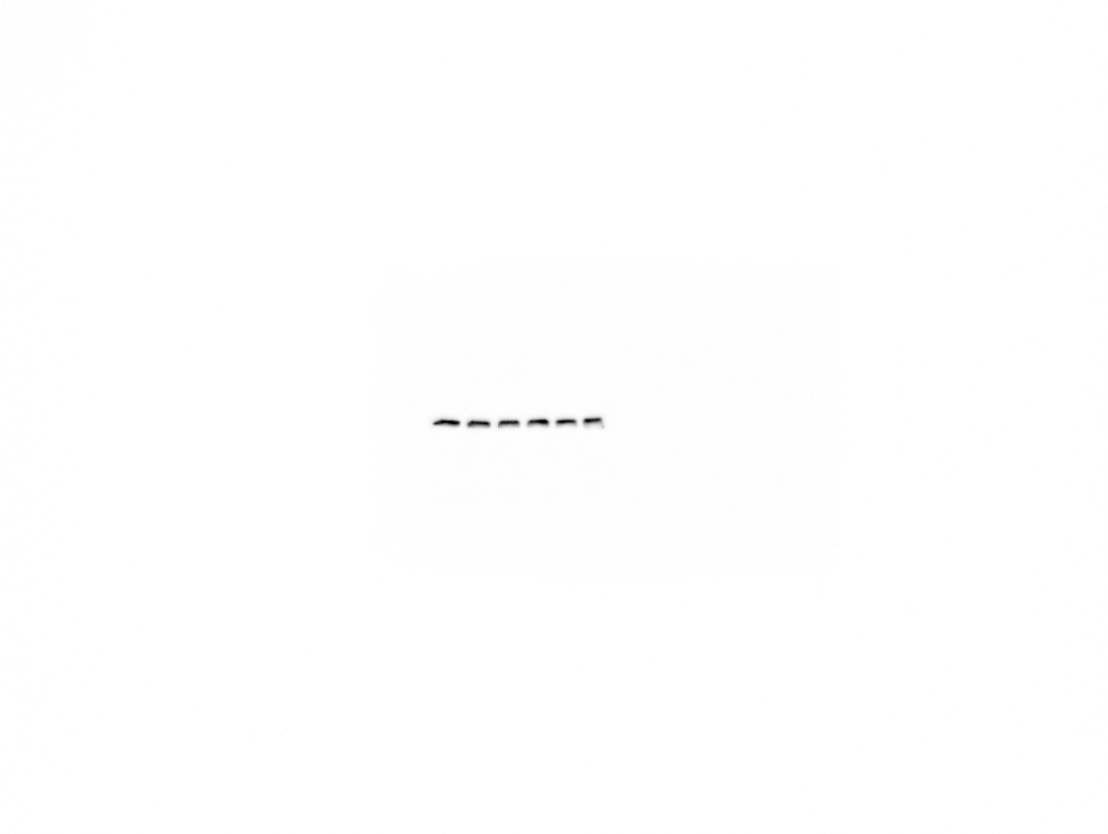

Supplement: Supplementary file 3 — Source data Fig. 2 [file 44318_2025_545_MOESM3_ESM.zip › Fig 2/2F/2F U2OS/24012024 U2OS BTZ TC Tubulin/2024-0124-113316.tif]

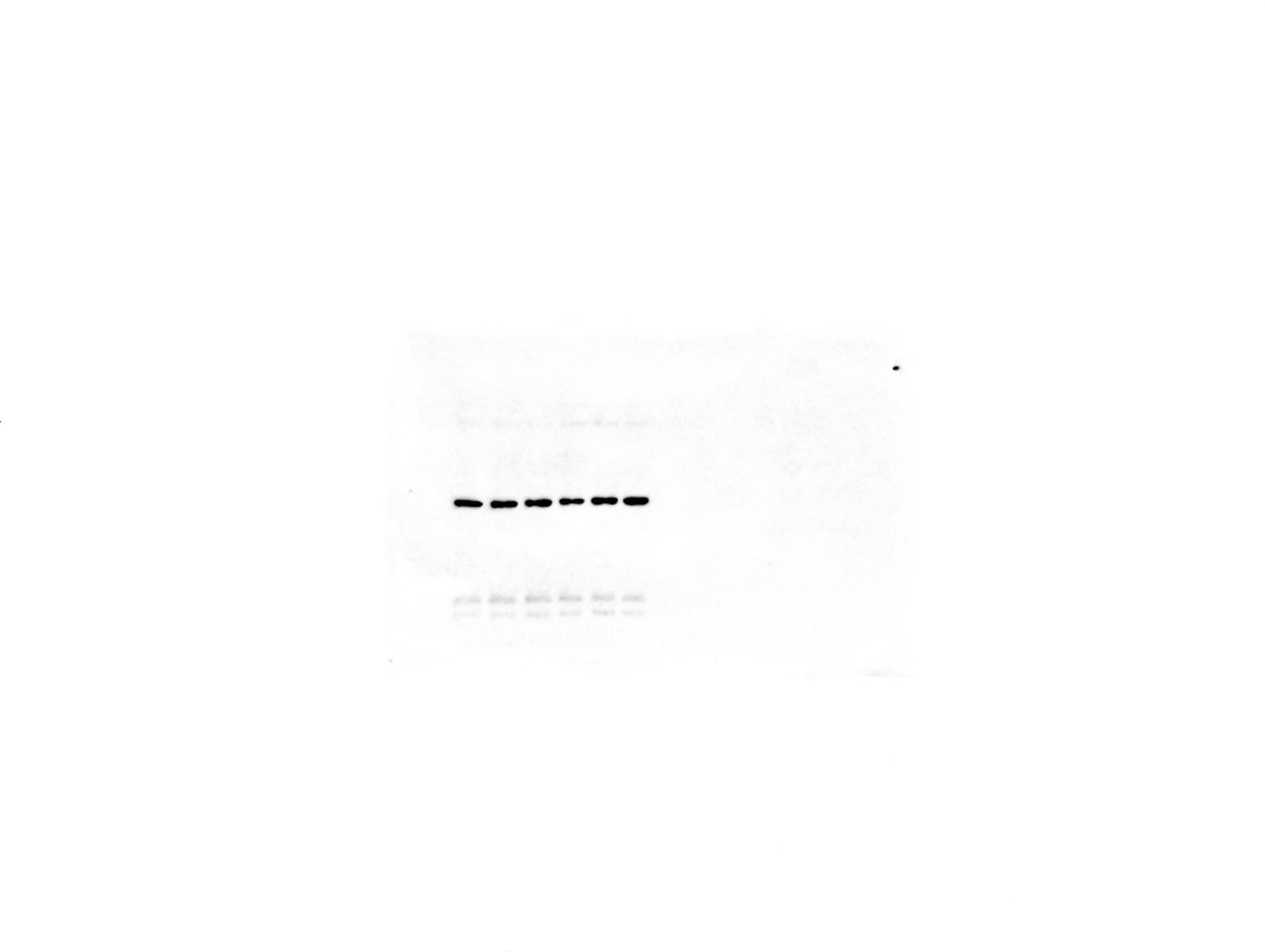

Supplement: Supplementary file 3 — Source data Fig. 2 [file 44318_2025_545_MOESM3_ESM.zip › Fig 2/2E/2E U2OS/22112023 U2OS Tubulin/2023-1122-145120_pub.tif]

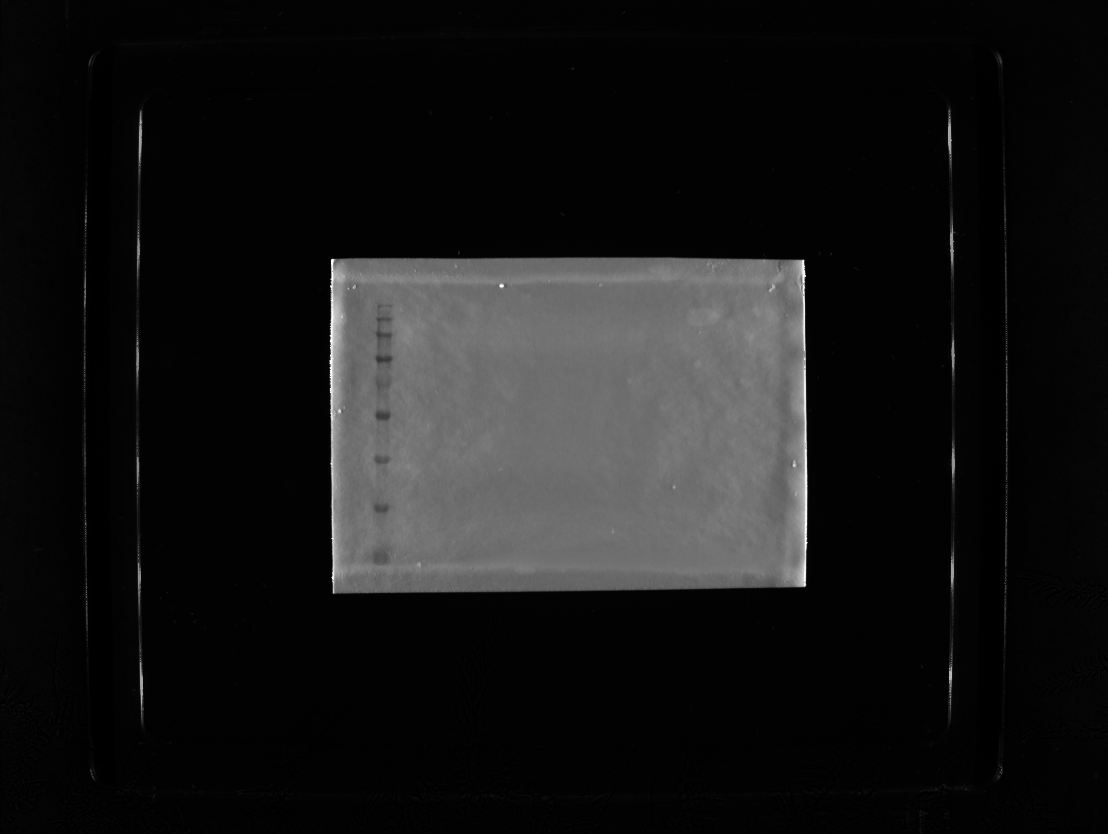

Supplement: Supplementary file 3 — Source data Fig. 2 [file 44318_2025_545_MOESM3_ESM.zip › Fig 2/2E/2E U2OS/22112023 U2OS Tubulin/2023-1122-145119.tif]

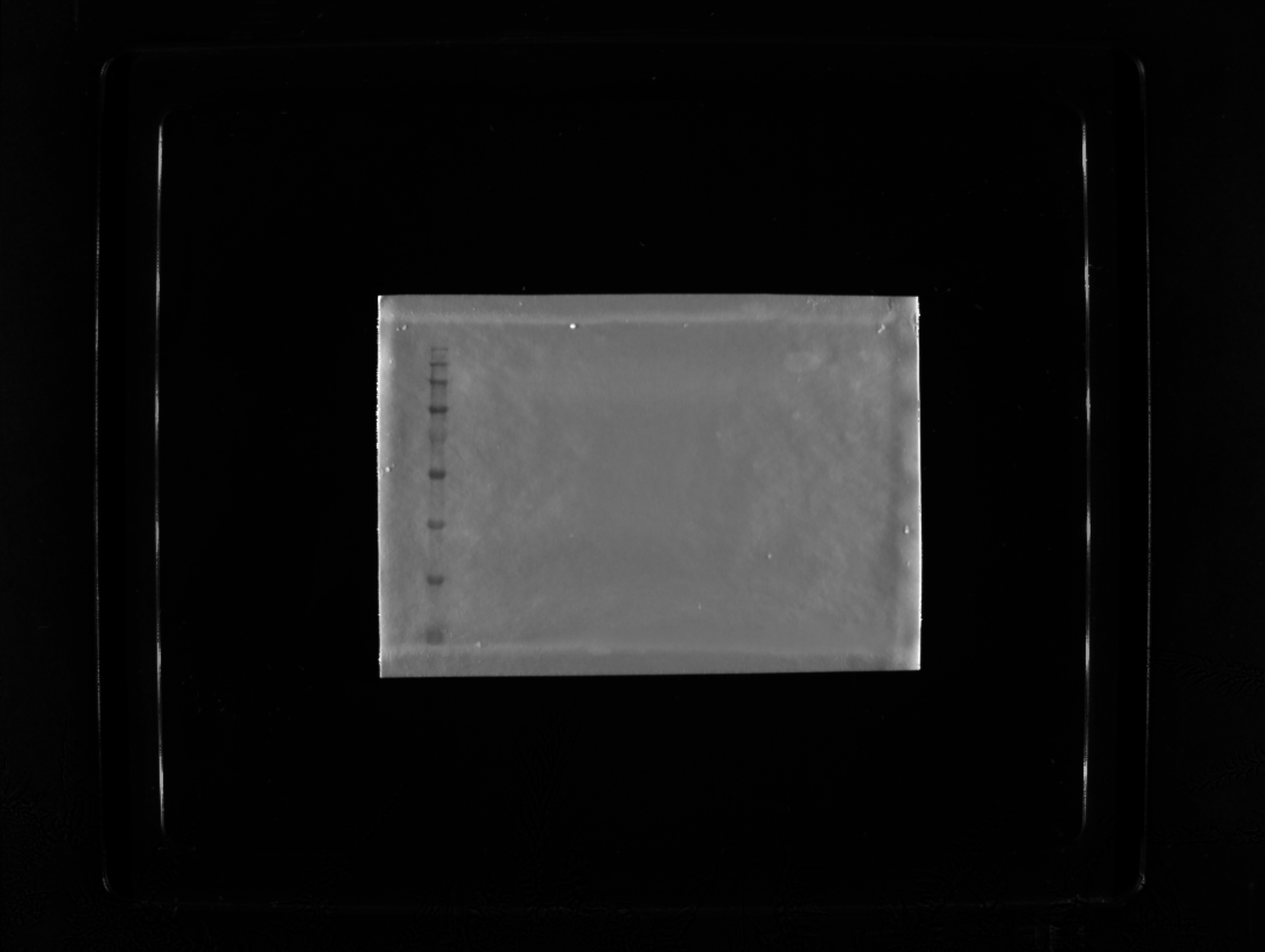

Supplement: Supplementary file 3 — Source data Fig. 2 [file 44318_2025_545_MOESM3_ESM.zip › Fig 2/2E/2E U2OS/22112023 U2OS Tubulin/2023-1122-145119_pub.tif]

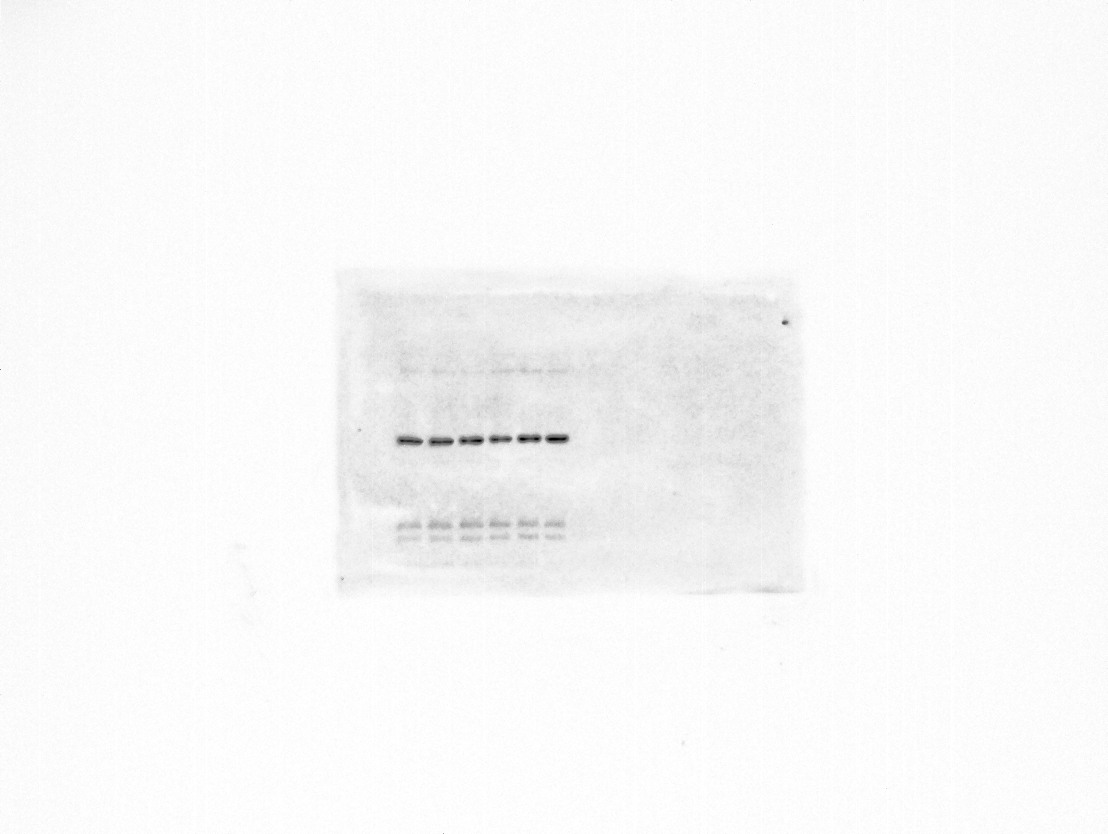

Supplement: Supplementary file 3 — Source data Fig. 2 [file 44318_2025_545_MOESM3_ESM.zip › Fig 2/2E/2E U2OS/22112023 U2OS Tubulin/2023-1122-145120.tif]

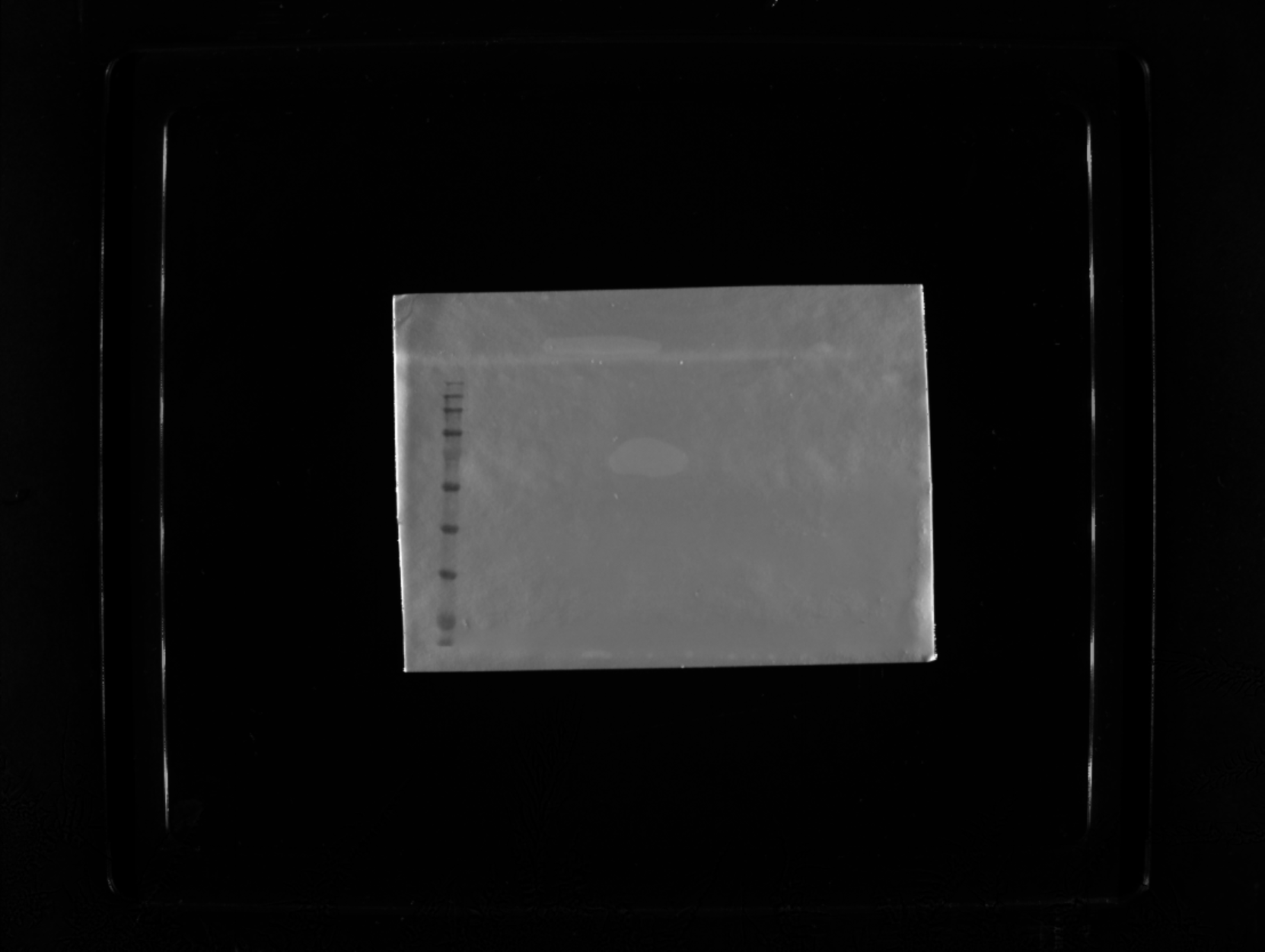

Supplement: Supplementary file 3 — Source data Fig. 2 [file 44318_2025_545_MOESM3_ESM.zip › Fig 2/2E/2E U2OS/22112023 U2OS CST/2023-1122-152249_pub.tif]

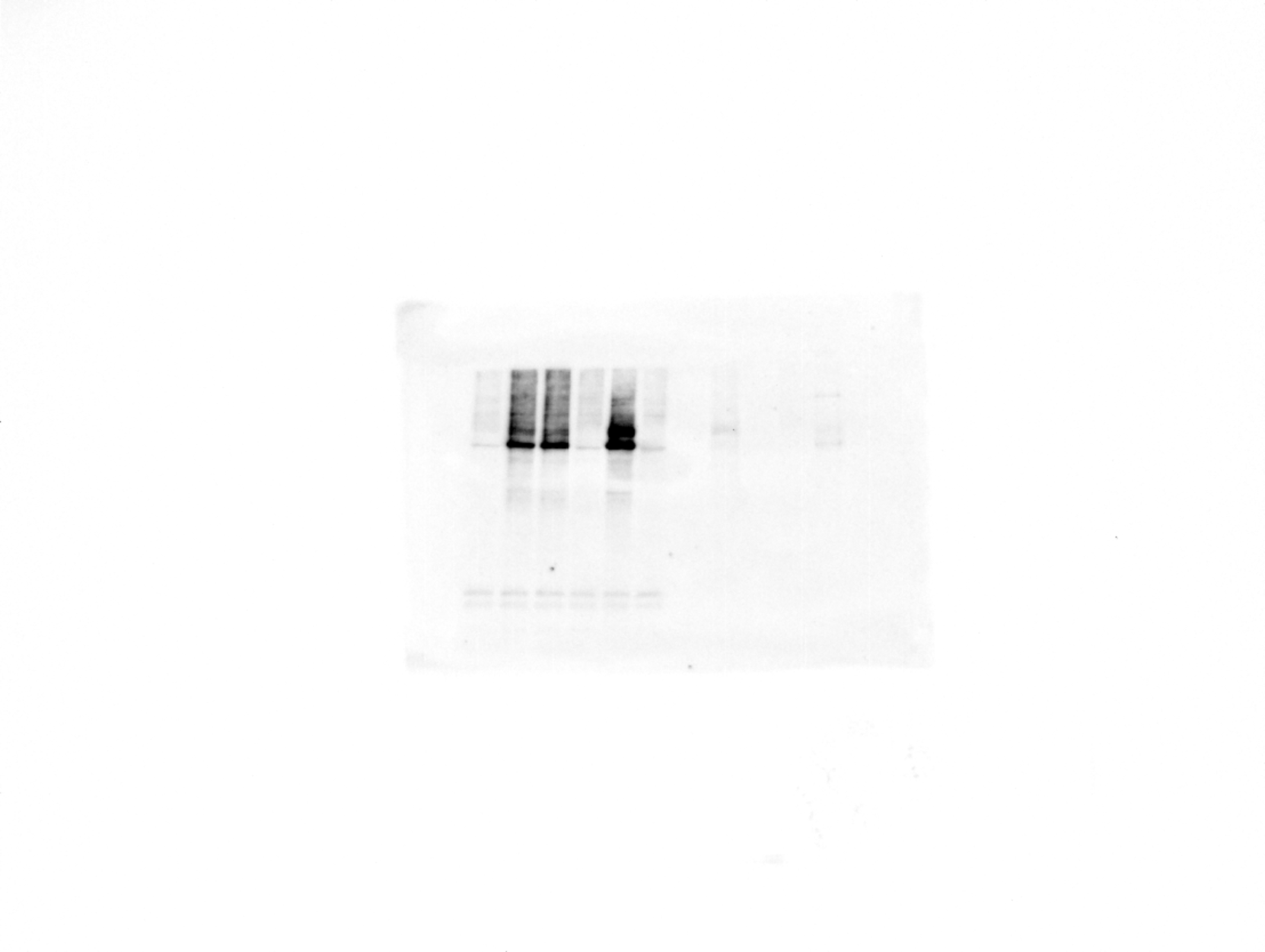

Supplement: Supplementary file 3 — Source data Fig. 2 [file 44318_2025_545_MOESM3_ESM.zip › Fig 2/2E/2E U2OS/22112023 U2OS CST/2023-1122-152250_pub.tif]

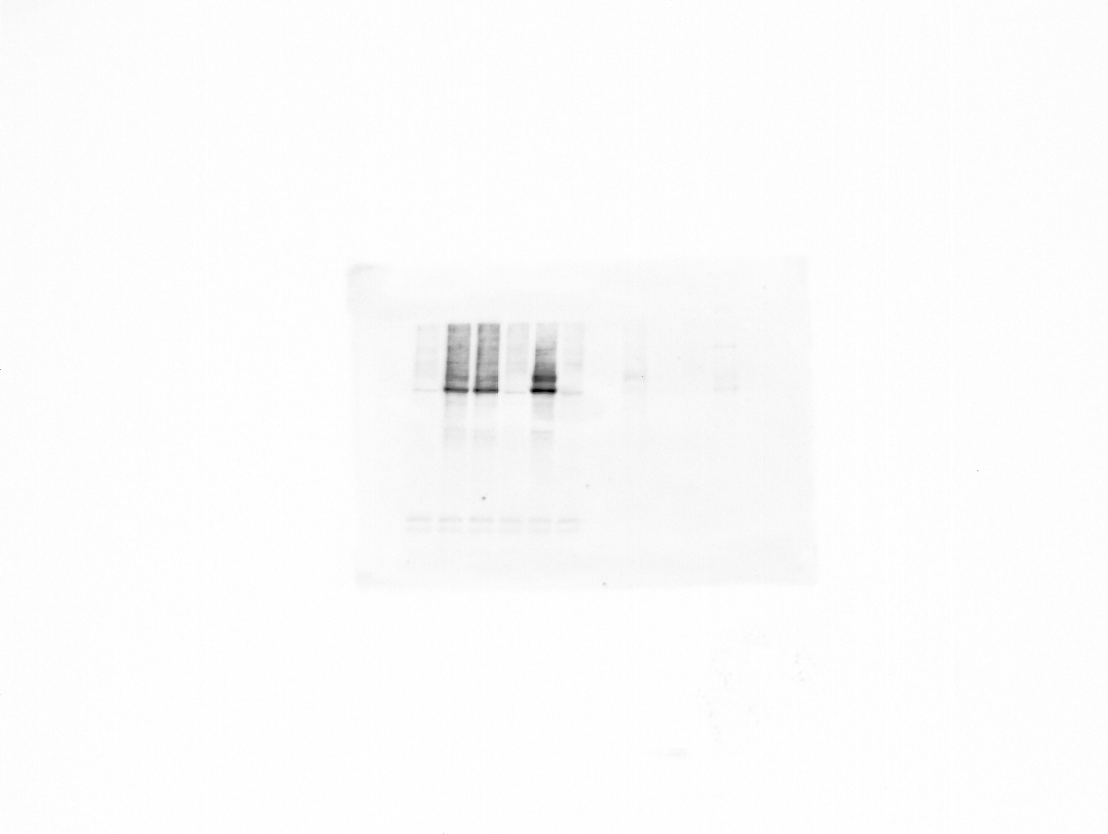

Supplement: Supplementary file 3 — Source data Fig. 2 [file 44318_2025_545_MOESM3_ESM.zip › Fig 2/2E/2E U2OS/22112023 U2OS CST/2023-1122-152250.tif]

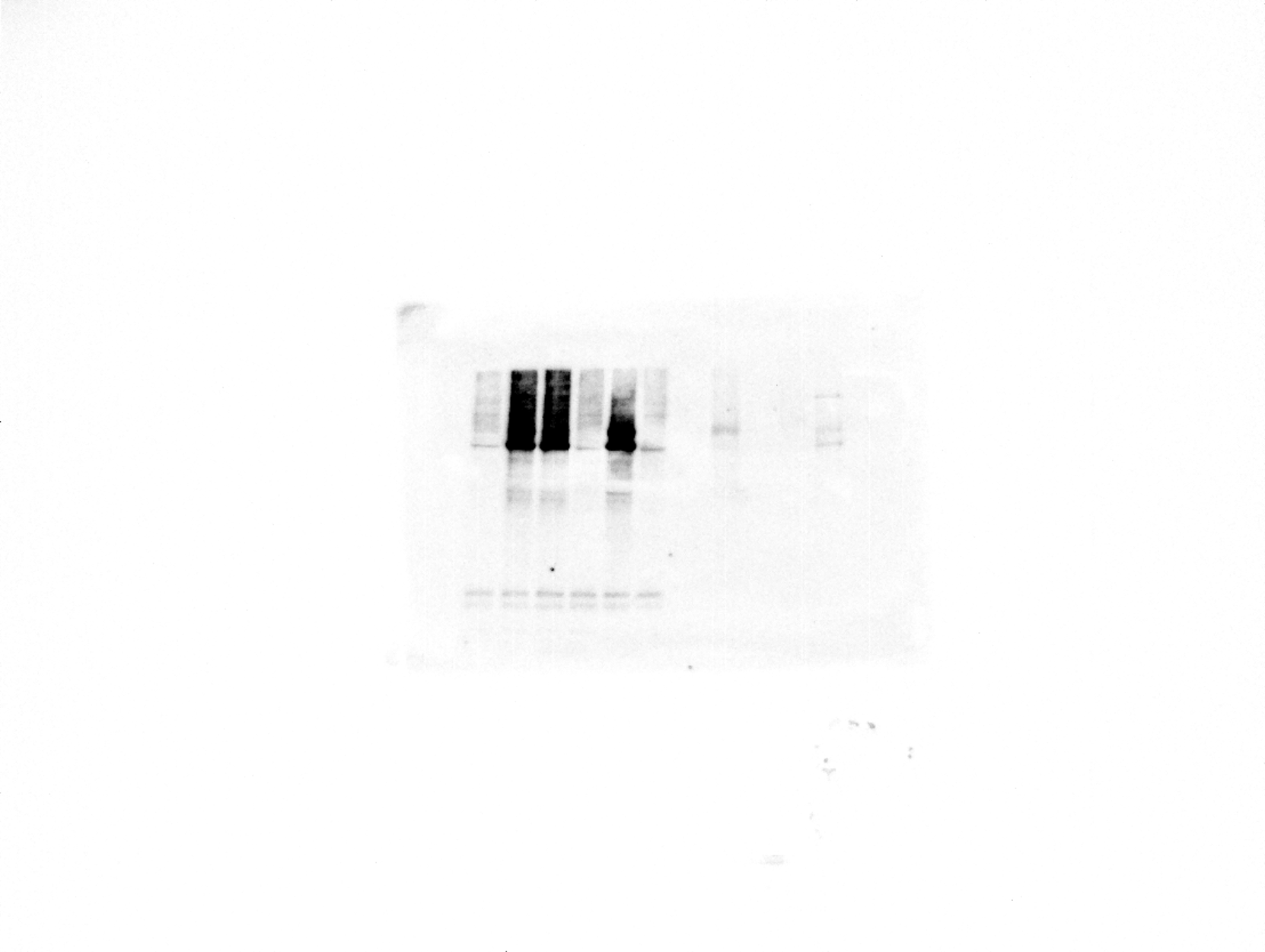

Supplement: Supplementary file 3 — Source data Fig. 2 [file 44318_2025_545_MOESM3_ESM.zip › Fig 2/2E/2E U2OS/22112023 U2OS CST/S4F1-1122-152252_pub.tif]

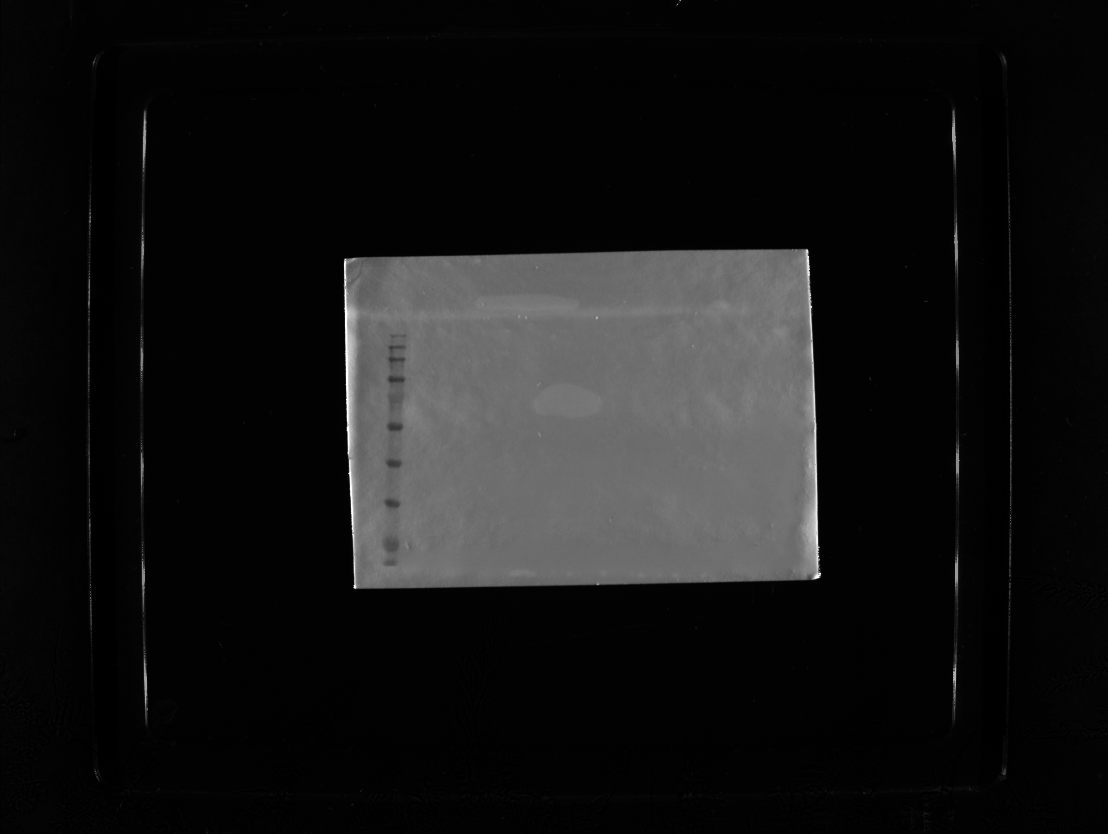

Supplement: Supplementary file 3 — Source data Fig. 2 [file 44318_2025_545_MOESM3_ESM.zip › Fig 2/2E/2E U2OS/22112023 U2OS CST/2023-1122-152249.tif]

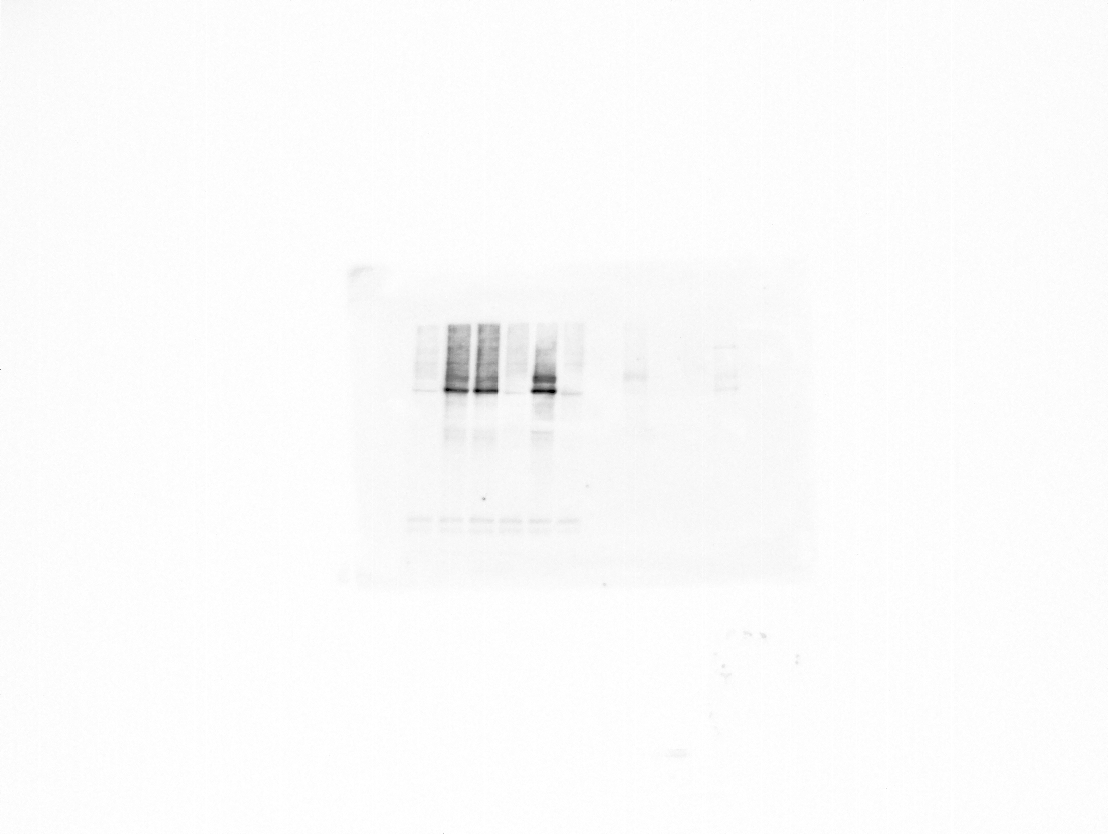

Supplement: Supplementary file 3 — Source data Fig. 2 [file 44318_2025_545_MOESM3_ESM.zip › Fig 2/2E/2E U2OS/22112023 U2OS CST/S4F1-1122-152252.tif]

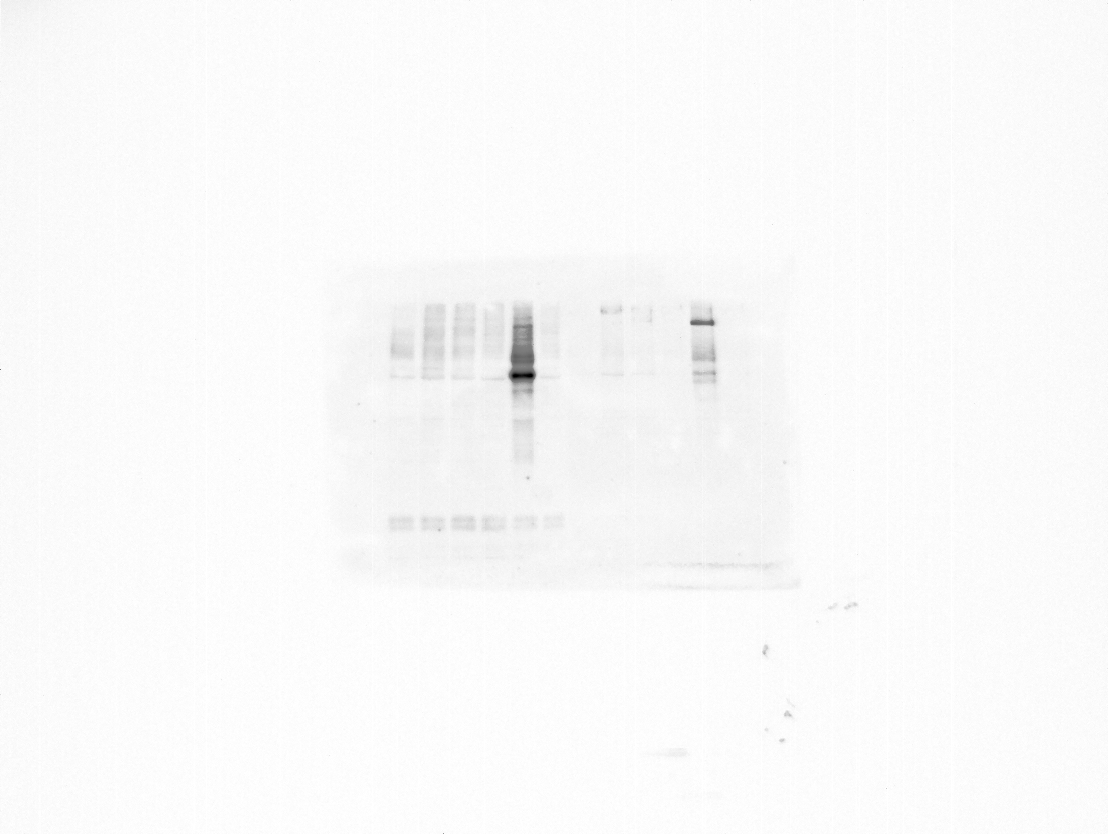

Supplement: Supplementary file 3 — Source data Fig. 2 [file 44318_2025_545_MOESM3_ESM.zip › Fig 2/2E/2E HeLa/22112023 HeLa CST/S3F2-1122-151712.tif]

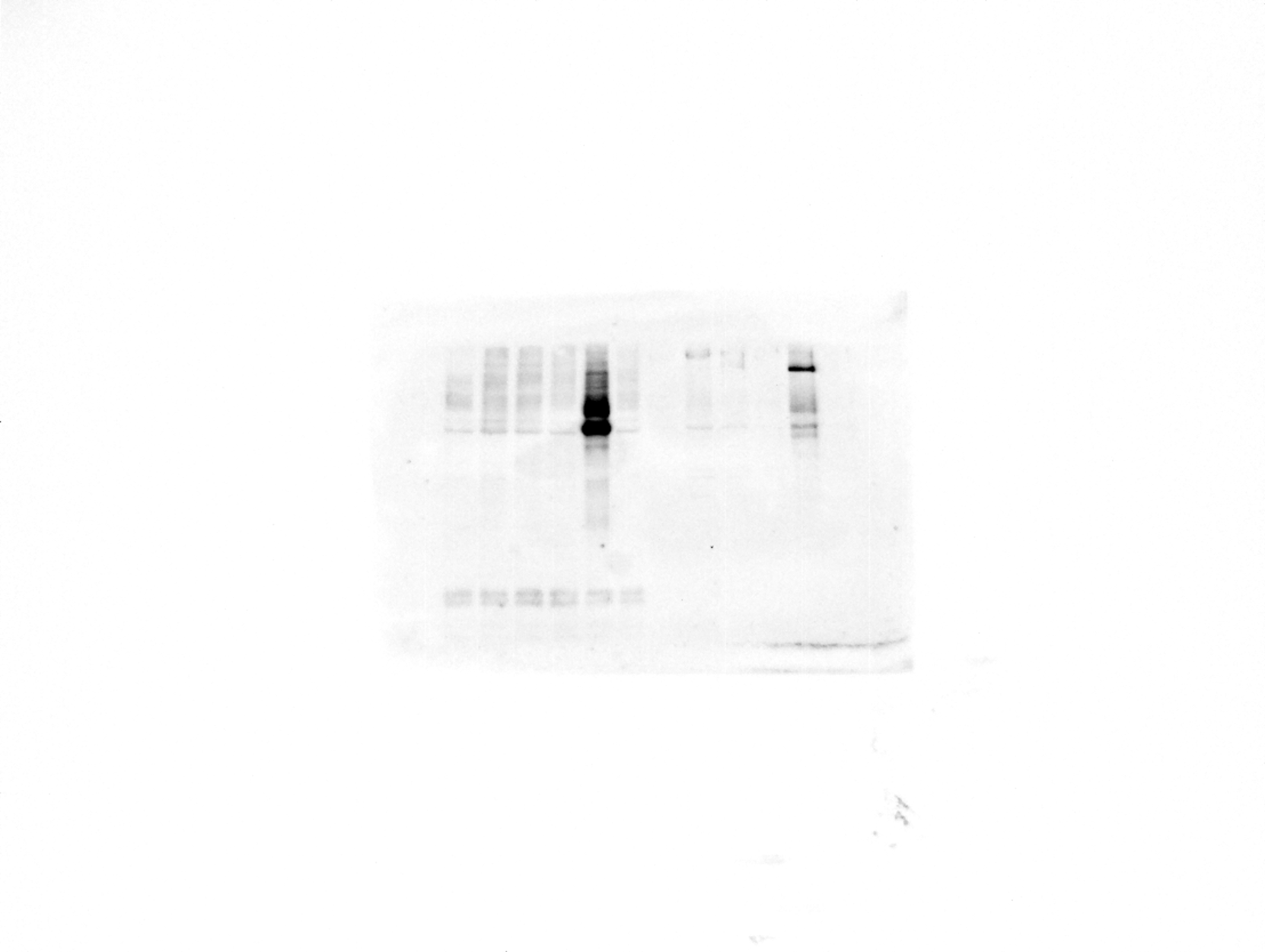

Supplement: Supplementary file 3 — Source data Fig. 2 [file 44318_2025_545_MOESM3_ESM.zip › Fig 2/2E/2E HeLa/22112023 HeLa CST/2023-1122-151709_pub.tif]

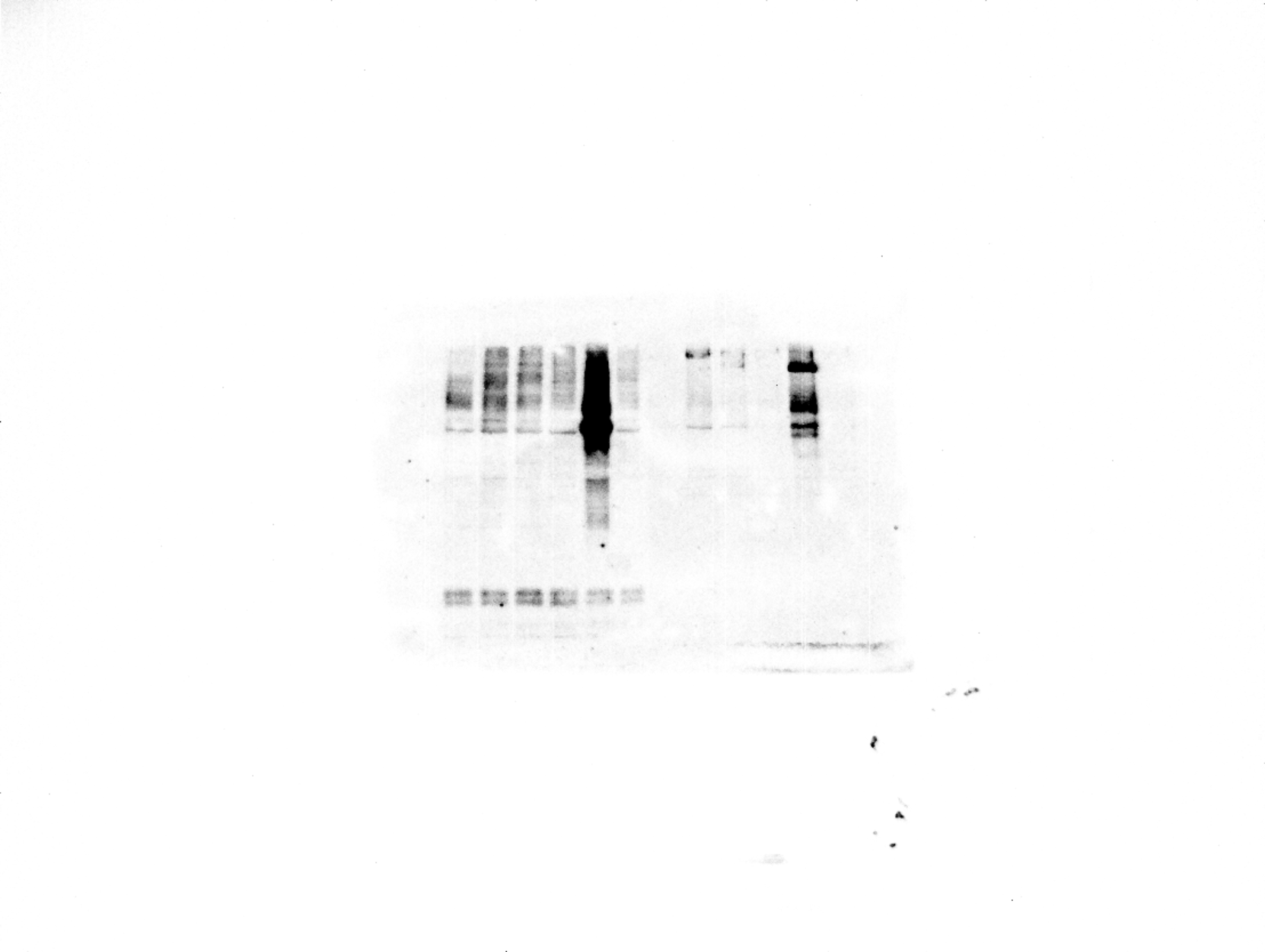

Supplement: Supplementary file 3 — Source data Fig. 2 [file 44318_2025_545_MOESM3_ESM.zip › Fig 2/2E/2E HeLa/22112023 HeLa CST/S3F2-1122-151712_pub.tif]

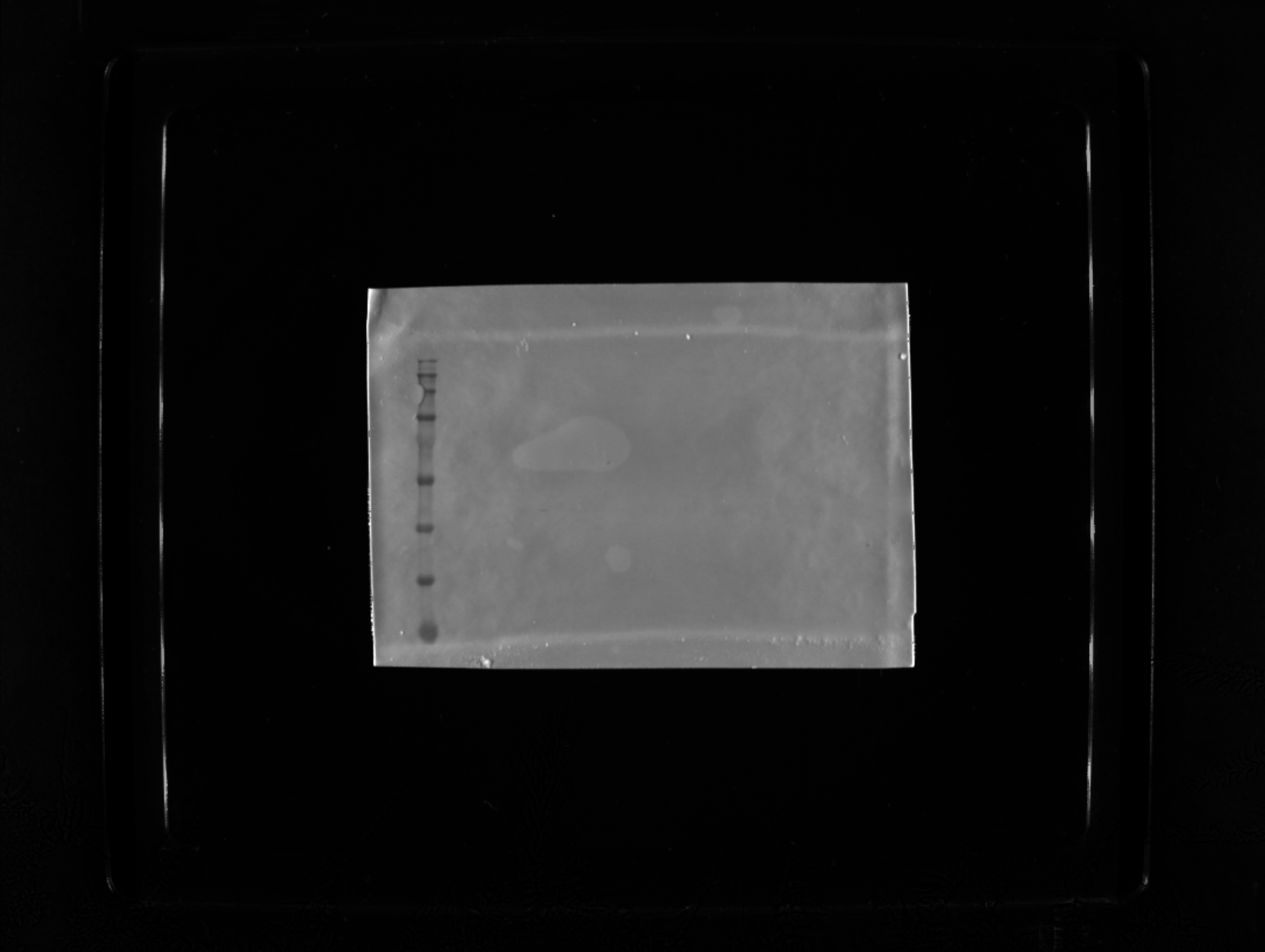

Supplement: Supplementary file 3 — Source data Fig. 2 [file 44318_2025_545_MOESM3_ESM.zip › Fig 2/2E/2E HeLa/22112023 HeLa CST/2023-1122-151708_pub.tif]

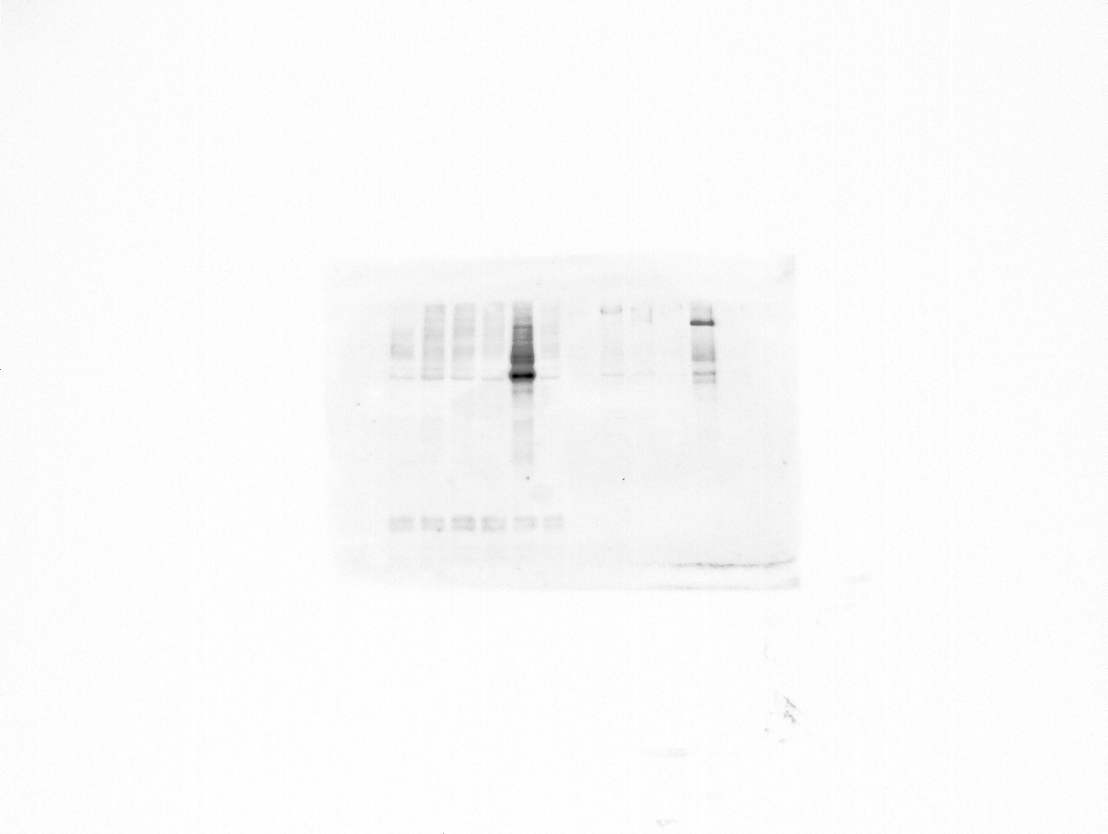

Supplement: Supplementary file 3 — Source data Fig. 2 [file 44318_2025_545_MOESM3_ESM.zip › Fig 2/2E/2E HeLa/22112023 HeLa CST/2023-1122-151709.tif]

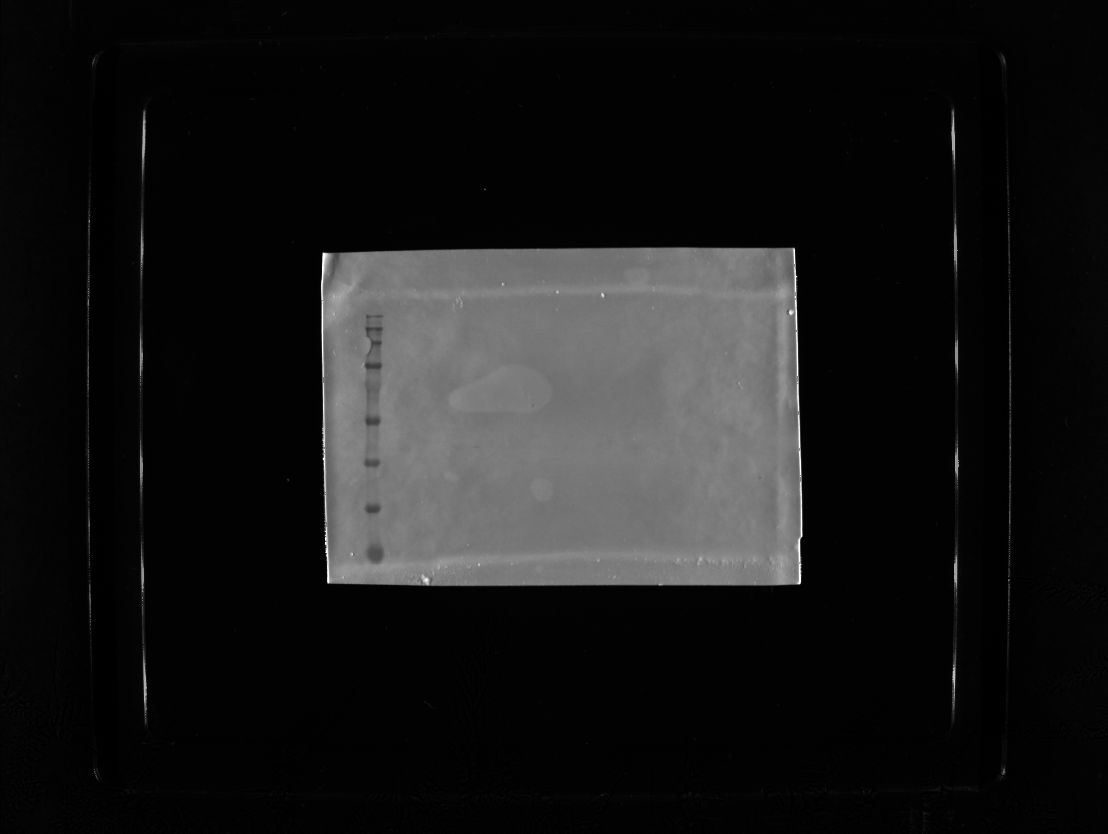

Supplement: Supplementary file 3 — Source data Fig. 2 [file 44318_2025_545_MOESM3_ESM.zip › Fig 2/2E/2E HeLa/22112023 HeLa CST/2023-1122-151708.tif]

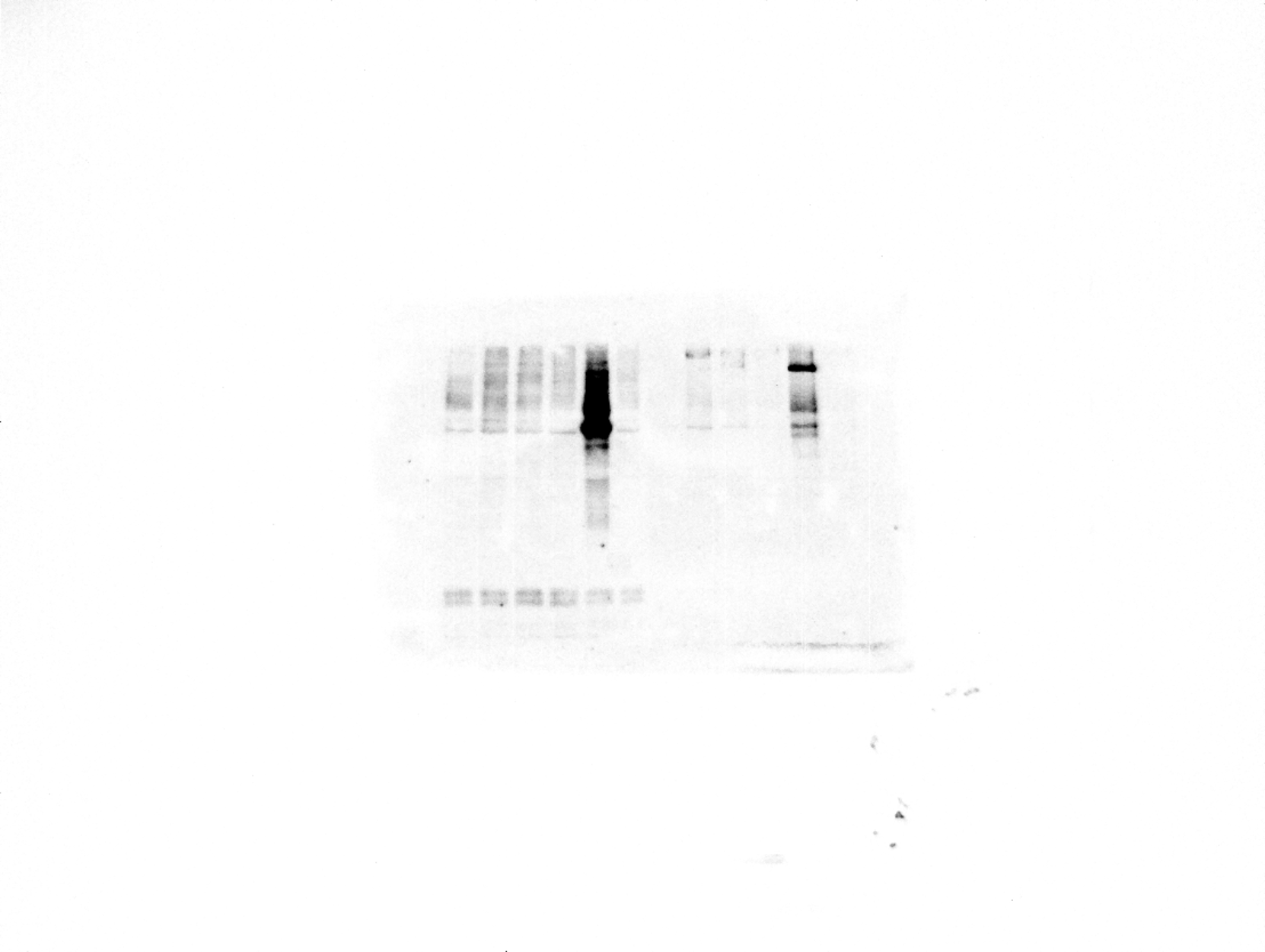

Supplement: Supplementary file 3 — Source data Fig. 2 [file 44318_2025_545_MOESM3_ESM.zip › Fig 2/2E/2E HeLa/22112023 HeLa CST/S3F1-1122-151711_pub.tif]

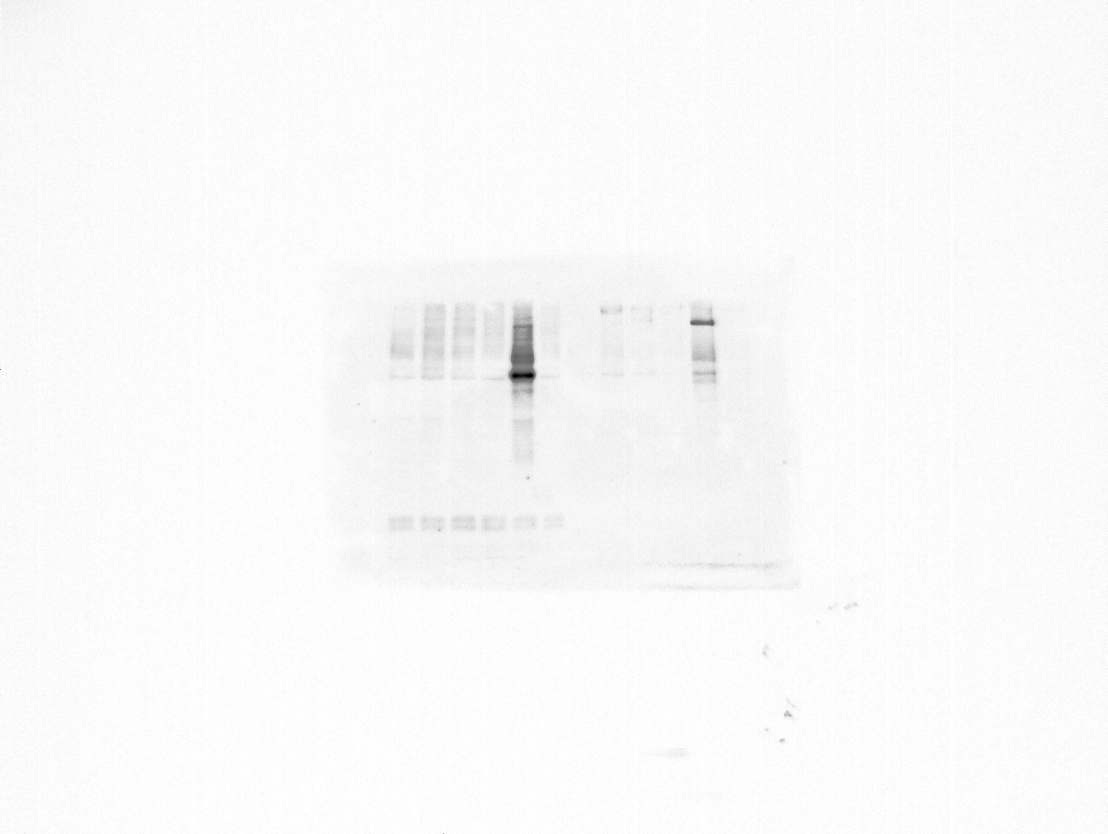

Supplement: Supplementary file 3 — Source data Fig. 2 [file 44318_2025_545_MOESM3_ESM.zip › Fig 2/2E/2E HeLa/22112023 HeLa CST/S3F1-1122-151711.tif]

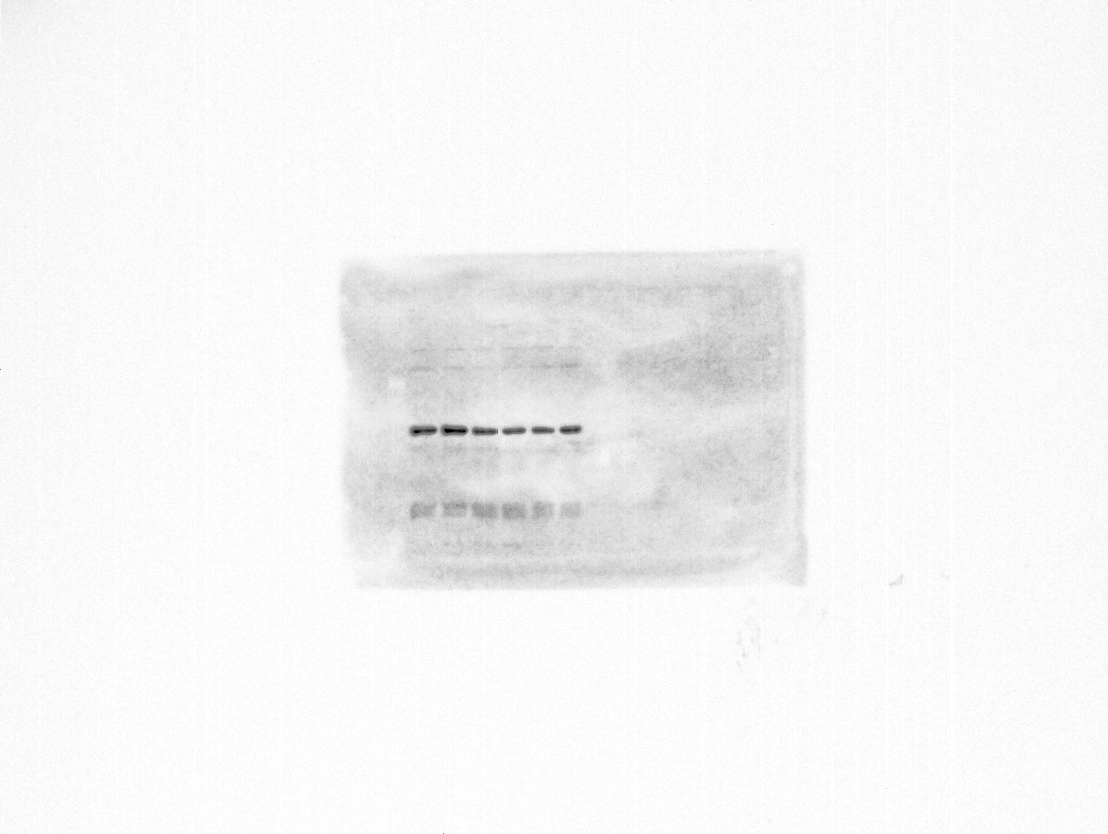

Supplement: Supplementary file 3 — Source data Fig. 2 [file 44318_2025_545_MOESM3_ESM.zip › Fig 2/2E/2E HeLa/22112023 HeLa Tubulin/2023-1122-144733.tif]

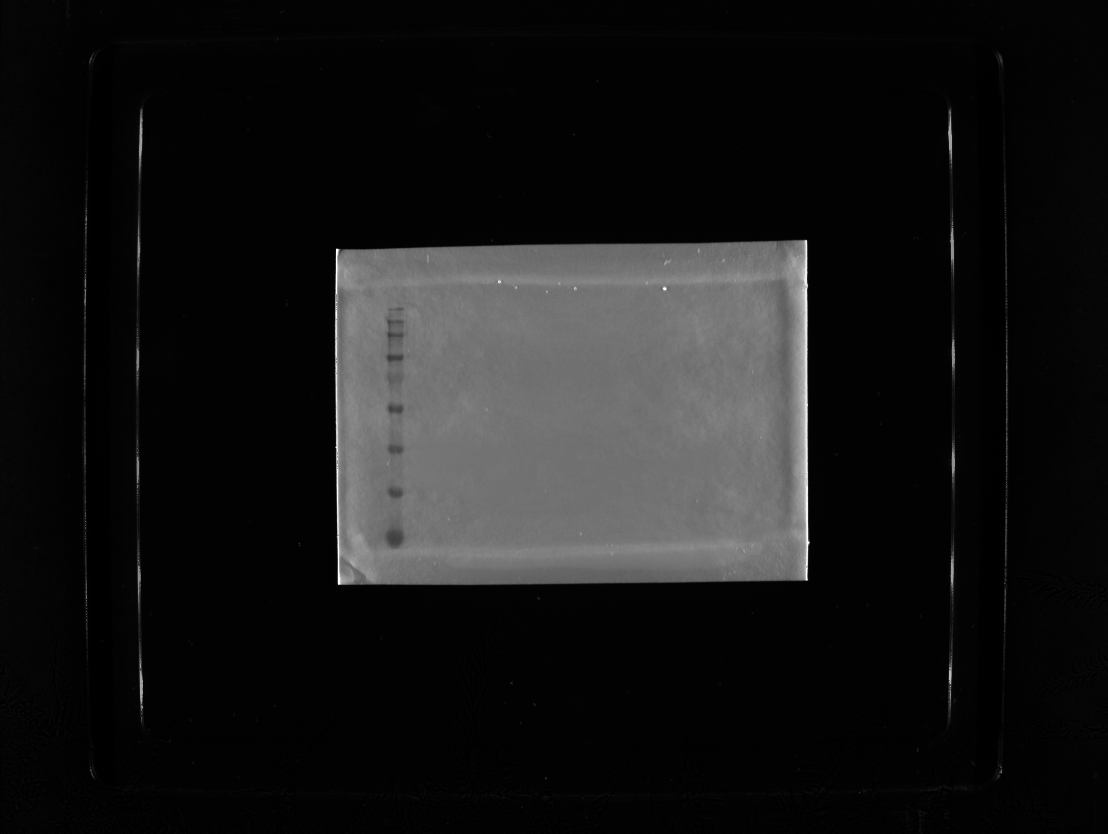

Supplement: Supplementary file 3 — Source data Fig. 2 [file 44318_2025_545_MOESM3_ESM.zip › Fig 2/2E/2E HeLa/22112023 HeLa Tubulin/2023-1122-144732.tif]

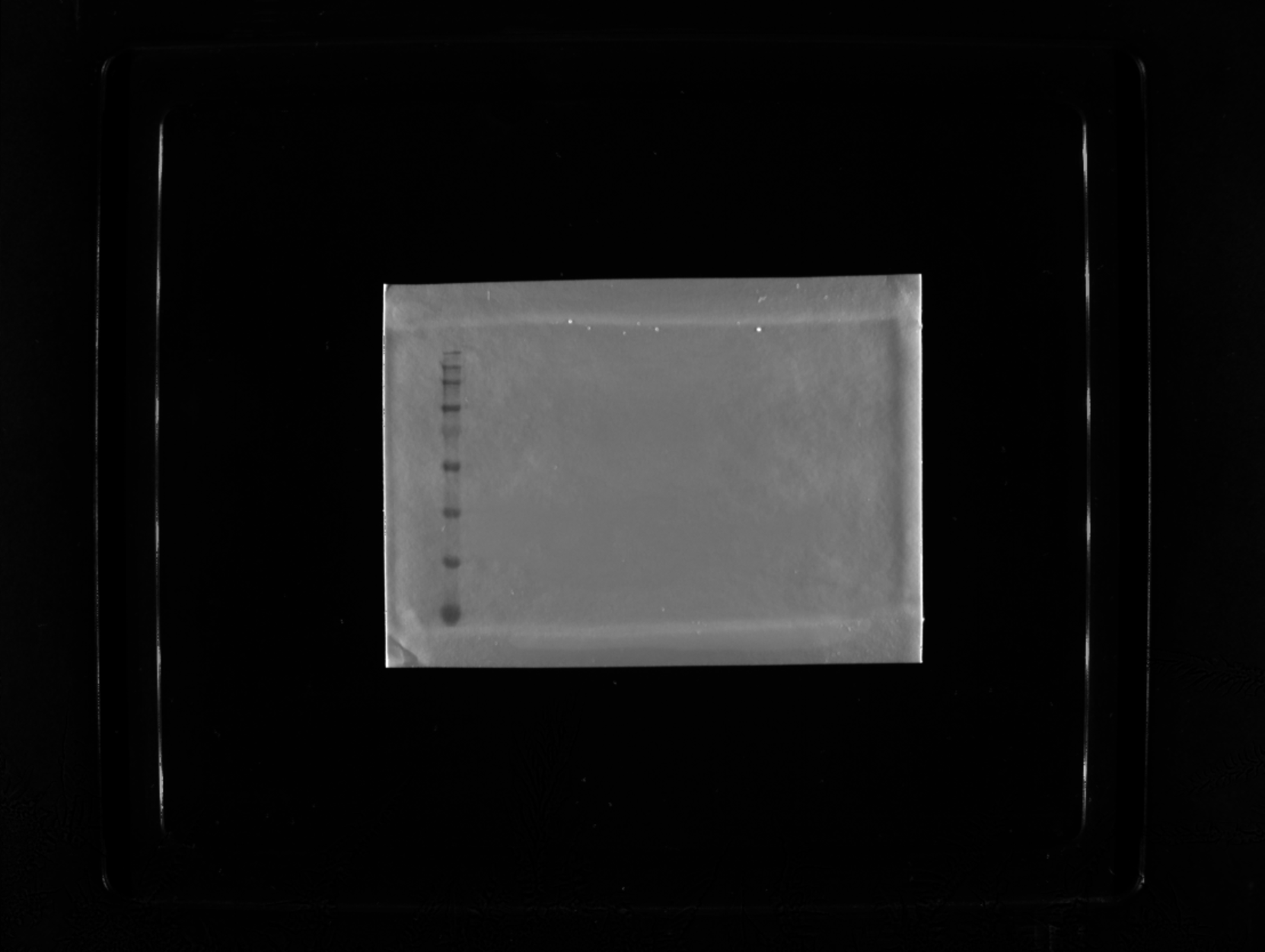

Supplement: Supplementary file 3 — Source data Fig. 2 [file 44318_2025_545_MOESM3_ESM.zip › Fig 2/2E/2E HeLa/22112023 HeLa Tubulin/2023-1122-144732_pub.tif]

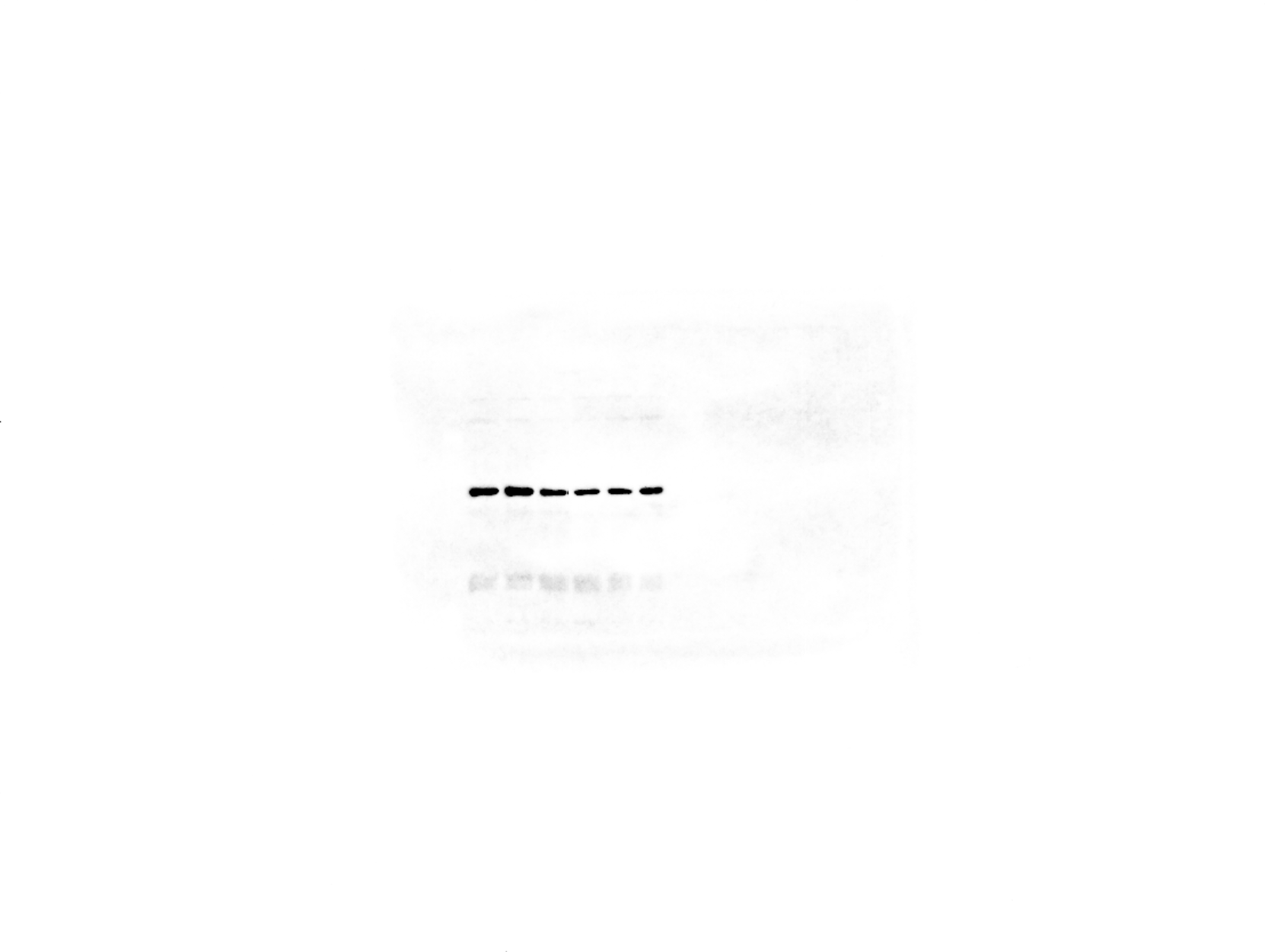

Supplement: Supplementary file 3 — Source data Fig. 2 [file 44318_2025_545_MOESM3_ESM.zip › Fig 2/2E/2E HeLa/22112023 HeLa Tubulin/2023-1122-144733_pub.tif]

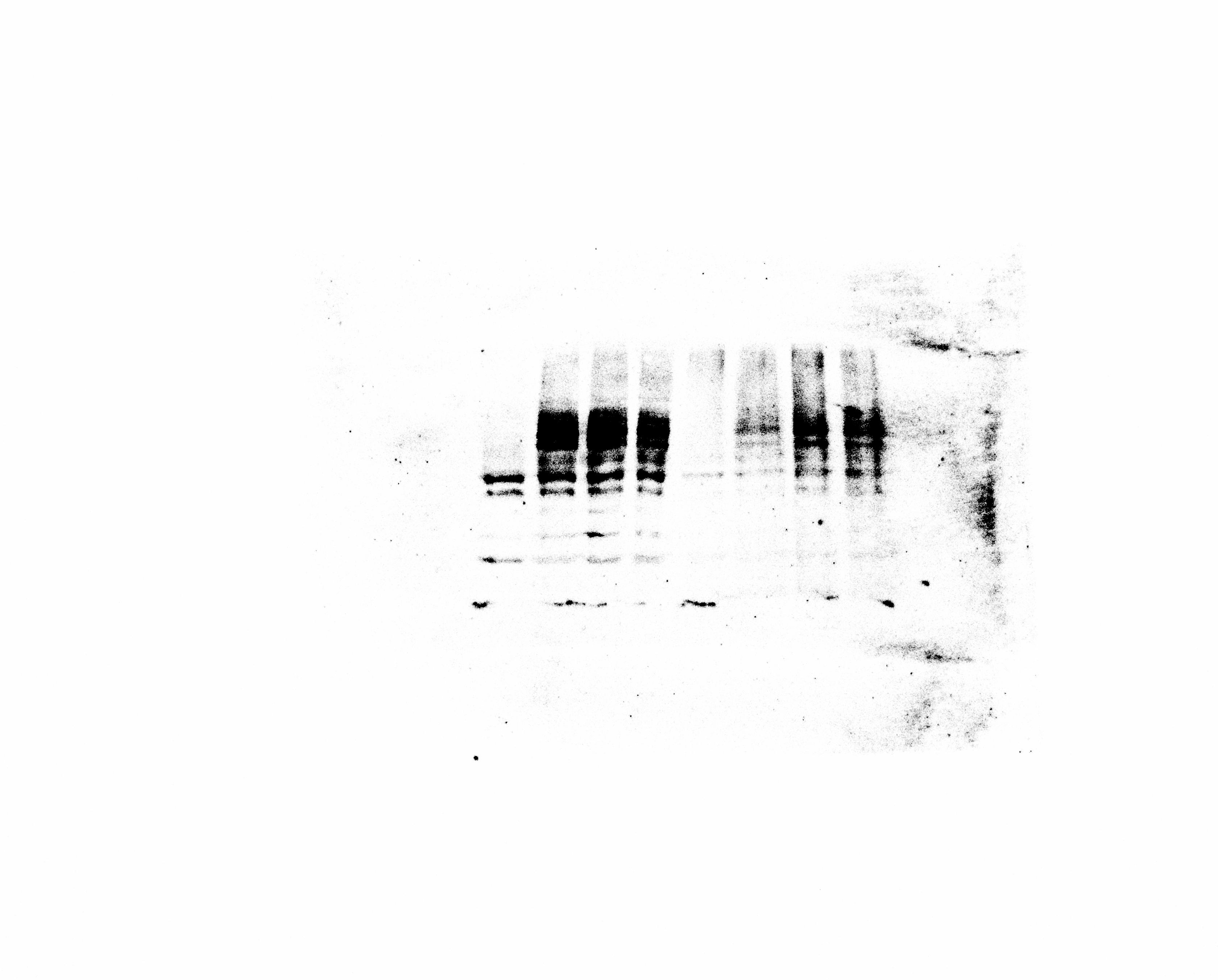

Supplement: Supplementary file 4 — Source data Fig. 3 [file 44318_2025_545_MOESM4_ESM.zip › Fig 3/3E/MAR insoluble/25.04.15_12.56.33_S4_F01_PUB_300.tif]

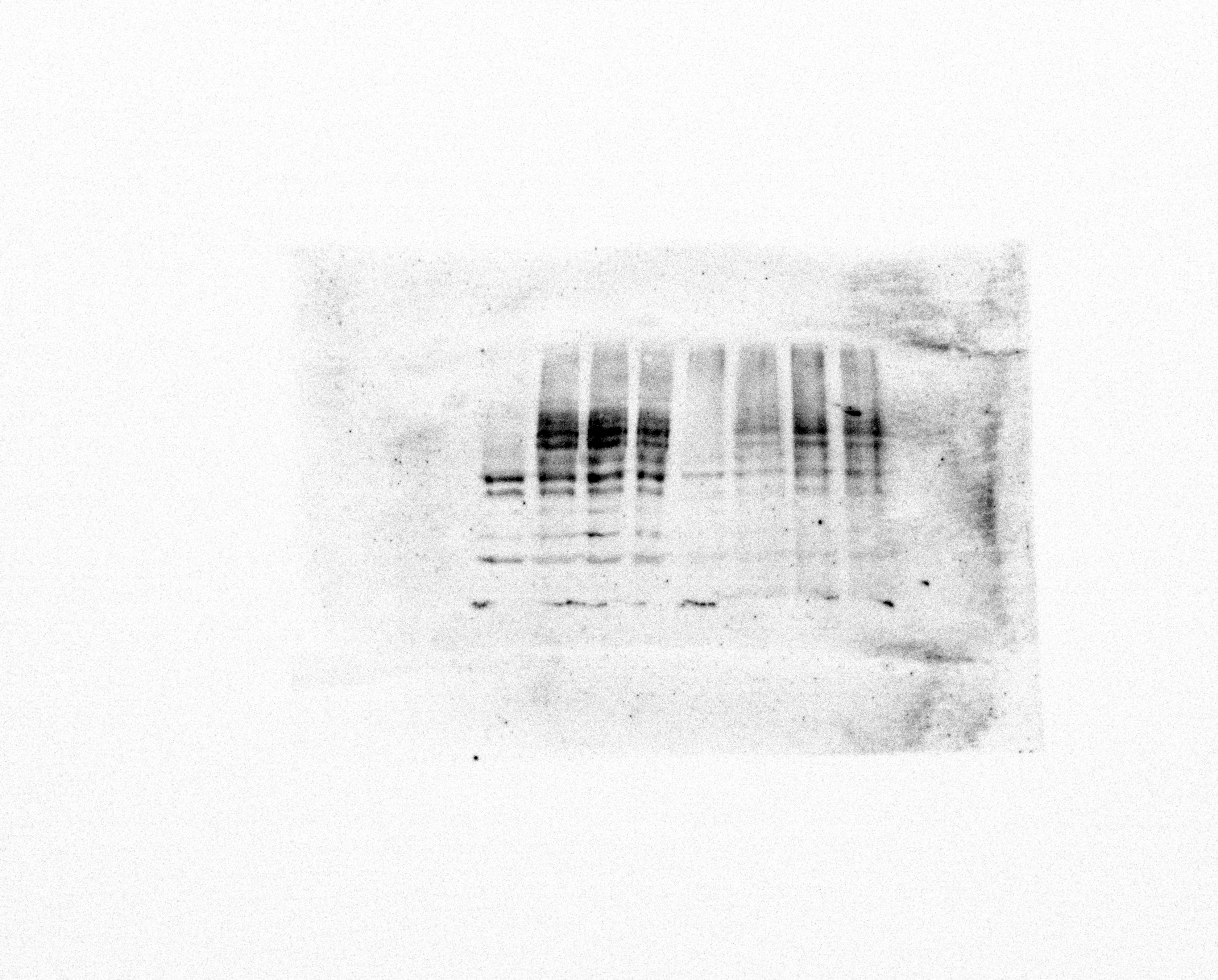

Supplement: Supplementary file 4 — Source data Fig. 3 [file 44318_2025_545_MOESM4_ESM.zip › Fig 3/3E/MAR insoluble/25.04.15_12.56.33_S4_F01.tif]

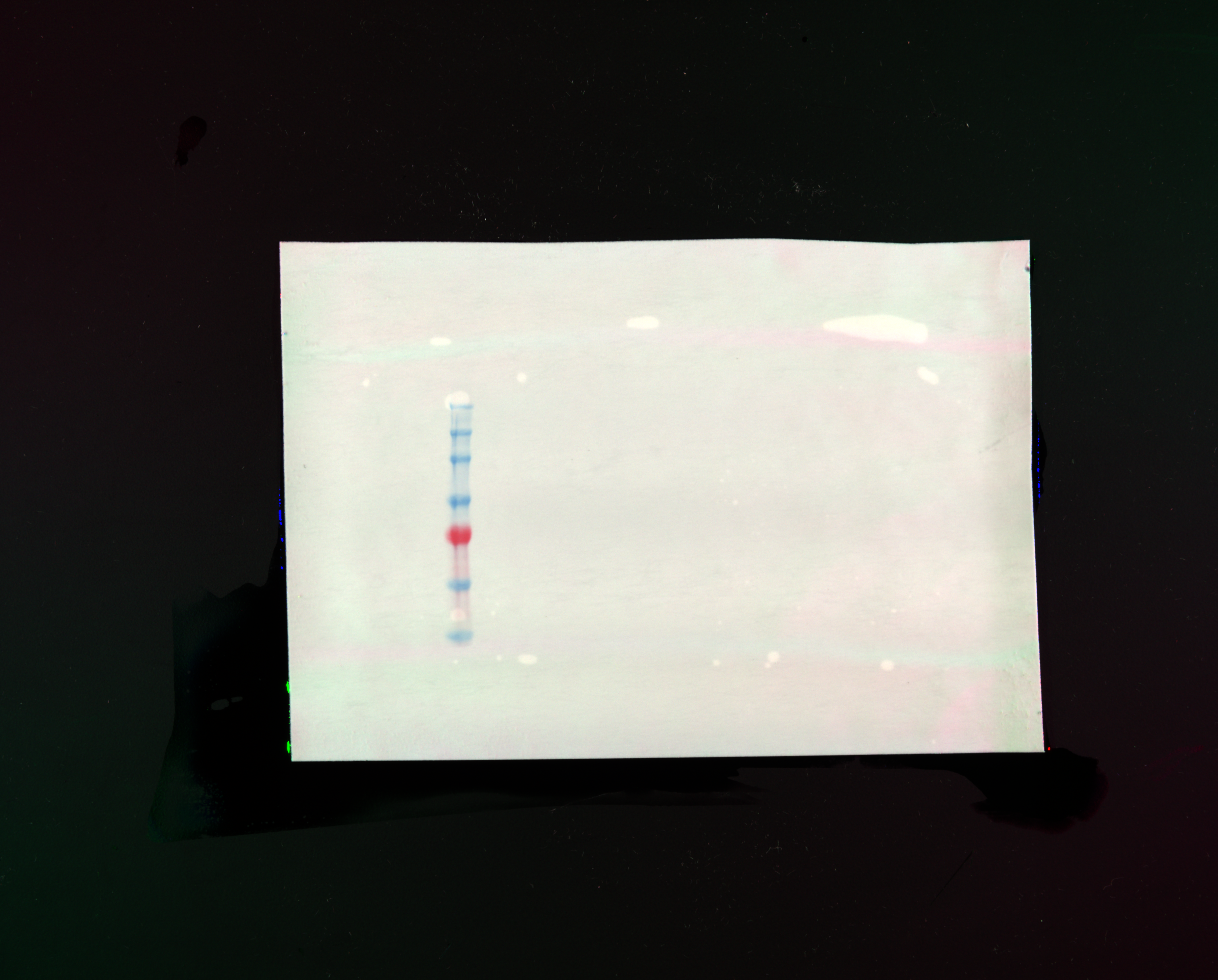

Supplement: Supplementary file 4 — Source data Fig. 3 [file 44318_2025_545_MOESM4_ESM.zip › Fig 3/3E/MAR insoluble/25.04.15_12.56.33_S4_marker.tif]

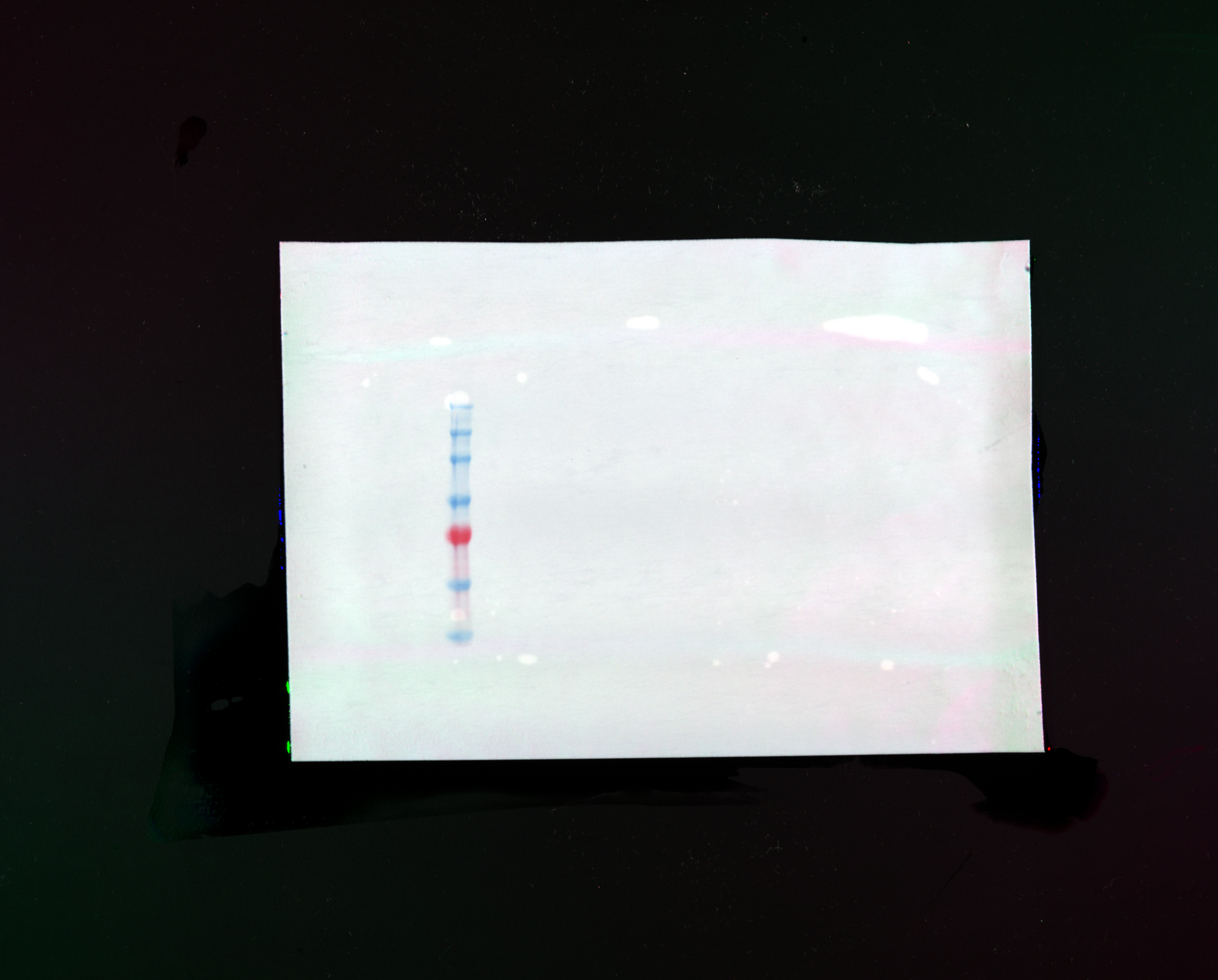

Supplement: Supplementary file 4 — Source data Fig. 3 [file 44318_2025_545_MOESM4_ESM.zip › Fig 3/3E/MAR insoluble/25.04.15_12.56.33_S4_marker_PUB_300.tif]

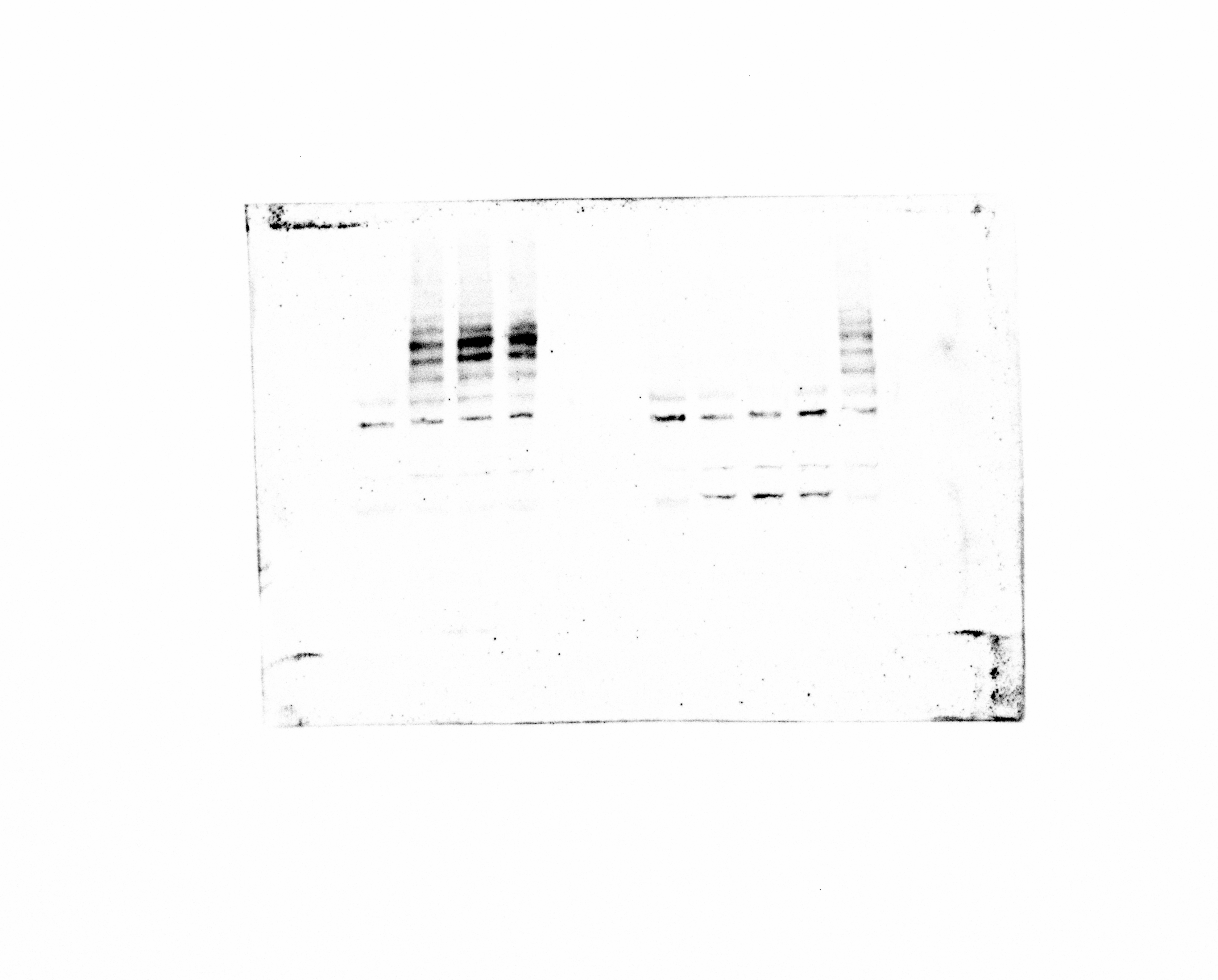

Supplement: Supplementary file 4 — Source data Fig. 3 [file 44318_2025_545_MOESM4_ESM.zip › Fig 3/3E/ MAR soluble/25.04.29_15.16.07_S2_F02_PUB_300.tif]

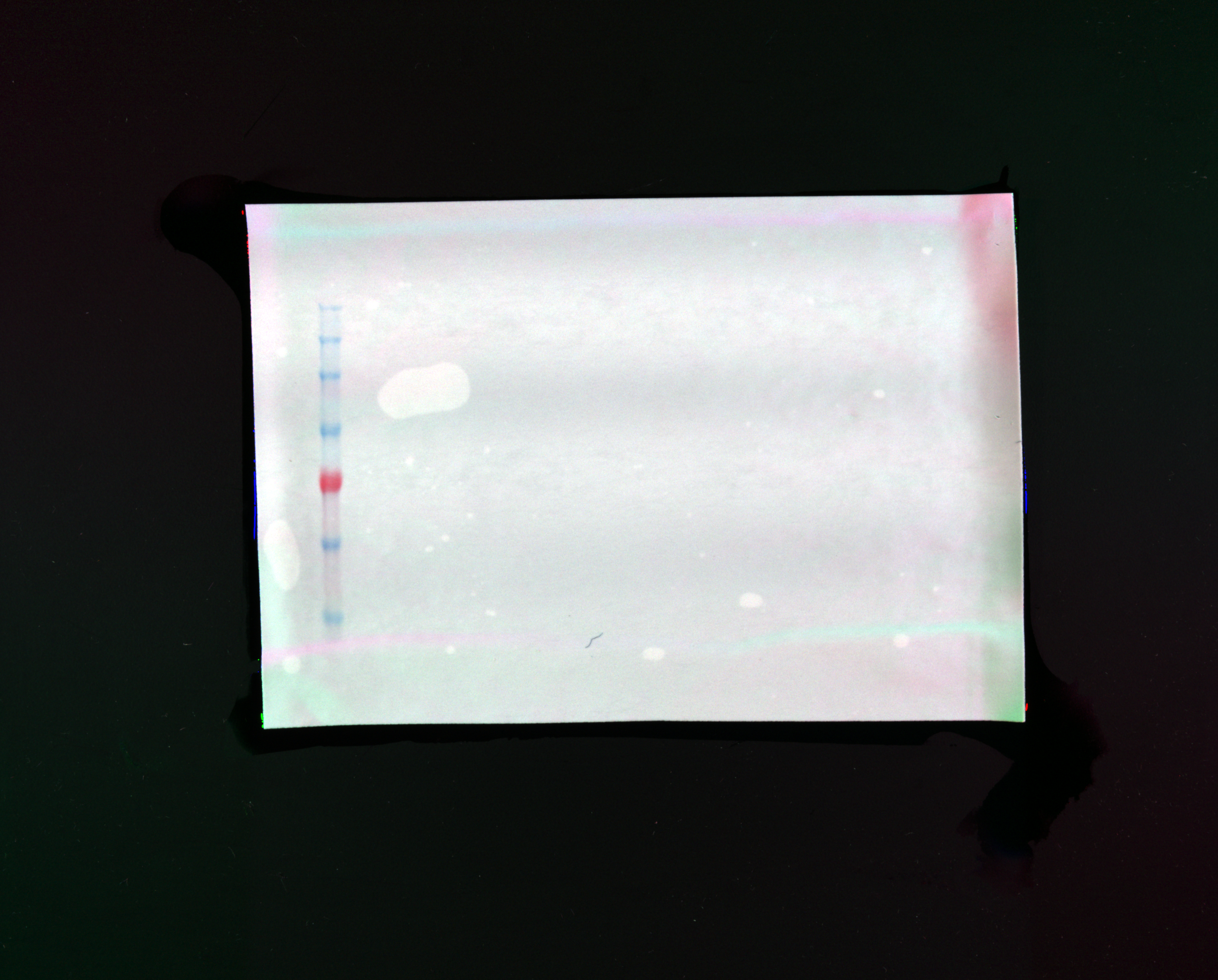

Supplement: Supplementary file 4 — Source data Fig. 3 [file 44318_2025_545_MOESM4_ESM.zip › Fig 3/3E/ MAR soluble/25.04.29_15.16.07_S2_marker_PUB_300.tif]

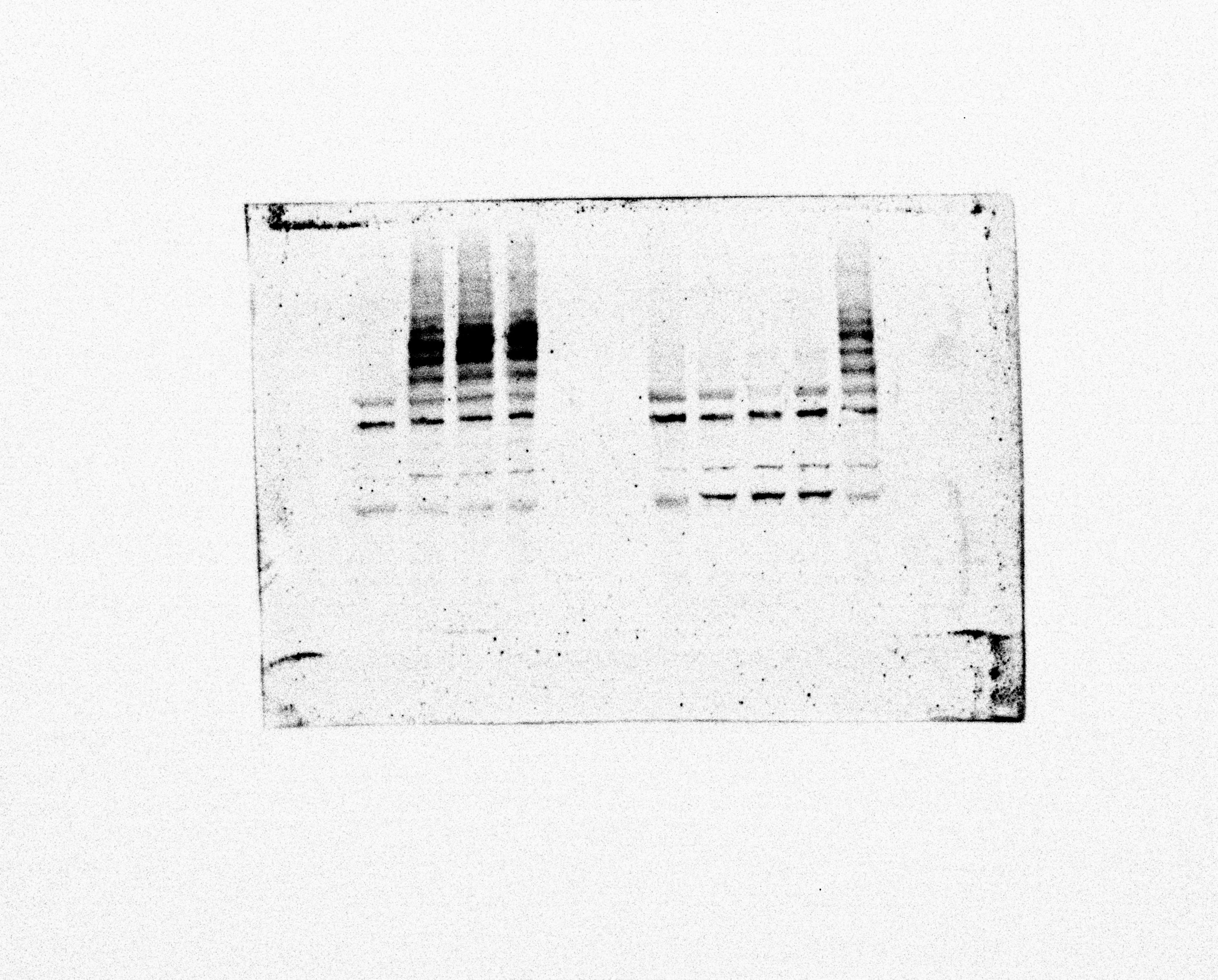

Supplement: Supplementary file 4 — Source data Fig. 3 [file 44318_2025_545_MOESM4_ESM.zip › Fig 3/3E/ MAR soluble/25.04.29_15.21.41_PUB_300.tif]

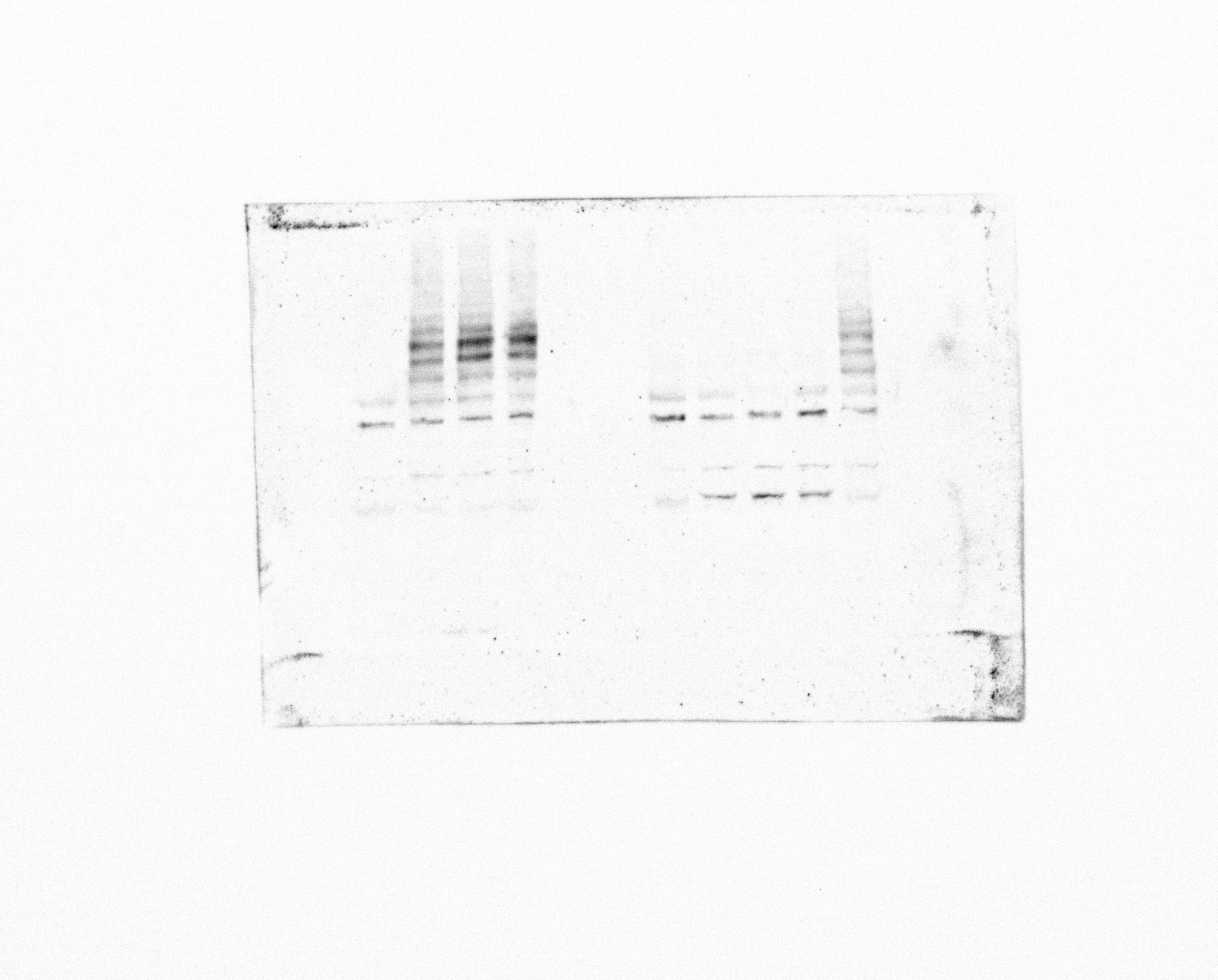

Supplement: Supplementary file 4 — Source data Fig. 3 [file 44318_2025_545_MOESM4_ESM.zip › Fig 3/3E/ MAR soluble/25.04.29_15.16.07_S2_F02.tif]

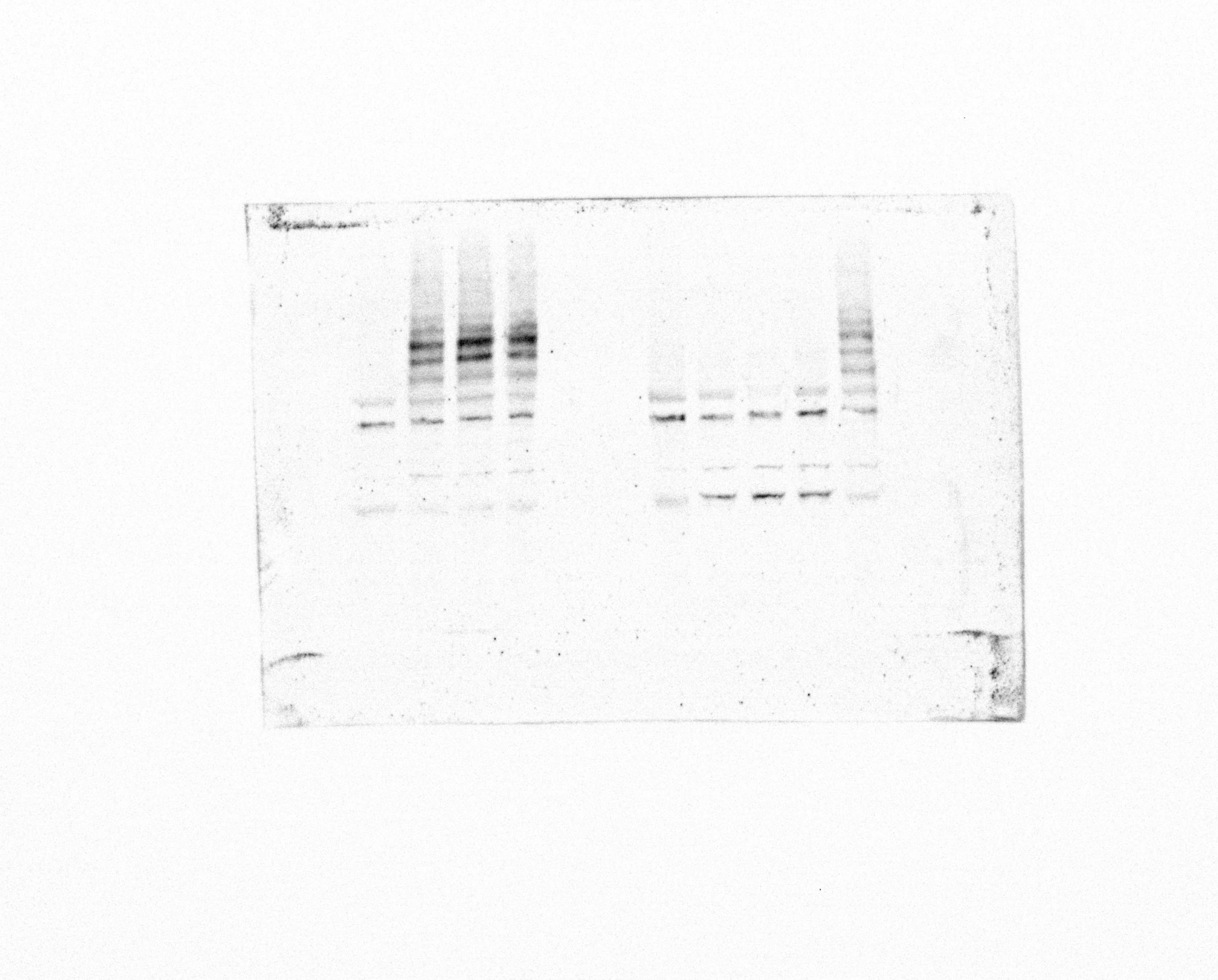

Supplement: Supplementary file 4 — Source data Fig. 3 [file 44318_2025_545_MOESM4_ESM.zip › Fig 3/3E/ MAR soluble/25.04.29_15.21.41.tif]

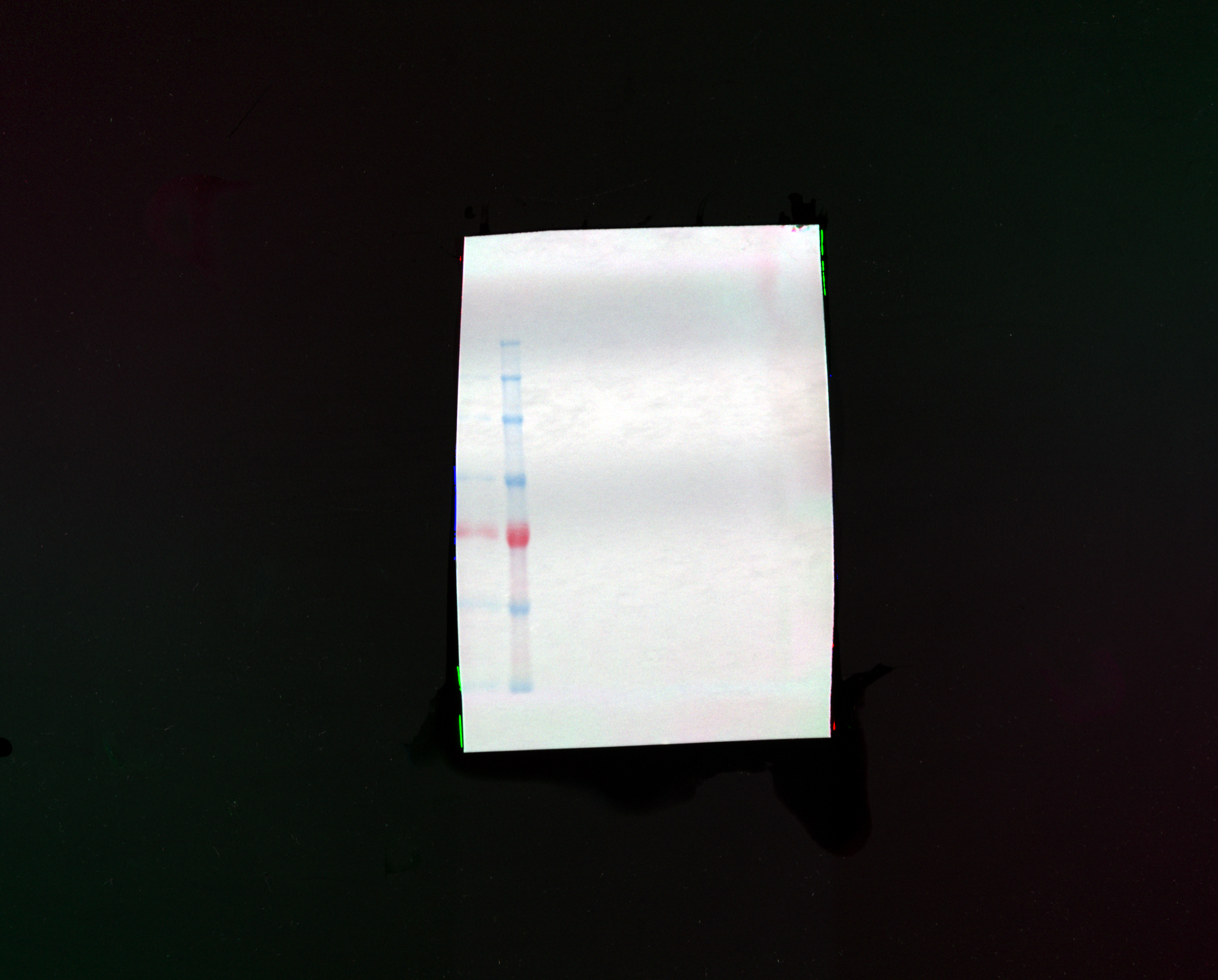

Supplement: Supplementary file 4 — Source data Fig. 3 [file 44318_2025_545_MOESM4_ESM.zip › Fig 3/3E/ATG7/25.04.29_15.31.53_S4_marker_PUB_300.tif]

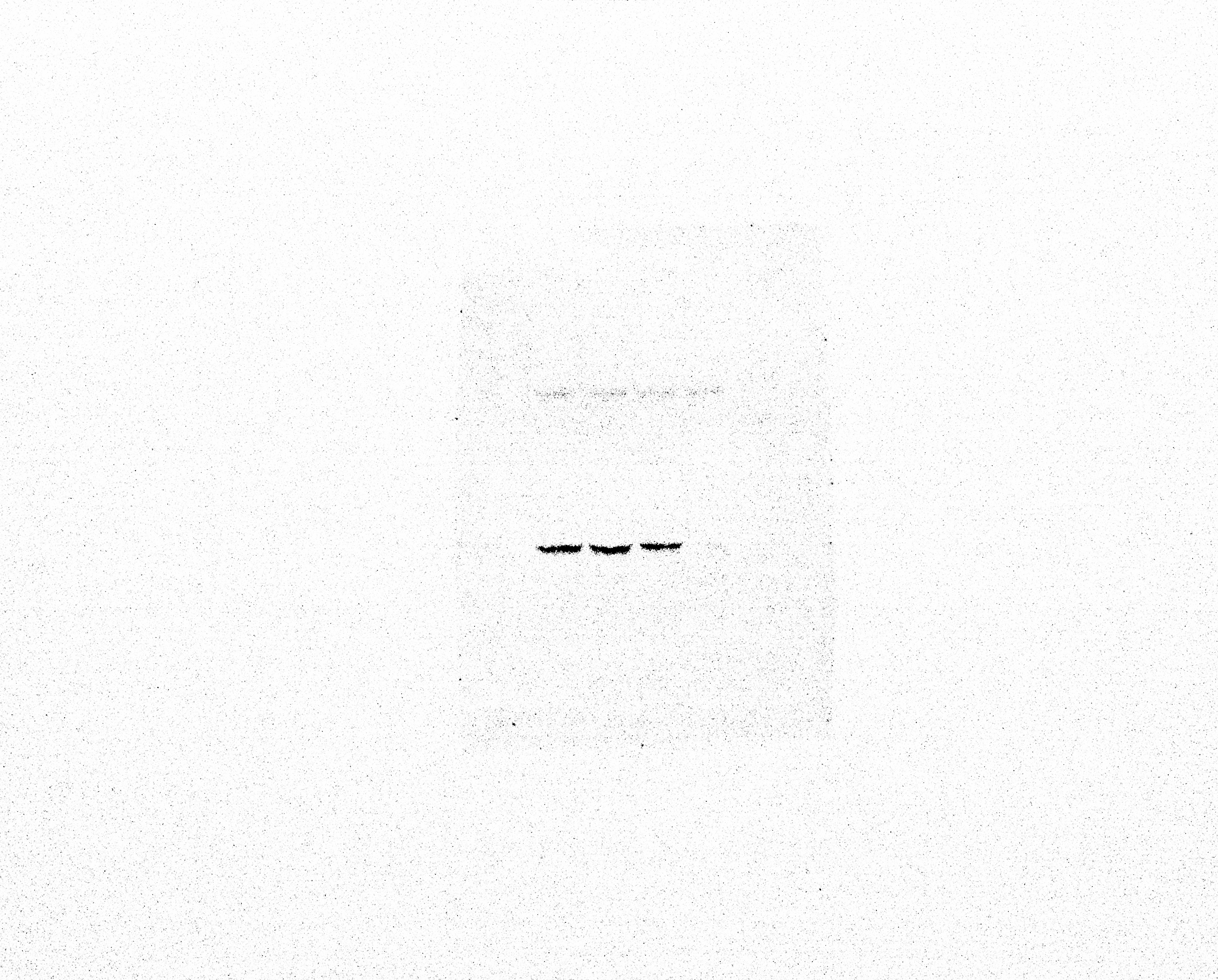

Supplement: Supplementary file 4 — Source data Fig. 3 [file 44318_2025_545_MOESM4_ESM.zip › Fig 3/3E/ATG7/25.04.29_15.31.53_S4_F01_PUB_300.tif]

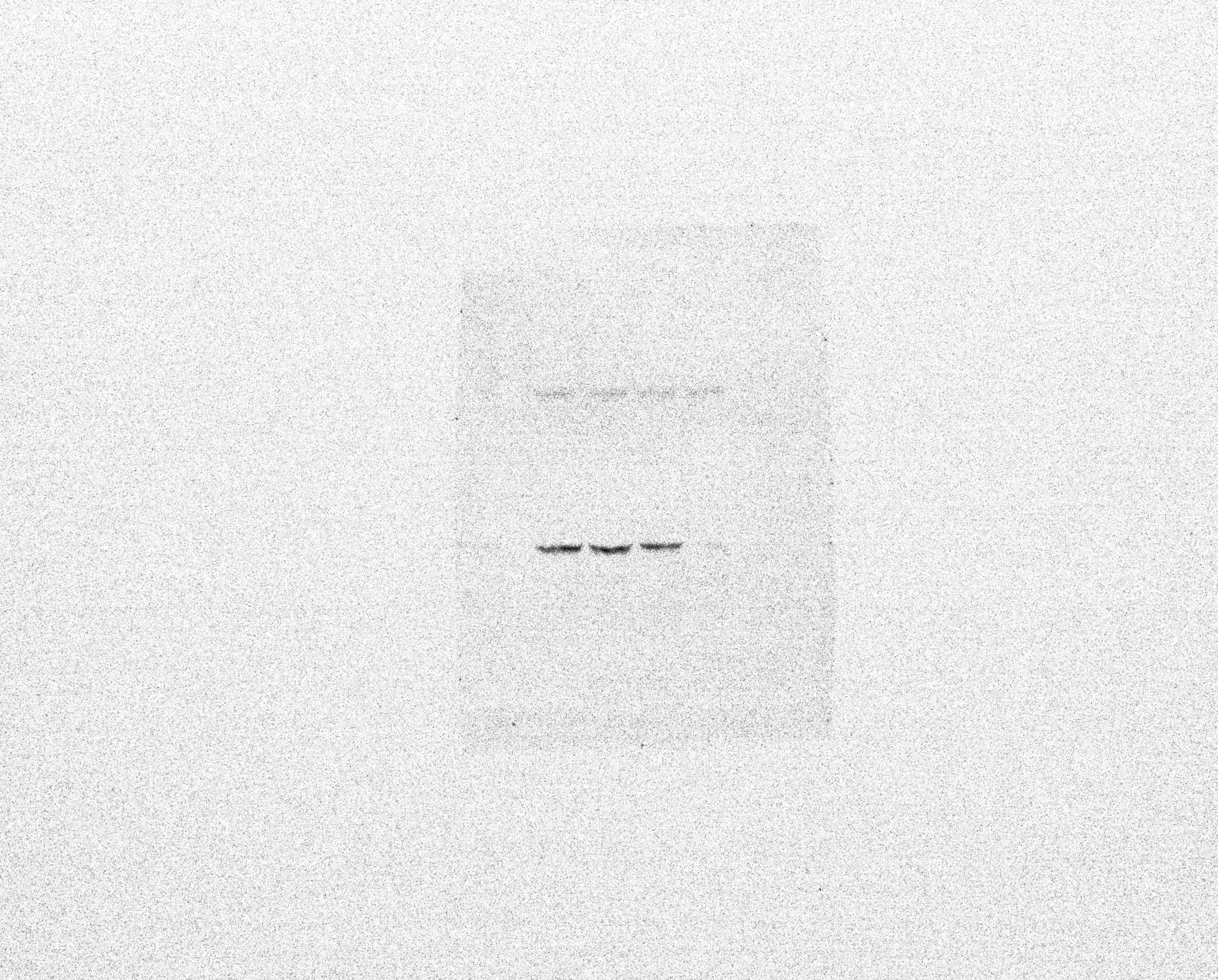

Supplement: Supplementary file 4 — Source data Fig. 3 [file 44318_2025_545_MOESM4_ESM.zip › Fig 3/3E/ATG7/25.04.29_15.31.53_S4_F01.tif]

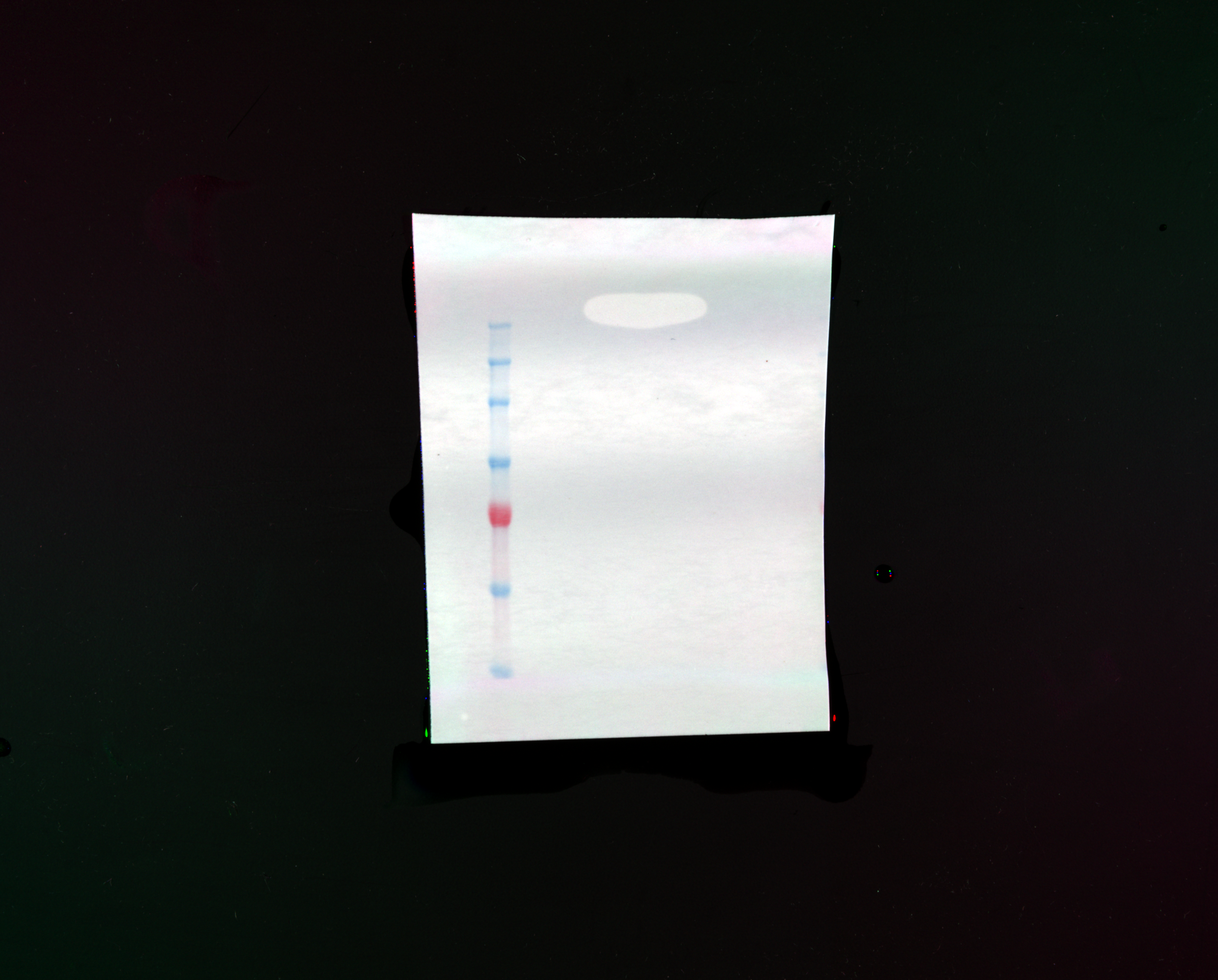

Supplement: Supplementary file 4 — Source data Fig. 3 [file 44318_2025_545_MOESM4_ESM.zip › Fig 3/3E/ATG5/25.04.29_15.38.46_S5_marker_PUB_300.tif]

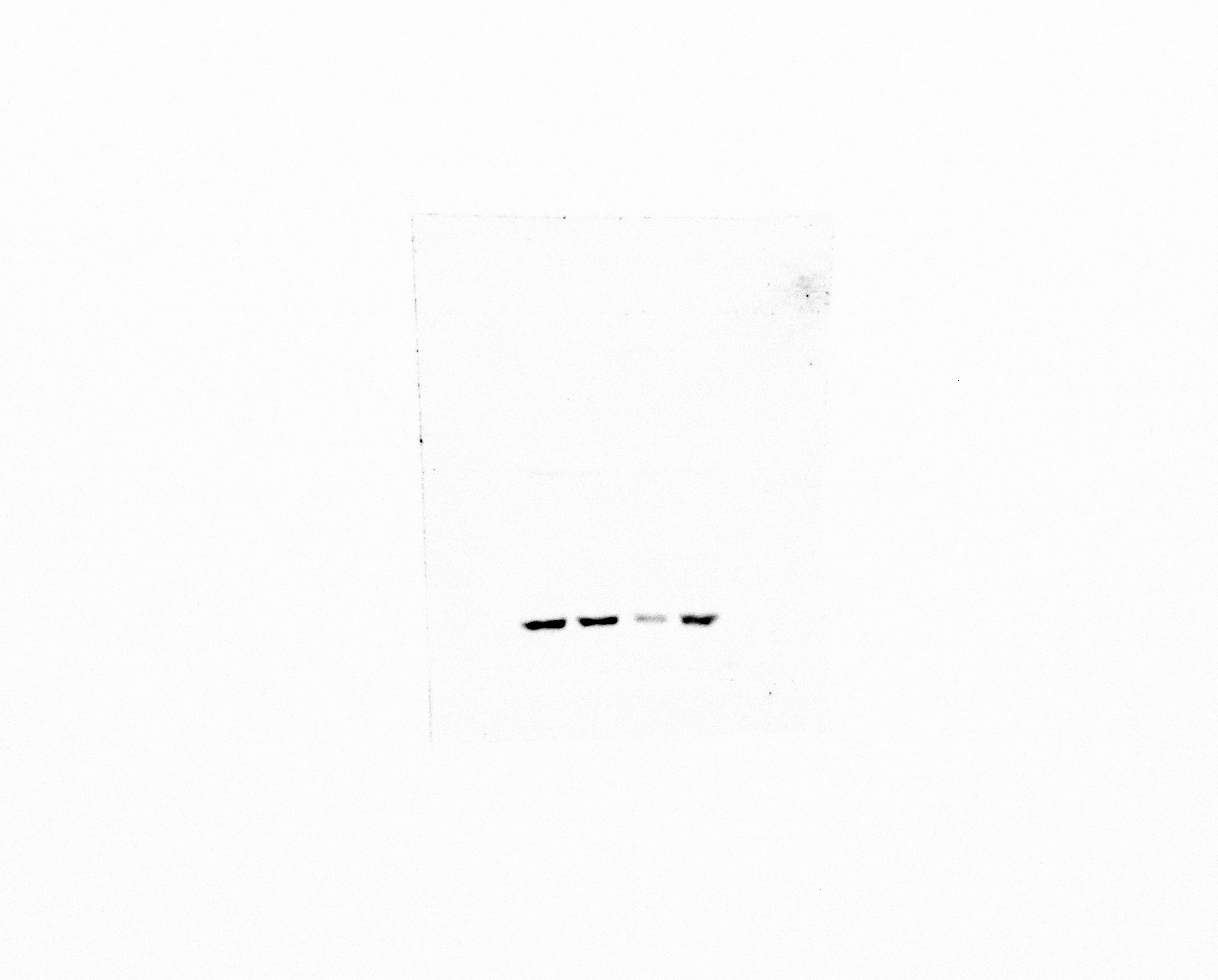

Supplement: Supplementary file 4 — Source data Fig. 3 [file 44318_2025_545_MOESM4_ESM.zip › Fig 3/3E/ATG5/25.04.29_15.38.46_S5_F02_PUB_300.tif]

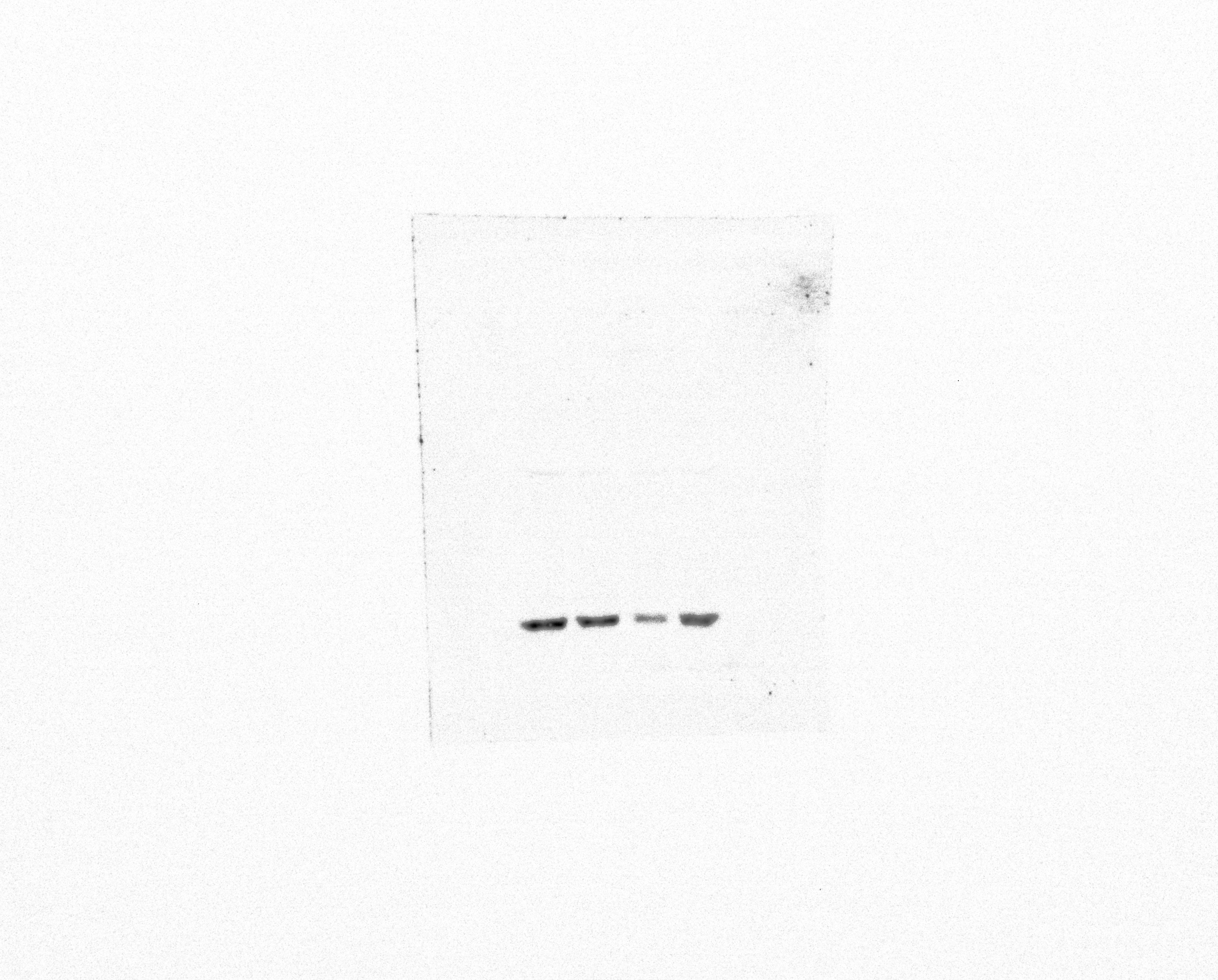

Supplement: Supplementary file 4 — Source data Fig. 3 [file 44318_2025_545_MOESM4_ESM.zip › Fig 3/3E/ATG5/25.04.29_15.38.46_S5_F02.tif]

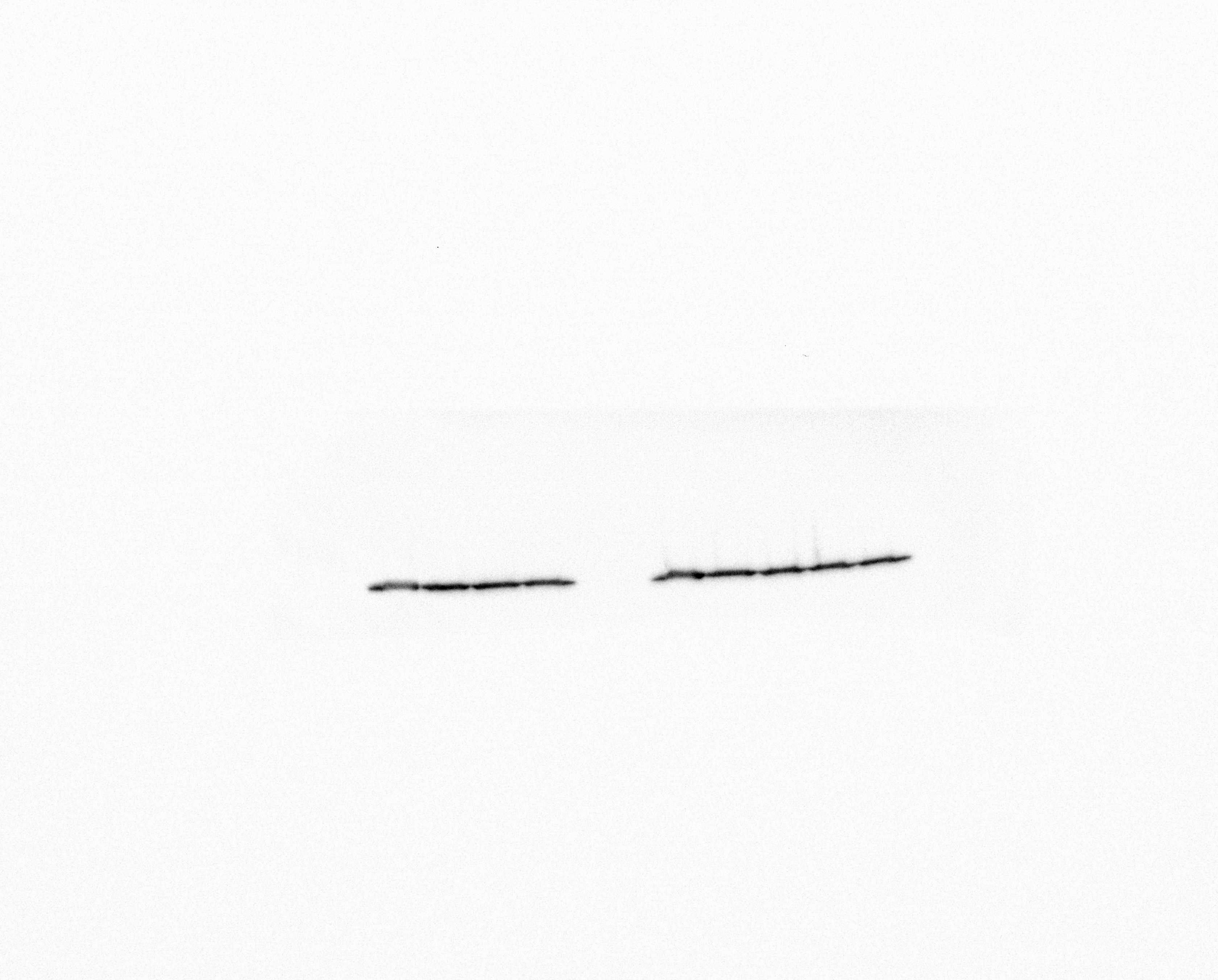

Supplement: Supplementary file 4 — Source data Fig. 3 [file 44318_2025_545_MOESM4_ESM.zip › Fig 3/3E/GAPDH/25.04.29_15.48.18_PUB_300.tif]

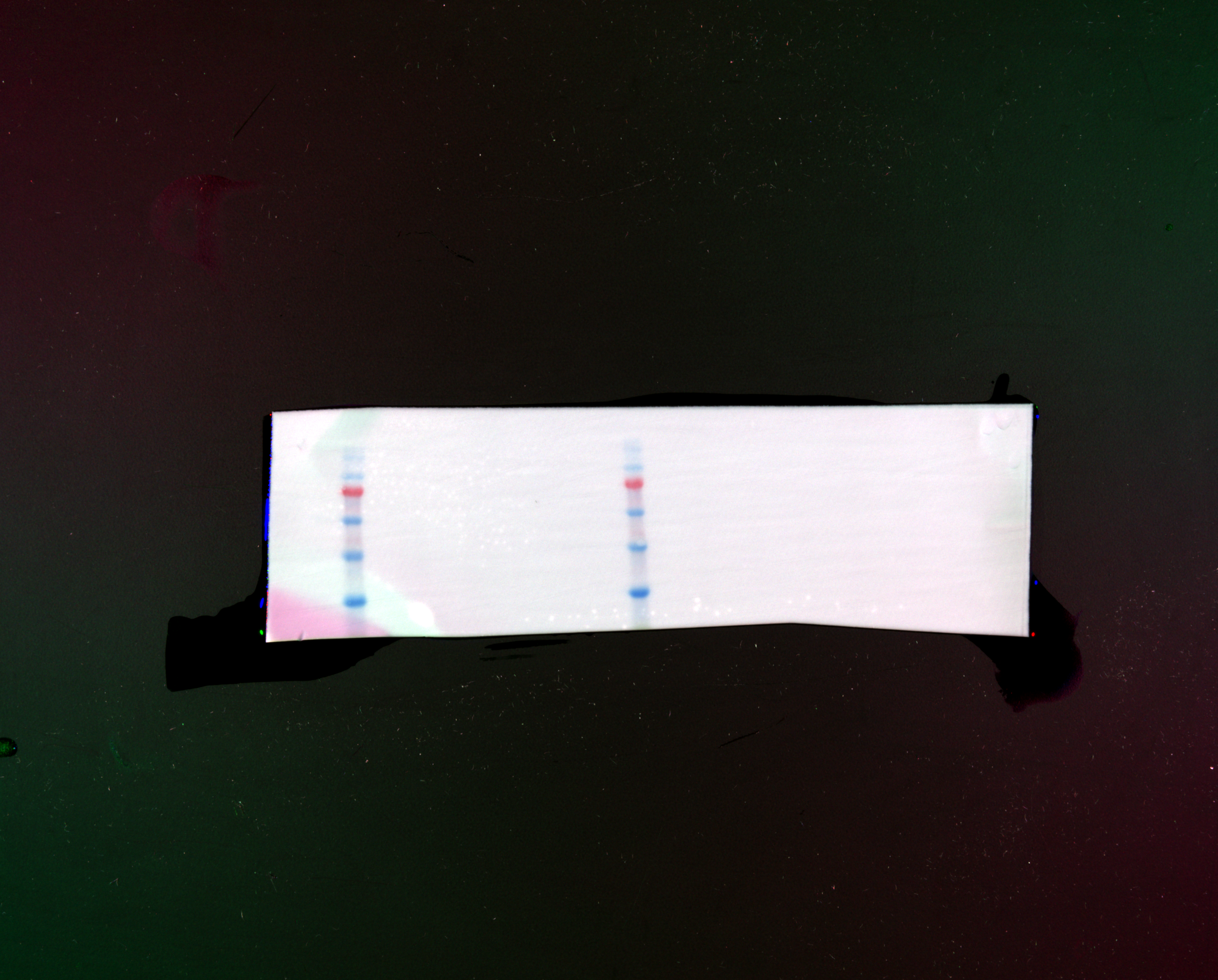

Supplement: Supplementary file 4 — Source data Fig. 3 [file 44318_2025_545_MOESM4_ESM.zip › Fig 3/3E/GAPDH/25.04.29_15.48.18_marker_PUB_300.tif]

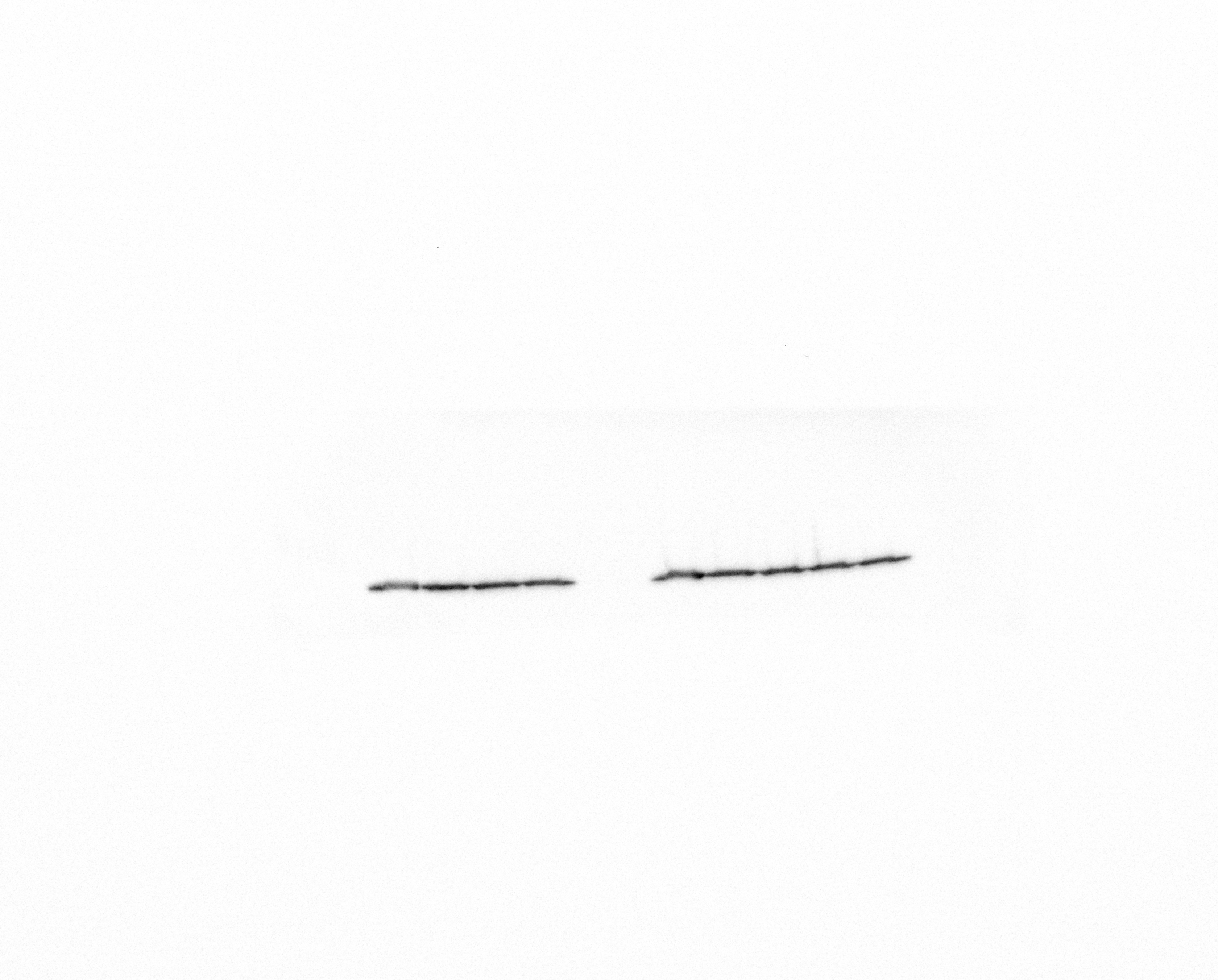

Supplement: Supplementary file 4 — Source data Fig. 3 [file 44318_2025_545_MOESM4_ESM.zip › Fig 3/3E/GAPDH/25.04.29_15.48.18.tif]

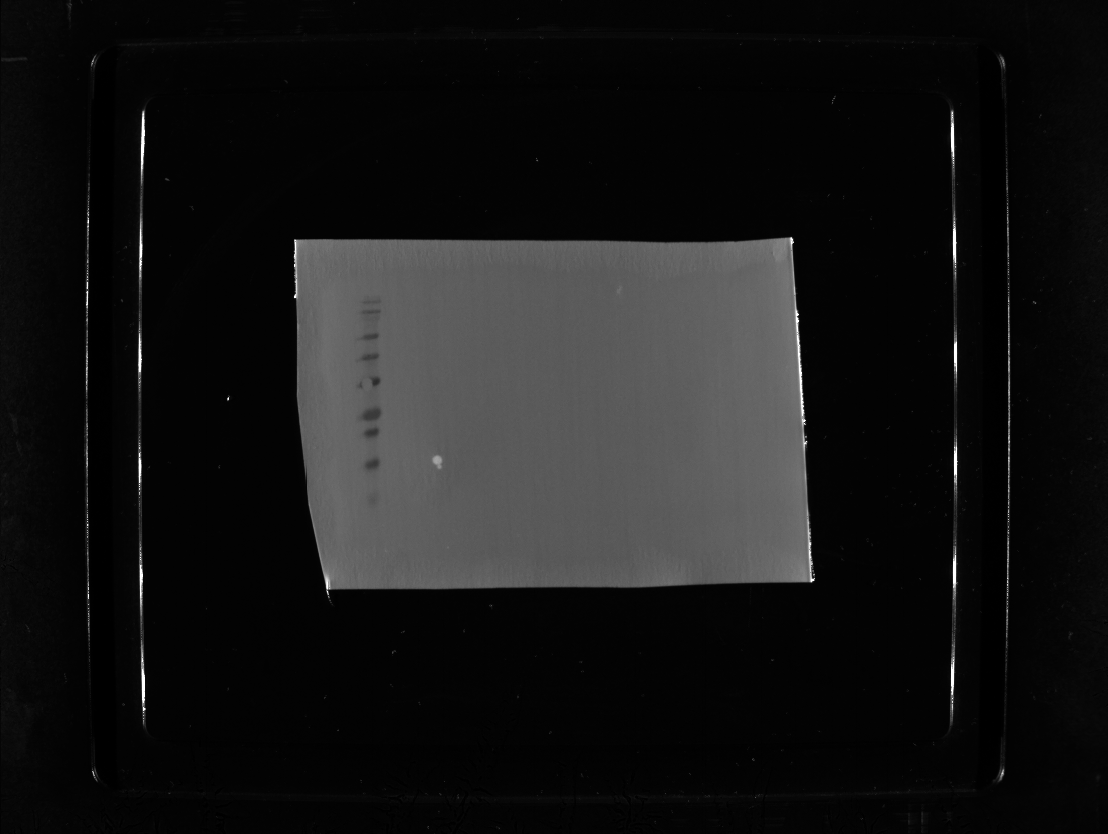

Supplement: Supplementary file 4 — Source data Fig. 3 [file 44318_2025_545_MOESM4_ESM.zip › Fig 3/3D/3D RD/LC3B/2024-0628-103944.tif]

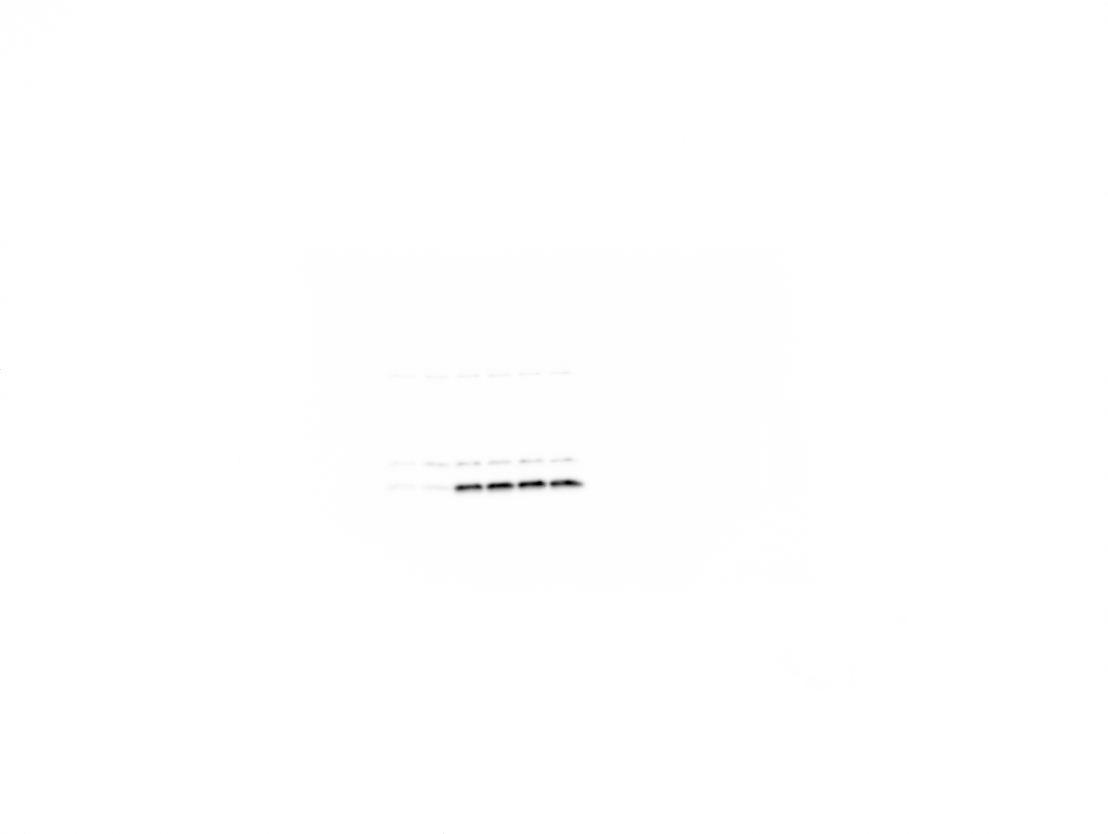

Supplement: Supplementary file 4 — Source data Fig. 3 [file 44318_2025_545_MOESM4_ESM.zip › Fig 3/3D/3D RD/LC3B/2024-0628-103945.tif]

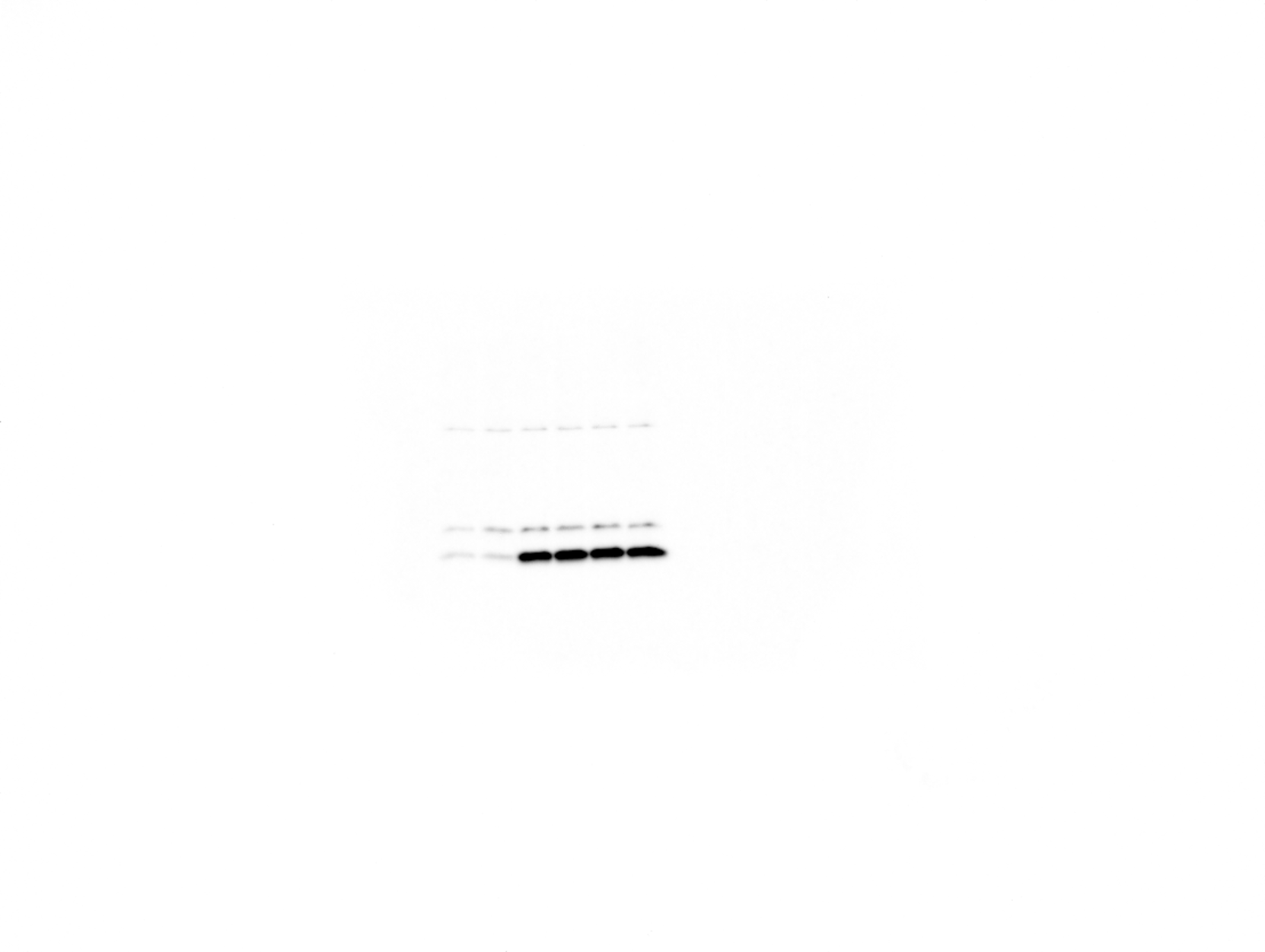

Supplement: Supplementary file 4 — Source data Fig. 3 [file 44318_2025_545_MOESM4_ESM.zip › Fig 3/3D/3D RD/LC3B/2024-0628-103945_pub.tif]

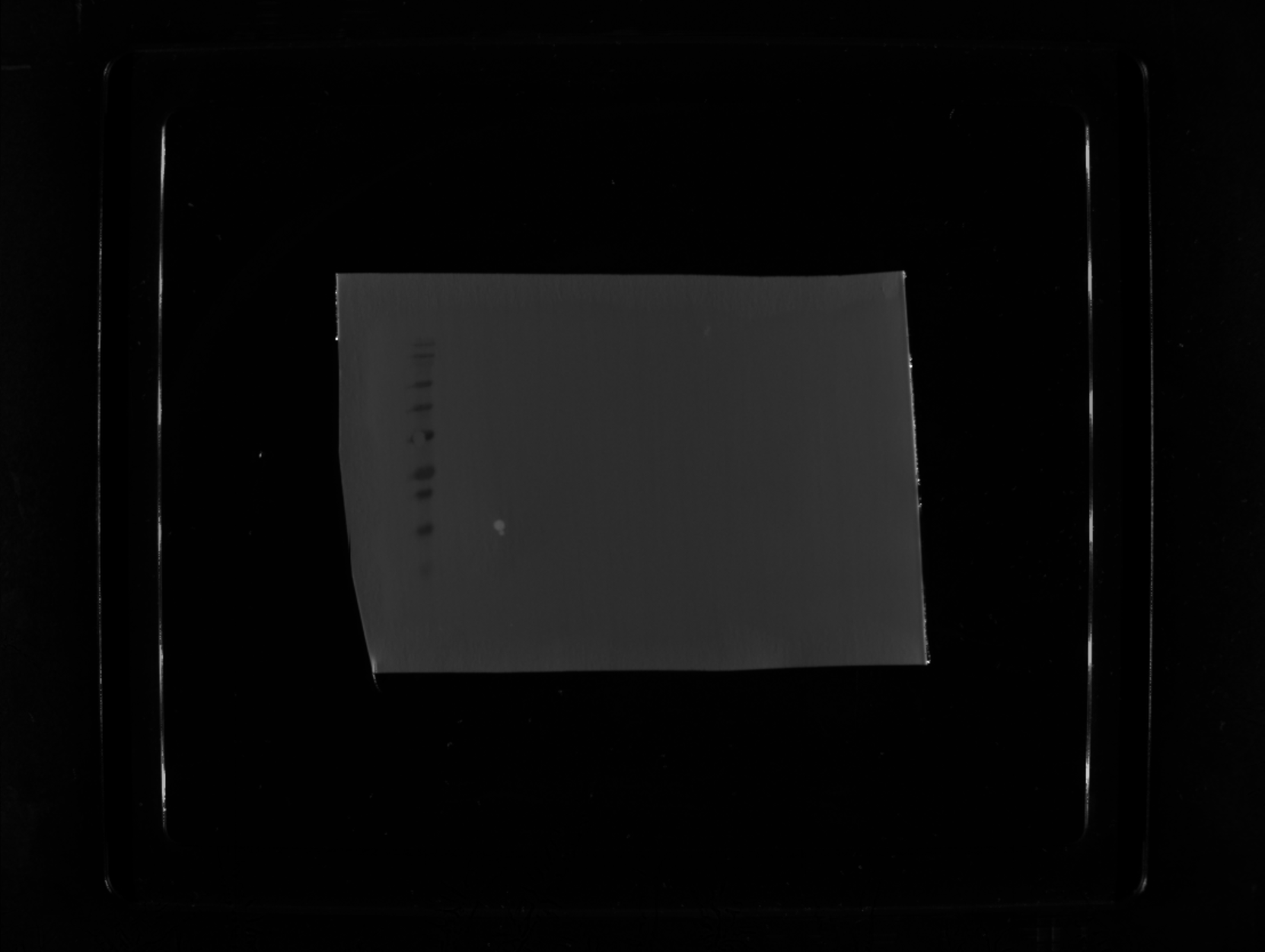

Supplement: Supplementary file 4 — Source data Fig. 3 [file 44318_2025_545_MOESM4_ESM.zip › Fig 3/3D/3D RD/LC3B/2024-0628-103944_pub.tif]

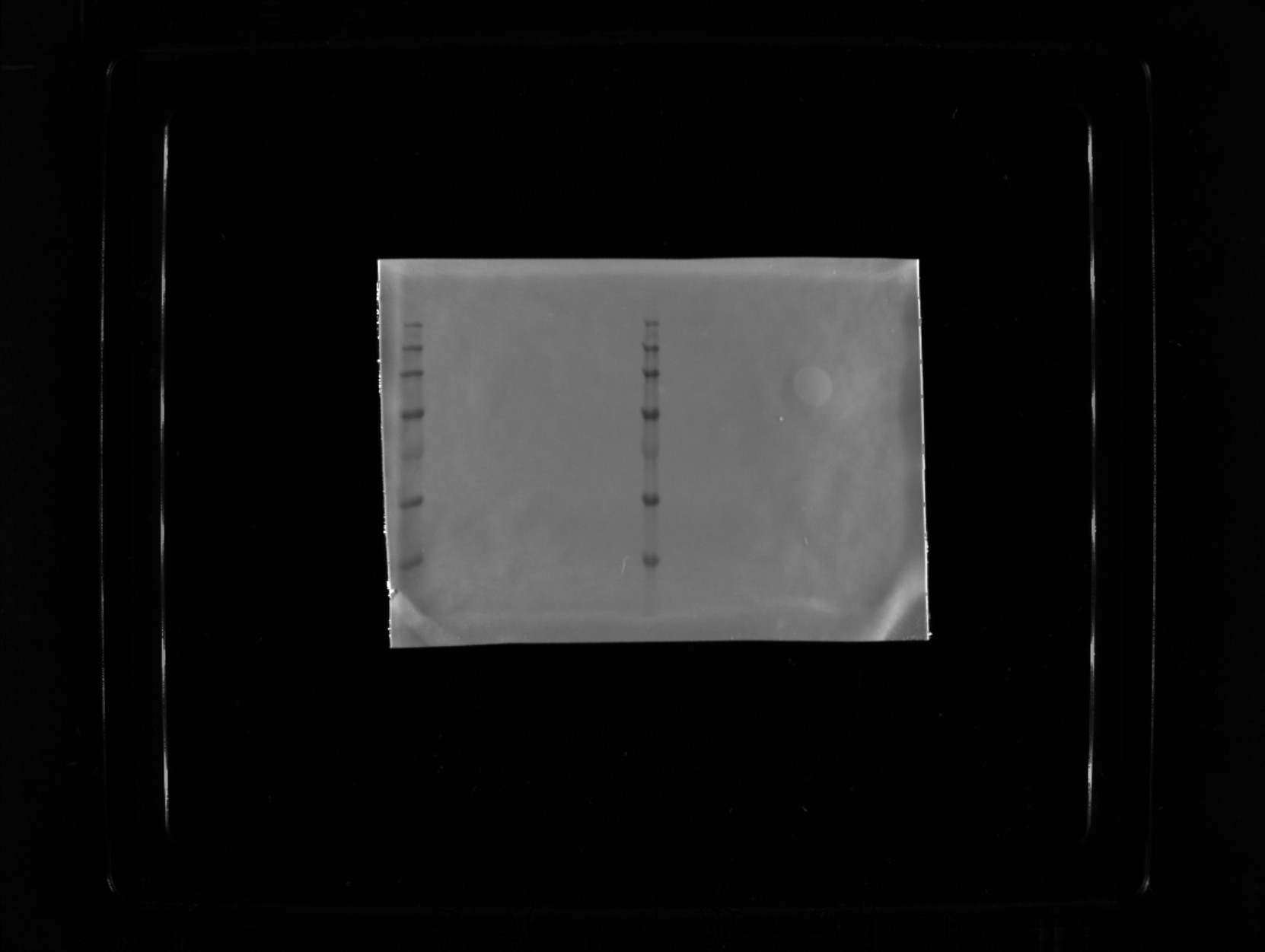

Supplement: Supplementary file 4 — Source data Fig. 3 [file 44318_2025_545_MOESM4_ESM.zip › Fig 3/3D/3D RD/ADPr/2024-0628-102957_pub.tif]

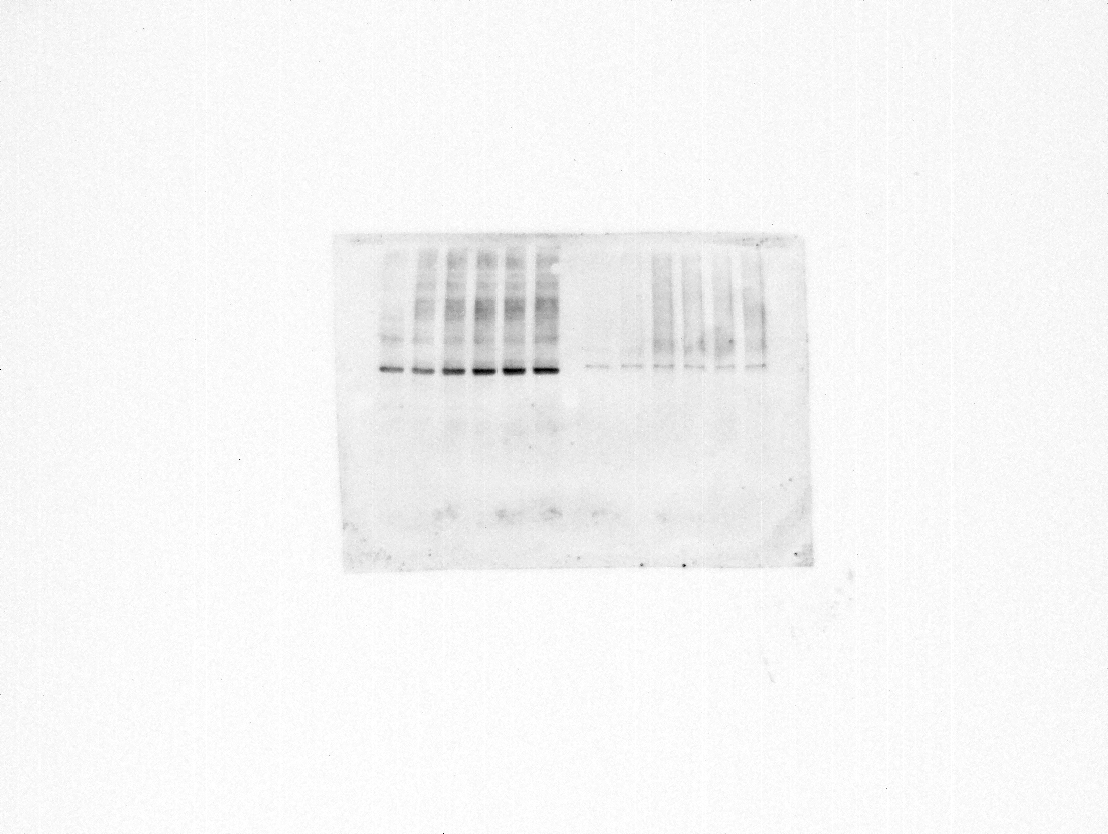

Supplement: Supplementary file 4 — Source data Fig. 3 [file 44318_2025_545_MOESM4_ESM.zip › Fig 3/3D/3D RD/ADPr/2024-0628-102958.tif]

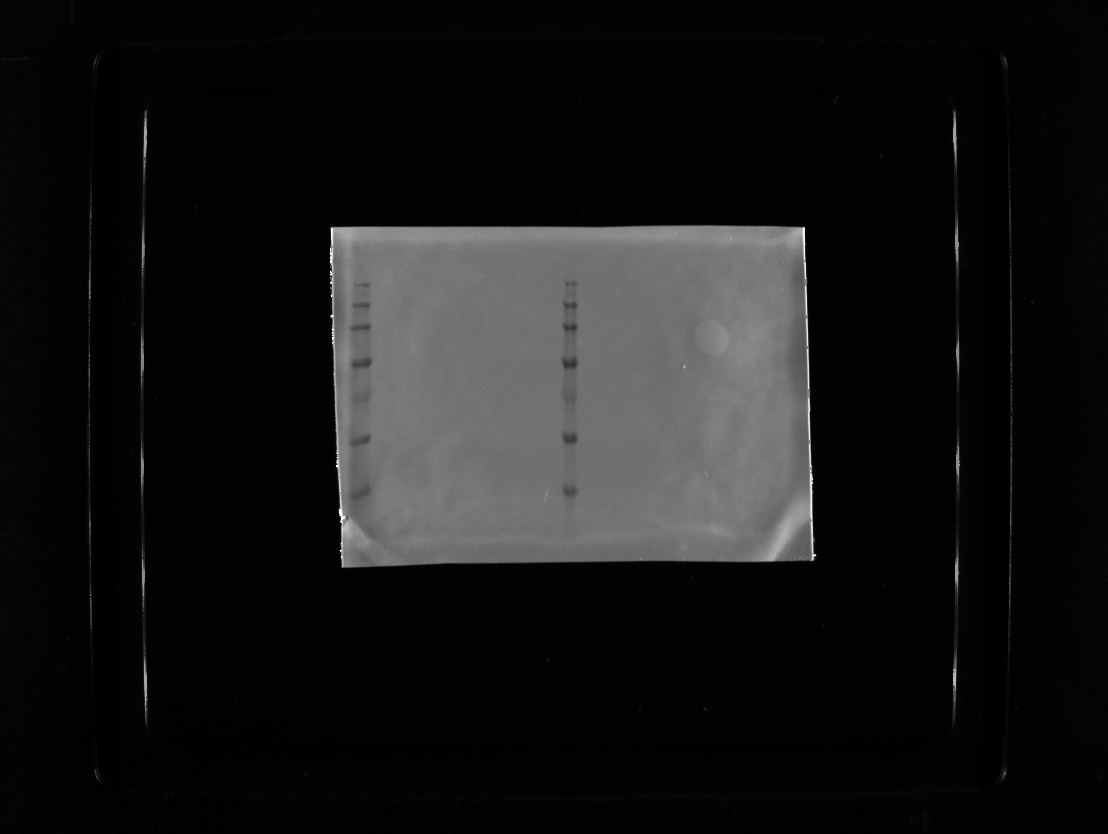

Supplement: Supplementary file 4 — Source data Fig. 3 [file 44318_2025_545_MOESM4_ESM.zip › Fig 3/3D/3D RD/ADPr/2024-0628-102957.tif]

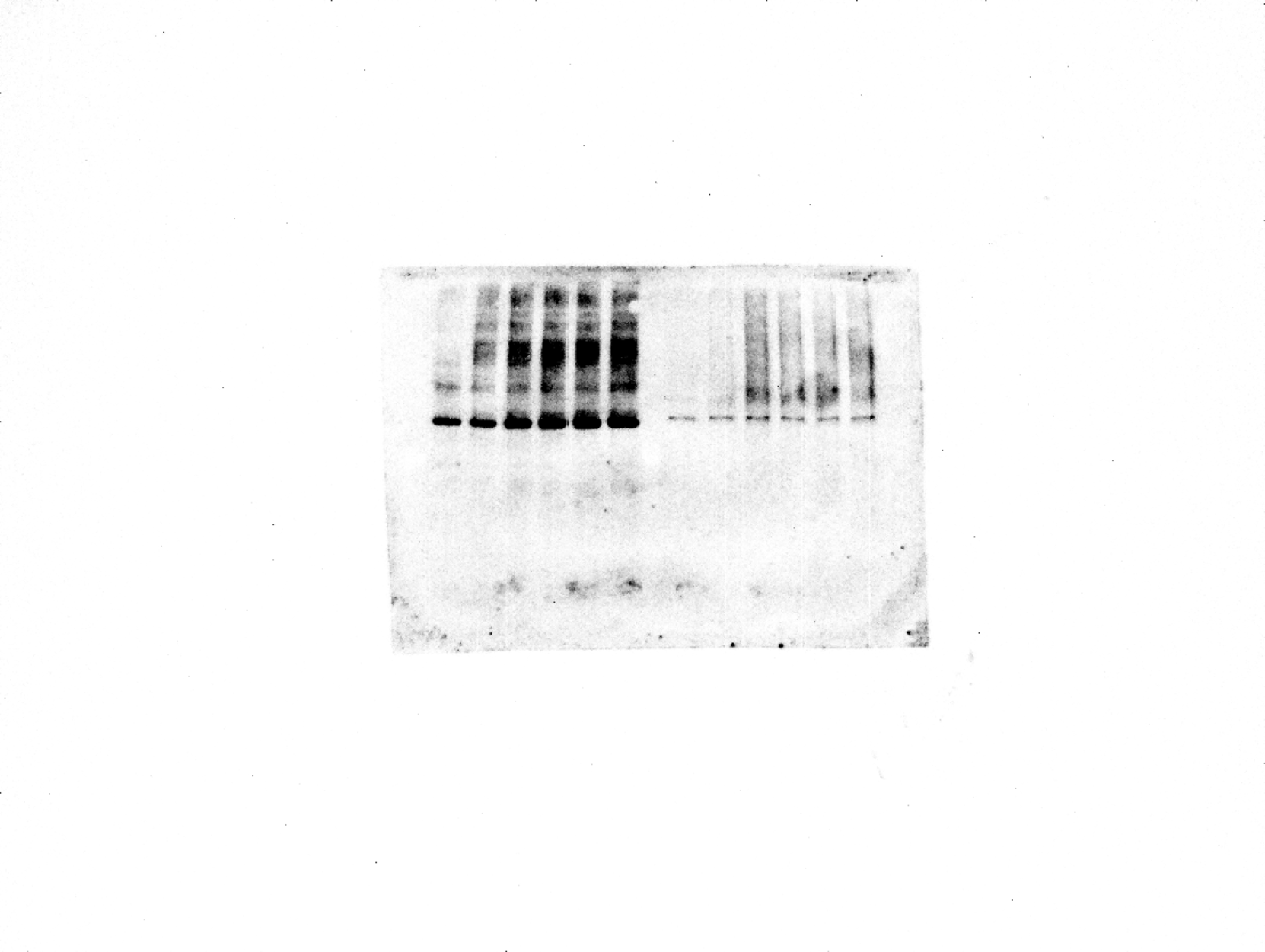

Supplement: Supplementary file 4 — Source data Fig. 3 [file 44318_2025_545_MOESM4_ESM.zip › Fig 3/3D/3D RD/ADPr/2024-0628-102958_pub.tif]

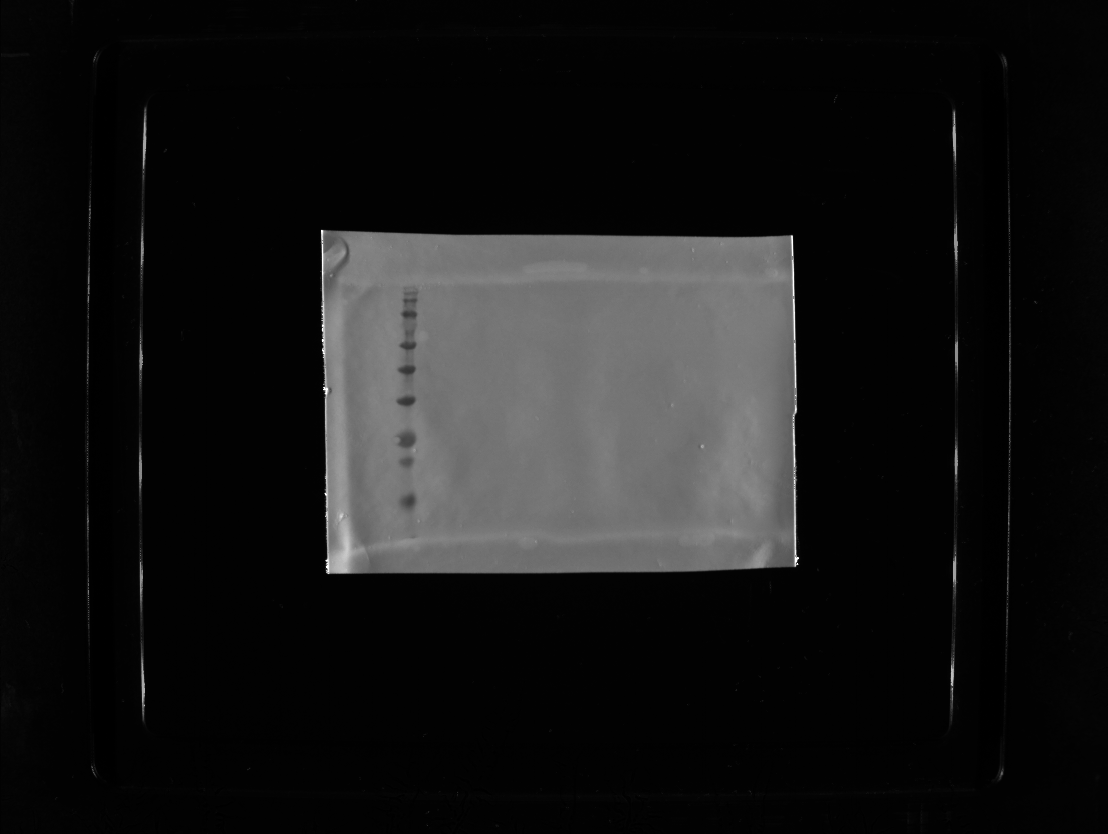

Supplement: Supplementary file 4 — Source data Fig. 3 [file 44318_2025_545_MOESM4_ESM.zip › Fig 3/3D/3D RD/Tub/2024-0628-104343.tif]

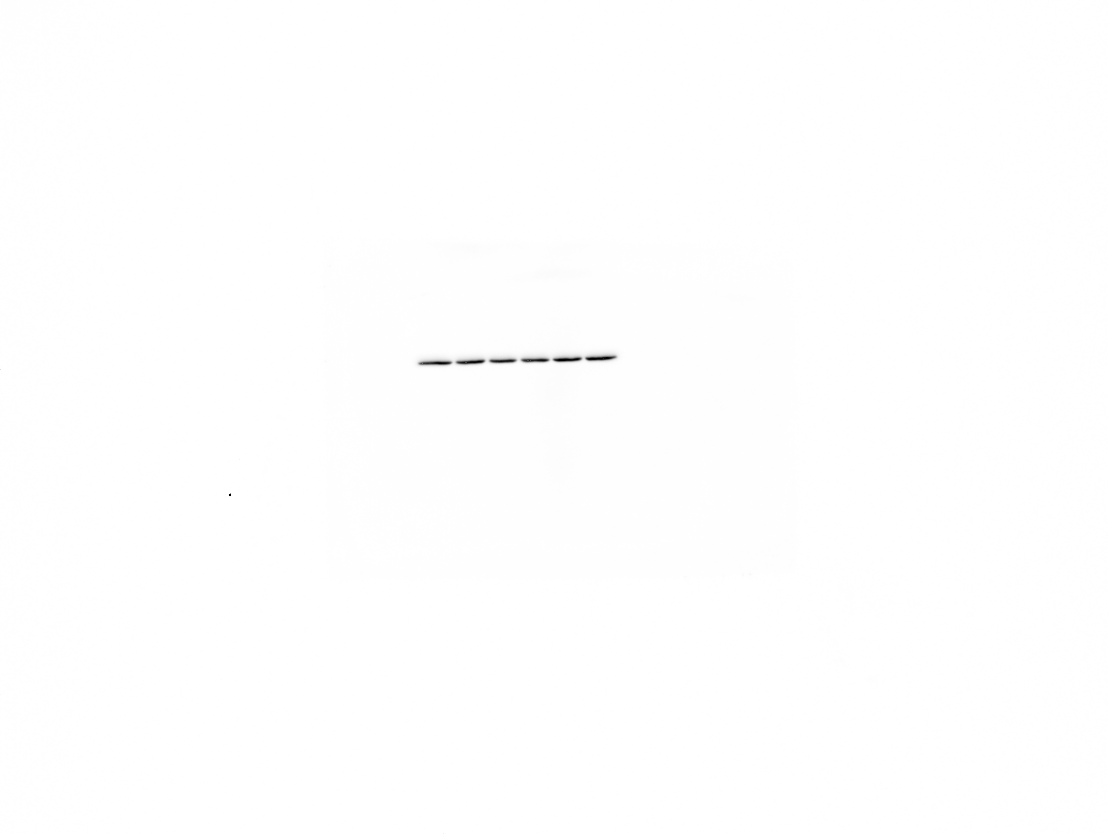

Supplement: Supplementary file 4 — Source data Fig. 3 [file 44318_2025_545_MOESM4_ESM.zip › Fig 3/3D/3D RD/Tub/2024-0628-104344.tif]

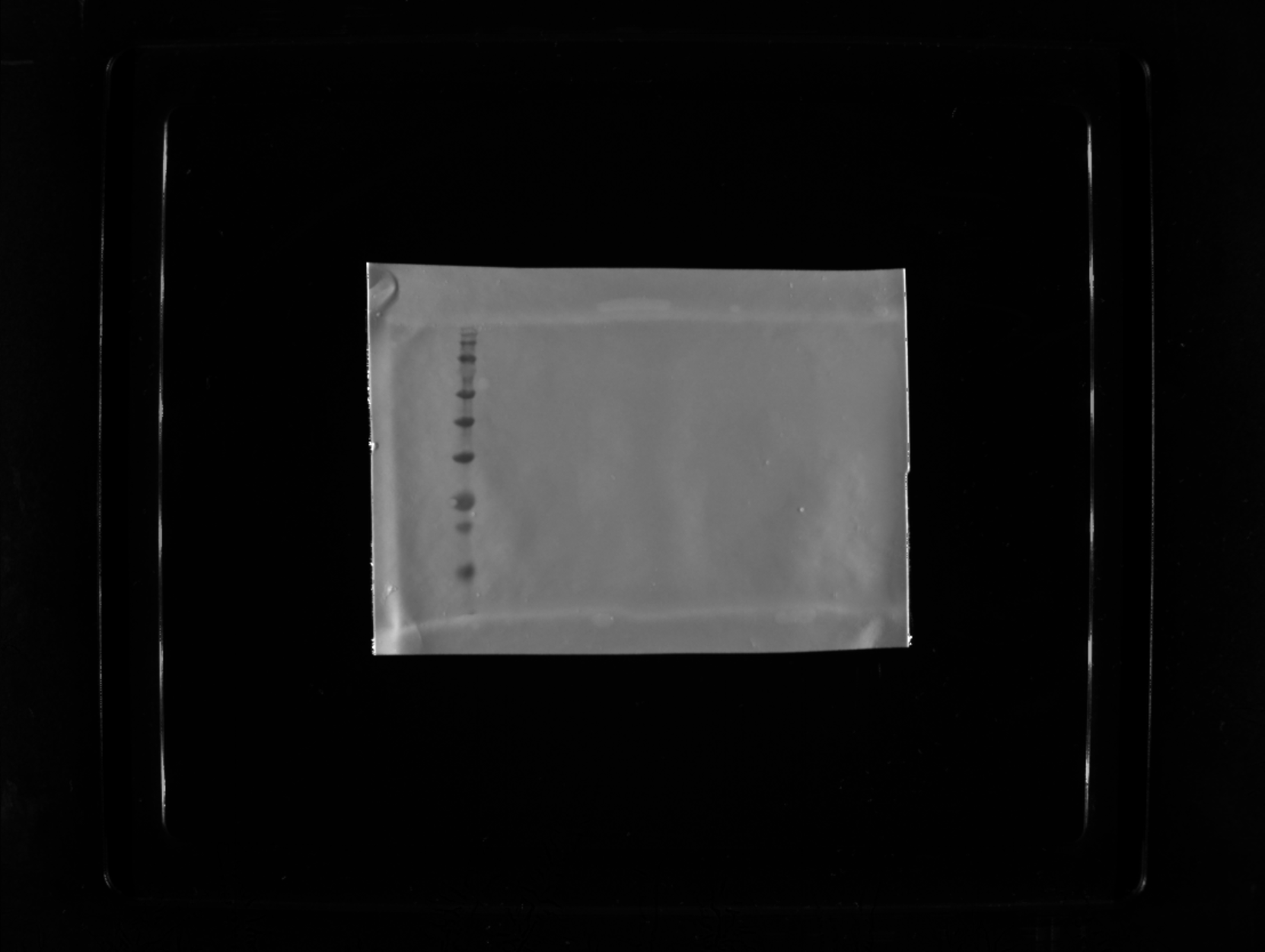

Supplement: Supplementary file 4 — Source data Fig. 3 [file 44318_2025_545_MOESM4_ESM.zip › Fig 3/3D/3D RD/Tub/2024-0628-104343_pub.tif]

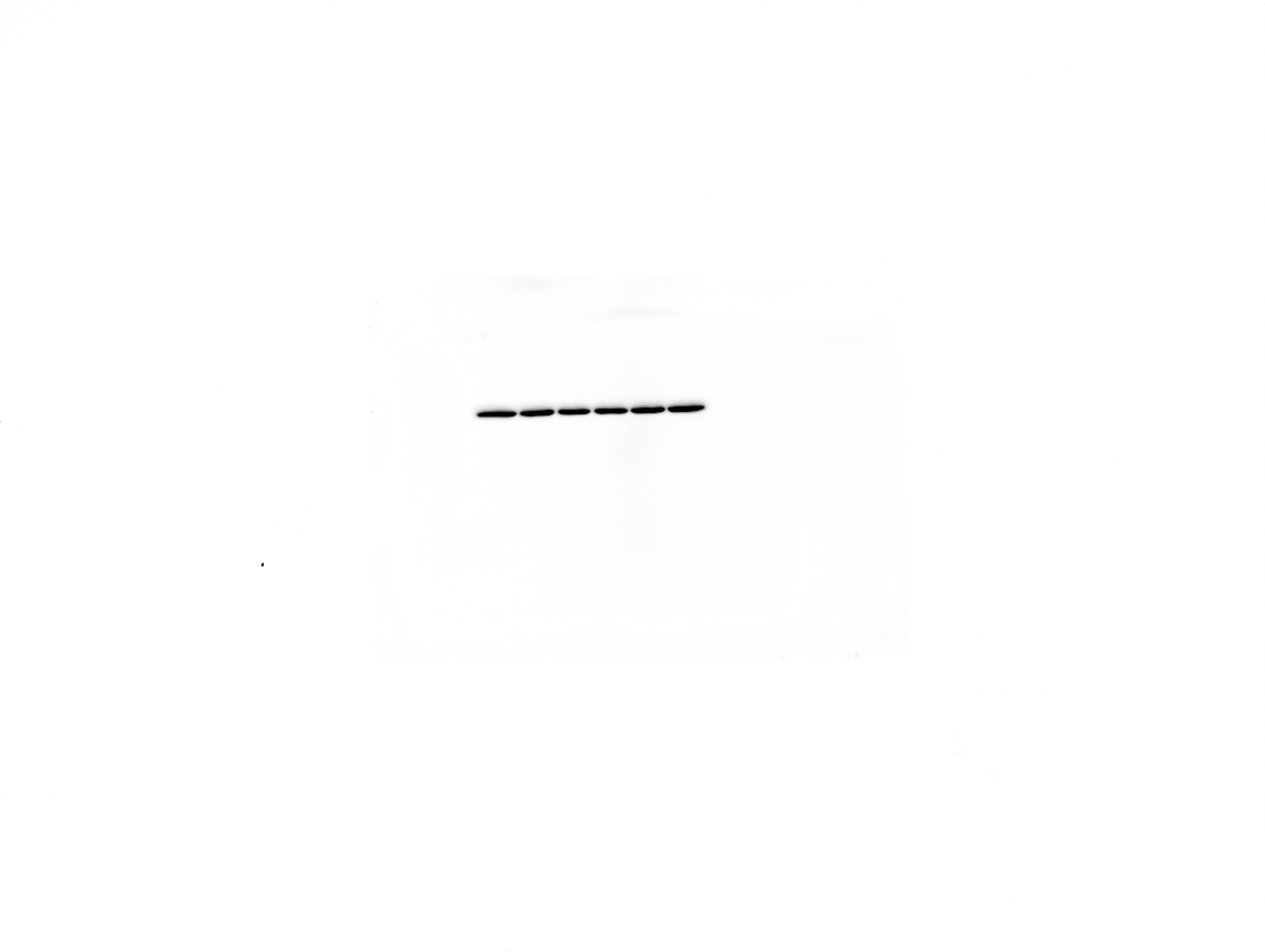

Supplement: Supplementary file 4 — Source data Fig. 3 [file 44318_2025_545_MOESM4_ESM.zip › Fig 3/3D/3D RD/Tub/2024-0628-104344_pub.tif]

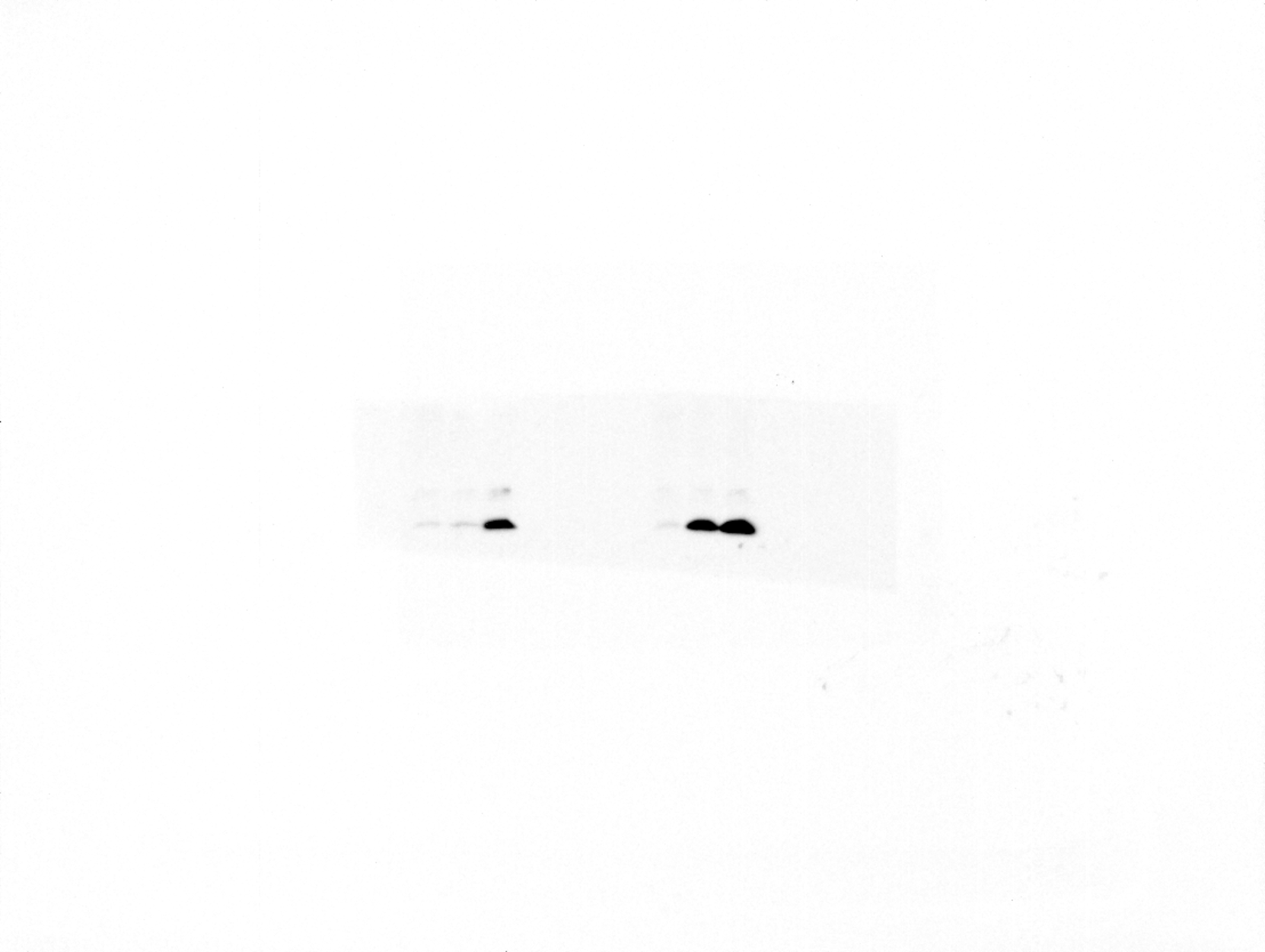

Supplement: Supplementary file 4 — Source data Fig. 3 [file 44318_2025_545_MOESM4_ESM.zip › Fig 3/3D/3D HeLa/LC3B/2024-0801-132342_pub.tif]

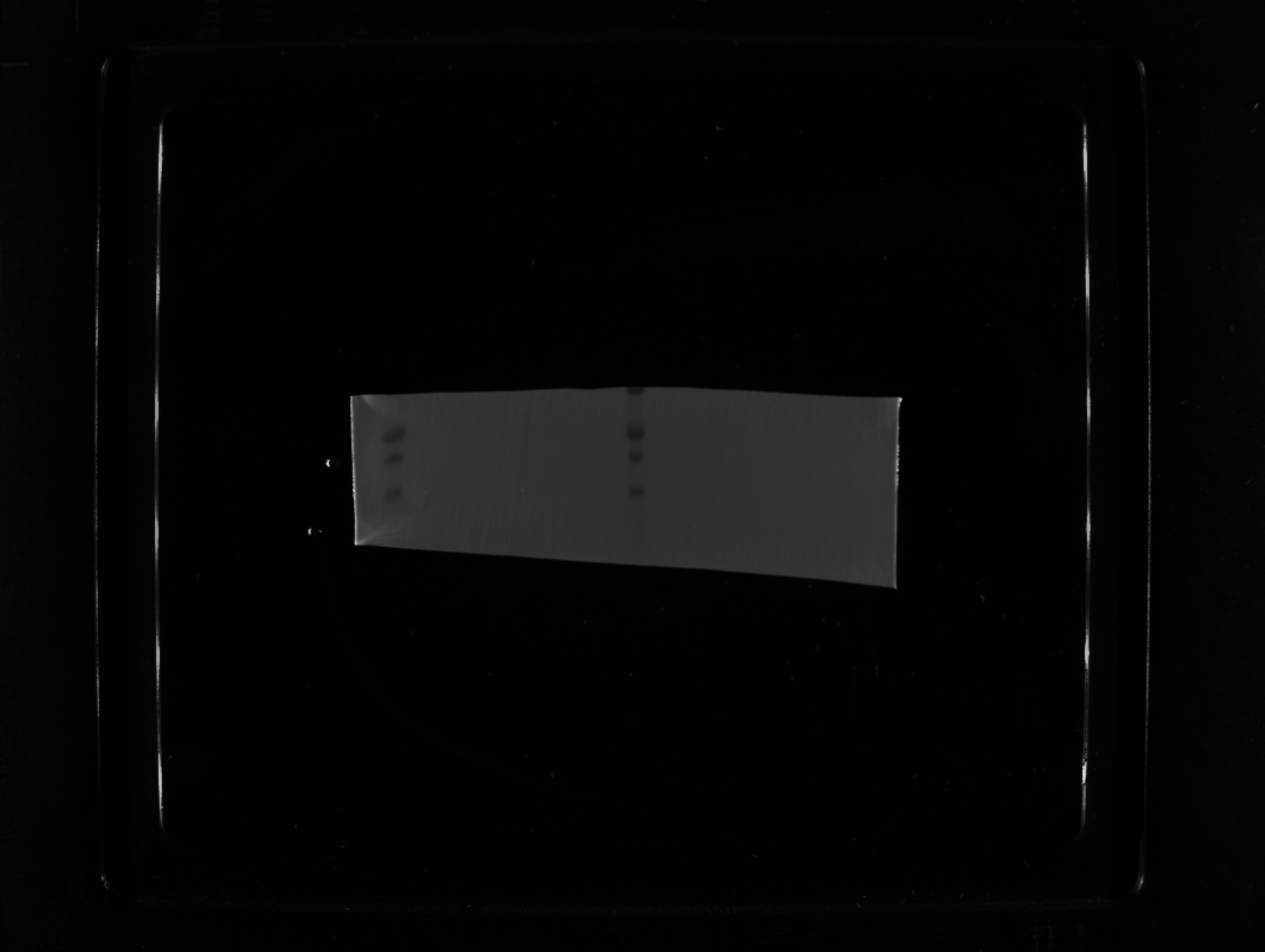

Supplement: Supplementary file 4 — Source data Fig. 3 [file 44318_2025_545_MOESM4_ESM.zip › Fig 3/3D/3D HeLa/LC3B/2024-0801-132340_pub.tif]

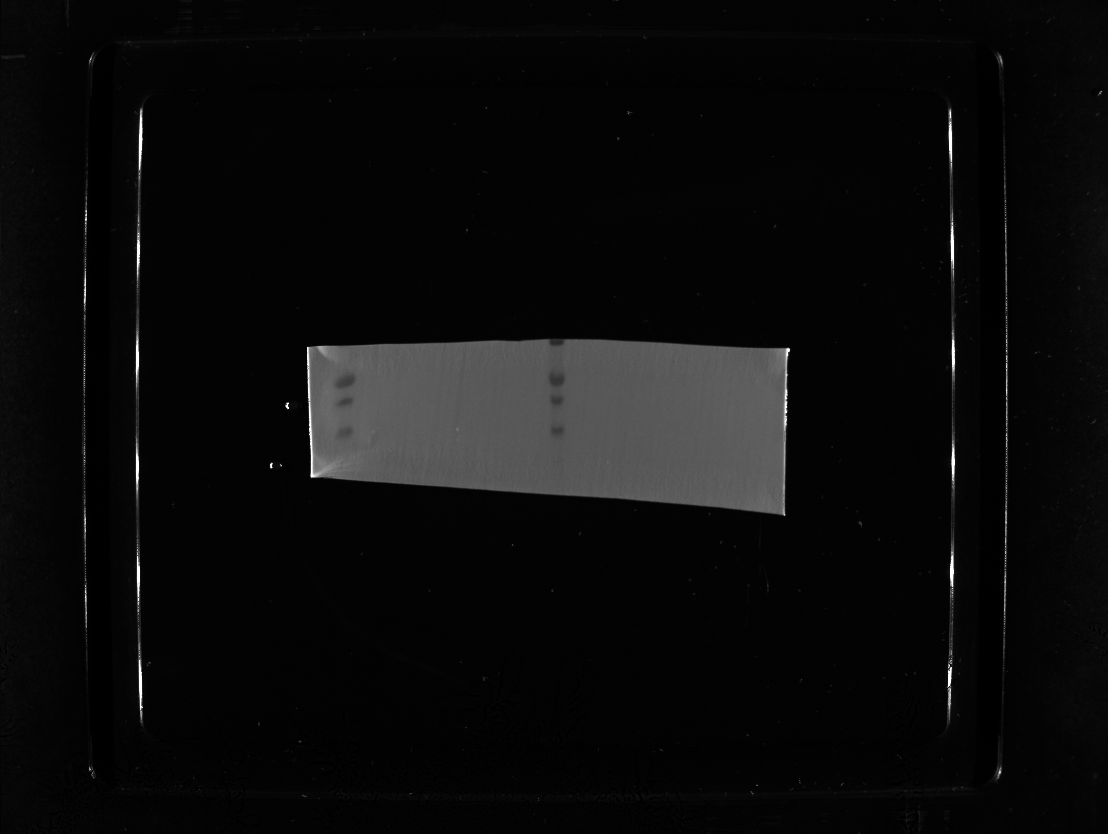

Supplement: Supplementary file 4 — Source data Fig. 3 [file 44318_2025_545_MOESM4_ESM.zip › Fig 3/3D/3D HeLa/LC3B/2024-0801-132340.tif]

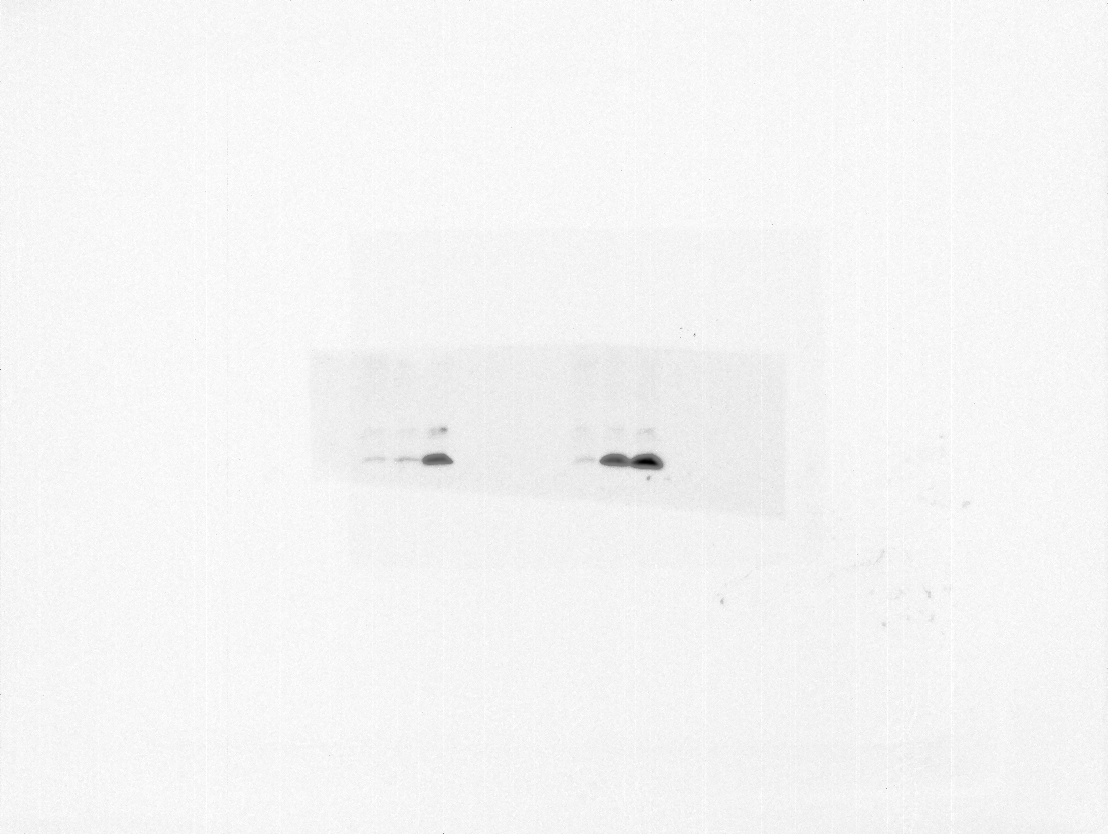

Supplement: Supplementary file 4 — Source data Fig. 3 [file 44318_2025_545_MOESM4_ESM.zip › Fig 3/3D/3D HeLa/LC3B/2024-0801-132342.tif]

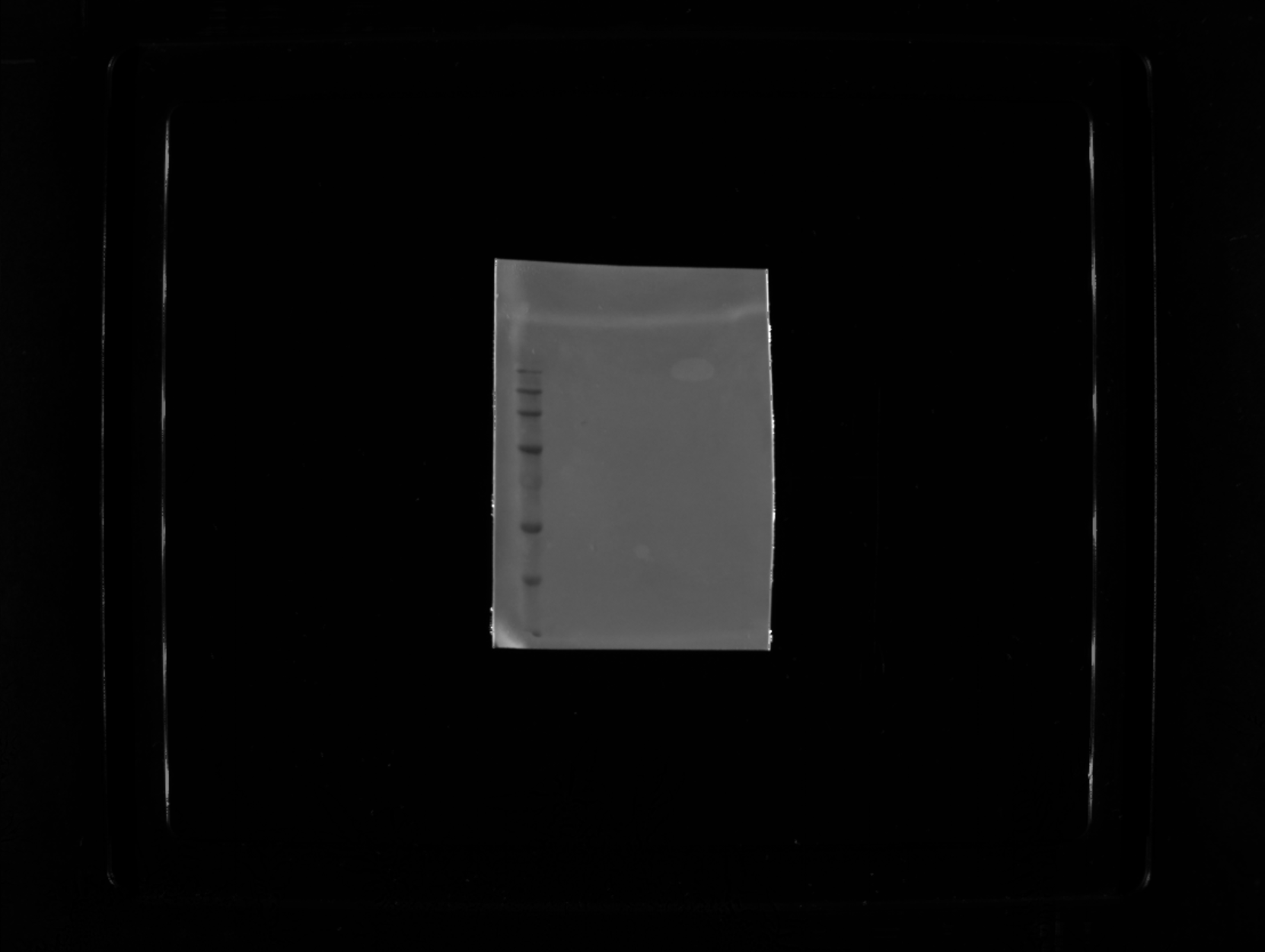

Supplement: Supplementary file 4 — Source data Fig. 3 [file 44318_2025_545_MOESM4_ESM.zip › Fig 3/3D/3D HeLa/ADPr/2024-0801-131123_pub.tif]

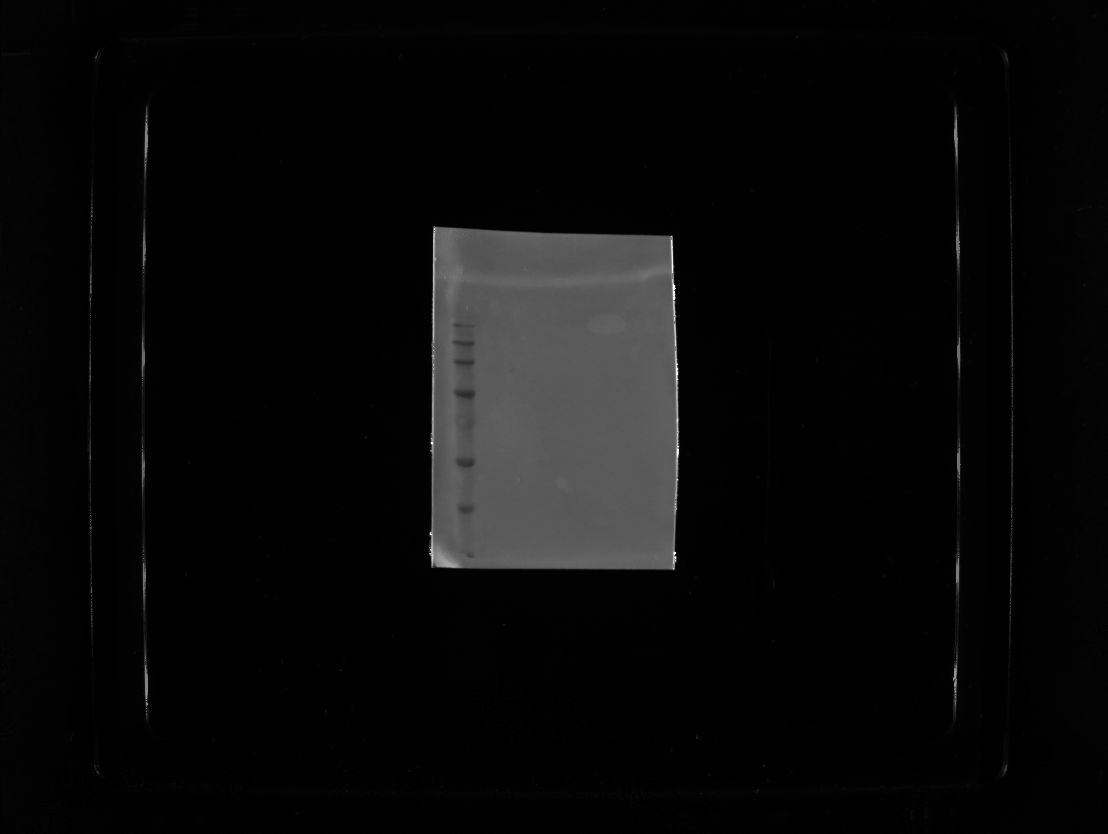

Supplement: Supplementary file 4 — Source data Fig. 3 [file 44318_2025_545_MOESM4_ESM.zip › Fig 3/3D/3D HeLa/ADPr/2024-0801-131123.tif]

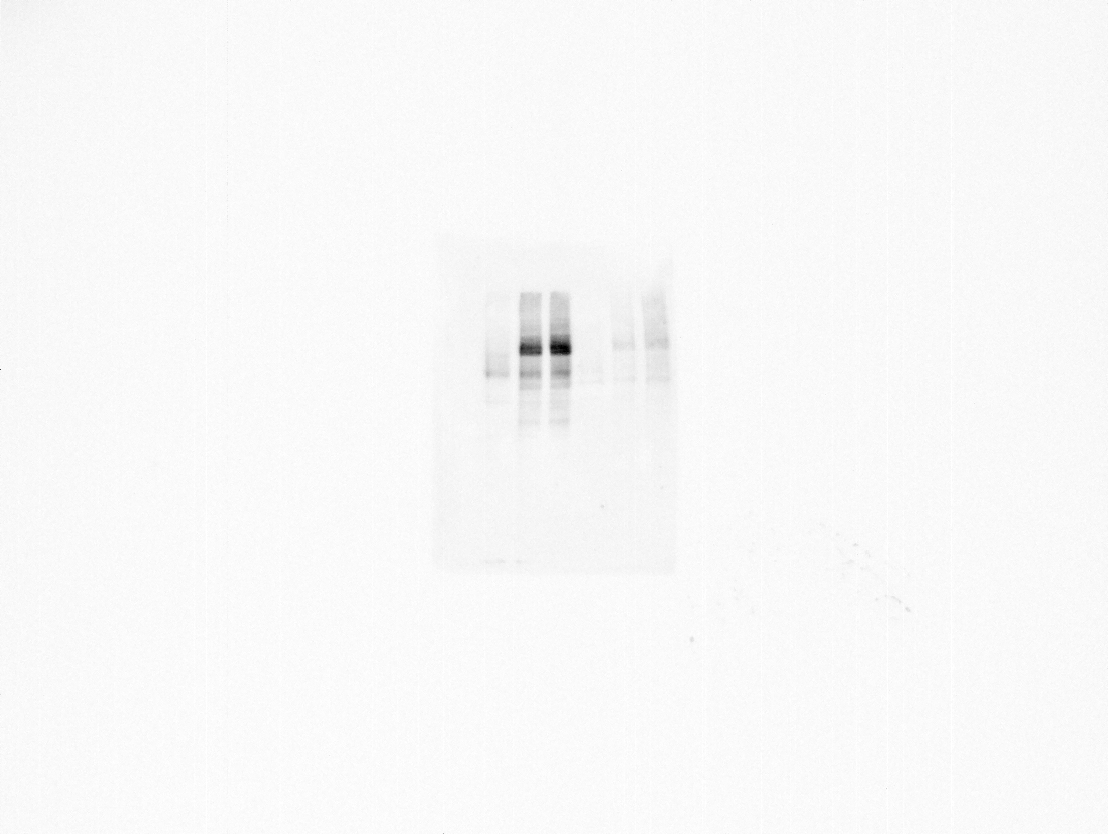

Supplement: Supplementary file 4 — Source data Fig. 3 [file 44318_2025_545_MOESM4_ESM.zip › Fig 3/3D/3D HeLa/ADPr/2024-0801-131127.tif]

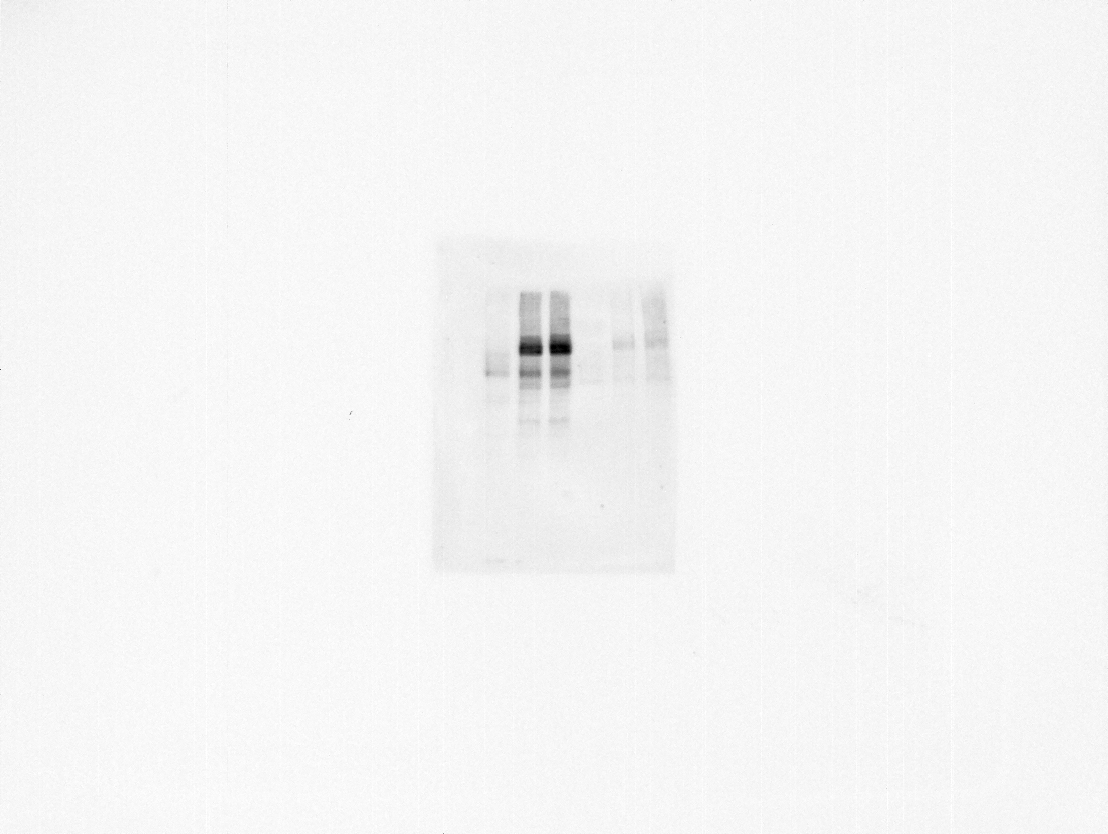

Supplement: Supplementary file 4 — Source data Fig. 3 [file 44318_2025_545_MOESM4_ESM.zip › Fig 3/3D/3D HeLa/ADPr/2024-0801-131125.tif]

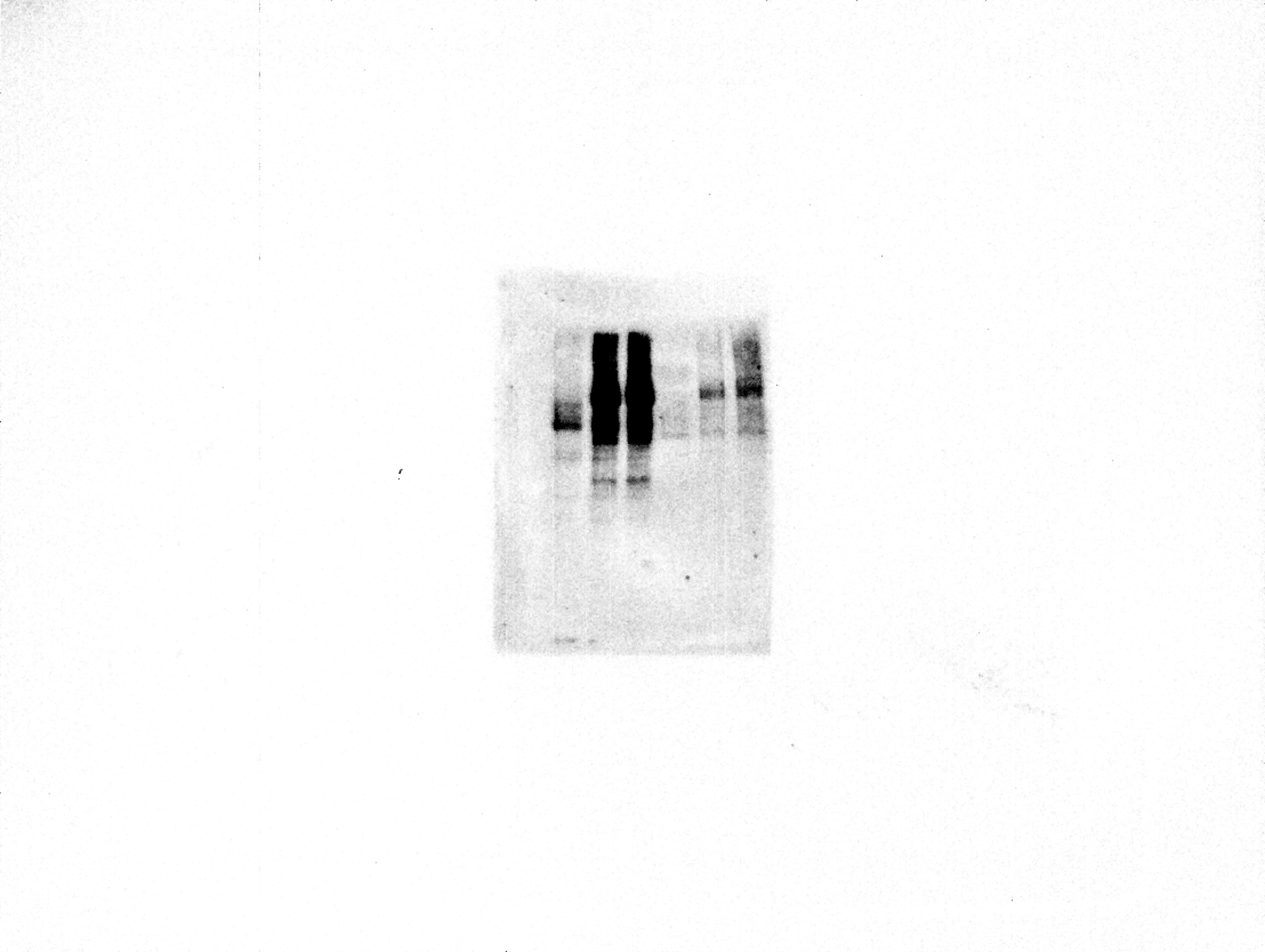

Supplement: Supplementary file 4 — Source data Fig. 3 [file 44318_2025_545_MOESM4_ESM.zip › Fig 3/3D/3D HeLa/ADPr/2024-0801-131125_pub.tif]

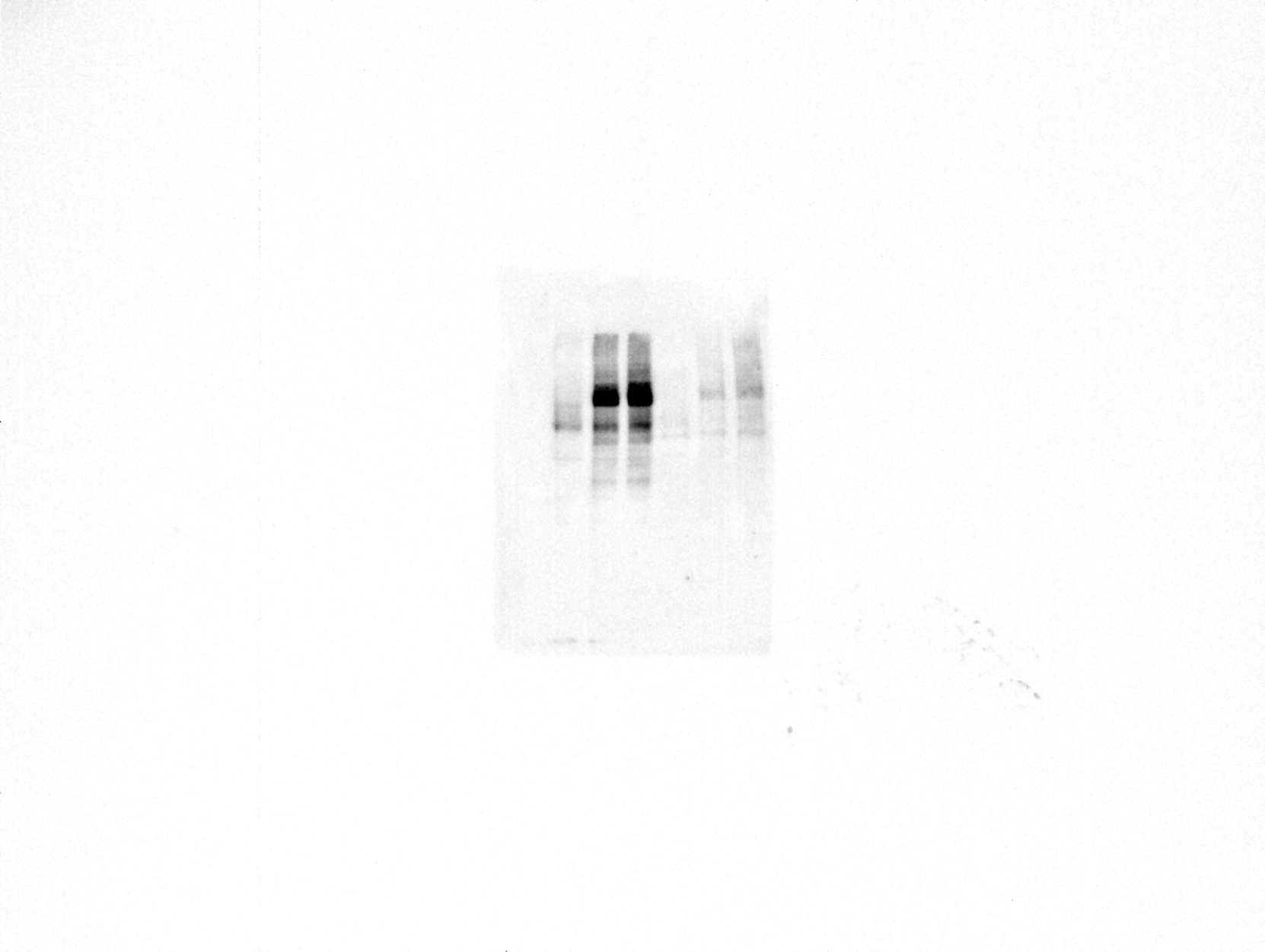

Supplement: Supplementary file 4 — Source data Fig. 3 [file 44318_2025_545_MOESM4_ESM.zip › Fig 3/3D/3D HeLa/ADPr/2024-0801-131127_pub.tif]

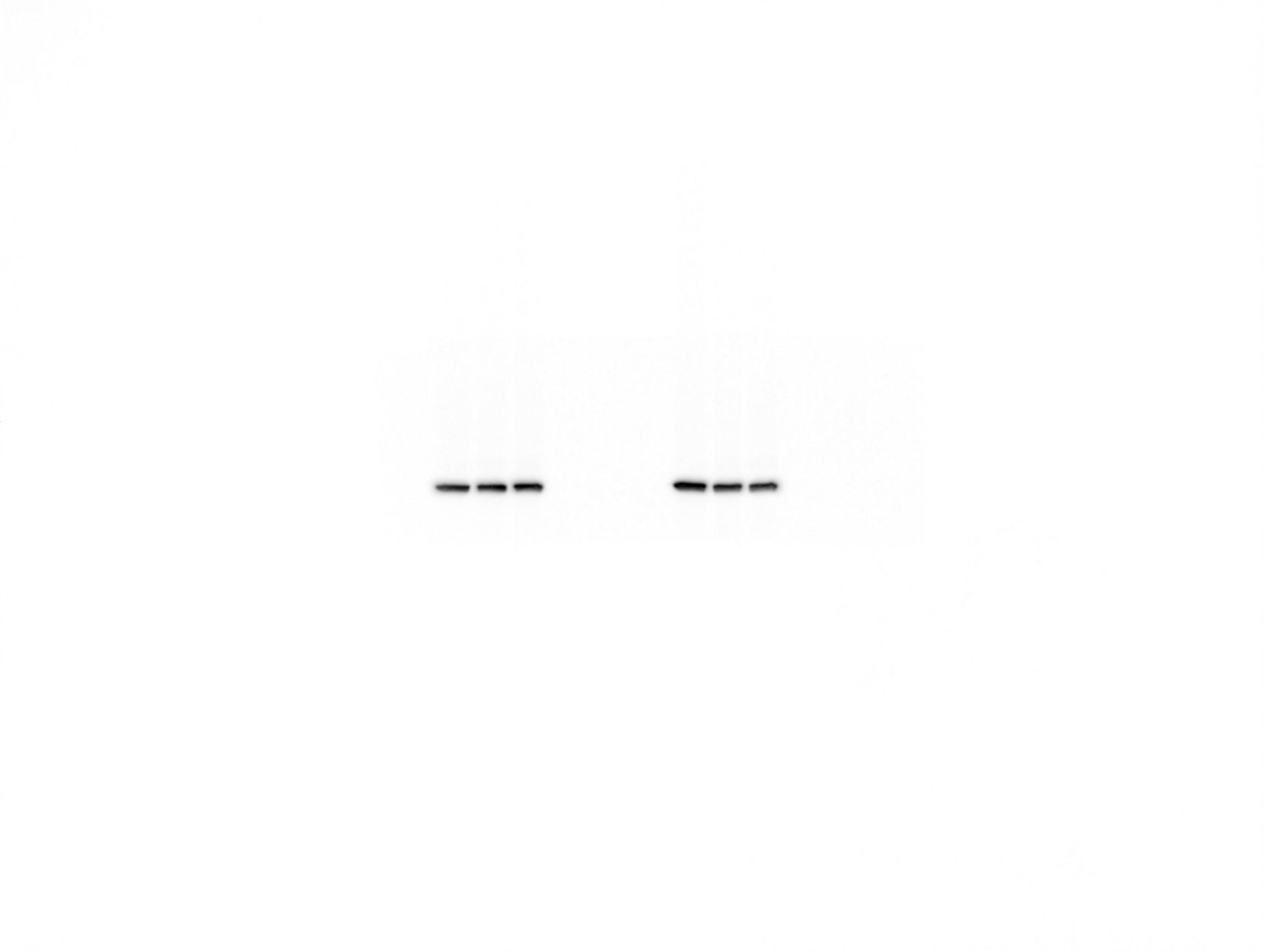

Supplement: Supplementary file 4 — Source data Fig. 3 [file 44318_2025_545_MOESM4_ESM.zip › Fig 3/3D/3D HeLa/Tub/2024-0801-132737_pub.tif]

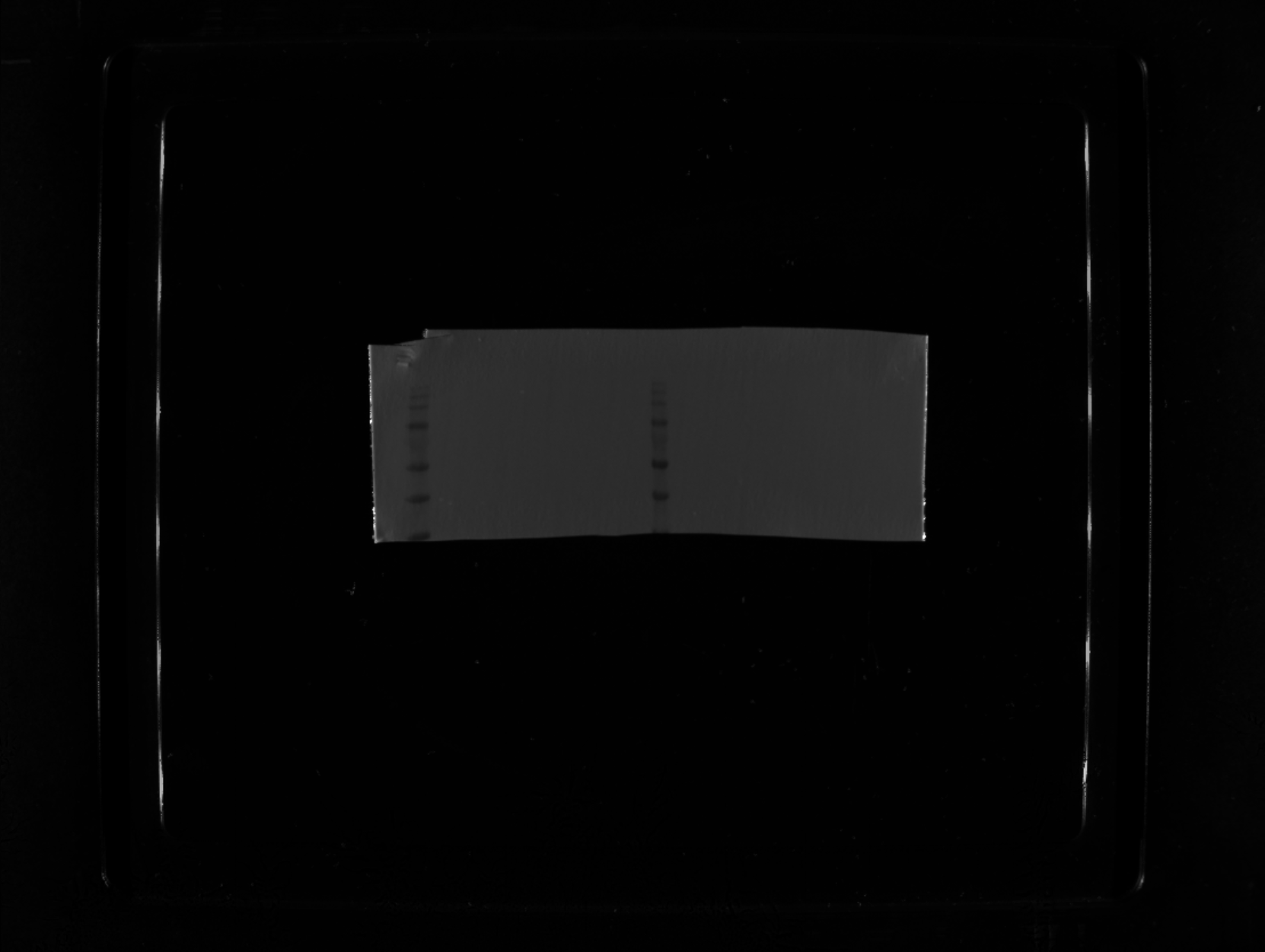

Supplement: Supplementary file 4 — Source data Fig. 3 [file 44318_2025_545_MOESM4_ESM.zip › Fig 3/3D/3D HeLa/Tub/2024-0801-132735_pub.tif]

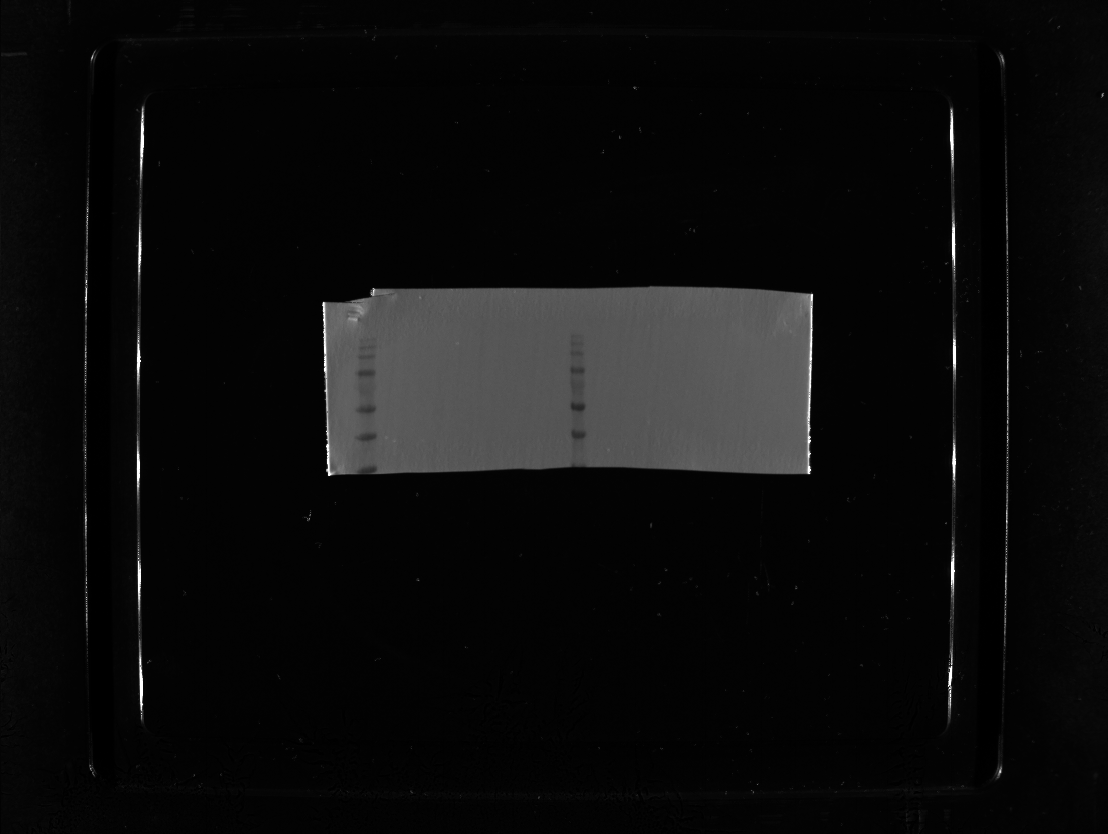

Supplement: Supplementary file 4 — Source data Fig. 3 [file 44318_2025_545_MOESM4_ESM.zip › Fig 3/3D/3D HeLa/Tub/2024-0801-132735.tif]

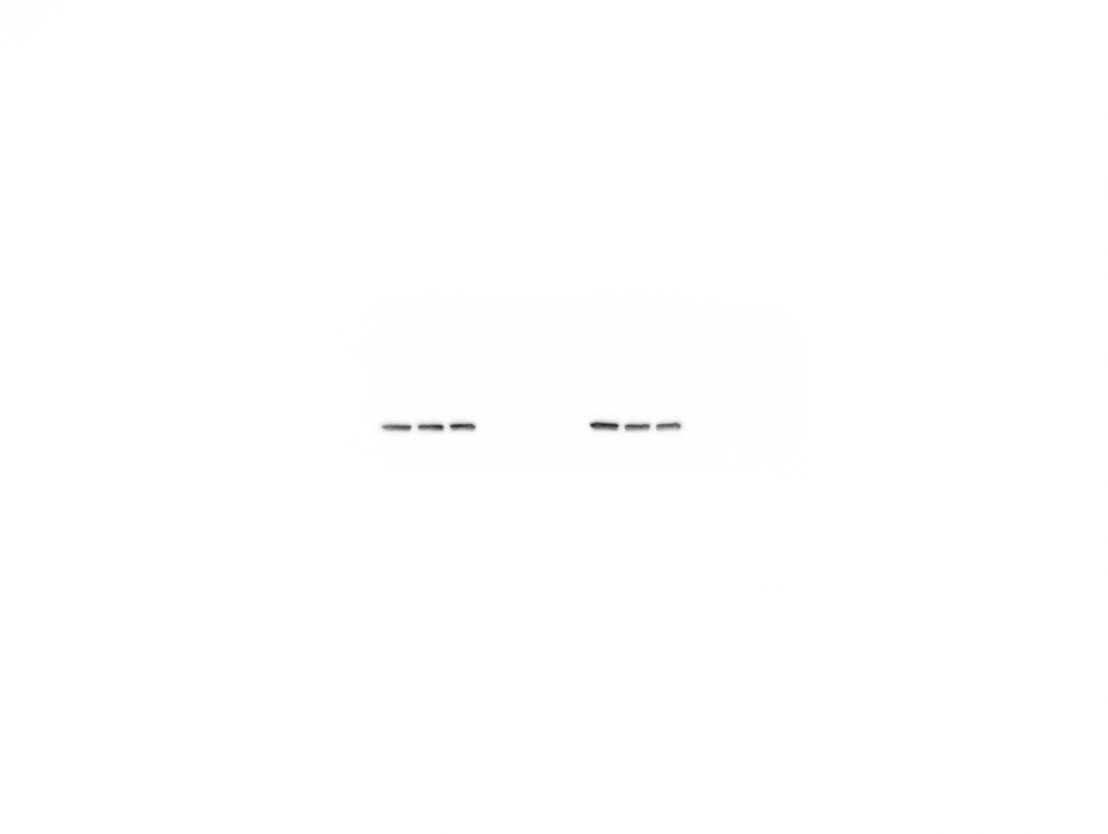

Supplement: Supplementary file 4 — Source data Fig. 3 [file 44318_2025_545_MOESM4_ESM.zip › Fig 3/3D/3D HeLa/Tub/2024-0801-132737.tif]

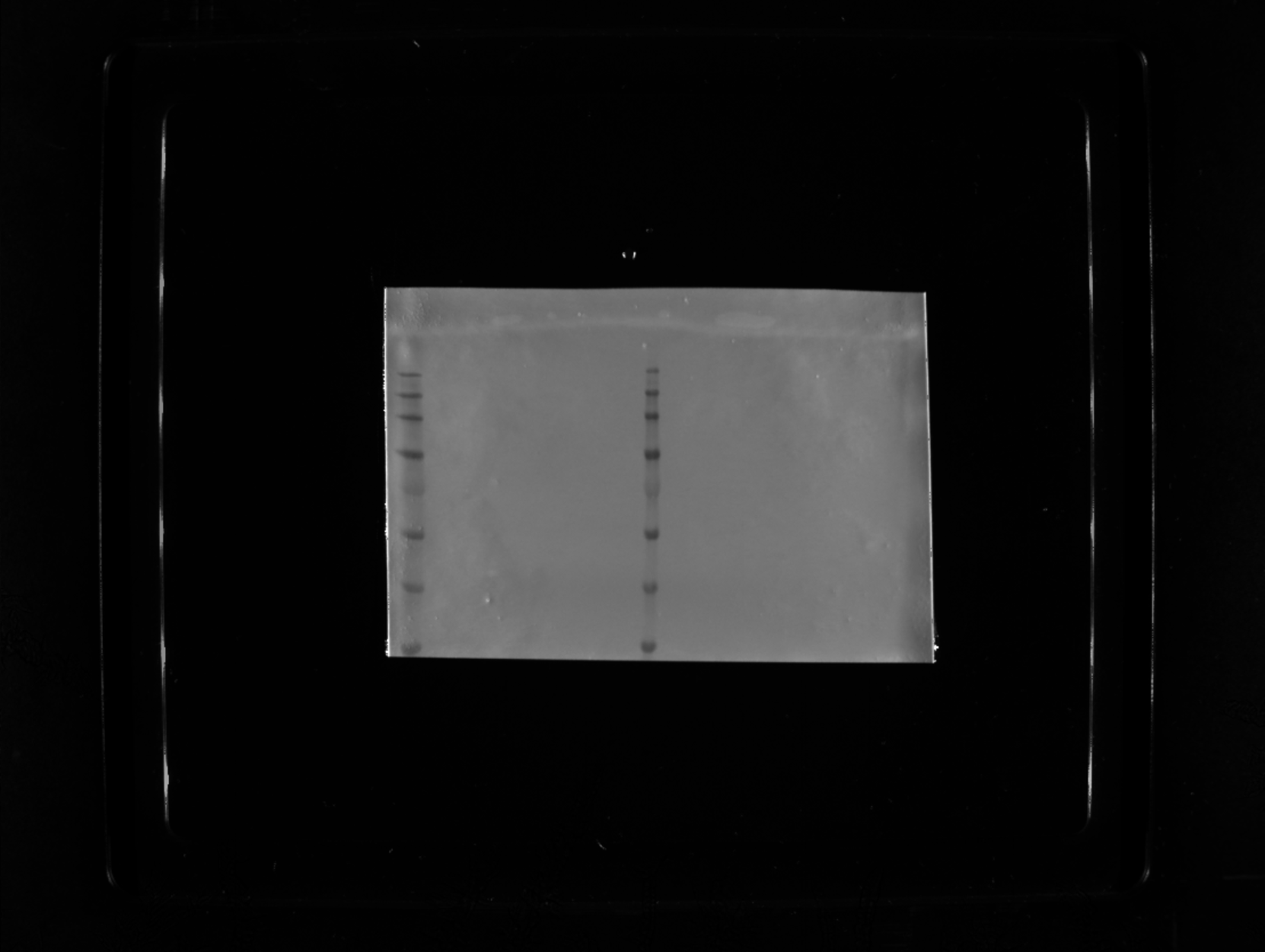

Supplement: Supplementary file 5 — Source data Fig. 4 [file 44318_2025_545_MOESM5_ESM.zip › Fig 4/4B/ADPr/2024-0626-101109_pub.tif]

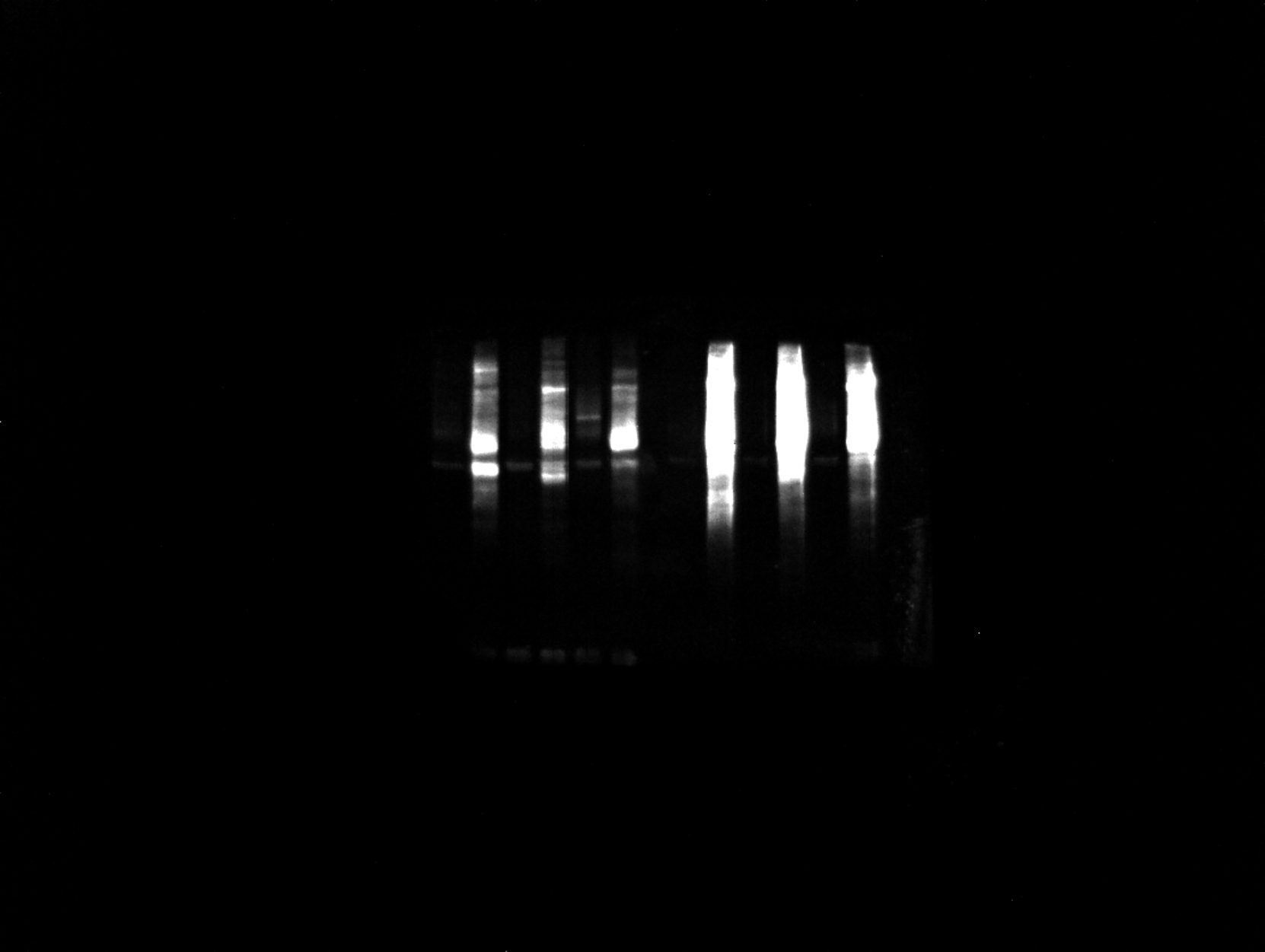

Supplement: Supplementary file 5 — Source data Fig. 4 [file 44318_2025_545_MOESM5_ESM.zip › Fig 4/4B/ADPr/2024-0626-101110_pub.tif]
