# Supplementary figures and images for: PARP7 is a proteotoxic stress sensor that labels proteins for degradation (part 2 of 2)
Source: EMBO J. 2025 Aug 20;44(19):5463–81. doi: 10.1038/s44318-025-00545-7 (PMC12488922; doi:10.1038/s44318-025-00545-7)

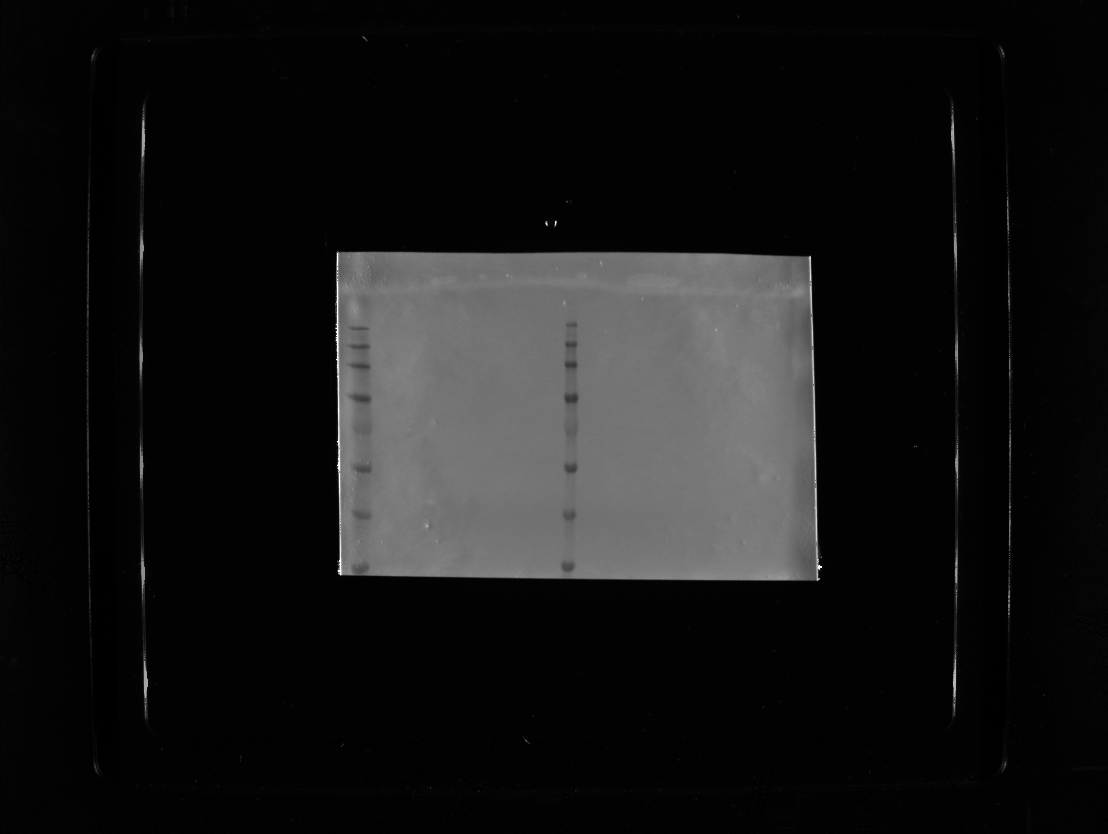

Supplement: Supplementary file 5 — Source data Fig. 4 [file 44318_2025_545_MOESM5_ESM.zip › Fig 4/4B/ADPr/2024-0626-101109.tif]

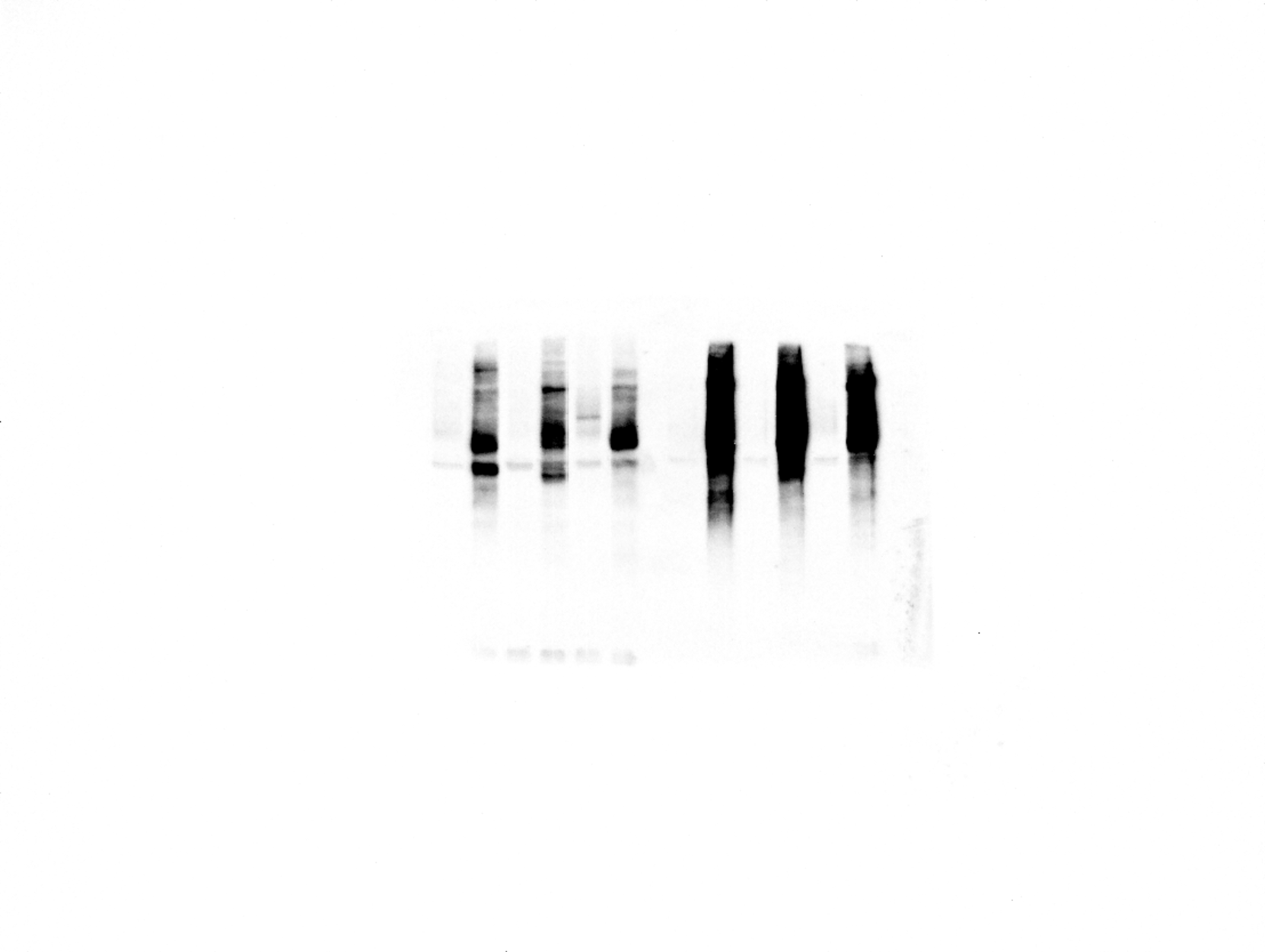

Supplement: Supplementary file 5 — Source data Fig. 4 [file 44318_2025_545_MOESM5_ESM.zip › Fig 4/4B/ADPr/2024-0626-101110_pub-1.tif]

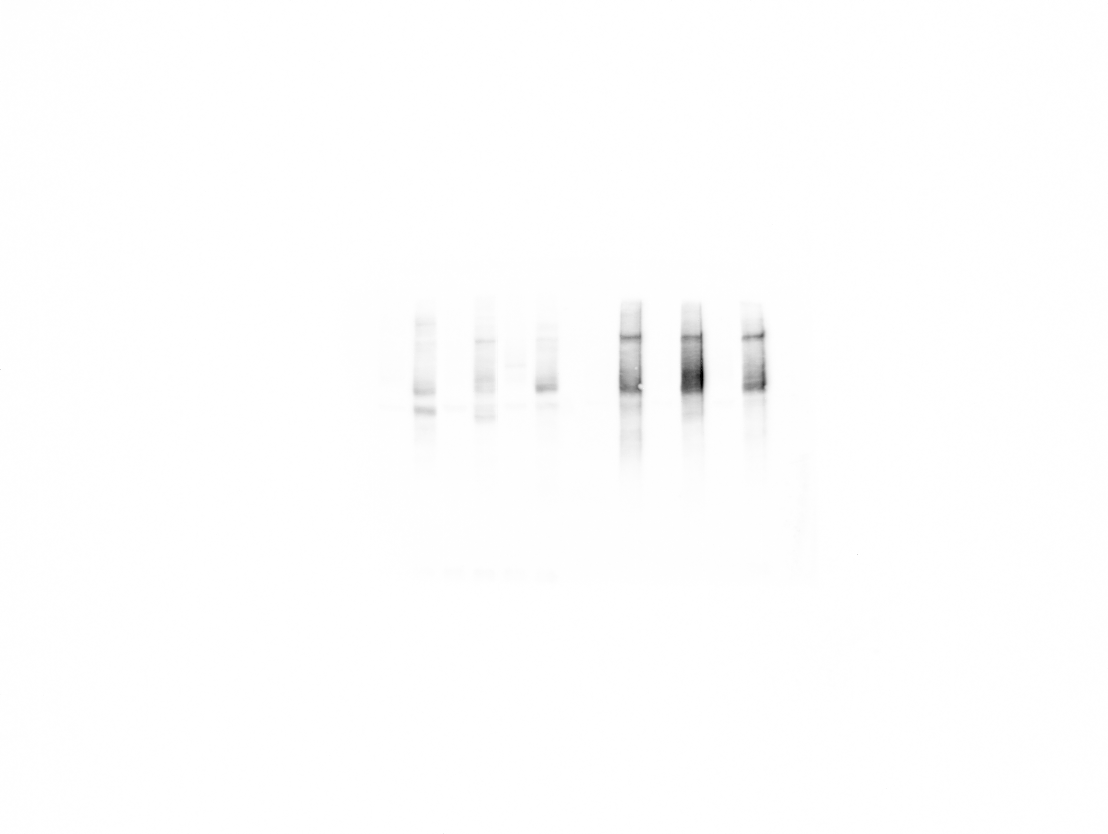

Supplement: Supplementary file 5 — Source data Fig. 4 [file 44318_2025_545_MOESM5_ESM.zip › Fig 4/4B/ADPr/2024-0626-101110.tif]

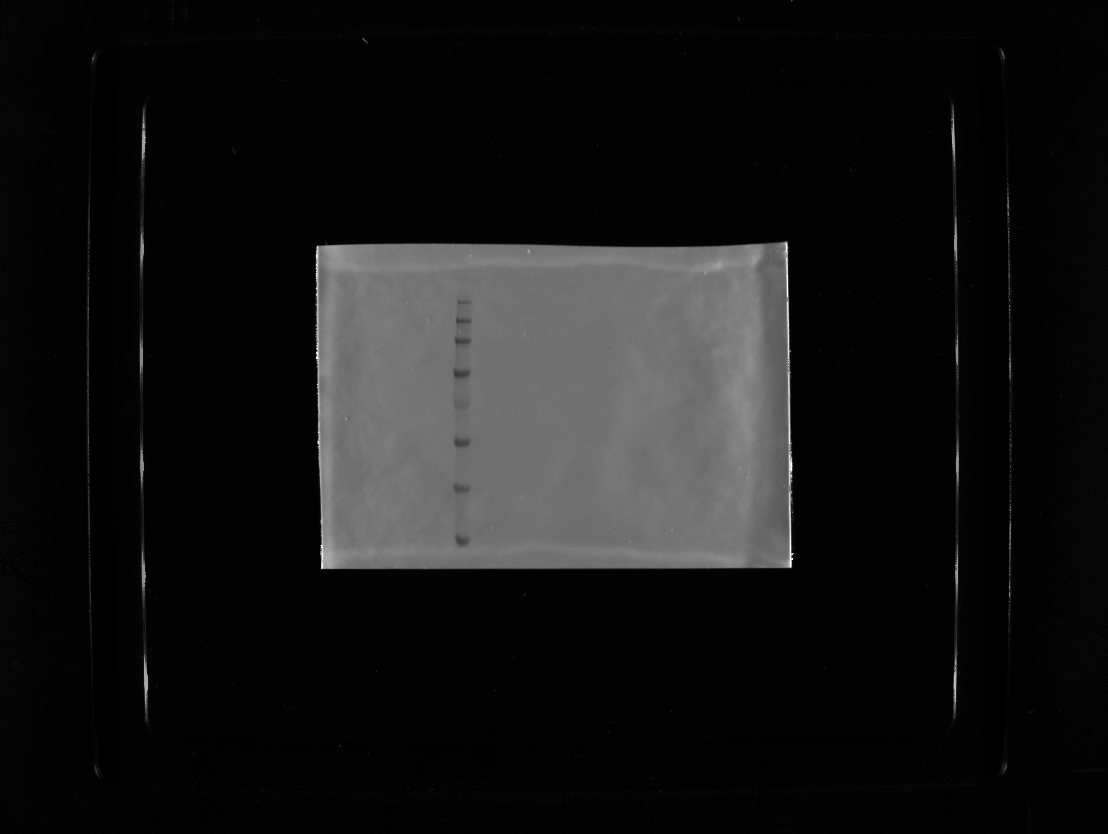

Supplement: Supplementary file 5 — Source data Fig. 4 [file 44318_2025_545_MOESM5_ESM.zip › Fig 4/4B/Ub/2024-0626-101557.tif]

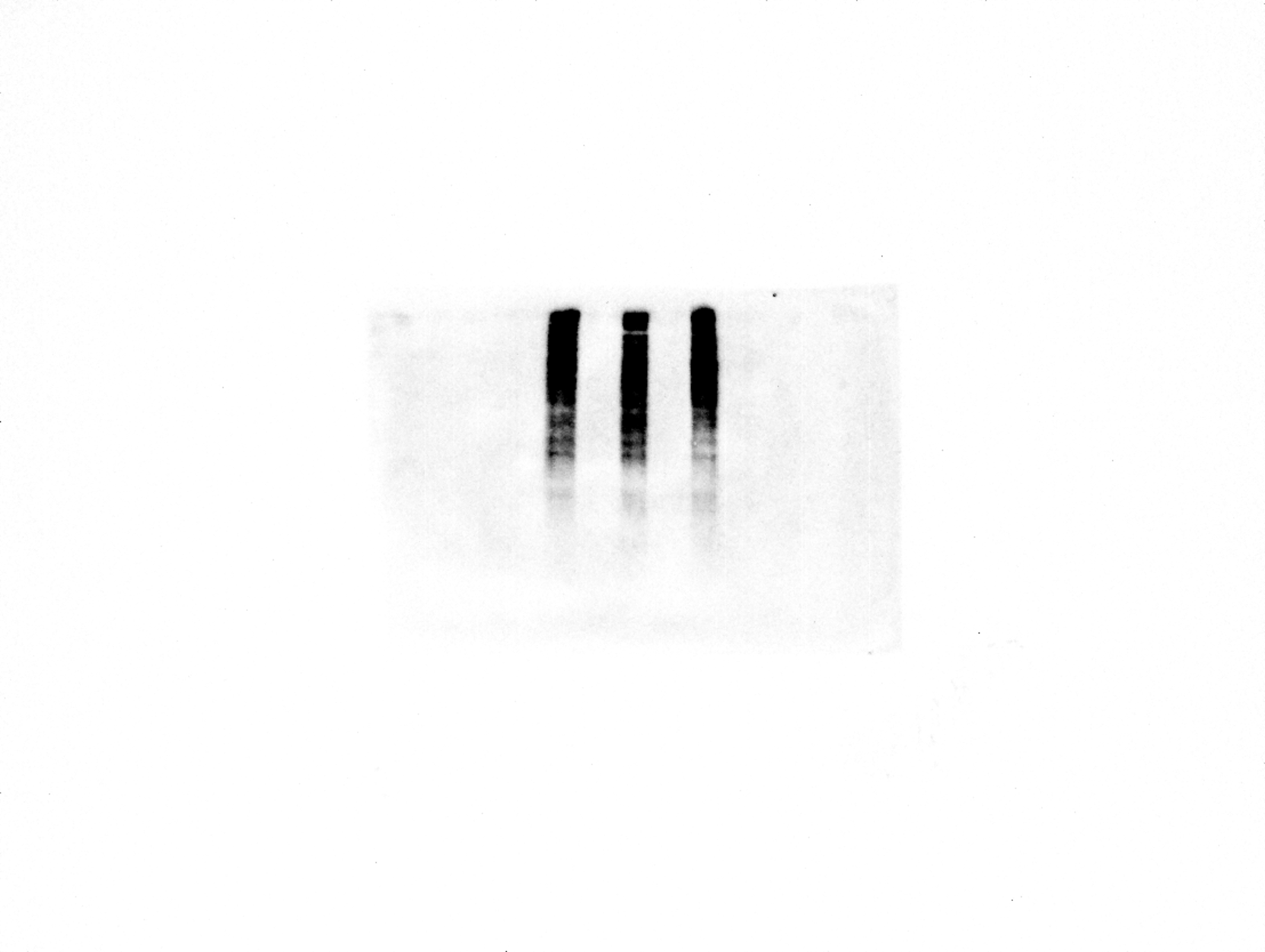

Supplement: Supplementary file 5 — Source data Fig. 4 [file 44318_2025_545_MOESM5_ESM.zip › Fig 4/4B/Ub/2024-0626-101559_pub.tif]

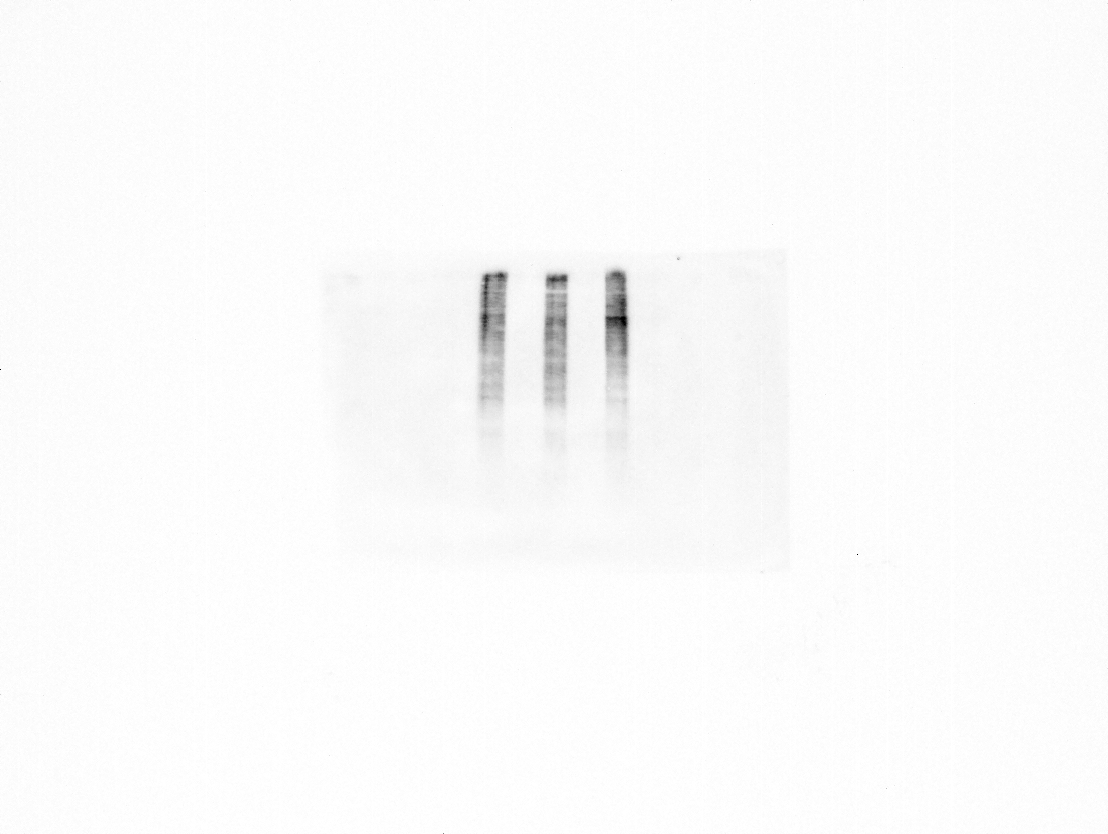

Supplement: Supplementary file 5 — Source data Fig. 4 [file 44318_2025_545_MOESM5_ESM.zip › Fig 4/4B/Ub/2024-0626-101559.tif]

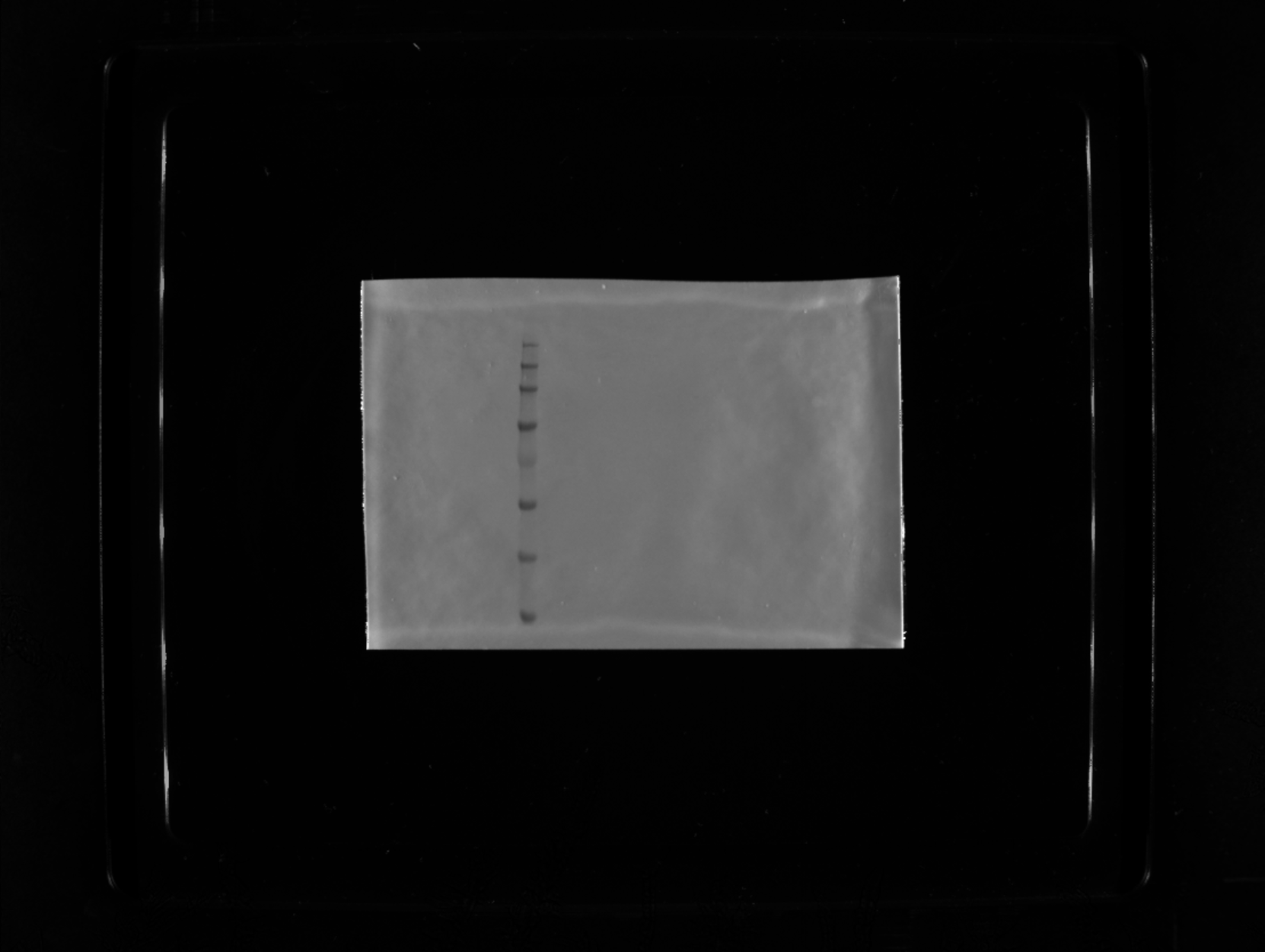

Supplement: Supplementary file 5 — Source data Fig. 4 [file 44318_2025_545_MOESM5_ESM.zip › Fig 4/4B/Ub/2024-0626-101557_pub.tif]

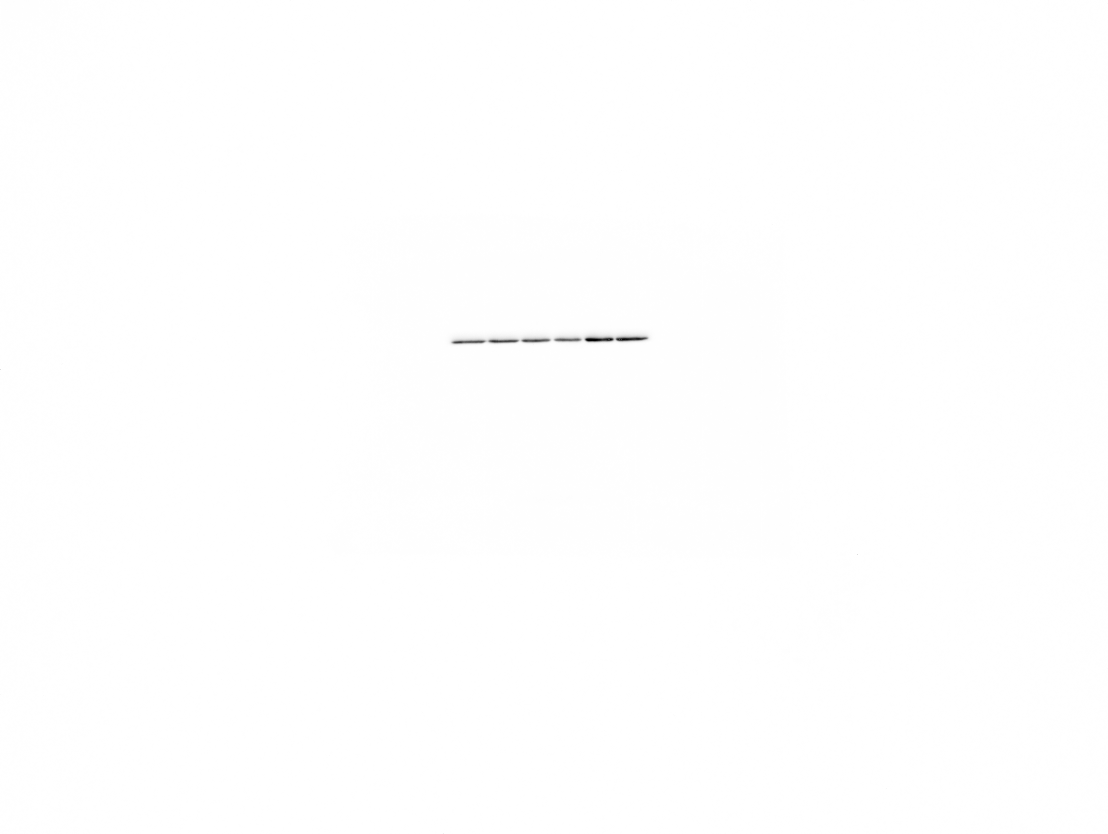

Supplement: Supplementary file 5 — Source data Fig. 4 [file 44318_2025_545_MOESM5_ESM.zip › Fig 4/4B/Tub/2024-0626-102147.tif]

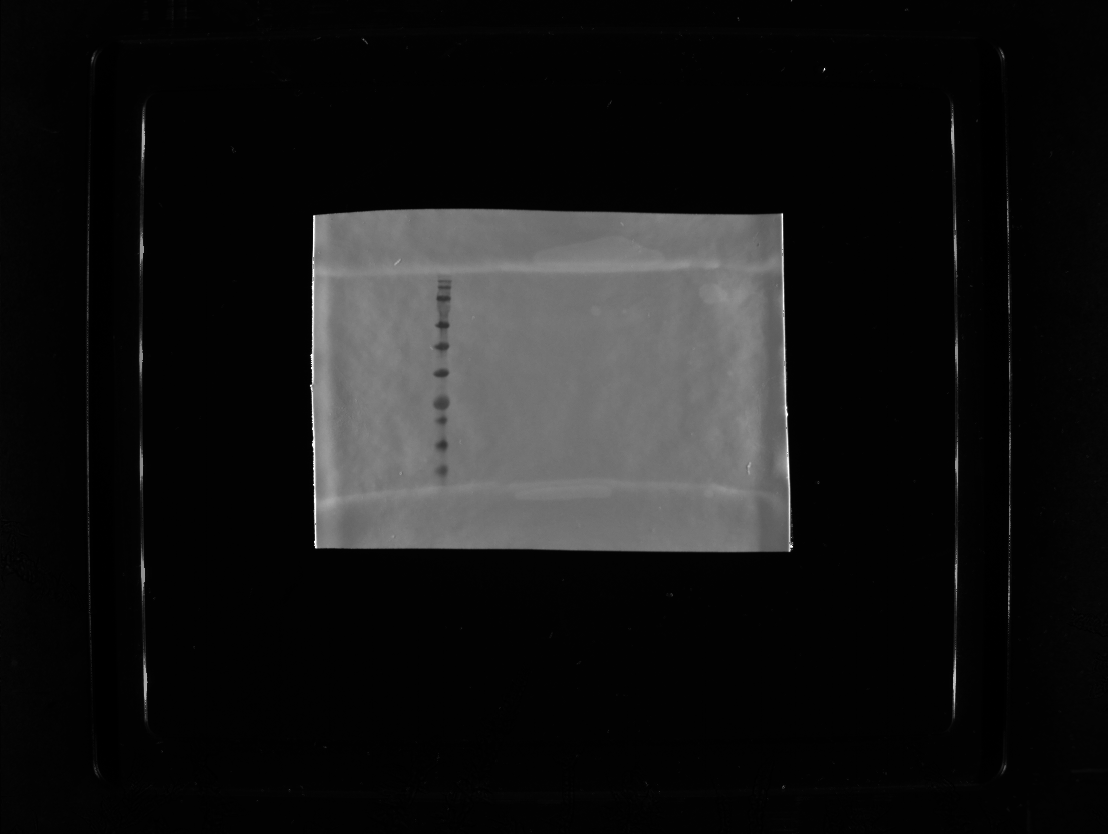

Supplement: Supplementary file 5 — Source data Fig. 4 [file 44318_2025_545_MOESM5_ESM.zip › Fig 4/4B/Tub/2024-0626-102146.tif]

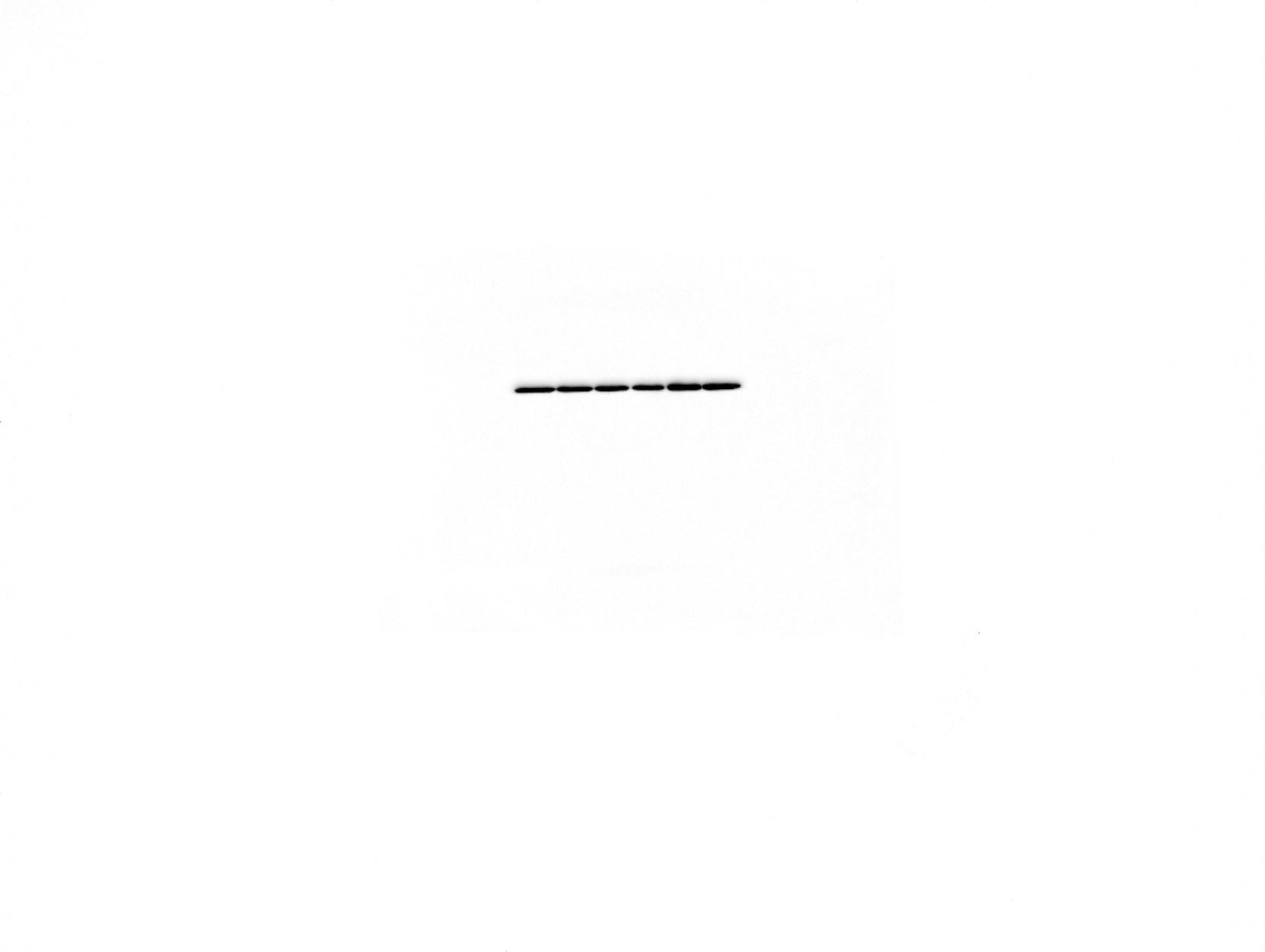

Supplement: Supplementary file 5 — Source data Fig. 4 [file 44318_2025_545_MOESM5_ESM.zip › Fig 4/4B/Tub/2024-0626-102147_pub.tif]

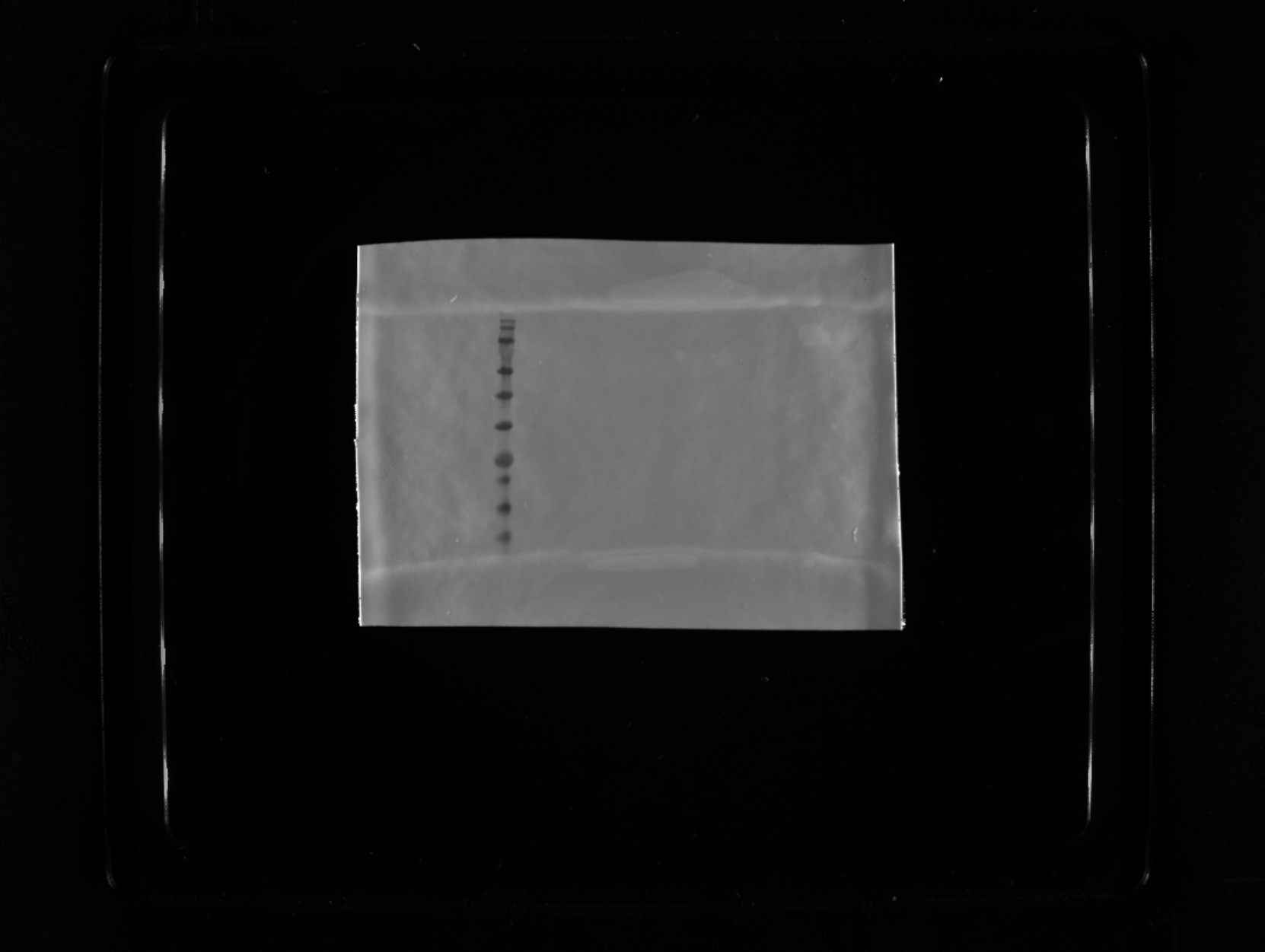

Supplement: Supplementary file 5 — Source data Fig. 4 [file 44318_2025_545_MOESM5_ESM.zip › Fig 4/4B/Tub/2024-0626-102146_pub.tif]

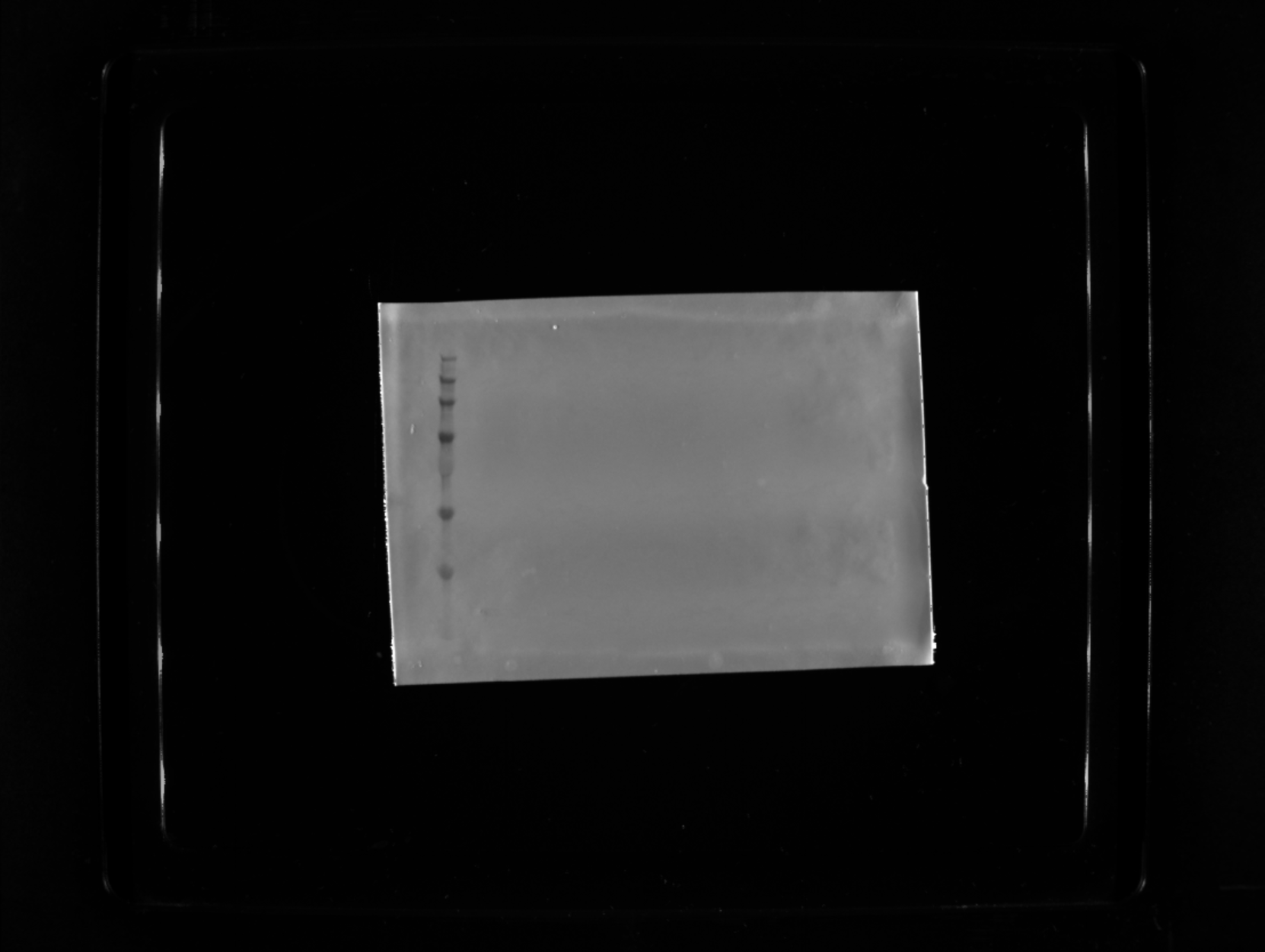

Supplement: Supplementary file 5 — Source data Fig. 4 [file 44318_2025_545_MOESM5_ESM.zip › Fig 4/4C/Soluble ADPr/2024-0614-165119_pub.tif]

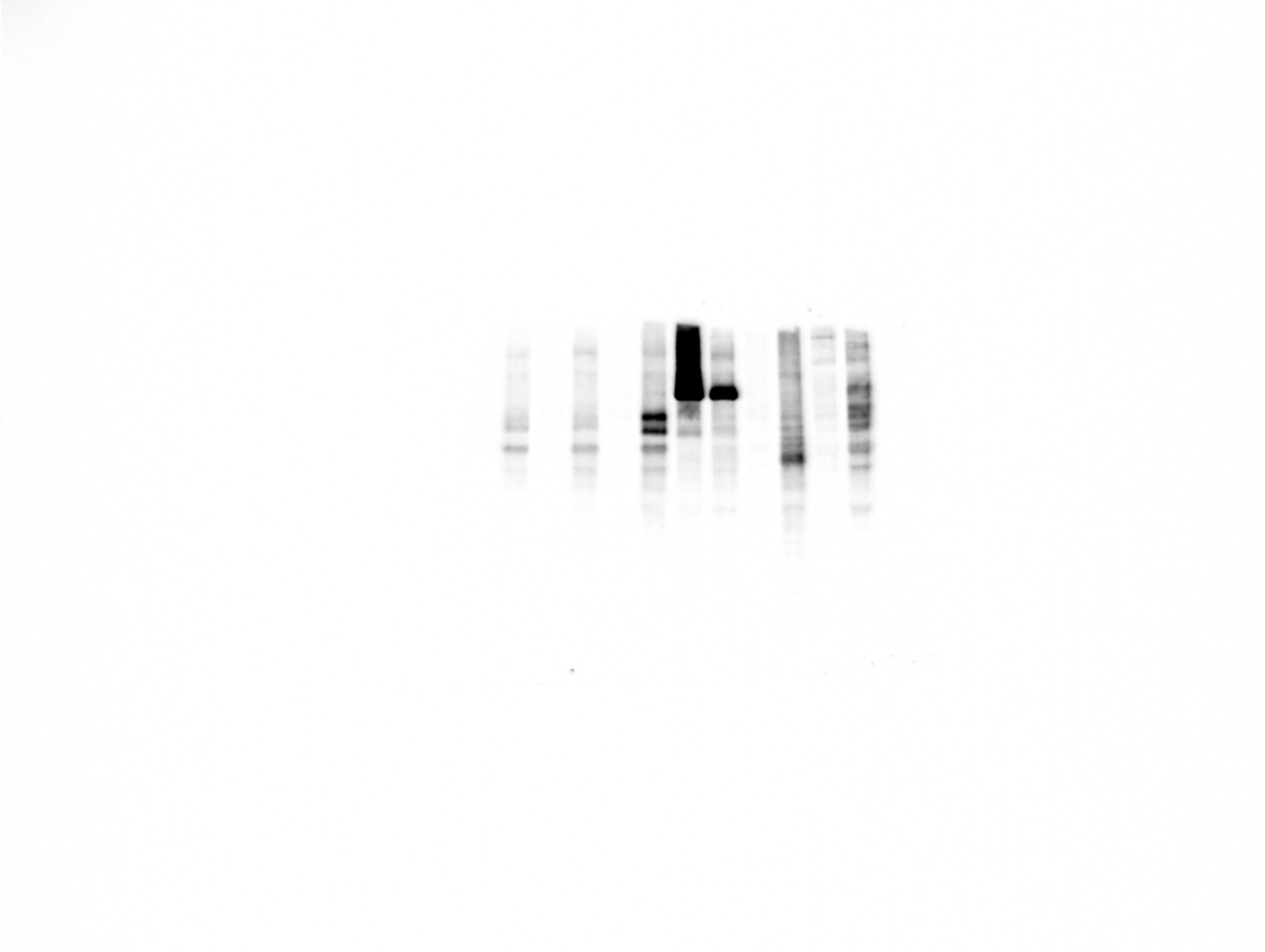

Supplement: Supplementary file 5 — Source data Fig. 4 [file 44318_2025_545_MOESM5_ESM.zip › Fig 4/4C/Soluble ADPr/2024-0614-165120_pub.tif]

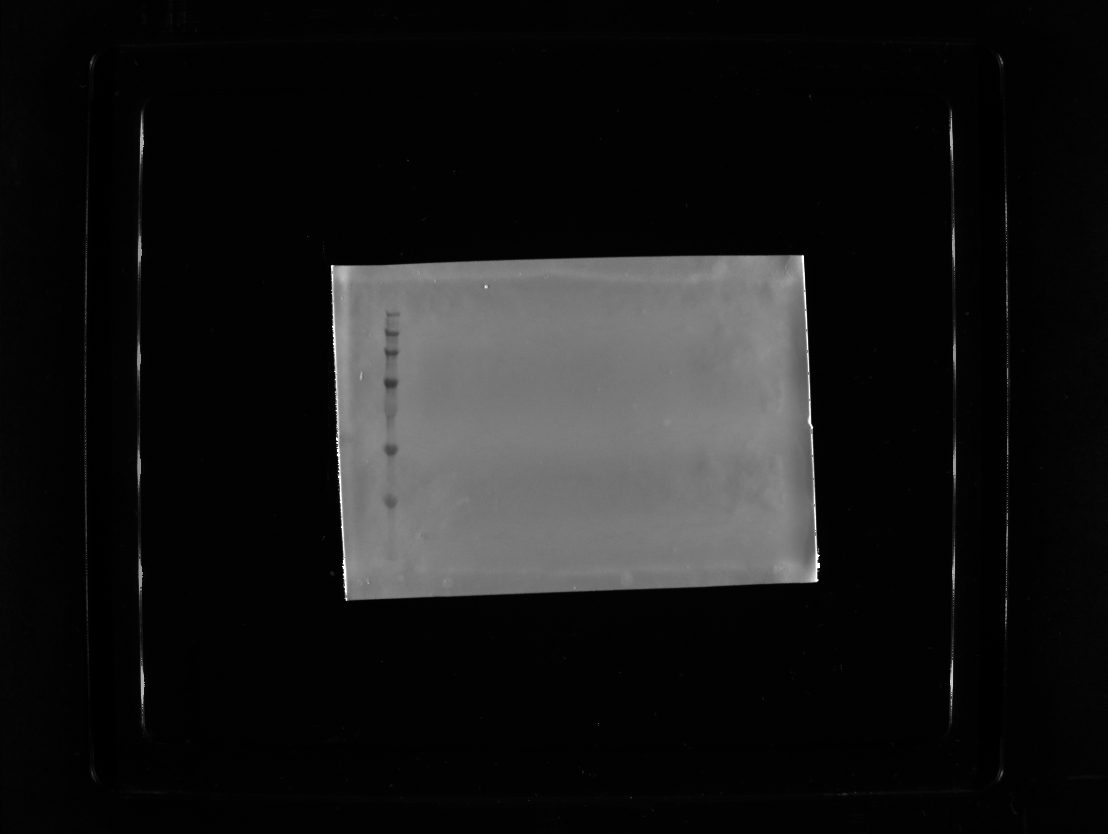

Supplement: Supplementary file 5 — Source data Fig. 4 [file 44318_2025_545_MOESM5_ESM.zip › Fig 4/4C/Soluble ADPr/2024-0614-165119.tif]

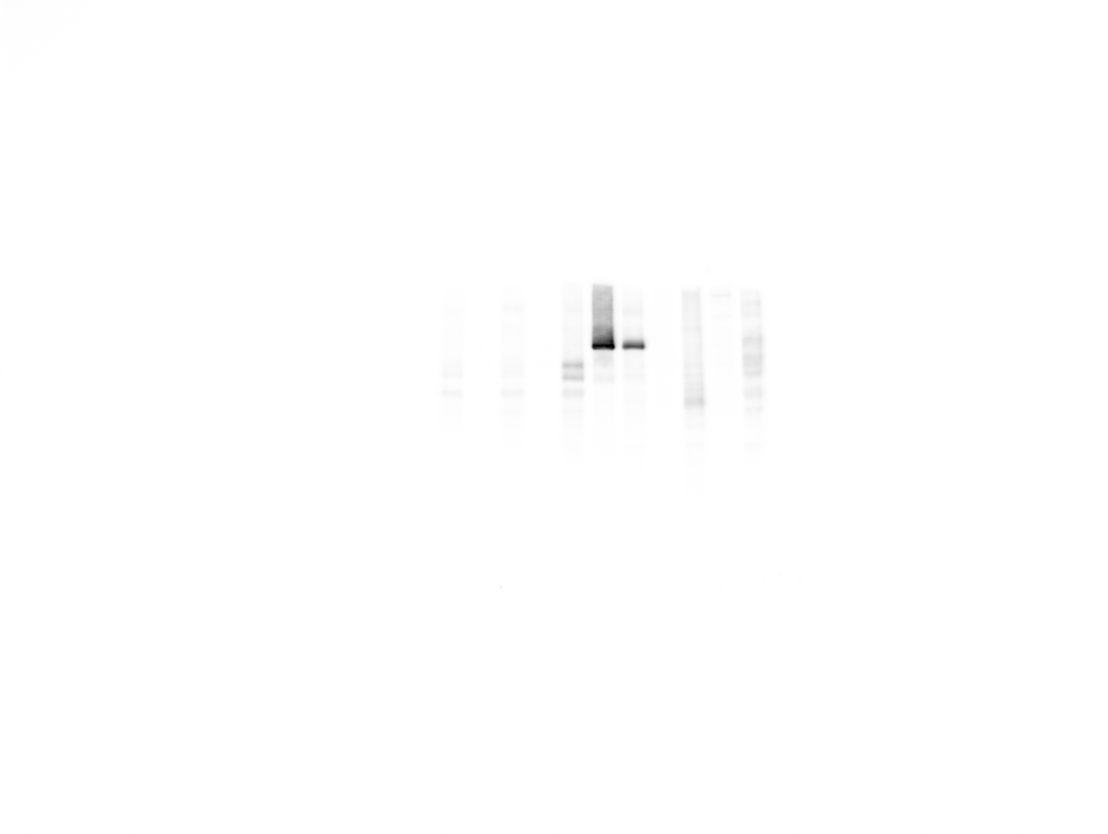

Supplement: Supplementary file 5 — Source data Fig. 4 [file 44318_2025_545_MOESM5_ESM.zip › Fig 4/4C/Soluble ADPr/2024-0614-165120.tif]

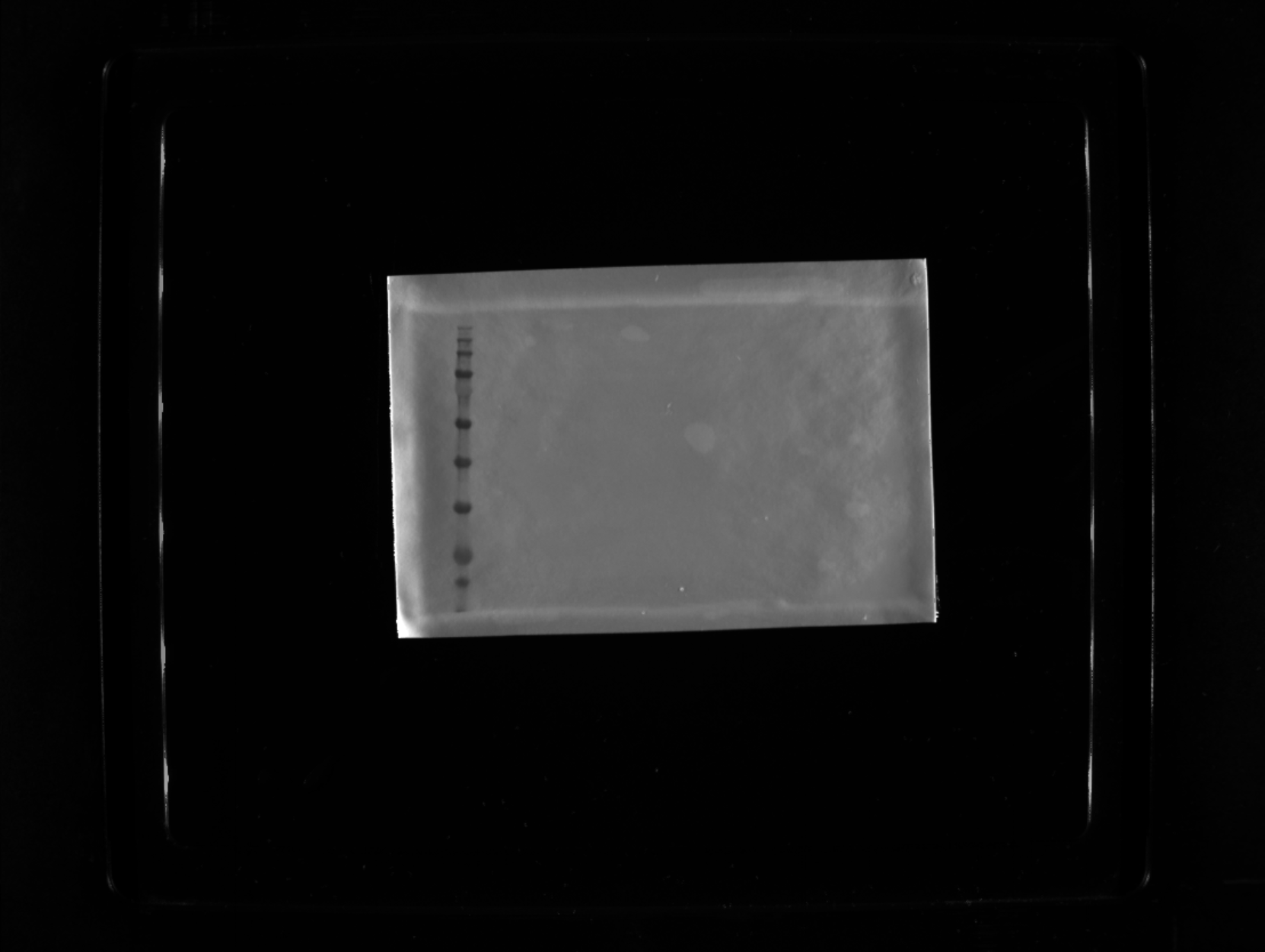

Supplement: Supplementary file 5 — Source data Fig. 4 [file 44318_2025_545_MOESM5_ESM.zip › Fig 4/4C/Soluble Tub/2024-0614-165940_pub.tif]

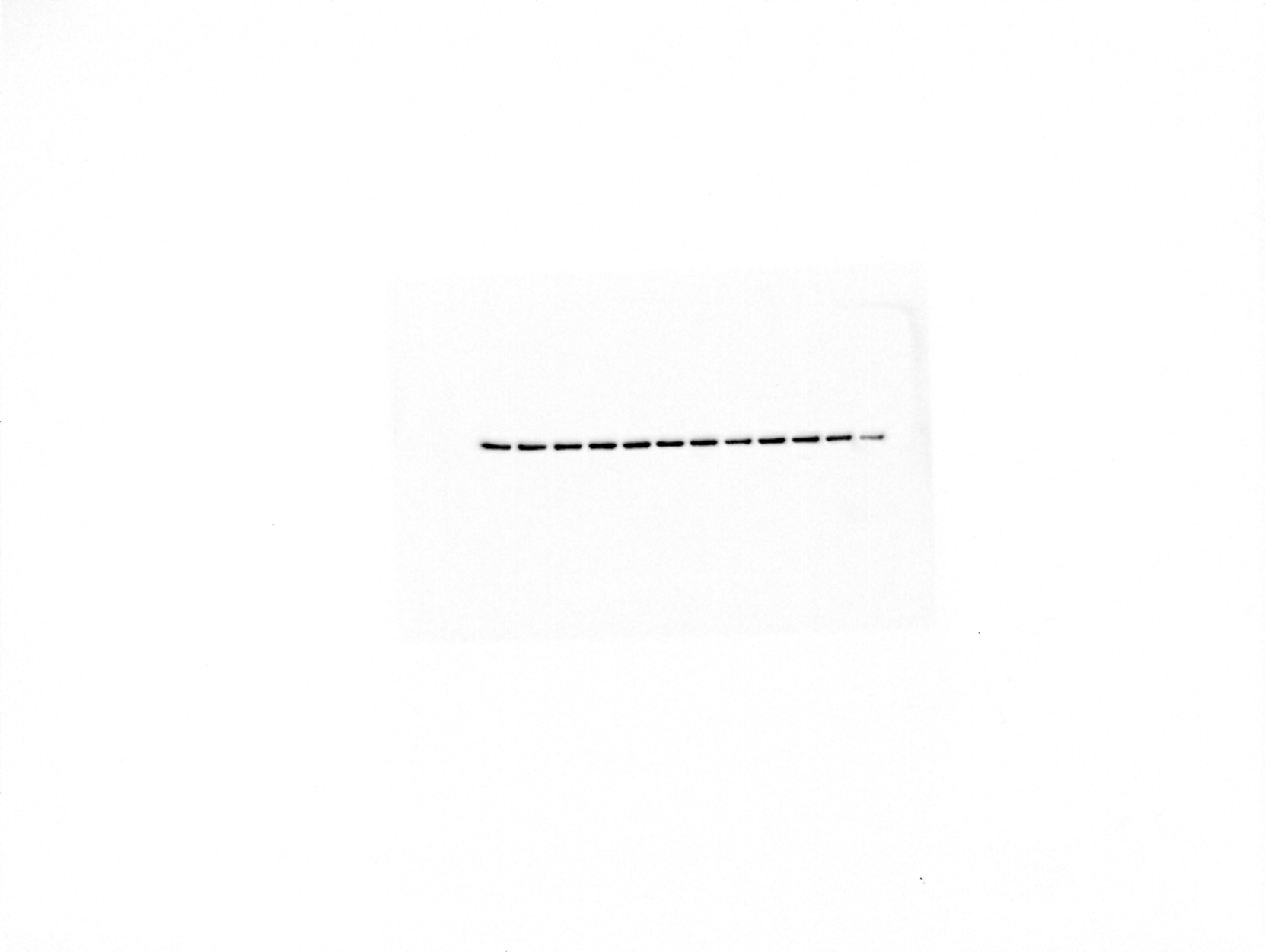

Supplement: Supplementary file 5 — Source data Fig. 4 [file 44318_2025_545_MOESM5_ESM.zip › Fig 4/4C/Soluble Tub/2024-0614-165941_pub.tif]

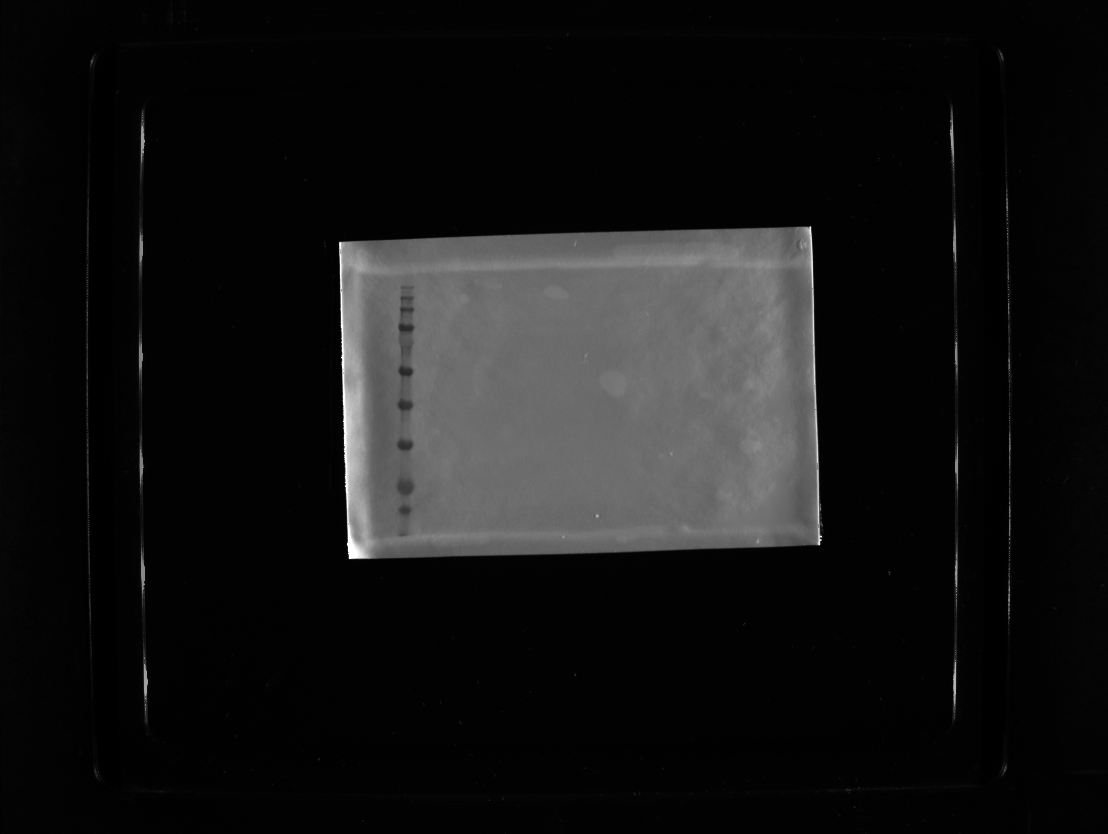

Supplement: Supplementary file 5 — Source data Fig. 4 [file 44318_2025_545_MOESM5_ESM.zip › Fig 4/4C/Soluble Tub/2024-0614-165940.tif]

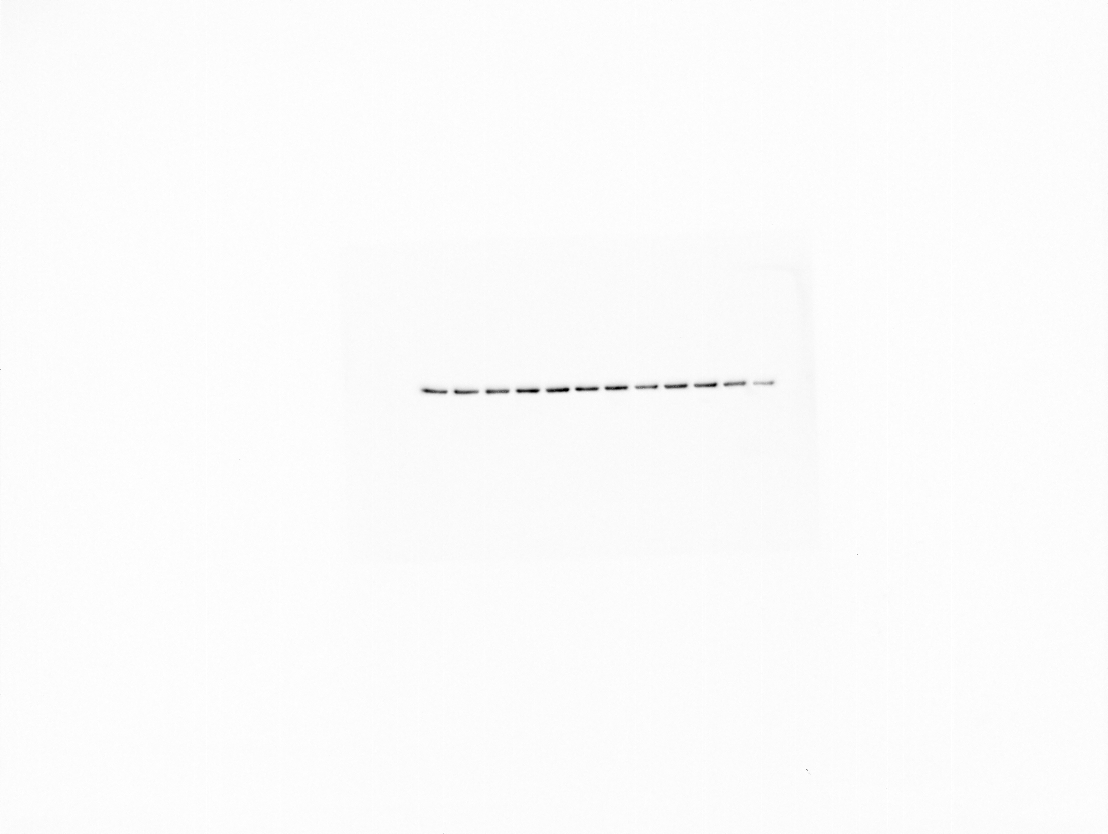

Supplement: Supplementary file 5 — Source data Fig. 4 [file 44318_2025_545_MOESM5_ESM.zip › Fig 4/4C/Soluble Tub/2024-0614-165941.tif]

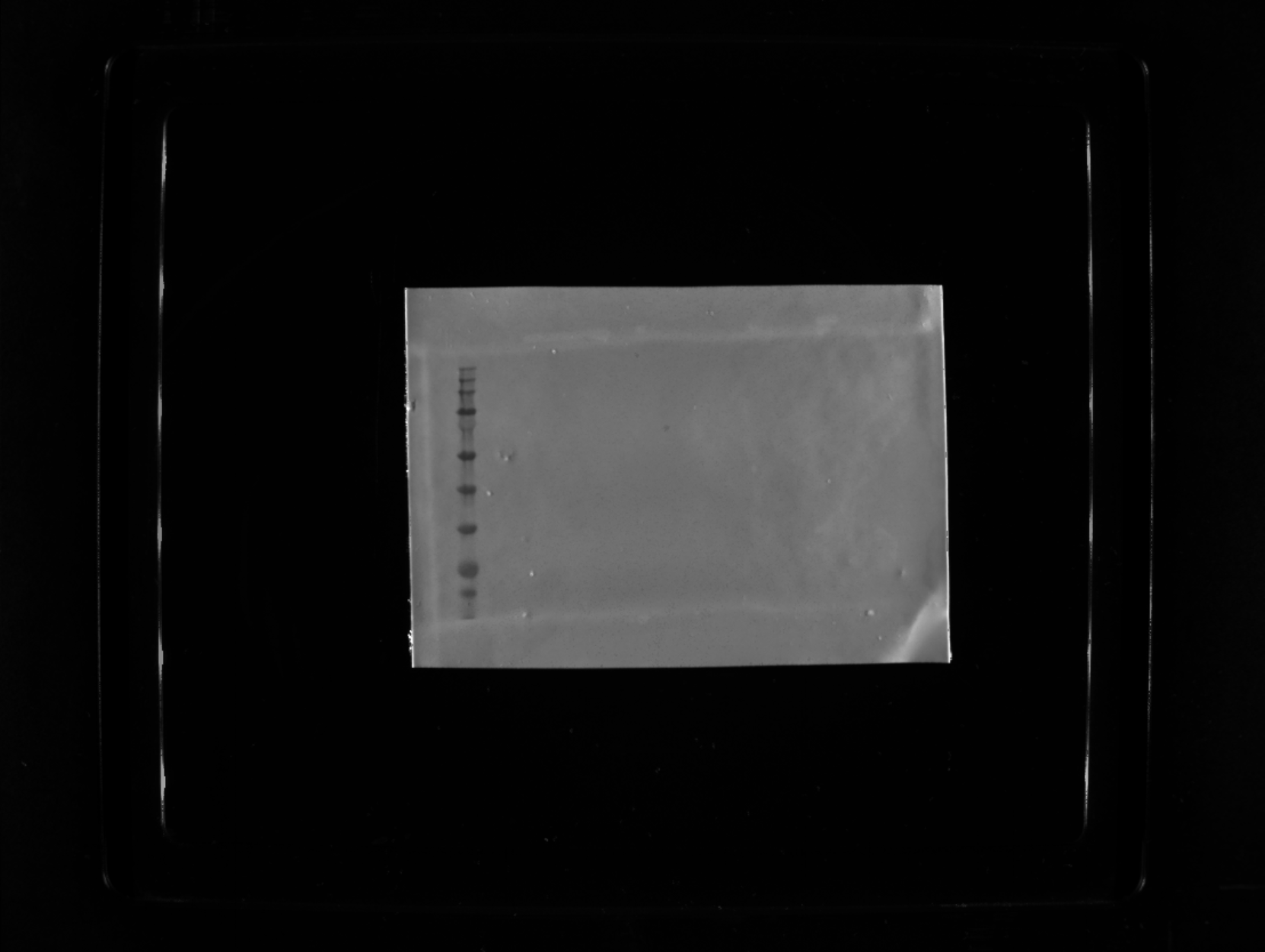

Supplement: Supplementary file 5 — Source data Fig. 4 [file 44318_2025_545_MOESM5_ESM.zip › Fig 4/4C/Soluble GFP/2024-0614-164730_pub.tif]

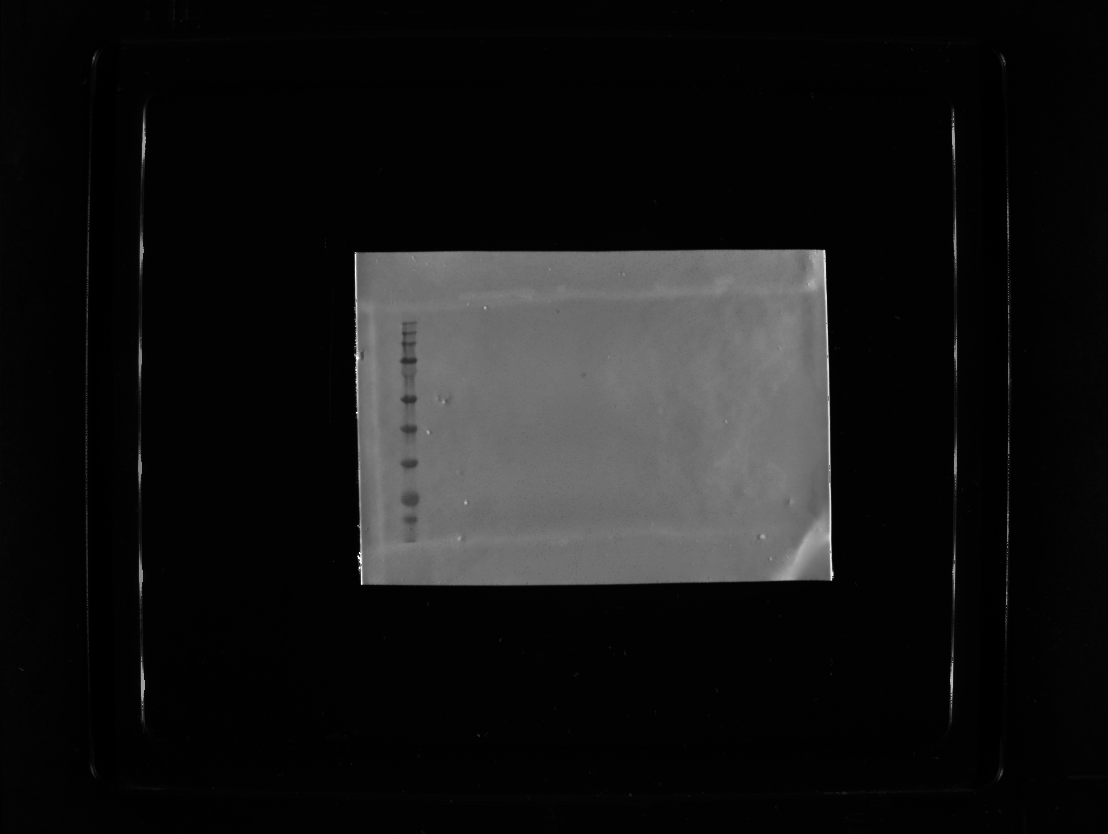

Supplement: Supplementary file 5 — Source data Fig. 4 [file 44318_2025_545_MOESM5_ESM.zip › Fig 4/4C/Soluble GFP/2024-0614-164730.tif]

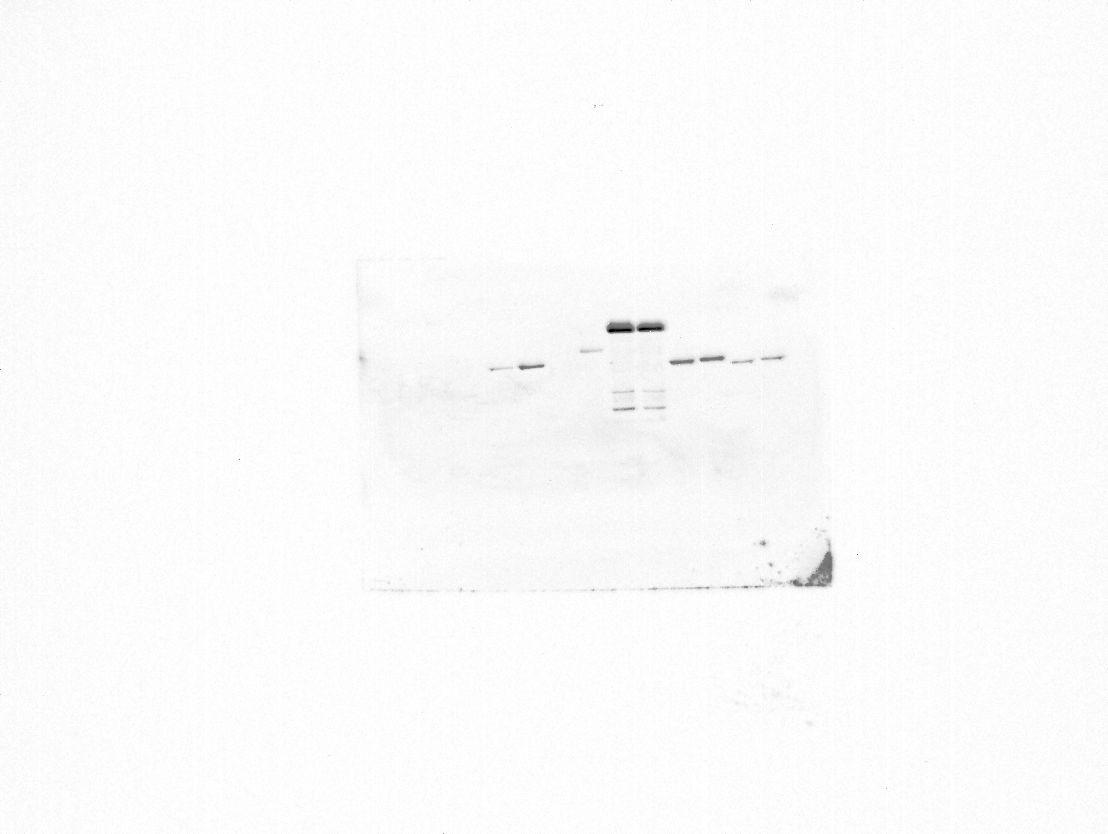

Supplement: Supplementary file 5 — Source data Fig. 4 [file 44318_2025_545_MOESM5_ESM.zip › Fig 4/4C/Soluble GFP/2024-0614-164732.tif]

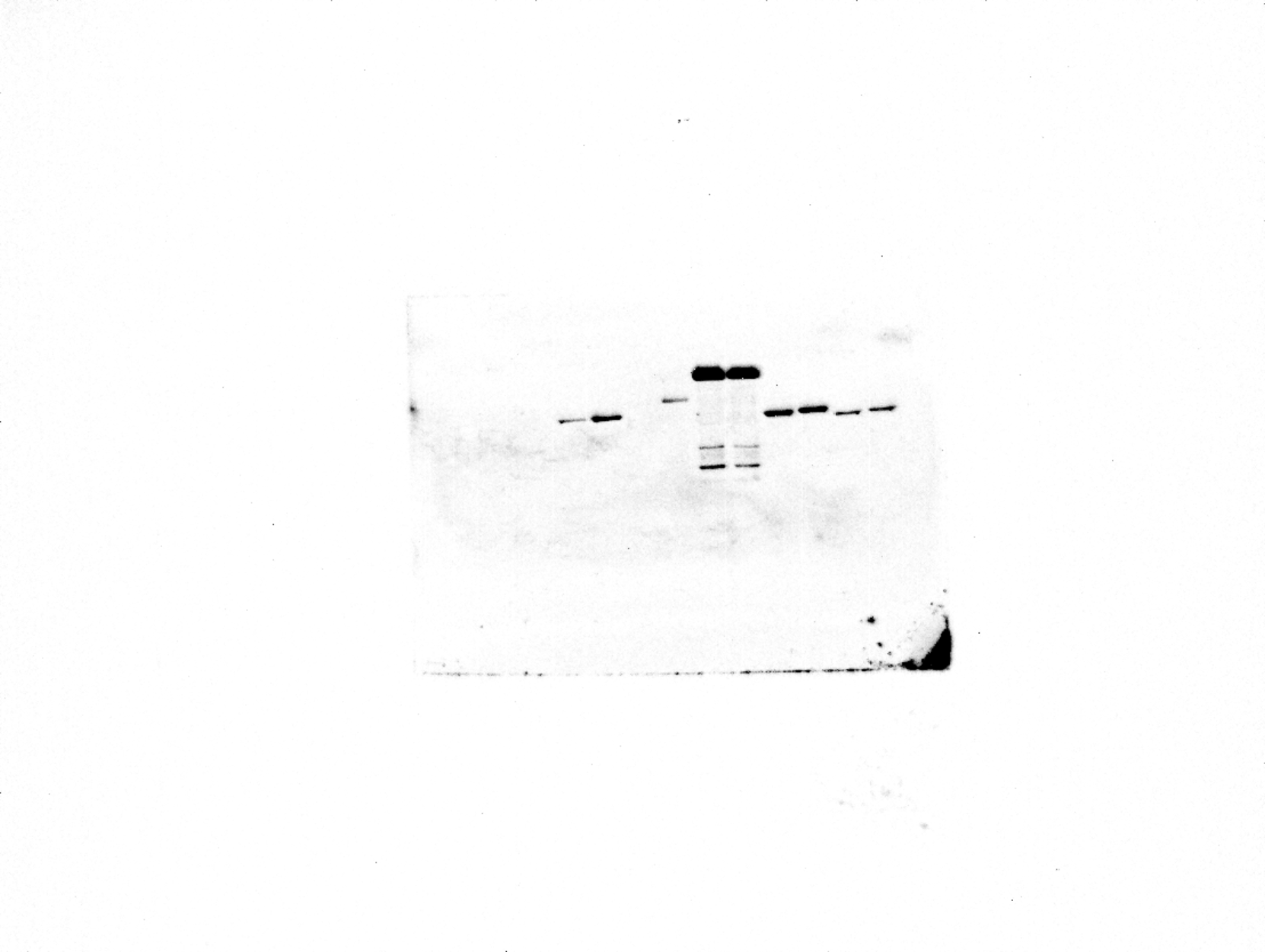

Supplement: Supplementary file 5 — Source data Fig. 4 [file 44318_2025_545_MOESM5_ESM.zip › Fig 4/4C/Soluble GFP/2024-0614-164732_pub.tif]

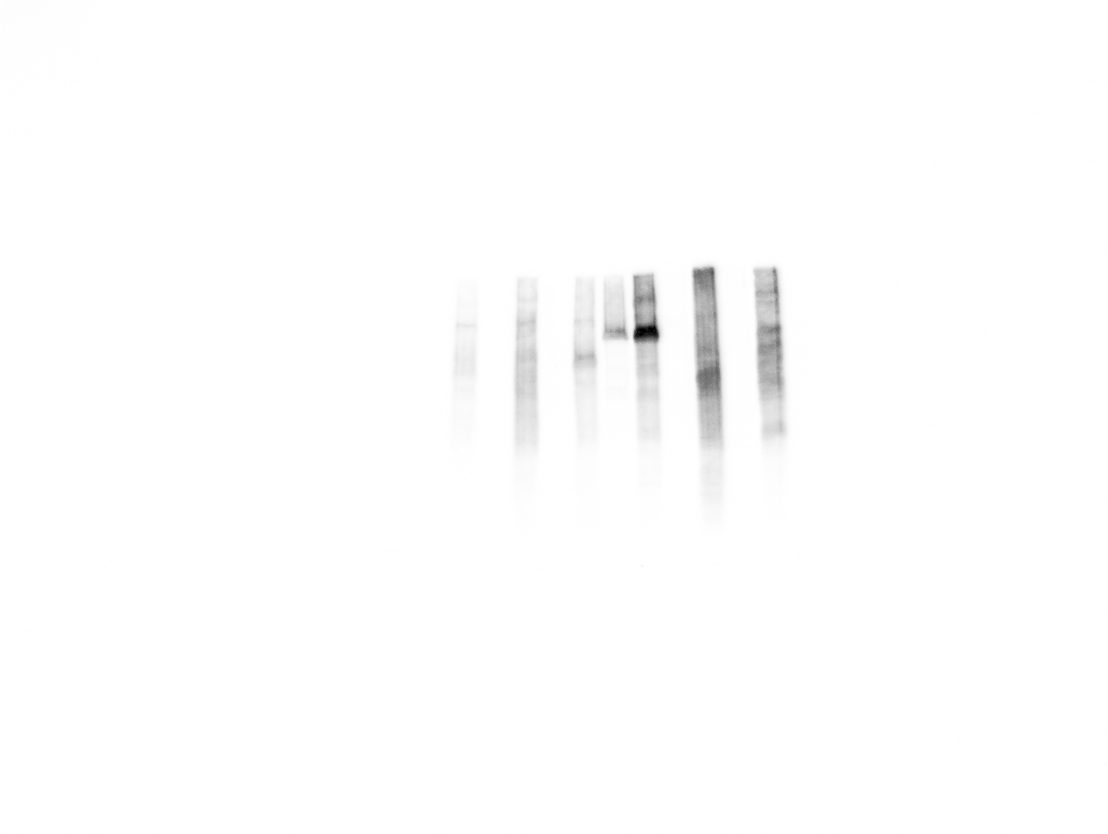

Supplement: Supplementary file 5 — Source data Fig. 4 [file 44318_2025_545_MOESM5_ESM.zip › Fig 4/4C/P ADPr/2024-0614-164133.tif]

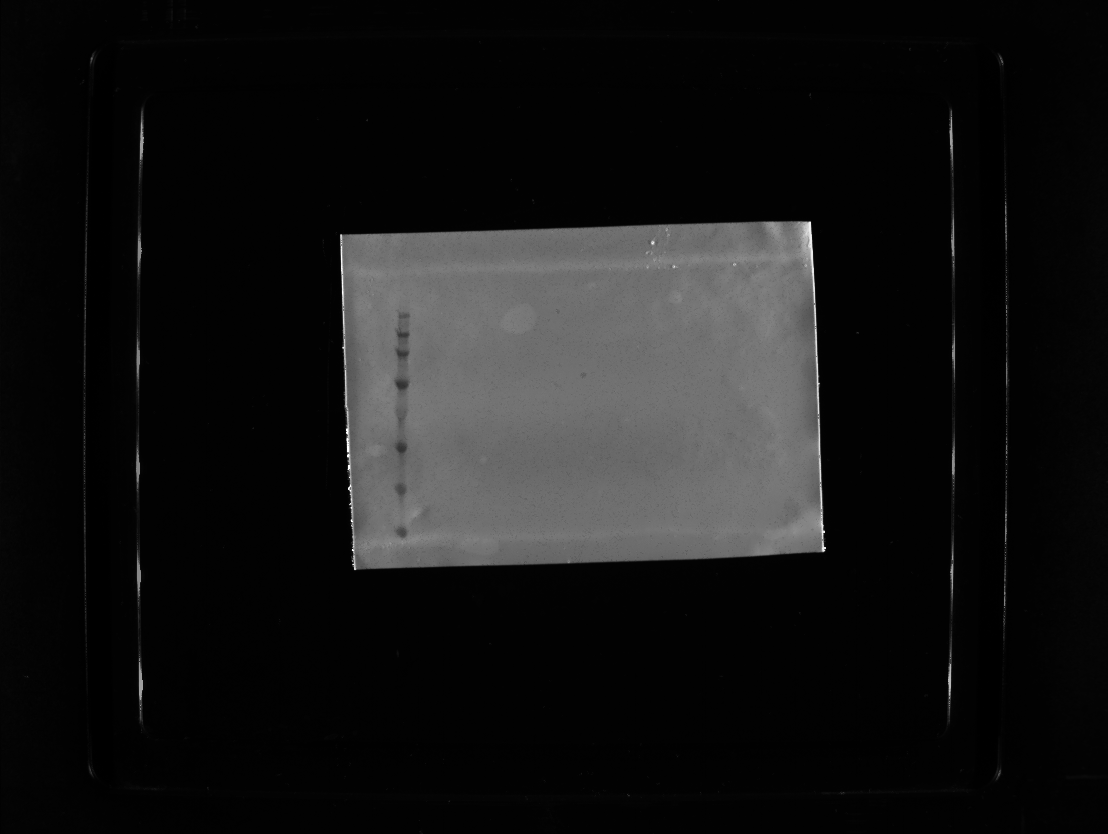

Supplement: Supplementary file 5 — Source data Fig. 4 [file 44318_2025_545_MOESM5_ESM.zip › Fig 4/4C/P ADPr/2024-0614-164132.tif]

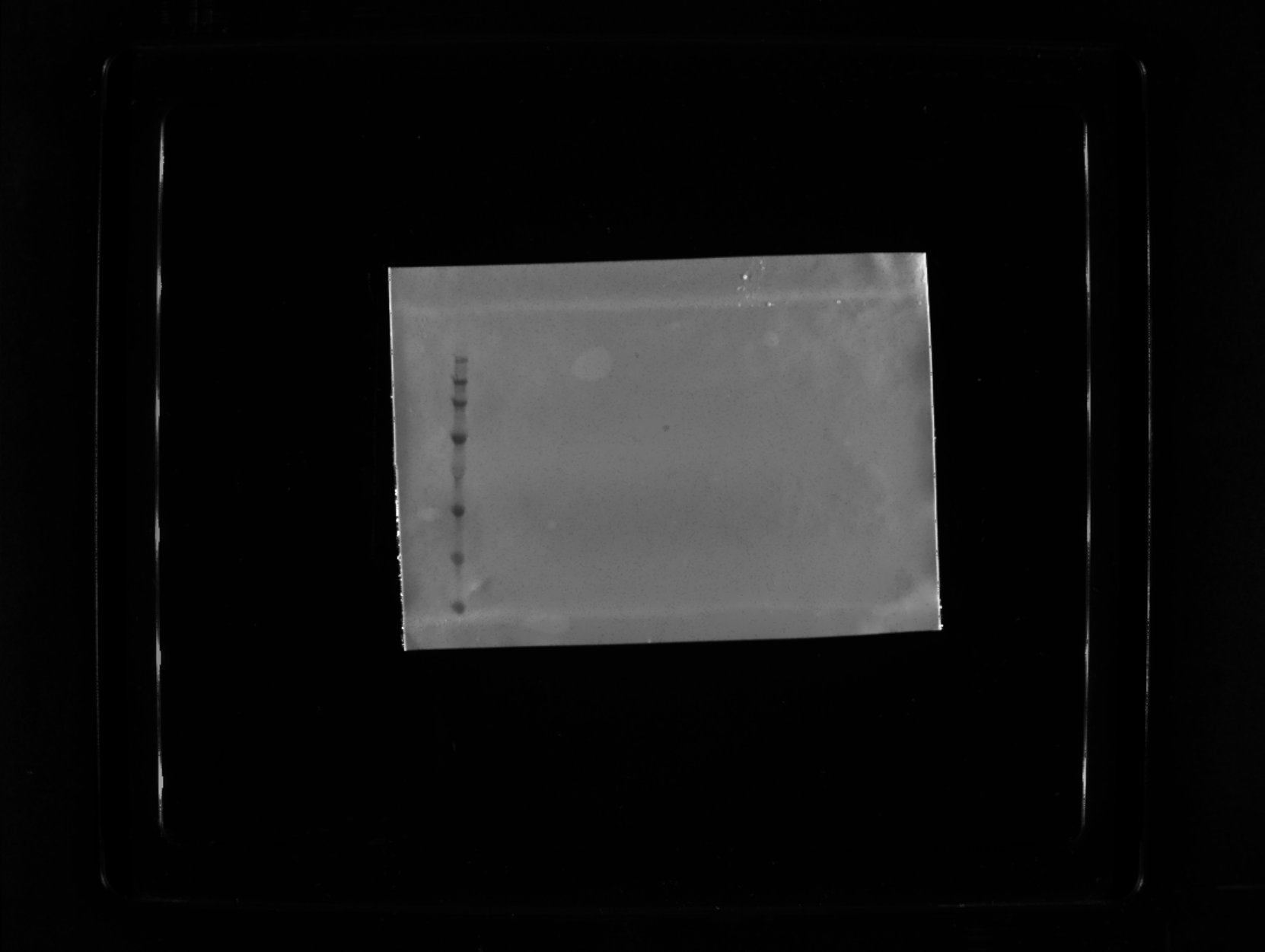

Supplement: Supplementary file 5 — Source data Fig. 4 [file 44318_2025_545_MOESM5_ESM.zip › Fig 4/4C/P ADPr/2024-0614-164132_pub.tif]

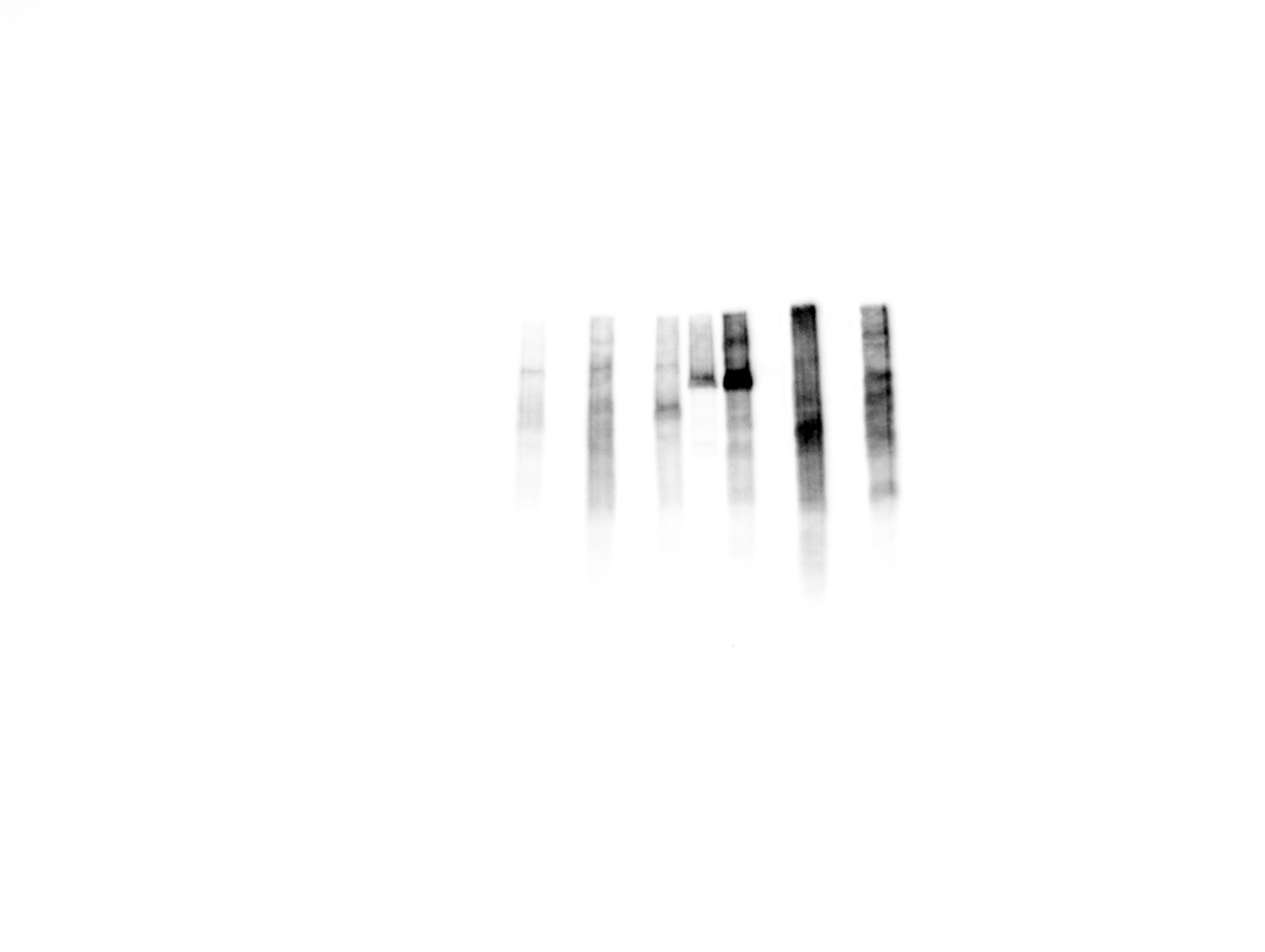

Supplement: Supplementary file 5 — Source data Fig. 4 [file 44318_2025_545_MOESM5_ESM.zip › Fig 4/4C/P ADPr/2024-0614-164133_pub.tif]

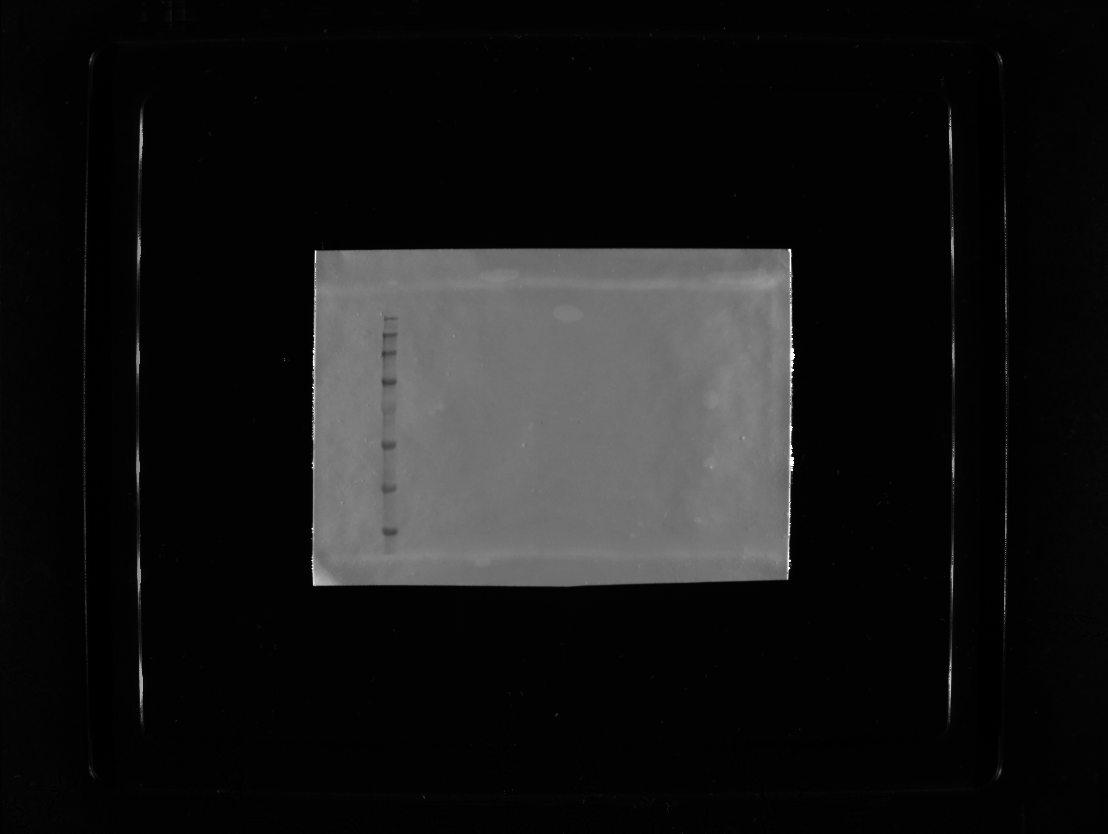

Supplement: Supplementary file 5 — Source data Fig. 4 [file 44318_2025_545_MOESM5_ESM.zip › Fig 4/4D/U2OS CST/2024-0216-110750.tif]

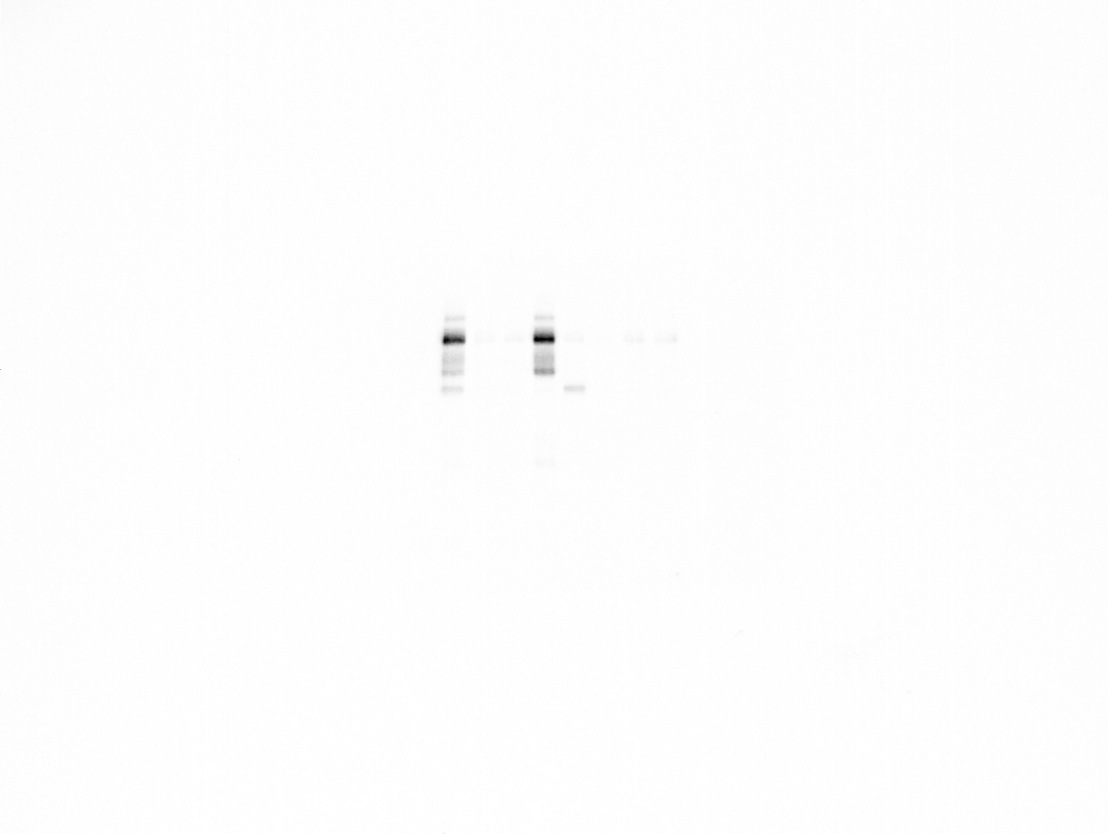

Supplement: Supplementary file 5 — Source data Fig. 4 [file 44318_2025_545_MOESM5_ESM.zip › Fig 4/4D/U2OS CST/2024-0216-110752.tif]

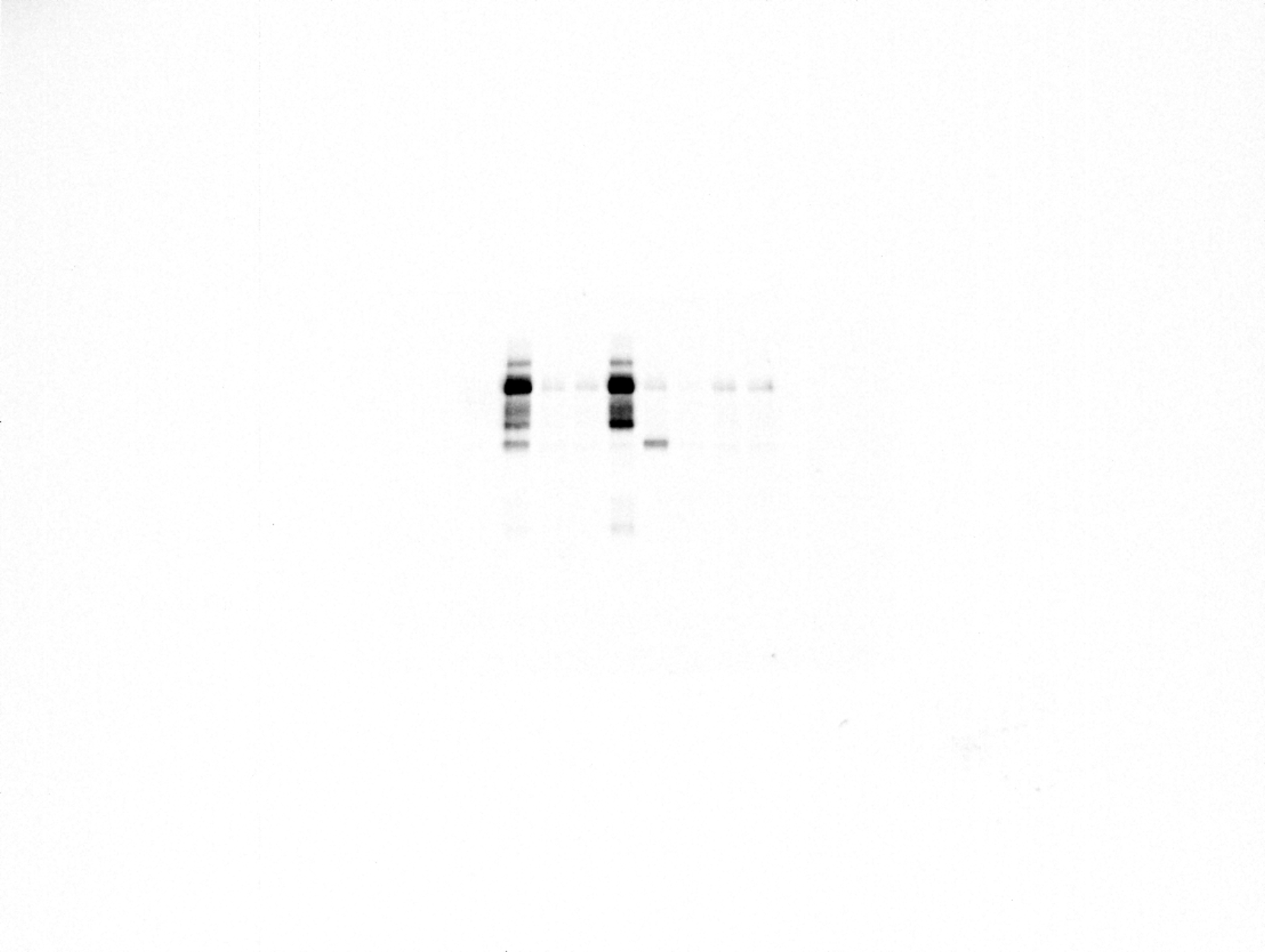

Supplement: Supplementary file 5 — Source data Fig. 4 [file 44318_2025_545_MOESM5_ESM.zip › Fig 4/4D/U2OS CST/2024-0216-110752_pub.tif]

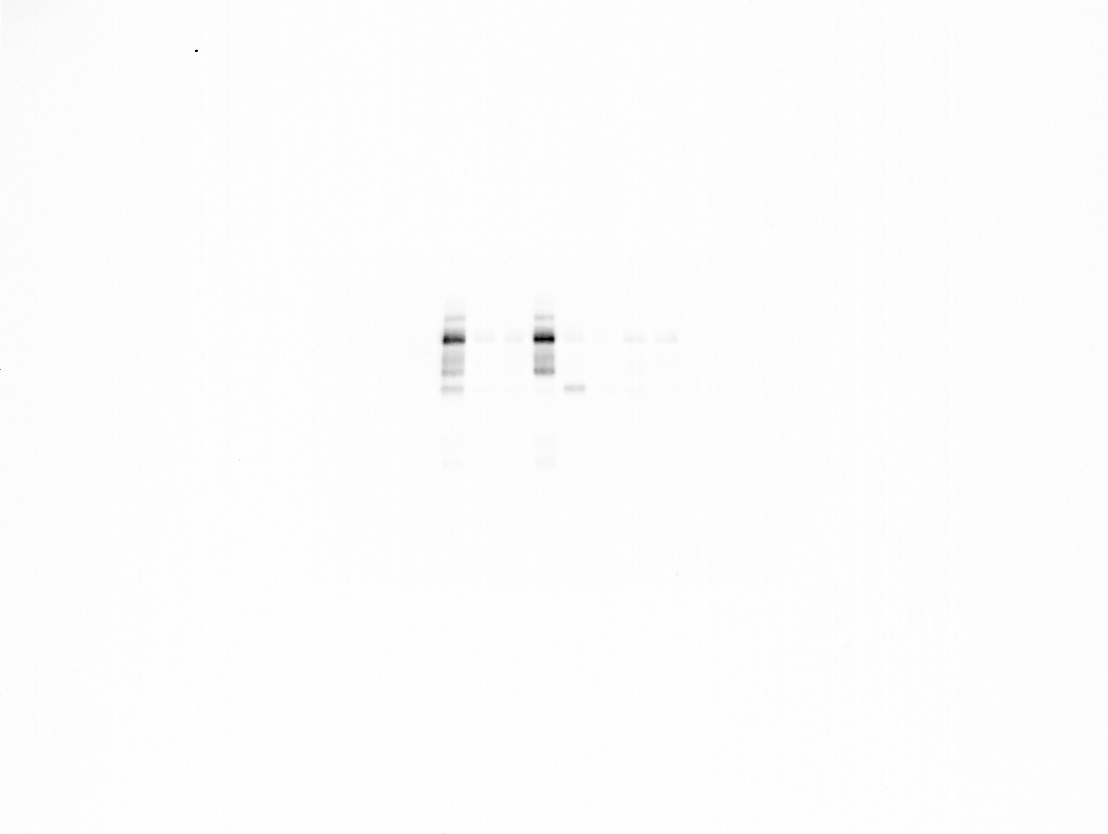

Supplement: Supplementary file 5 — Source data Fig. 4 [file 44318_2025_545_MOESM5_ESM.zip › Fig 4/4D/U2OS CST/S1F2-0216-110755.tif]

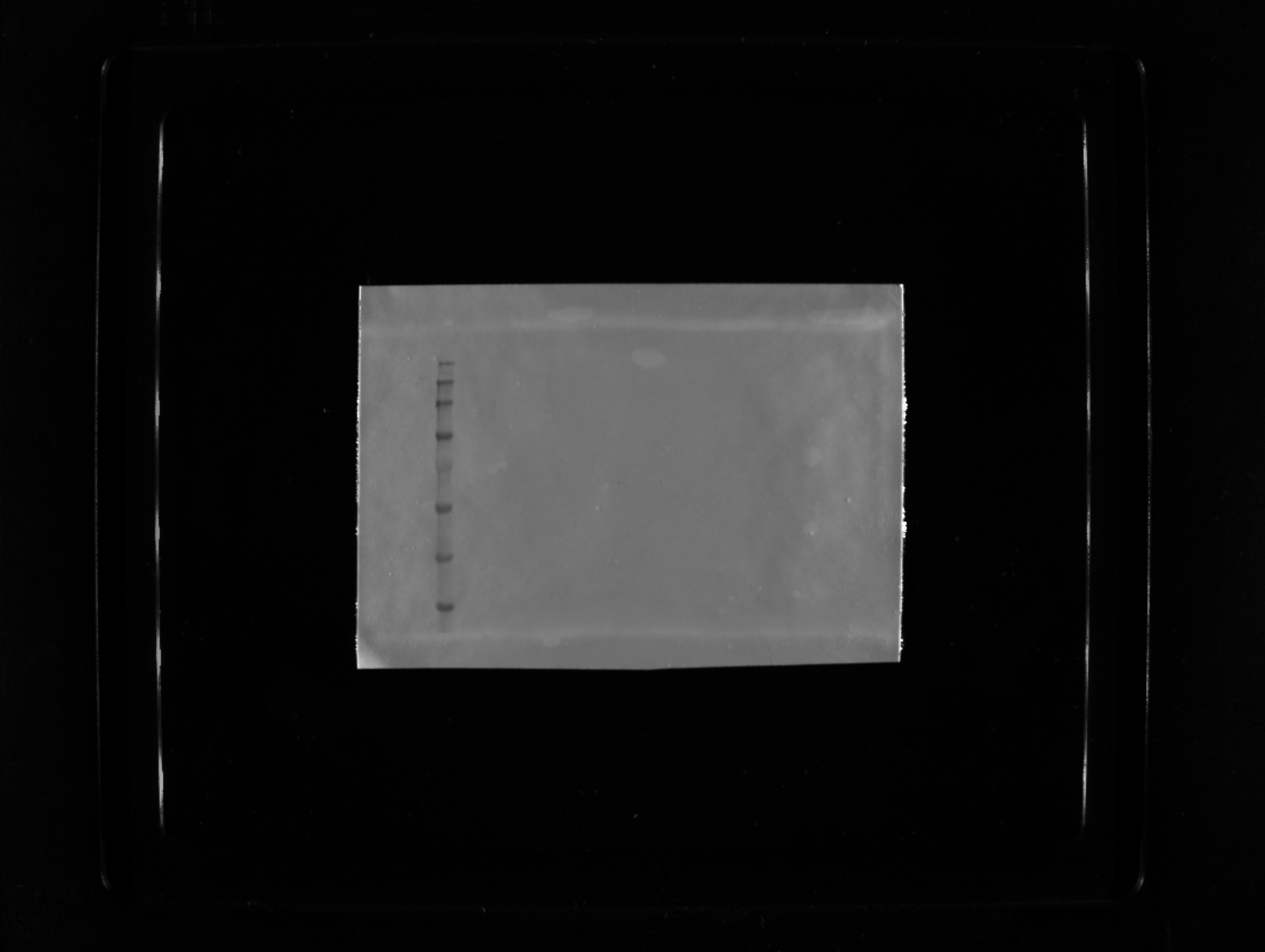

Supplement: Supplementary file 5 — Source data Fig. 4 [file 44318_2025_545_MOESM5_ESM.zip › Fig 4/4D/U2OS CST/2024-0216-110750_pub.tif]

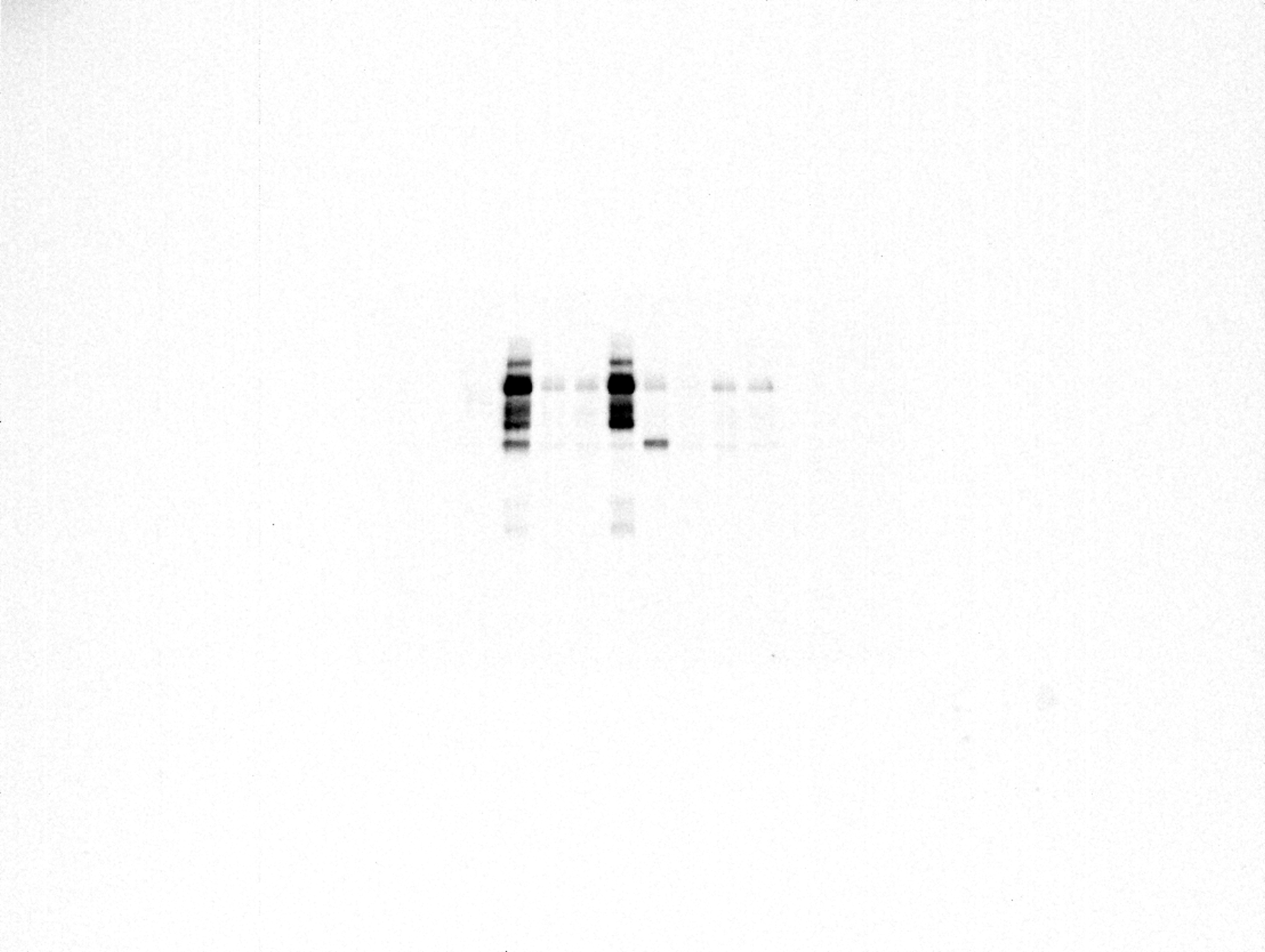

Supplement: Supplementary file 5 — Source data Fig. 4 [file 44318_2025_545_MOESM5_ESM.zip › Fig 4/4D/U2OS CST/S1F1-0216-110753_pub.tif]

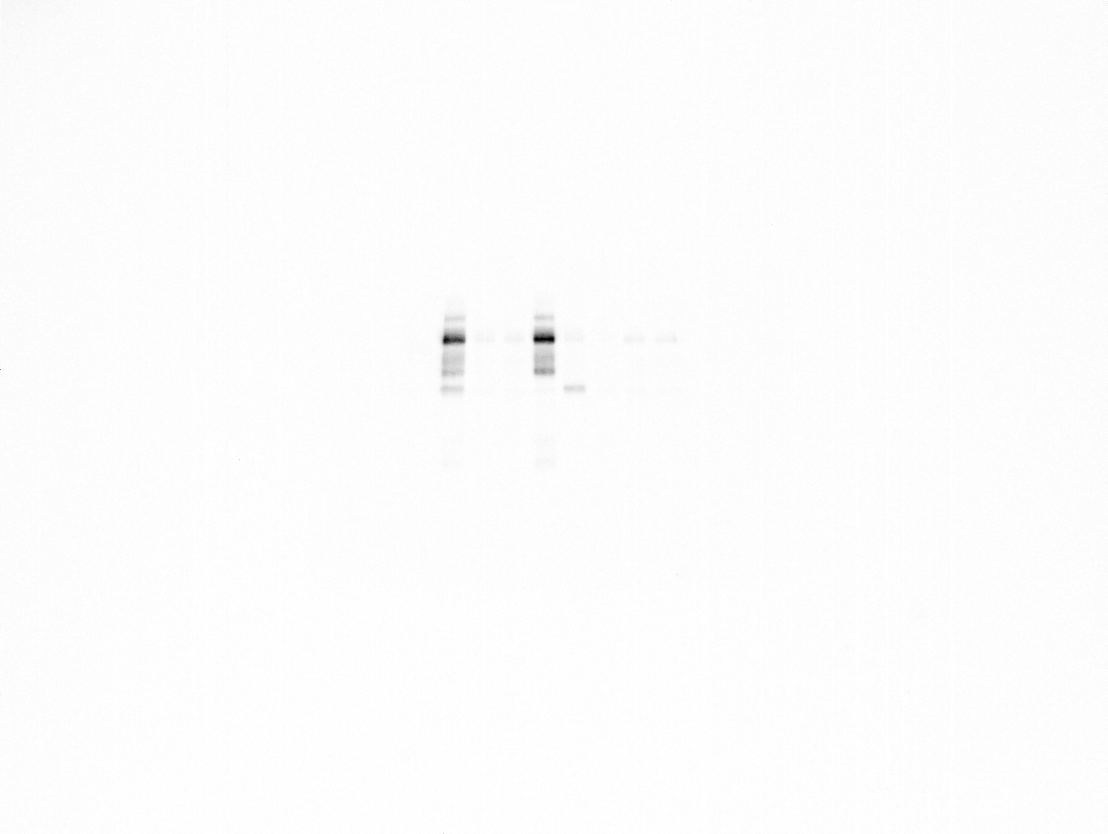

Supplement: Supplementary file 5 — Source data Fig. 4 [file 44318_2025_545_MOESM5_ESM.zip › Fig 4/4D/U2OS CST/S1F1-0216-110753.tif]

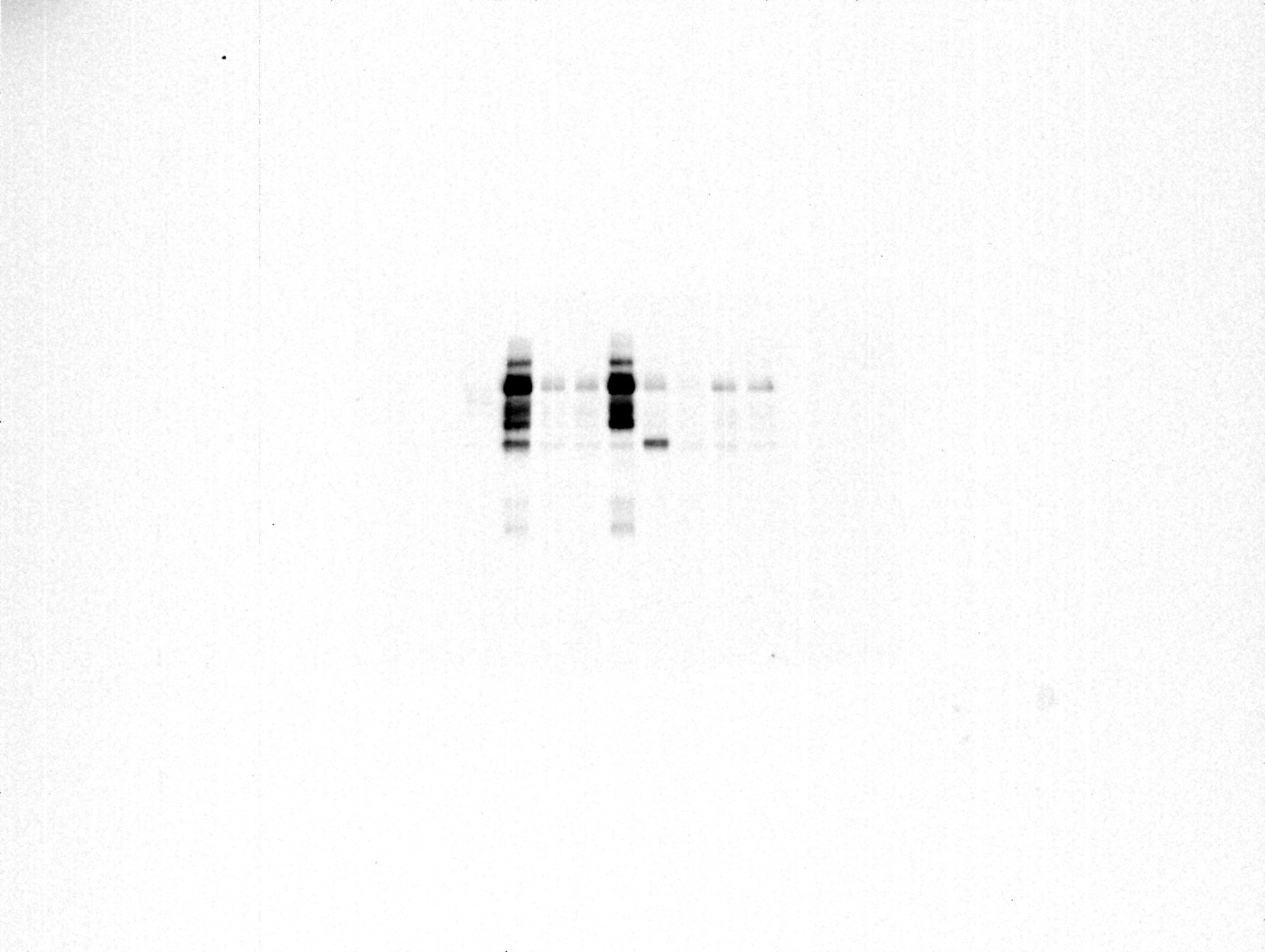

Supplement: Supplementary file 5 — Source data Fig. 4 [file 44318_2025_545_MOESM5_ESM.zip › Fig 4/4D/U2OS CST/S1F2-0216-110755_pub.tif]

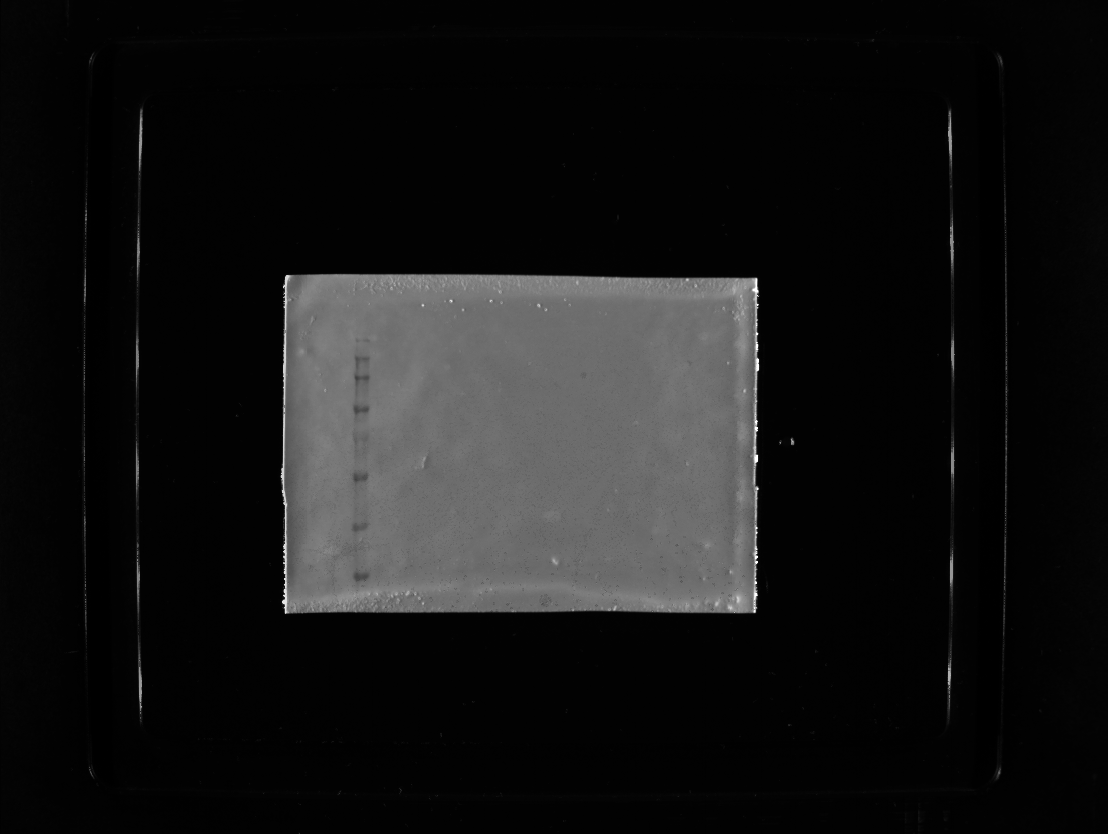

Supplement: Supplementary file 5 — Source data Fig. 4 [file 44318_2025_545_MOESM5_ESM.zip › Fig 4/4D/ HeLa Tub/2024-0219-133607.tif]

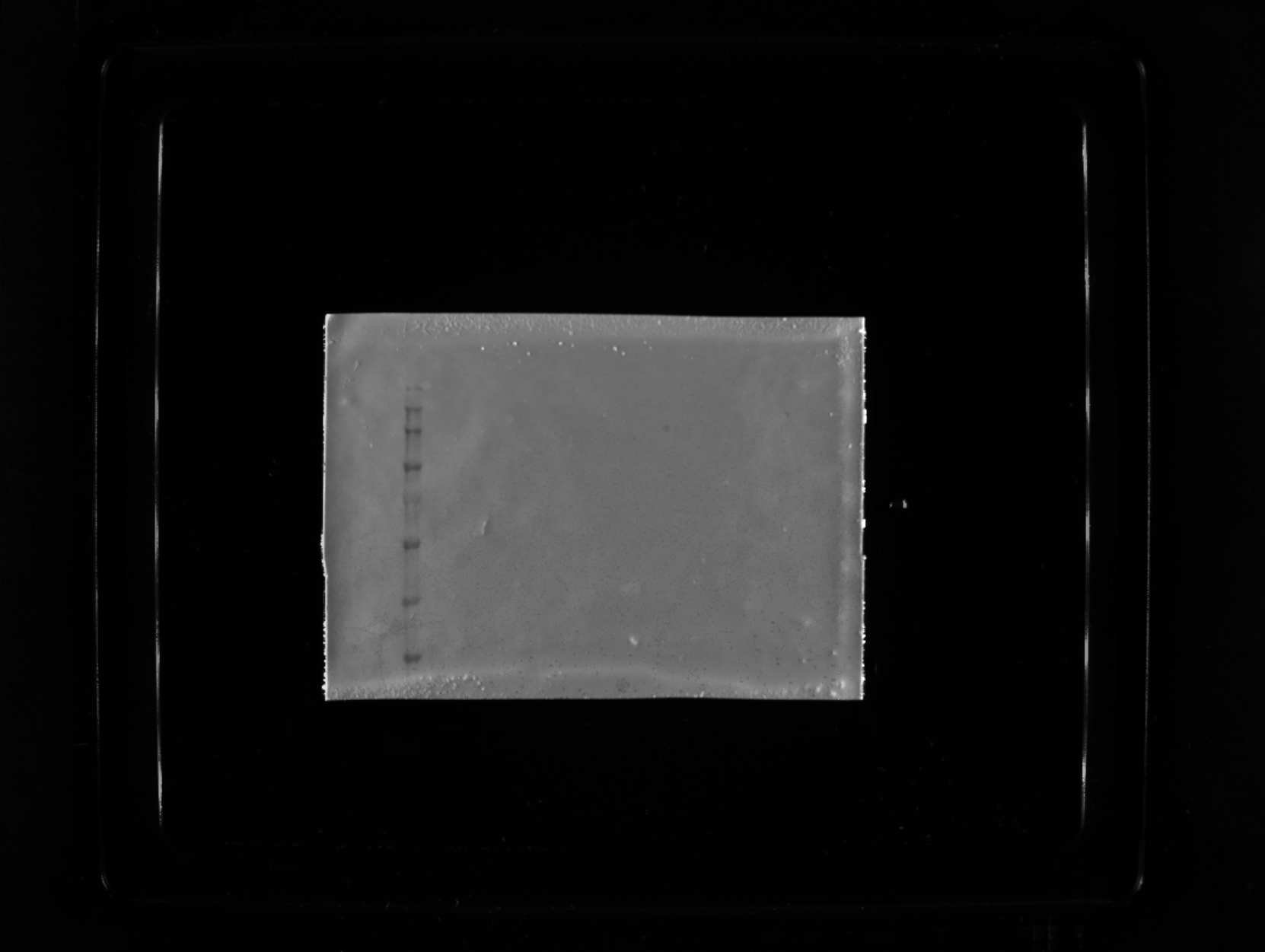

Supplement: Supplementary file 5 — Source data Fig. 4 [file 44318_2025_545_MOESM5_ESM.zip › Fig 4/4D/ HeLa Tub/2024-0219-133607_pub.tif]

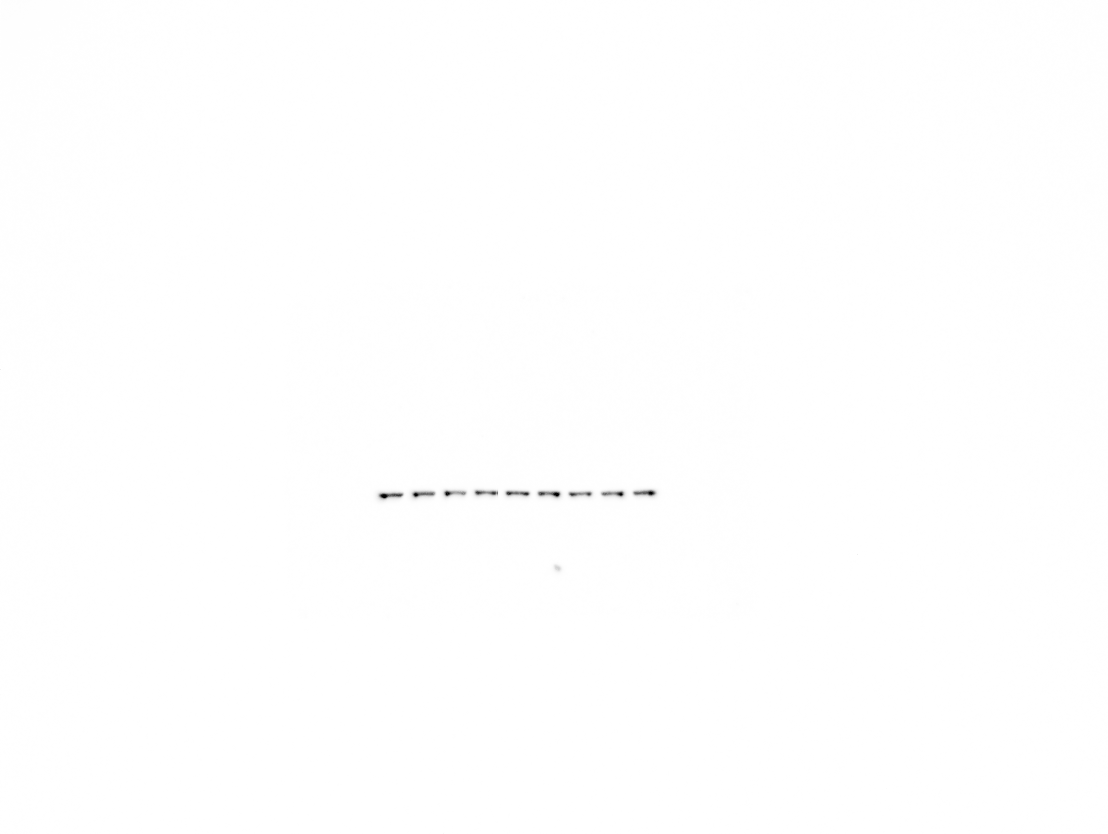

Supplement: Supplementary file 5 — Source data Fig. 4 [file 44318_2025_545_MOESM5_ESM.zip › Fig 4/4D/ HeLa Tub/2024-0219-133608.tif]

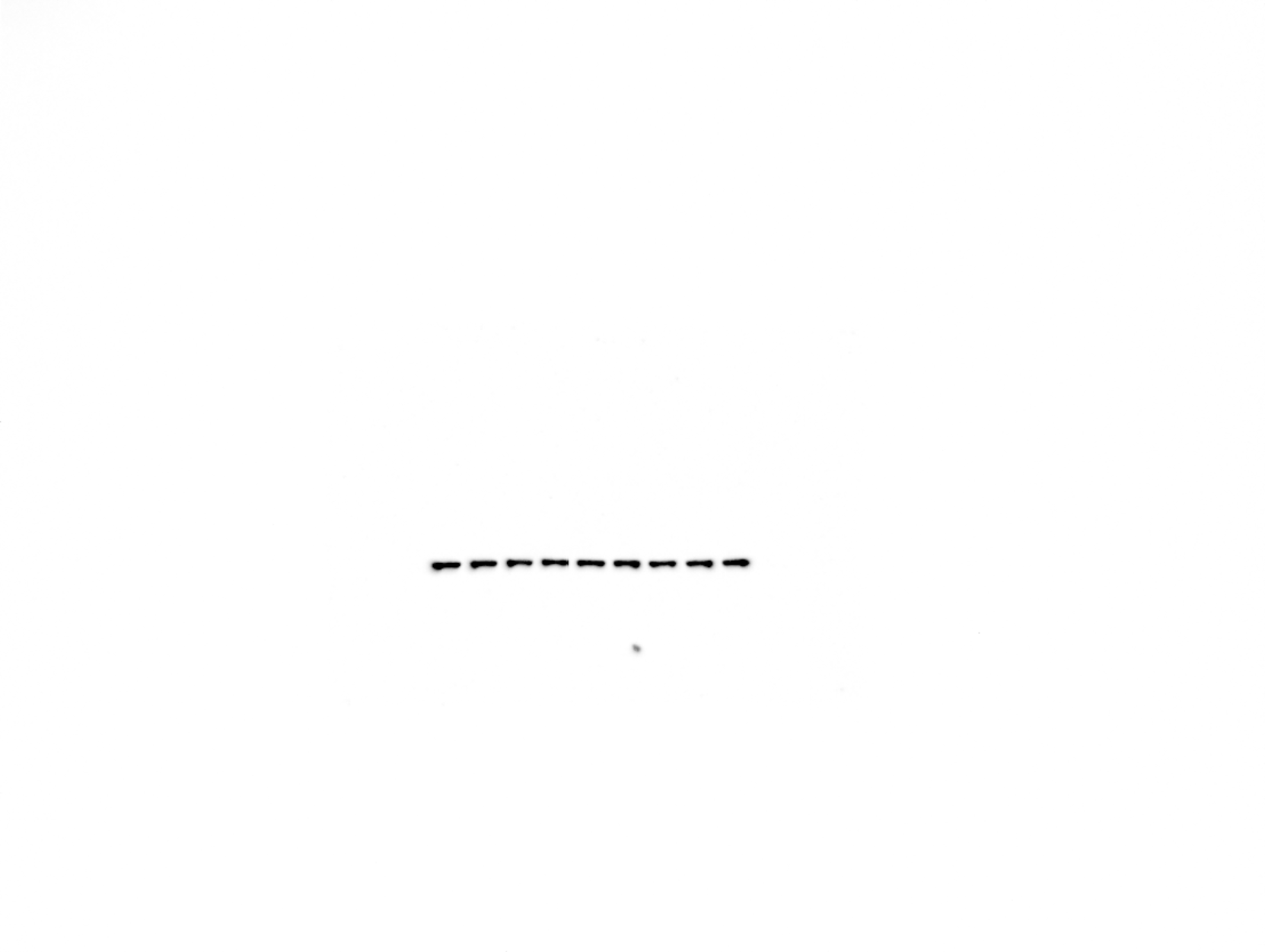

Supplement: Supplementary file 5 — Source data Fig. 4 [file 44318_2025_545_MOESM5_ESM.zip › Fig 4/4D/ HeLa Tub/2024-0219-133608_pub.tif]

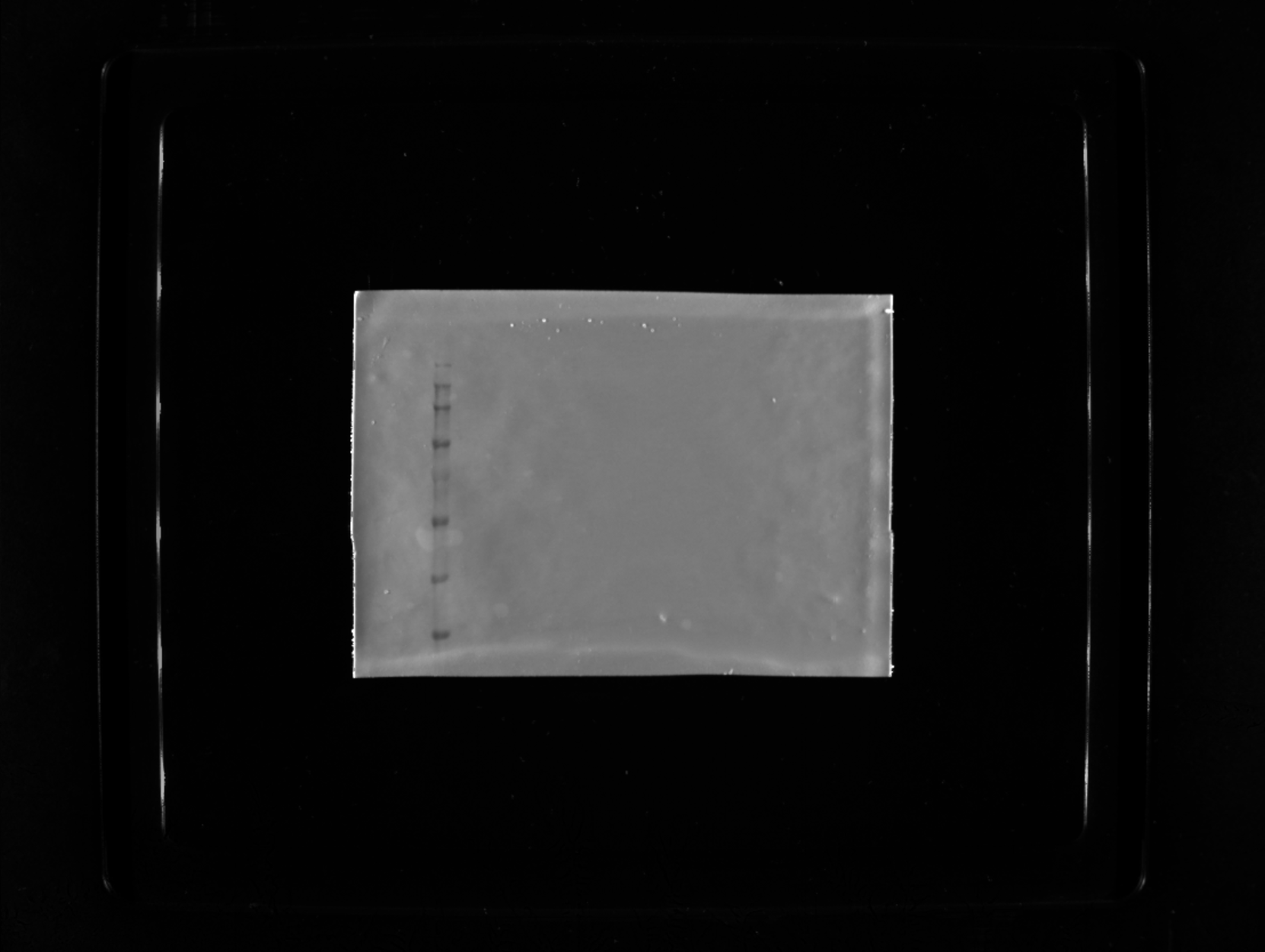

Supplement: Supplementary file 5 — Source data Fig. 4 [file 44318_2025_545_MOESM5_ESM.zip › Fig 4/4D/HeLa CST/2024-0216-105859_pub.tif]

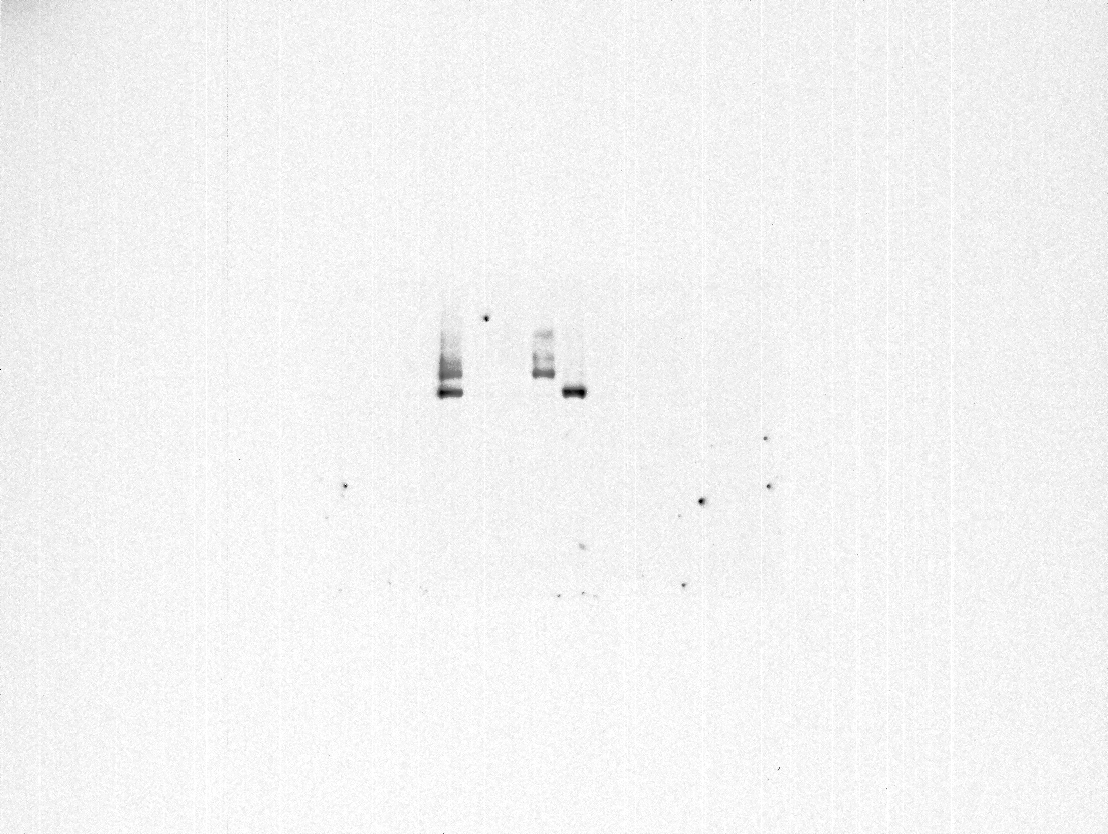

Supplement: Supplementary file 5 — Source data Fig. 4 [file 44318_2025_545_MOESM5_ESM.zip › Fig 4/4D/HeLa CST/2024-0216-105900.tif]

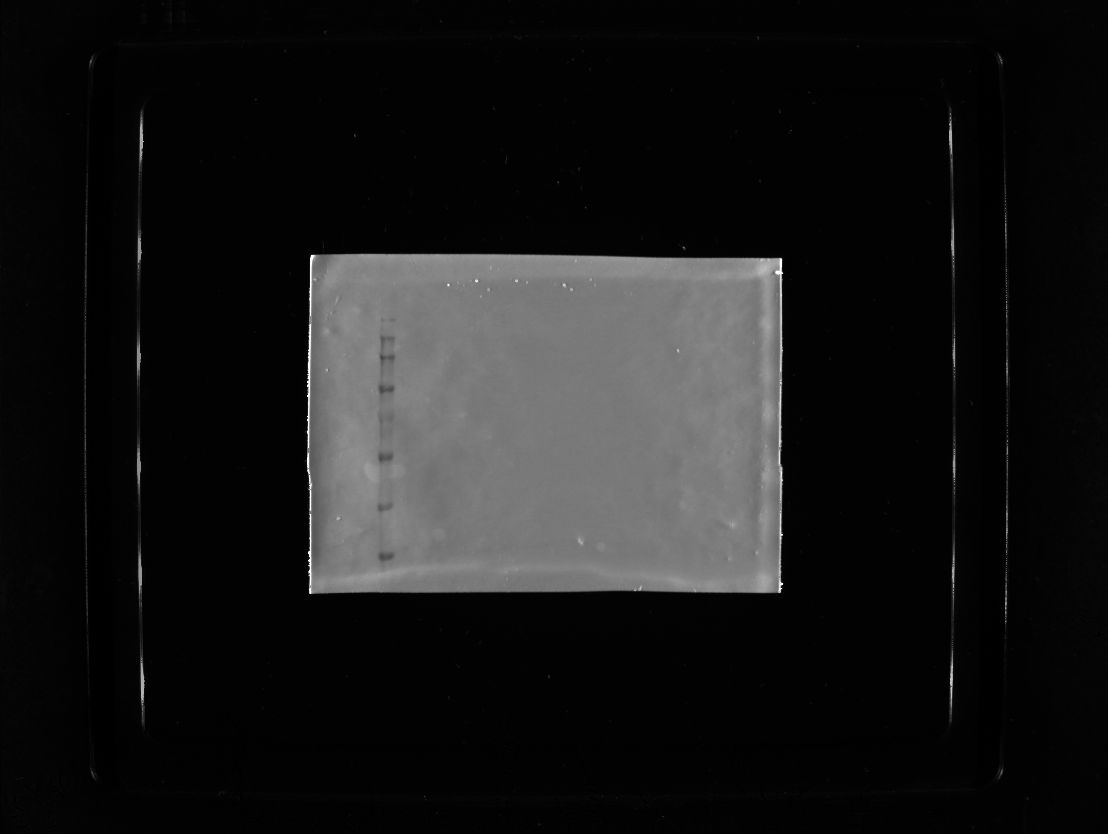

Supplement: Supplementary file 5 — Source data Fig. 4 [file 44318_2025_545_MOESM5_ESM.zip › Fig 4/4D/HeLa CST/2024-0216-105859.tif]

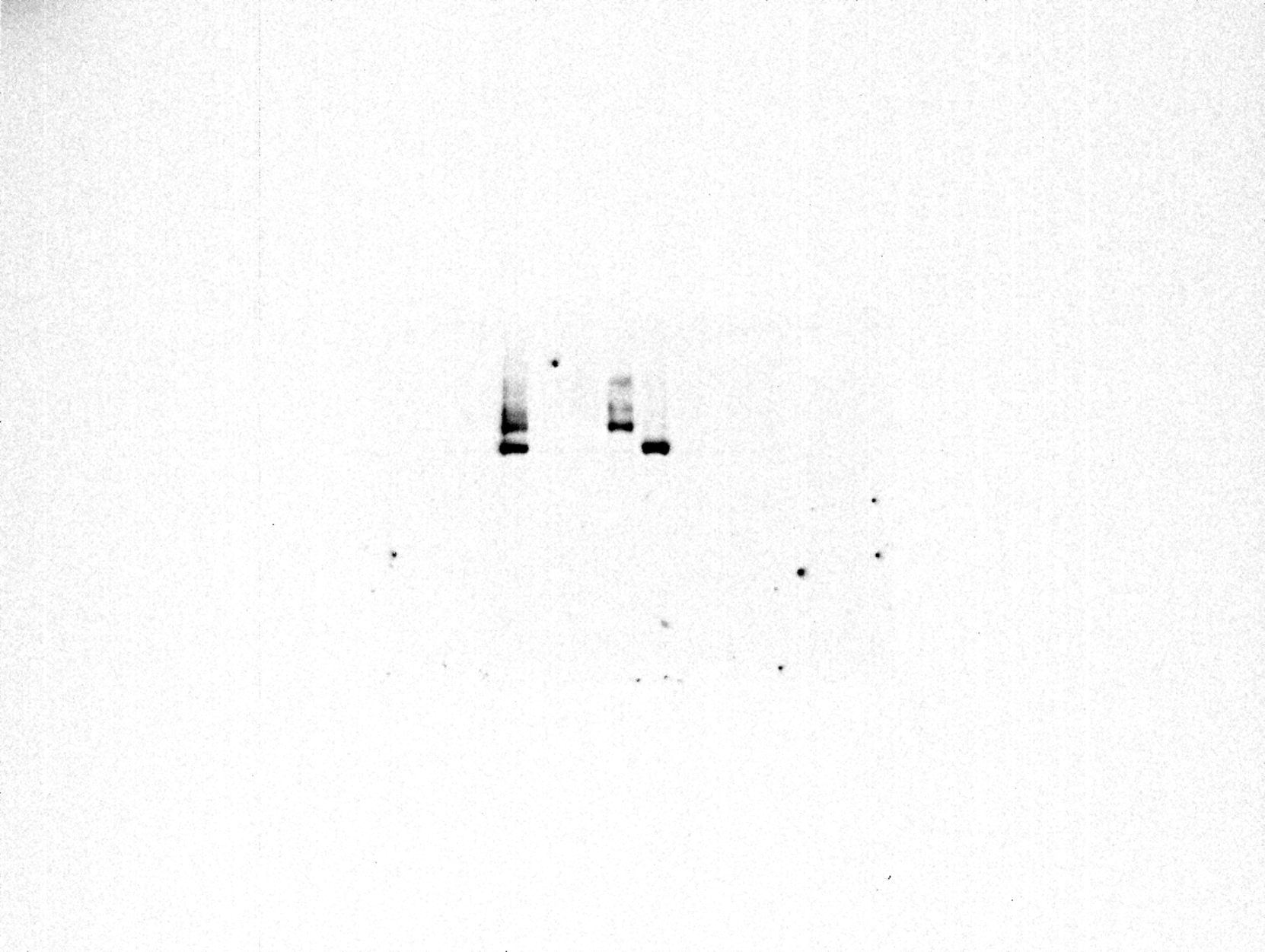

Supplement: Supplementary file 5 — Source data Fig. 4 [file 44318_2025_545_MOESM5_ESM.zip › Fig 4/4D/HeLa CST/2024-0216-105900_pub.tif]

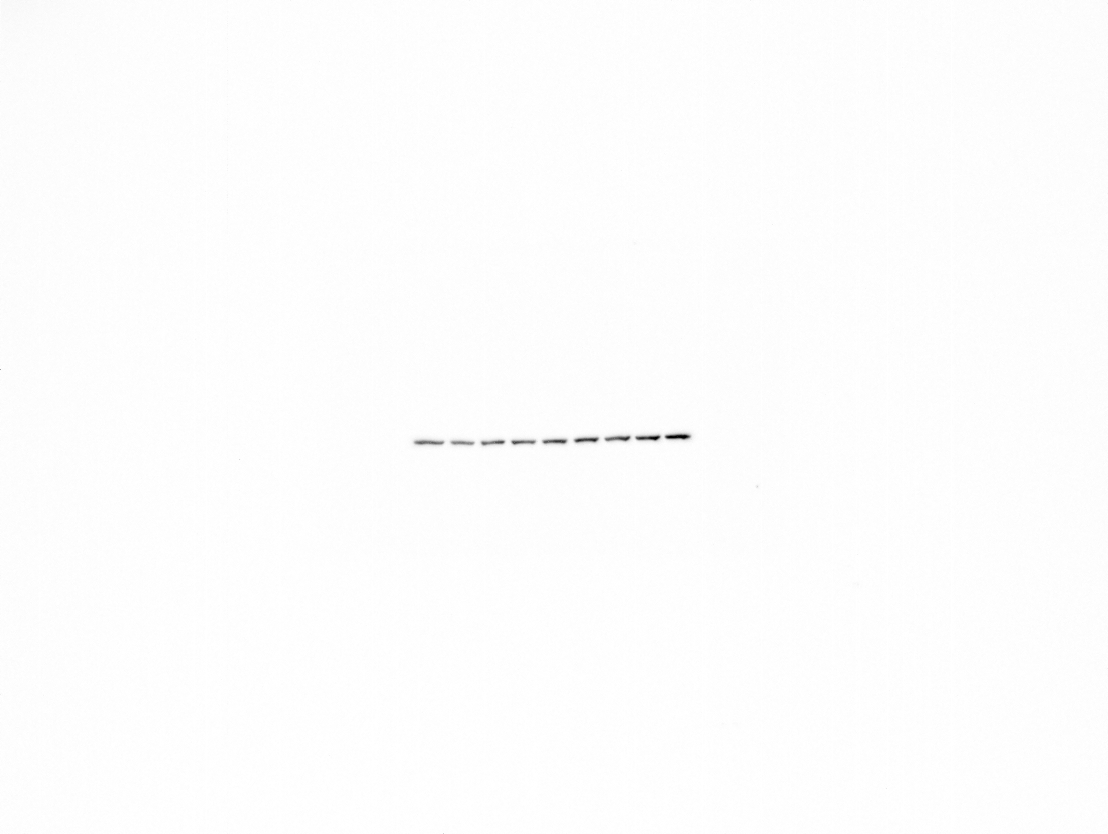

Supplement: Supplementary file 5 — Source data Fig. 4 [file 44318_2025_545_MOESM5_ESM.zip › Fig 4/4D/U2OS Tub/2024-0216-112420.tif]

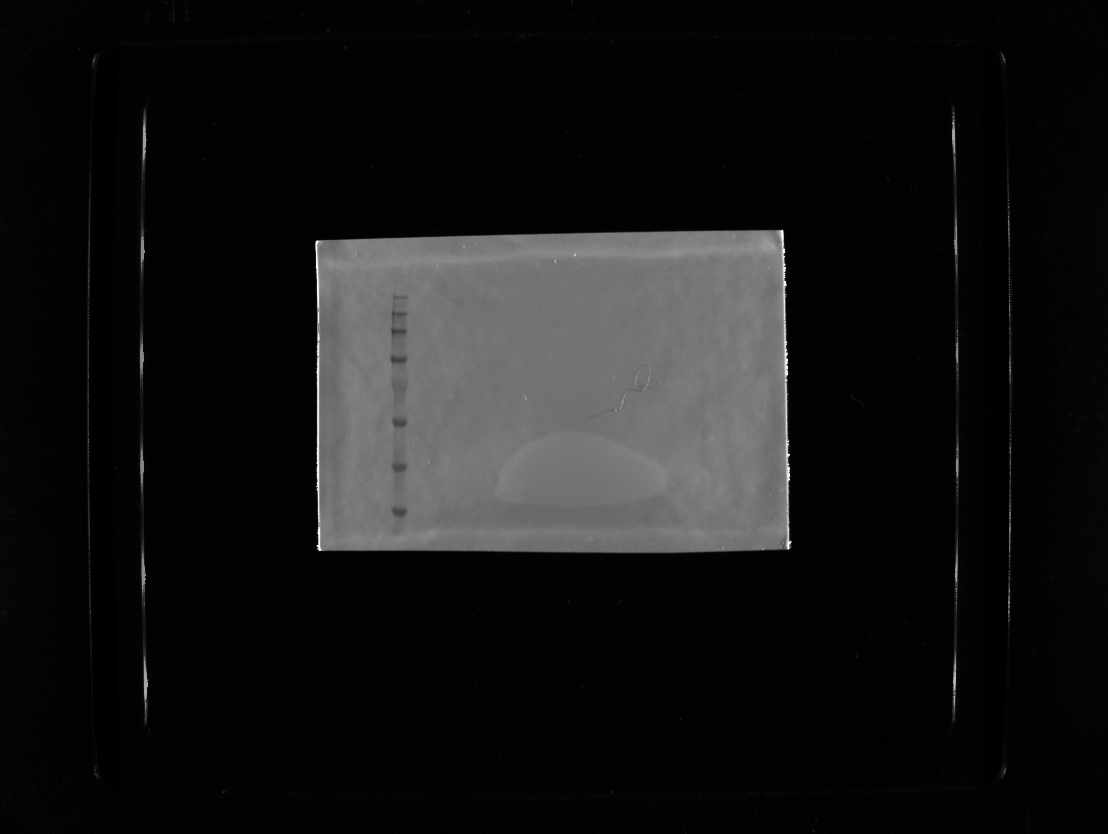

Supplement: Supplementary file 5 — Source data Fig. 4 [file 44318_2025_545_MOESM5_ESM.zip › Fig 4/4D/U2OS Tub/2024-0216-112418.tif]

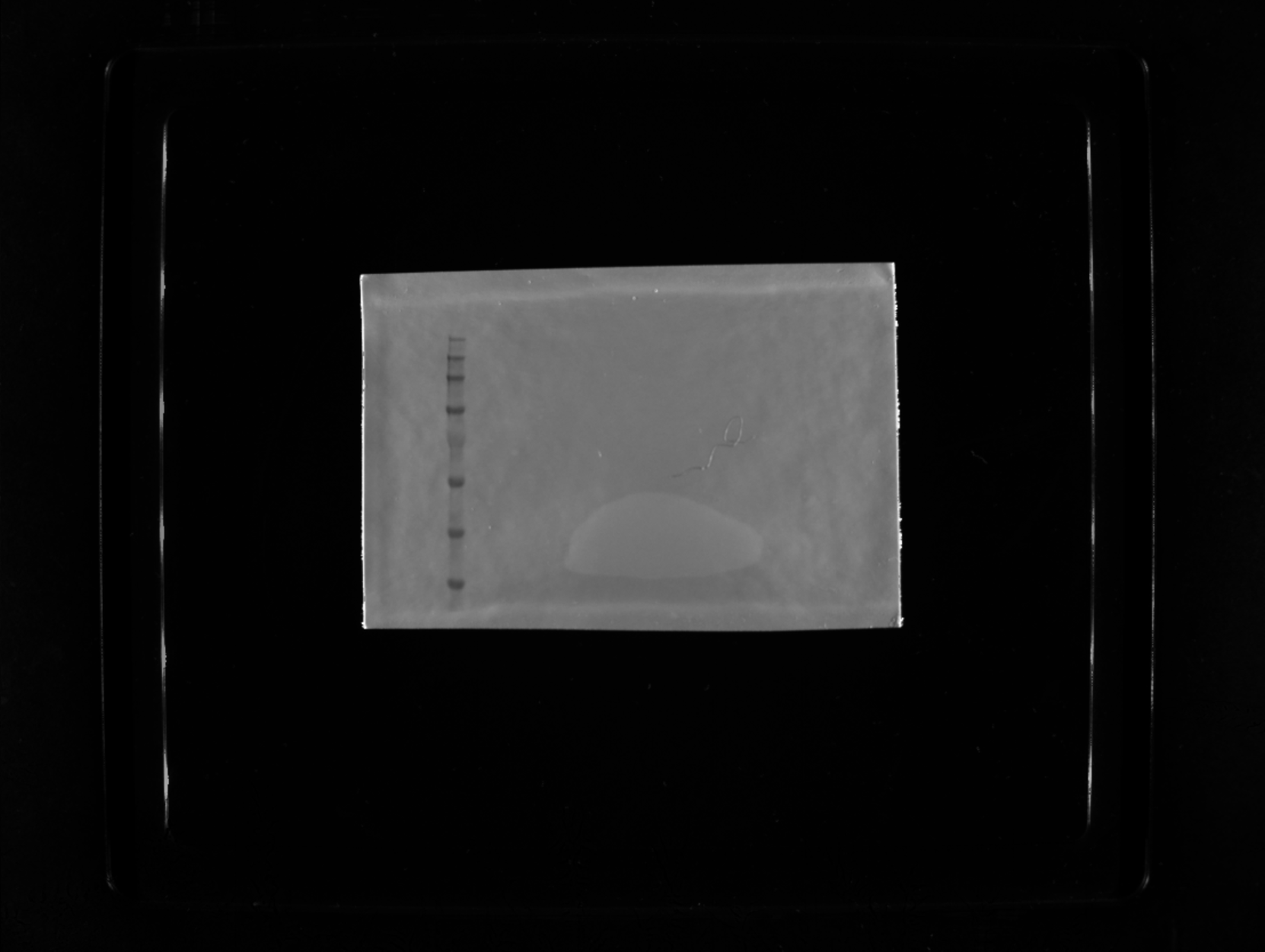

Supplement: Supplementary file 5 — Source data Fig. 4 [file 44318_2025_545_MOESM5_ESM.zip › Fig 4/4D/U2OS Tub/2024-0216-112418_pub.tif]

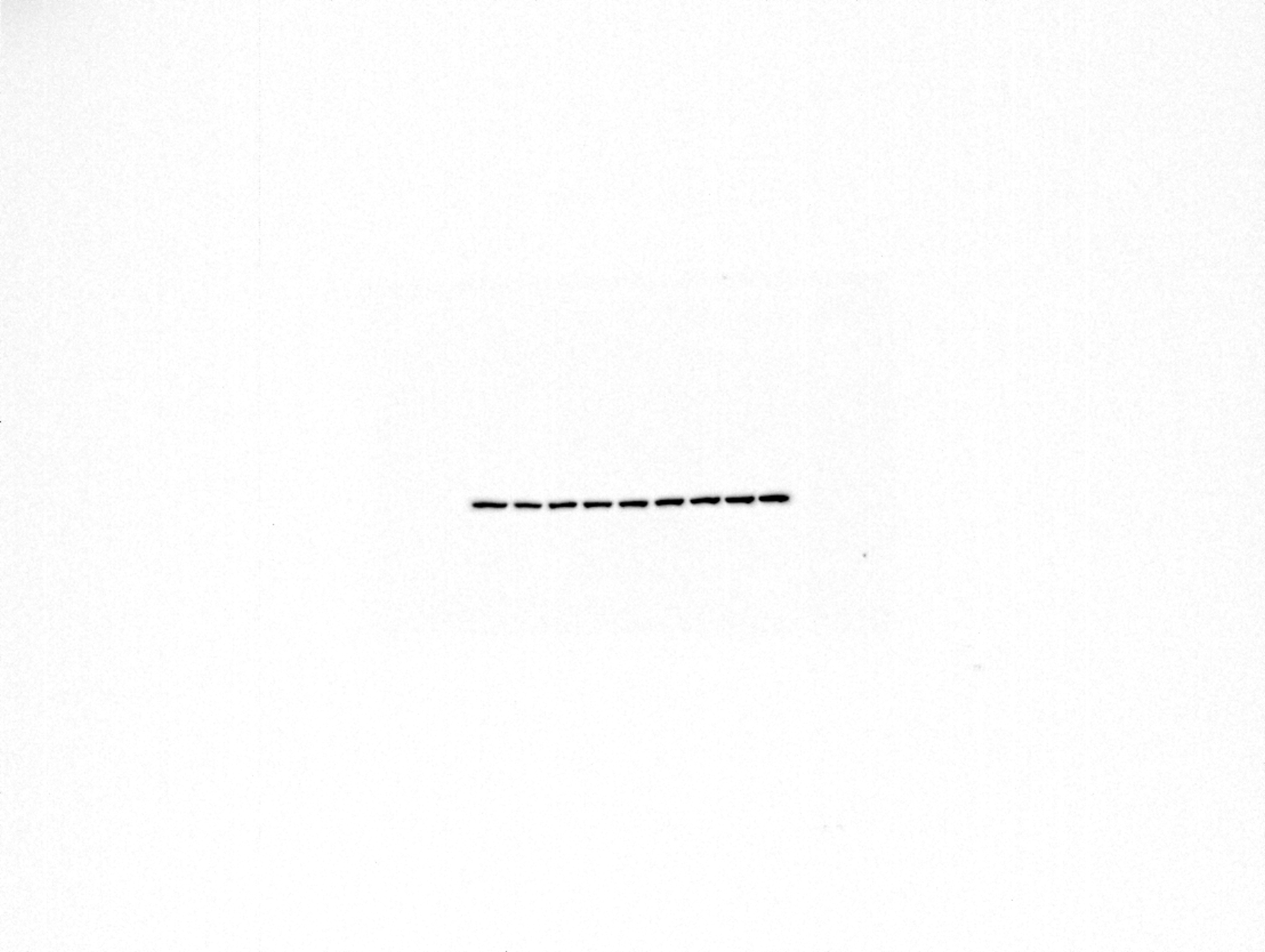

Supplement: Supplementary file 5 — Source data Fig. 4 [file 44318_2025_545_MOESM5_ESM.zip › Fig 4/4D/U2OS Tub/2024-0216-112420_pub.tif]

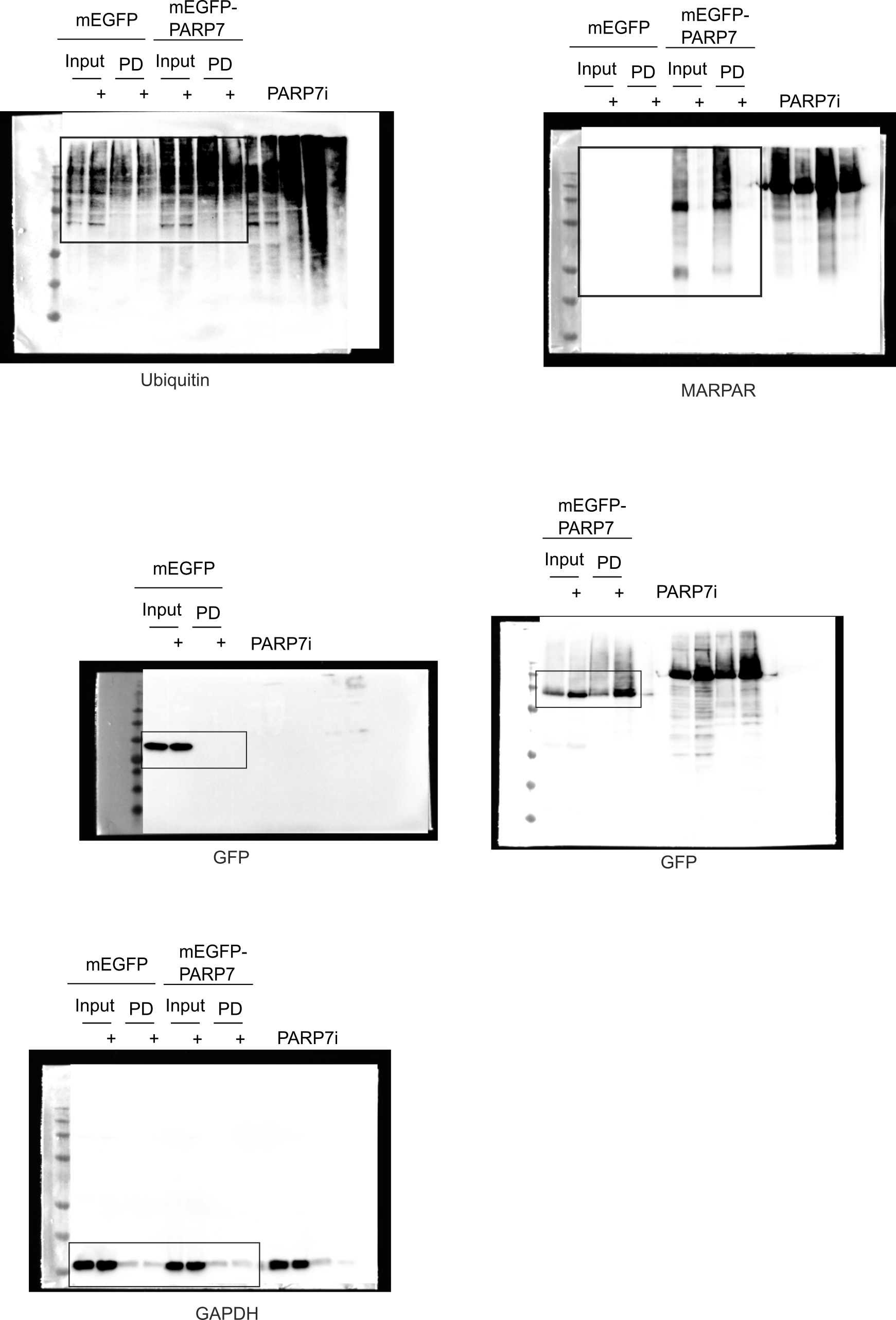

Supplement: Supplementary file 6 — Source data Fig. 5 [file 44318_2025_545_MOESM6_ESM.zip › Fig 5 SD/5C/EMBOJ-2025-120138R1-Figure_5_Source_Data_labeled-sd.png]

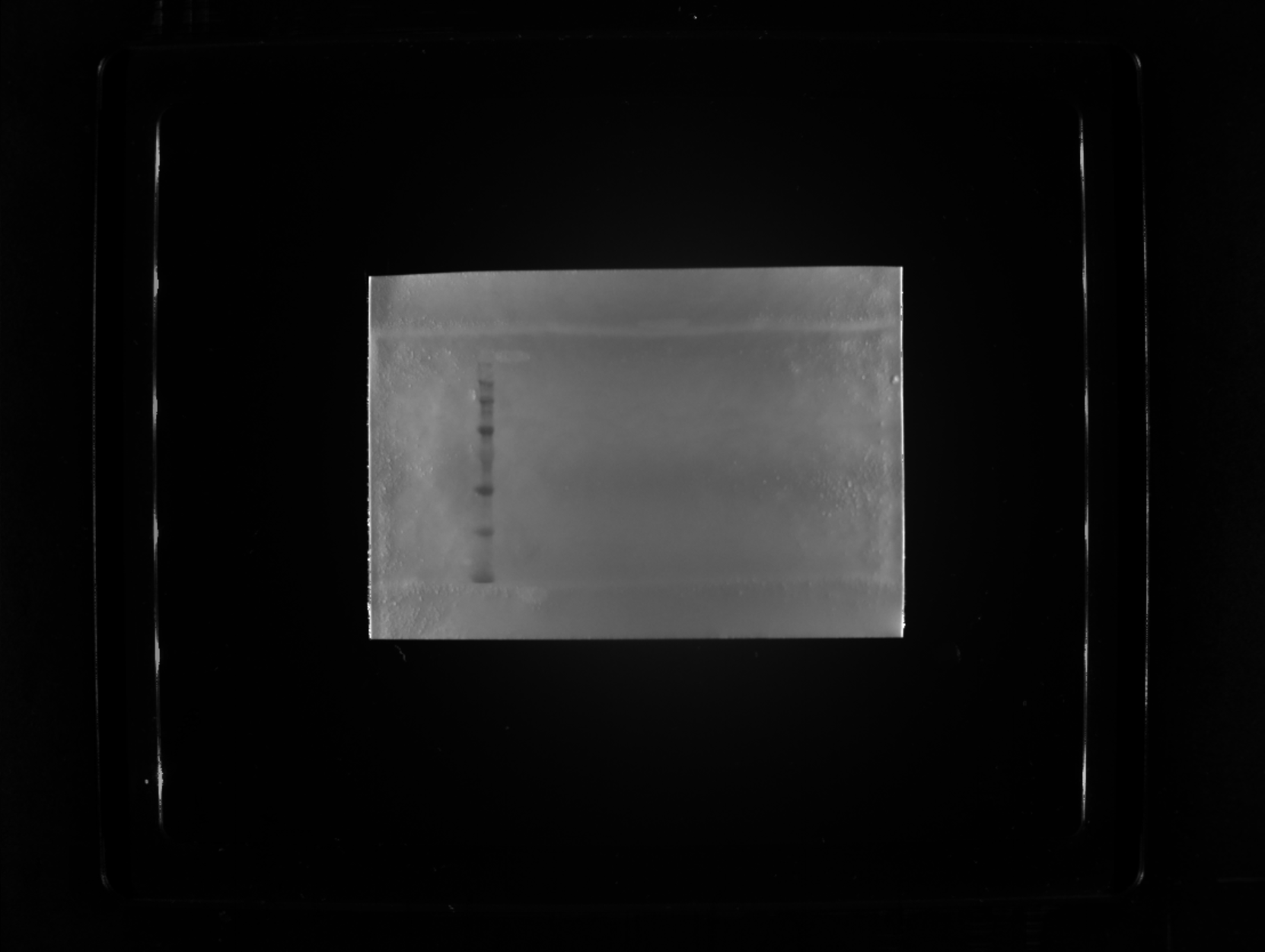

Supplement: Supplementary file 6 — Source data Fig. 5 [file 44318_2025_545_MOESM6_ESM.zip › Fig 5 SD/5A/ADPr/2024-0819-113756_pub.tif]

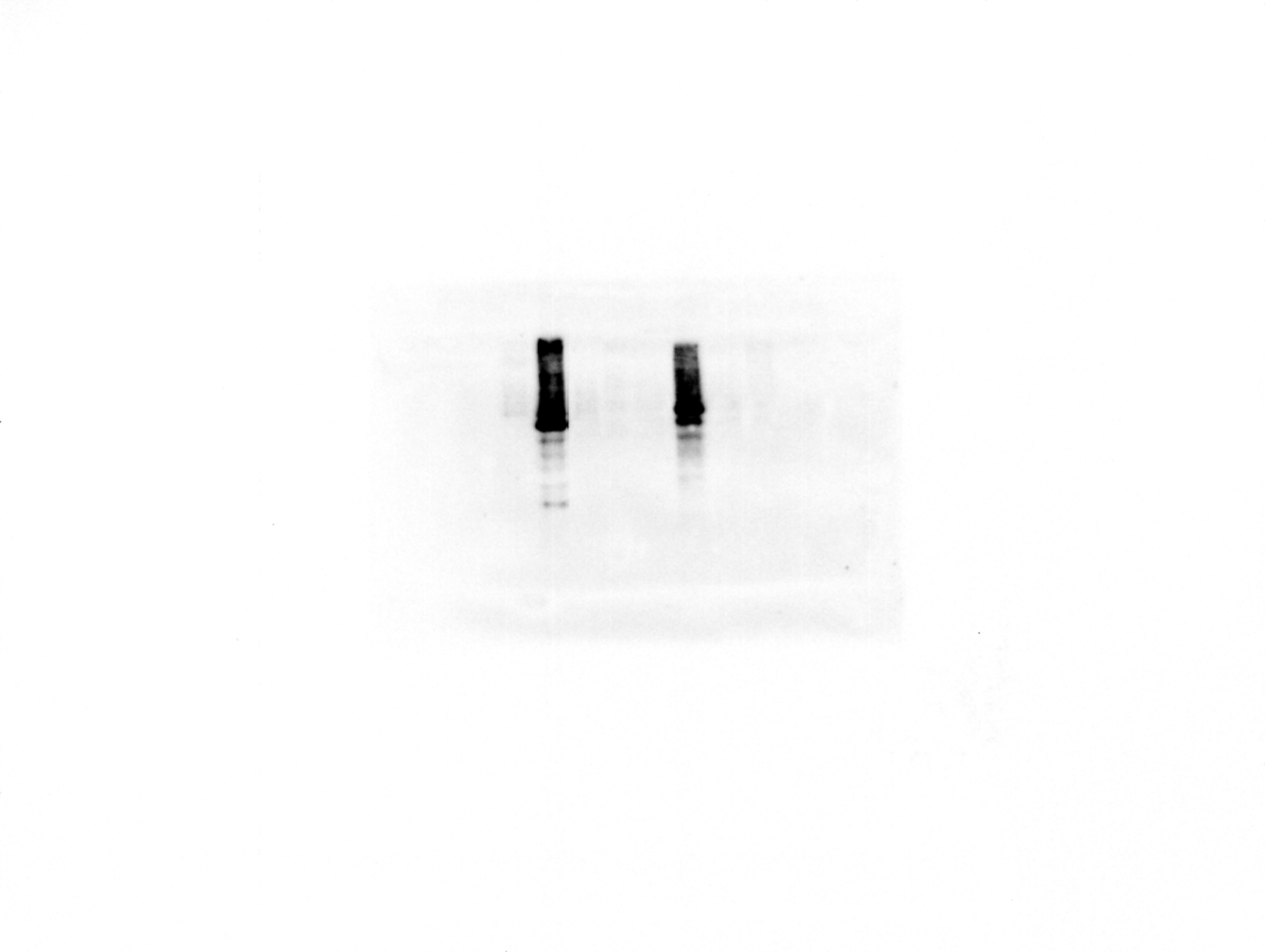

Supplement: Supplementary file 6 — Source data Fig. 5 [file 44318_2025_545_MOESM6_ESM.zip › Fig 5 SD/5A/ADPr/2024-0819-113757_pub.tif]

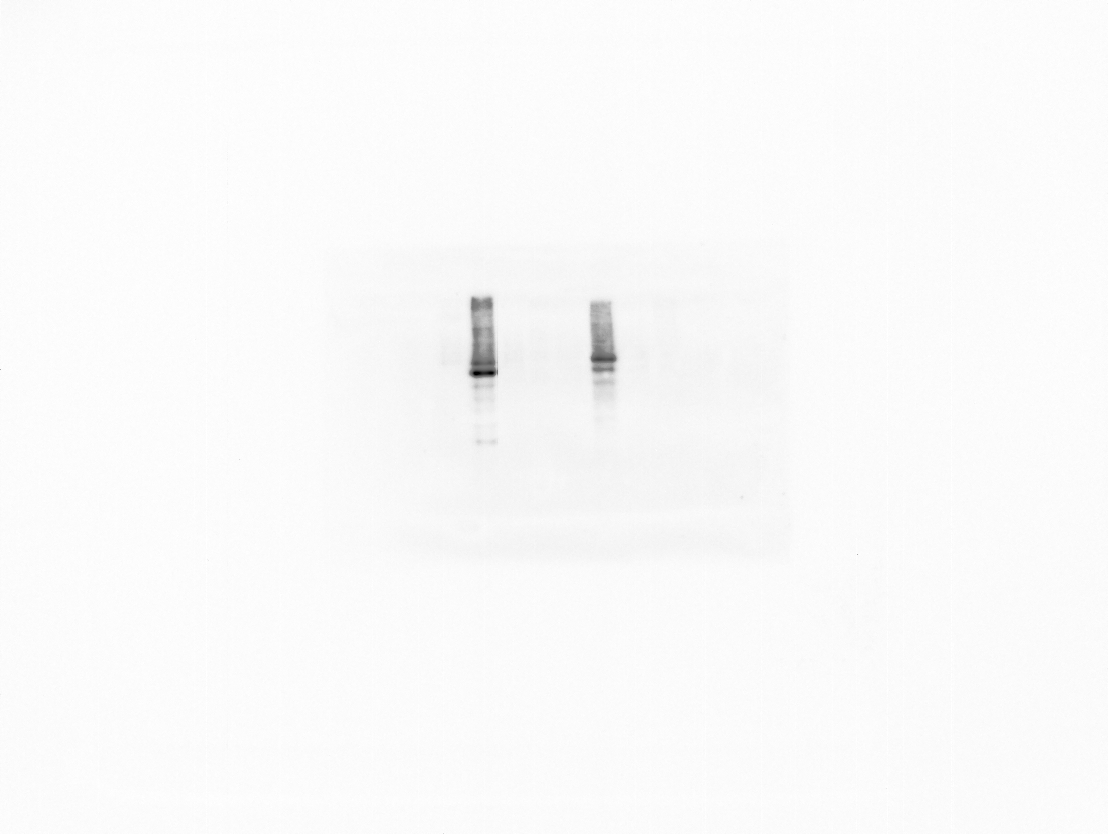

Supplement: Supplementary file 6 — Source data Fig. 5 [file 44318_2025_545_MOESM6_ESM.zip › Fig 5 SD/5A/ADPr/2024-0819-113757.tif]

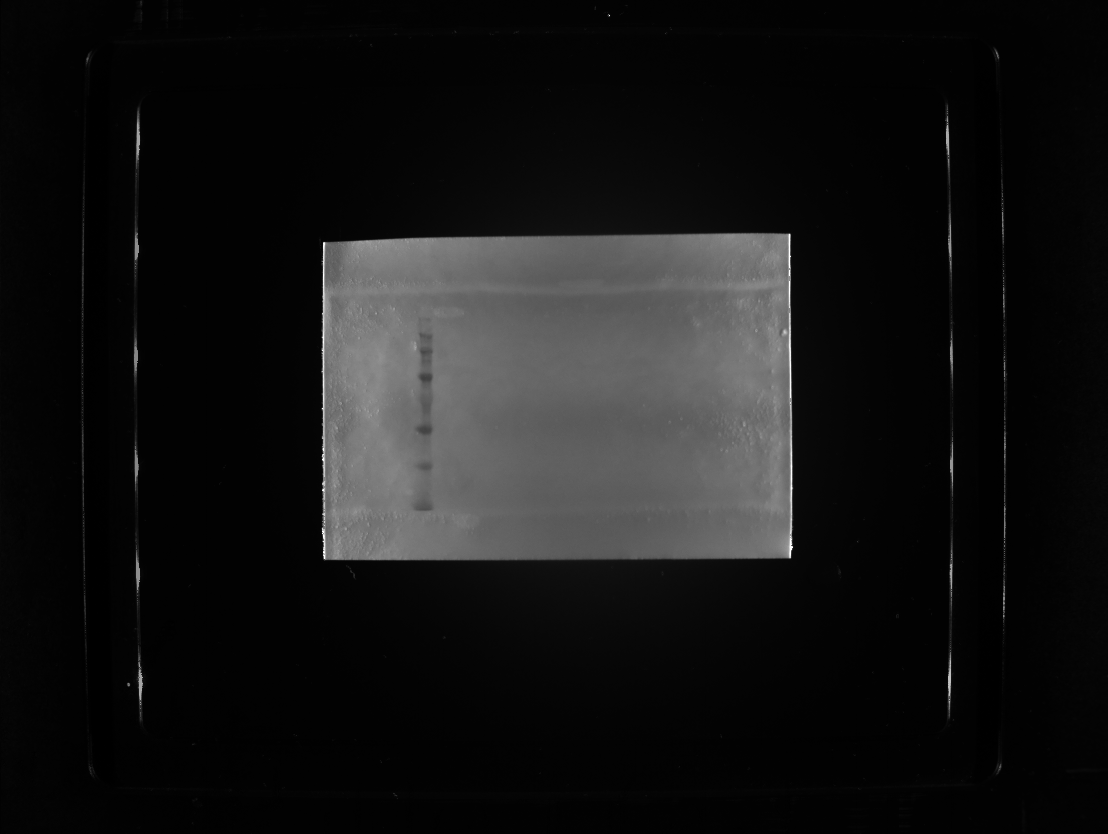

Supplement: Supplementary file 6 — Source data Fig. 5 [file 44318_2025_545_MOESM6_ESM.zip › Fig 5 SD/5A/ADPr/2024-0819-113756.tif]

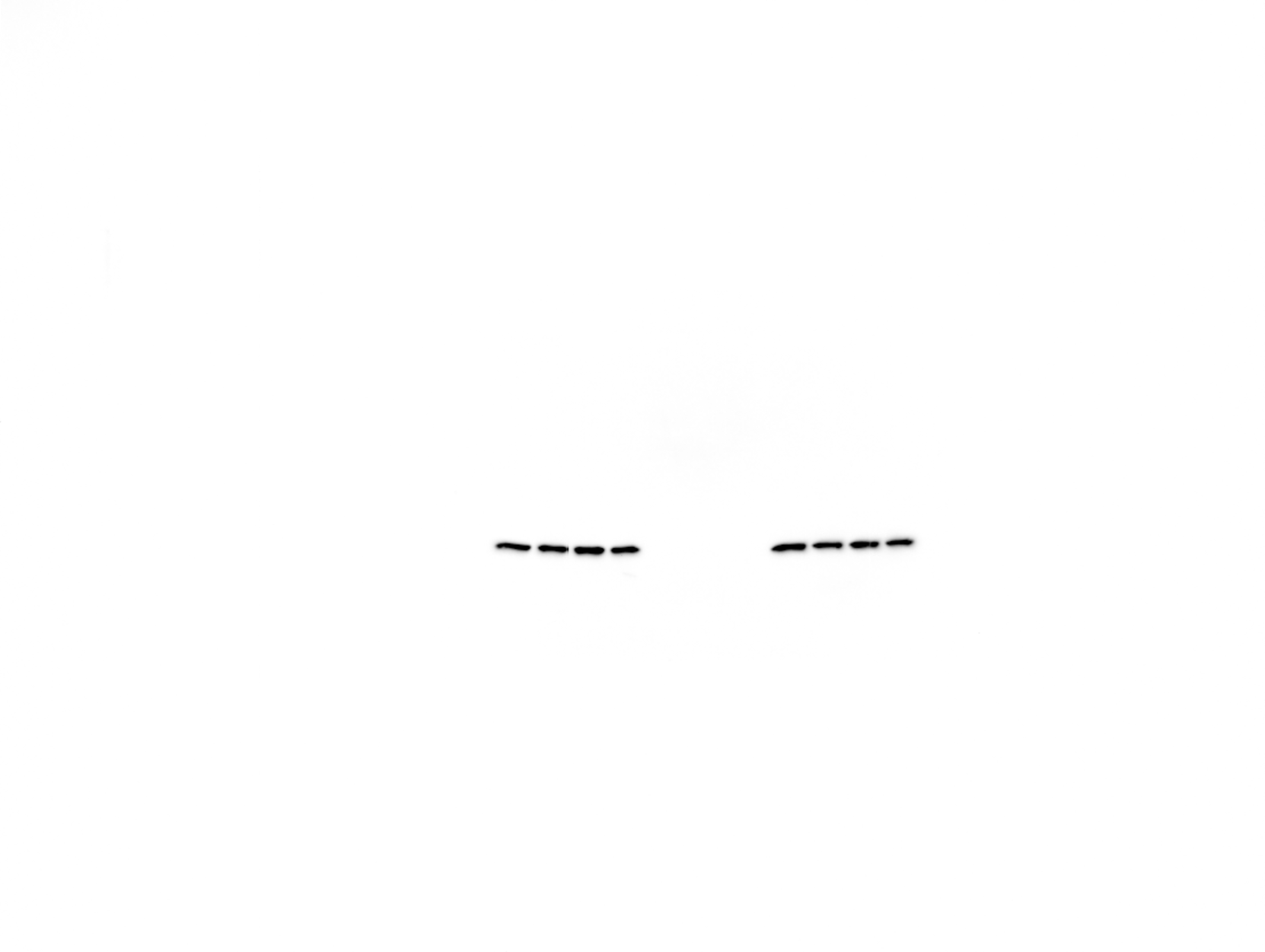

Supplement: Supplementary file 6 — Source data Fig. 5 [file 44318_2025_545_MOESM6_ESM.zip › Fig 5 SD/5A/Tub/2024-0906-115833_pub.tif]

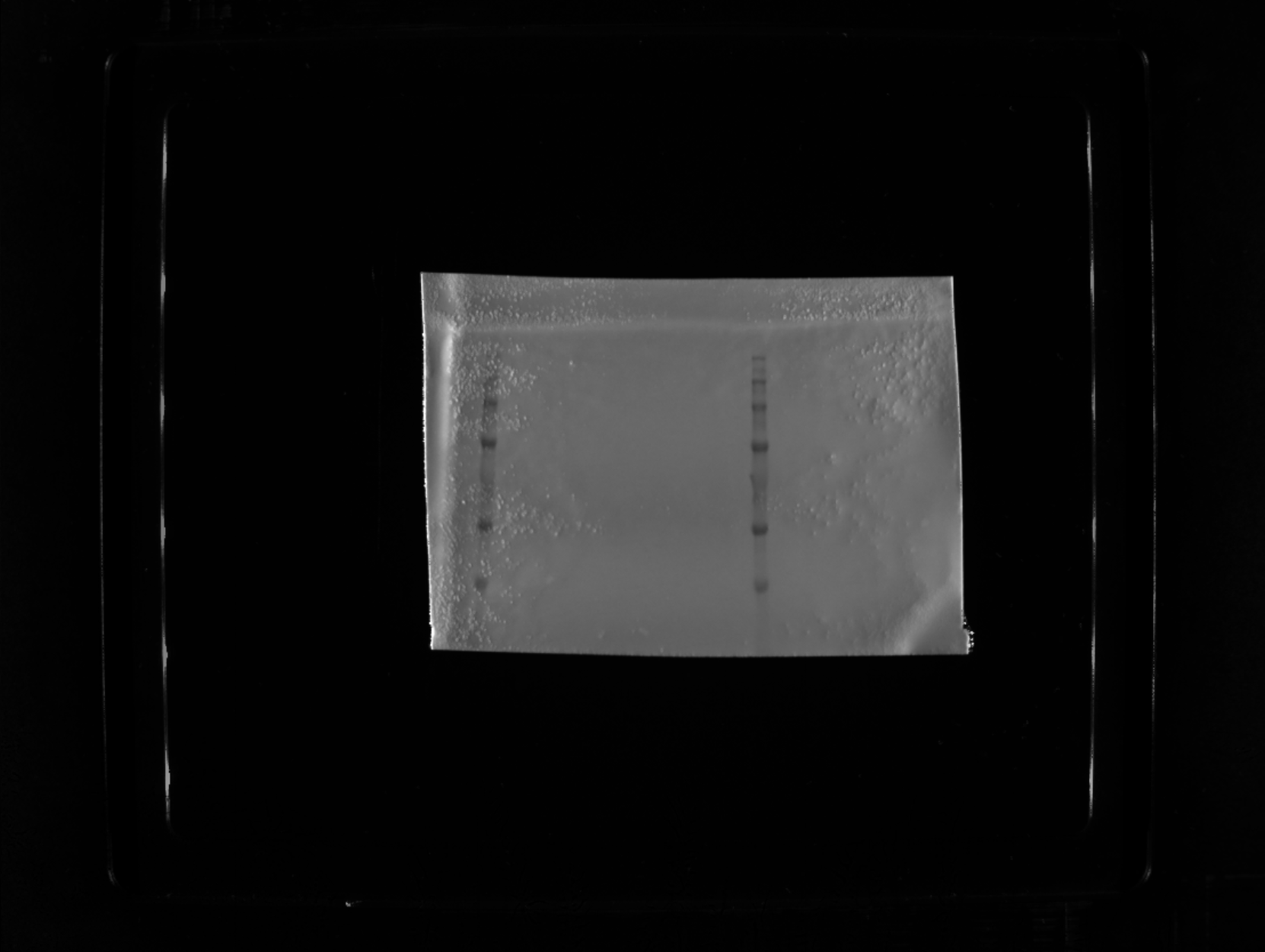

Supplement: Supplementary file 6 — Source data Fig. 5 [file 44318_2025_545_MOESM6_ESM.zip › Fig 5 SD/5A/Tub/2024-0906-115831_pub.tif]

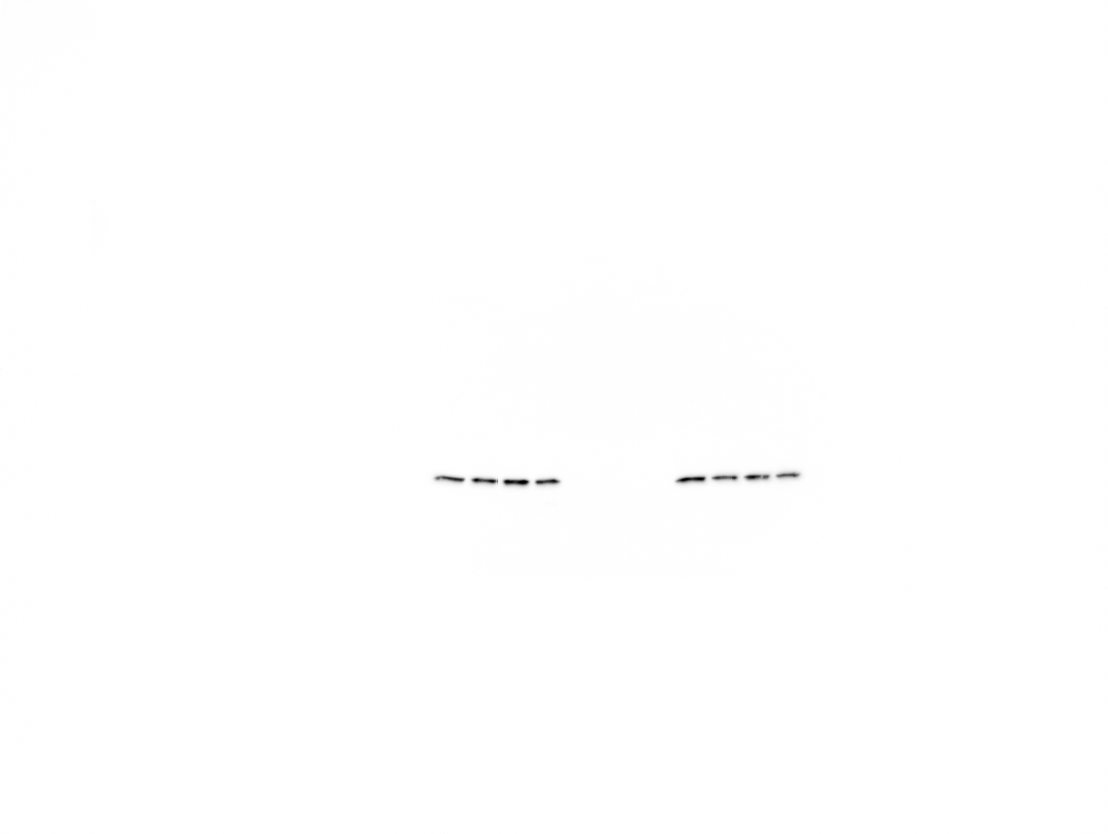

Supplement: Supplementary file 6 — Source data Fig. 5 [file 44318_2025_545_MOESM6_ESM.zip › Fig 5 SD/5A/Tub/2024-0906-115833.tif]

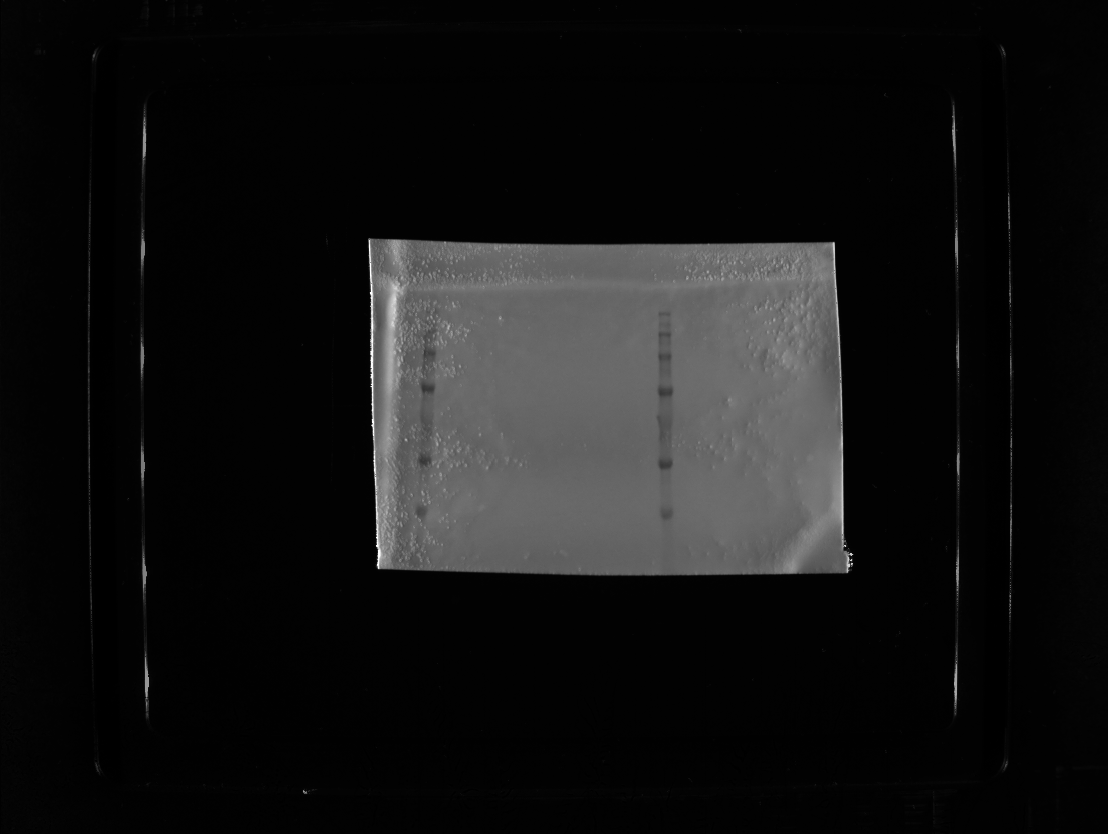

Supplement: Supplementary file 6 — Source data Fig. 5 [file 44318_2025_545_MOESM6_ESM.zip › Fig 5 SD/5A/Tub/2024-0906-115831.tif]

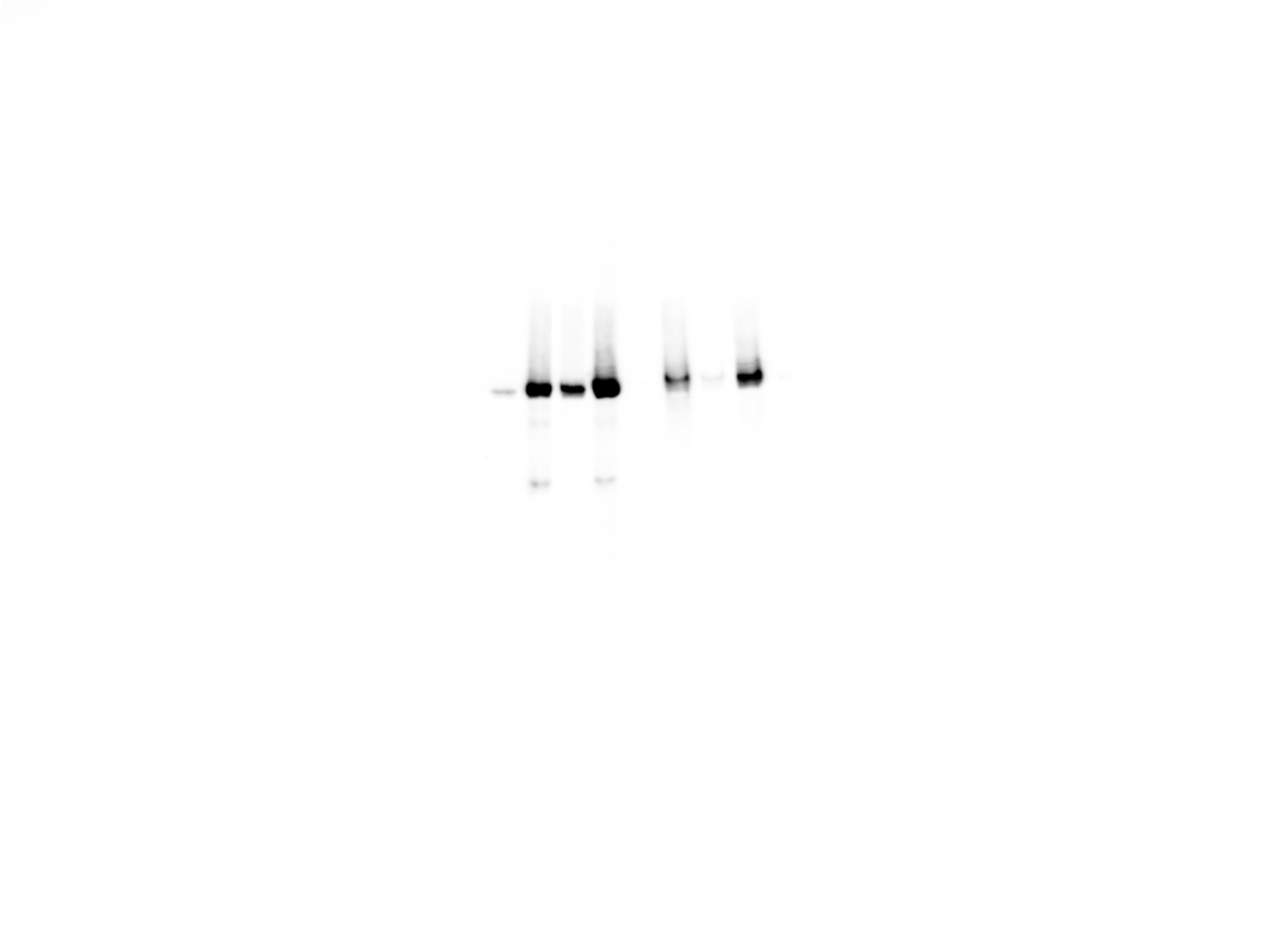

Supplement: Supplementary file 6 — Source data Fig. 5 [file 44318_2025_545_MOESM6_ESM.zip › Fig 5 SD/5A/GFP/2024-0819-114112_pub.tif]

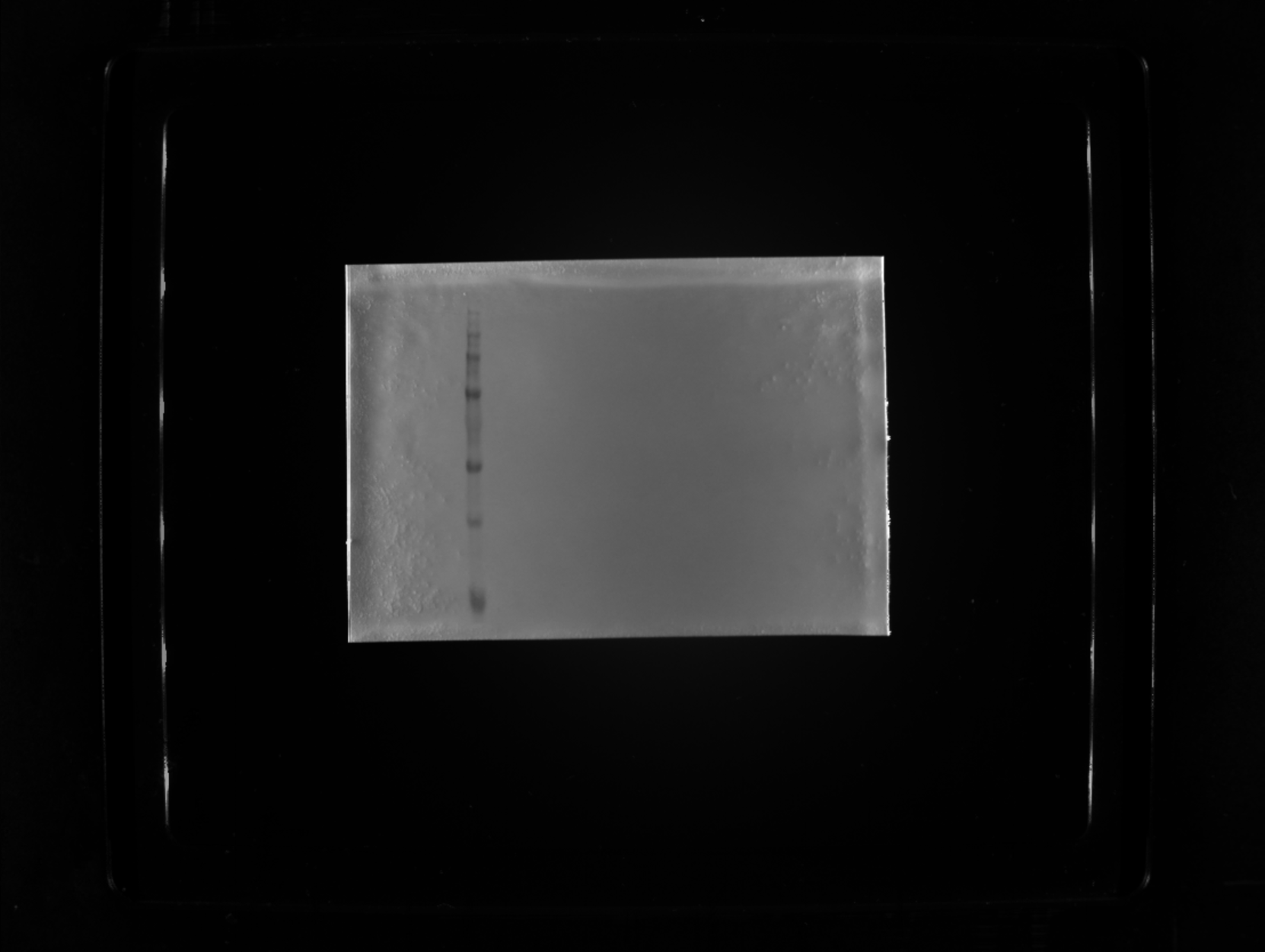

Supplement: Supplementary file 6 — Source data Fig. 5 [file 44318_2025_545_MOESM6_ESM.zip › Fig 5 SD/5A/GFP/2024-0819-114111_pub.tif]

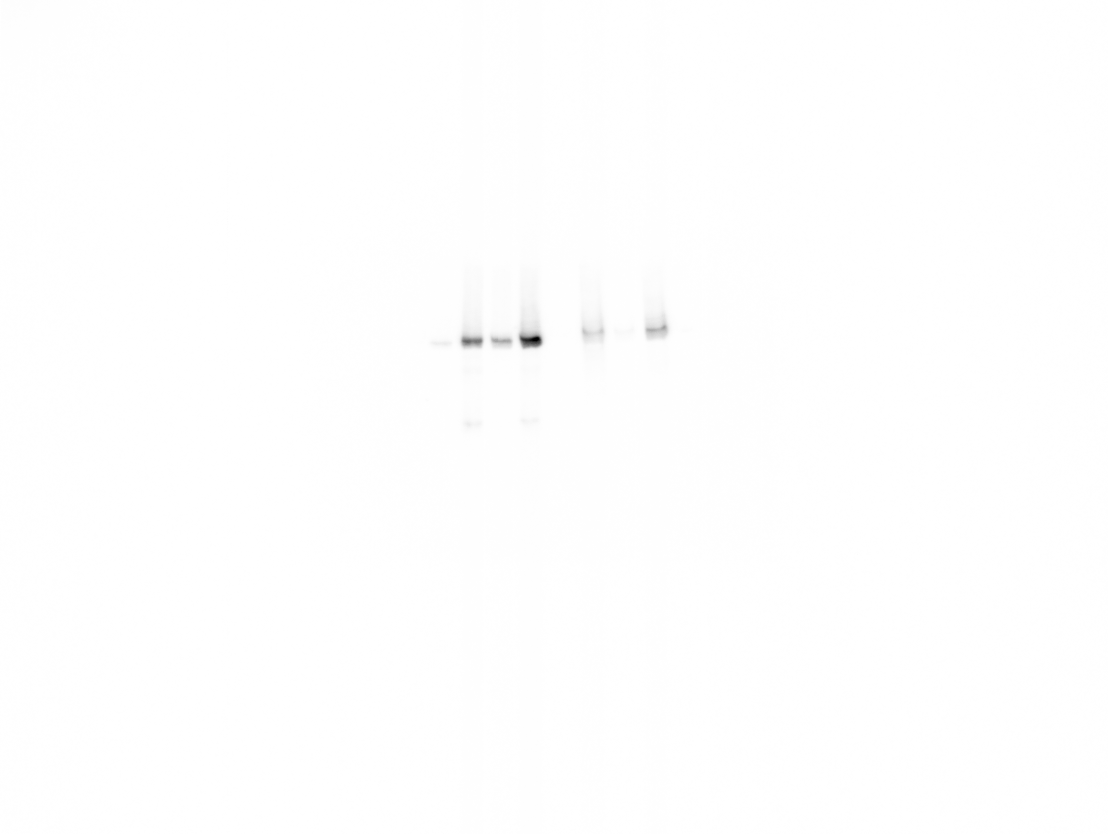

Supplement: Supplementary file 6 — Source data Fig. 5 [file 44318_2025_545_MOESM6_ESM.zip › Fig 5 SD/5A/GFP/2024-0819-114112.tif]

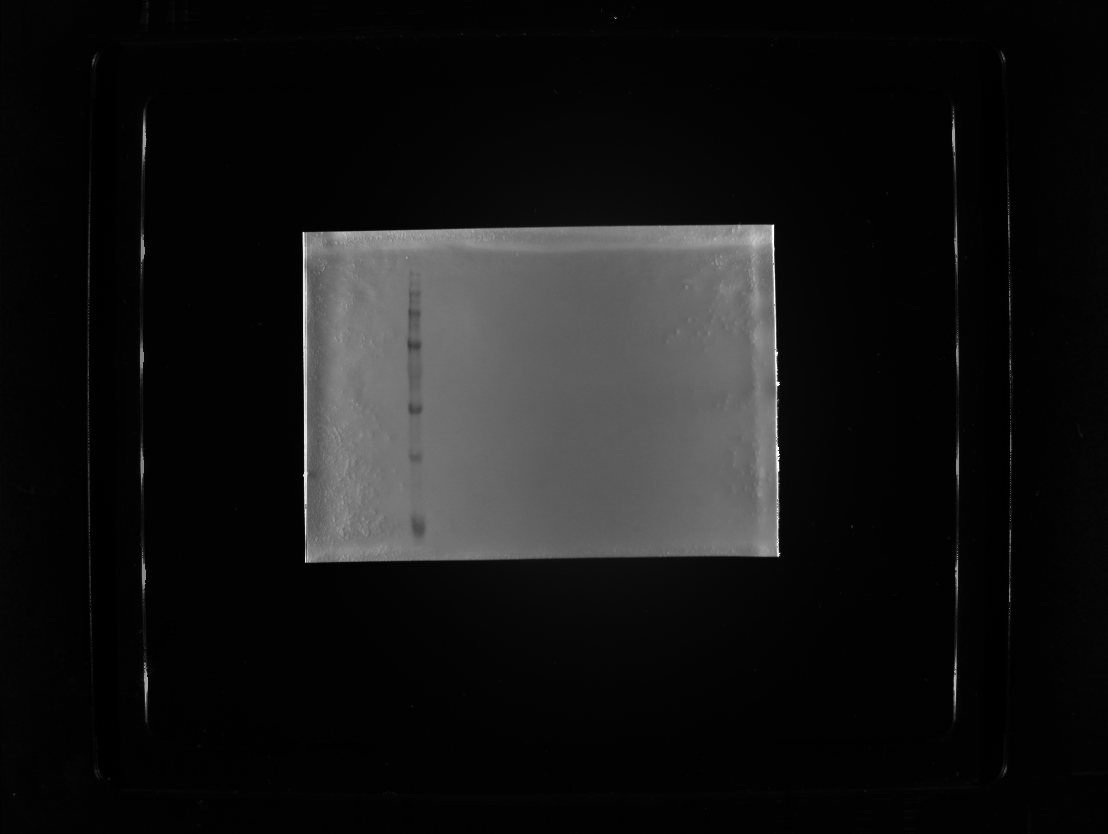

Supplement: Supplementary file 6 — Source data Fig. 5 [file 44318_2025_545_MOESM6_ESM.zip › Fig 5 SD/5A/GFP/2024-0819-114111.tif]

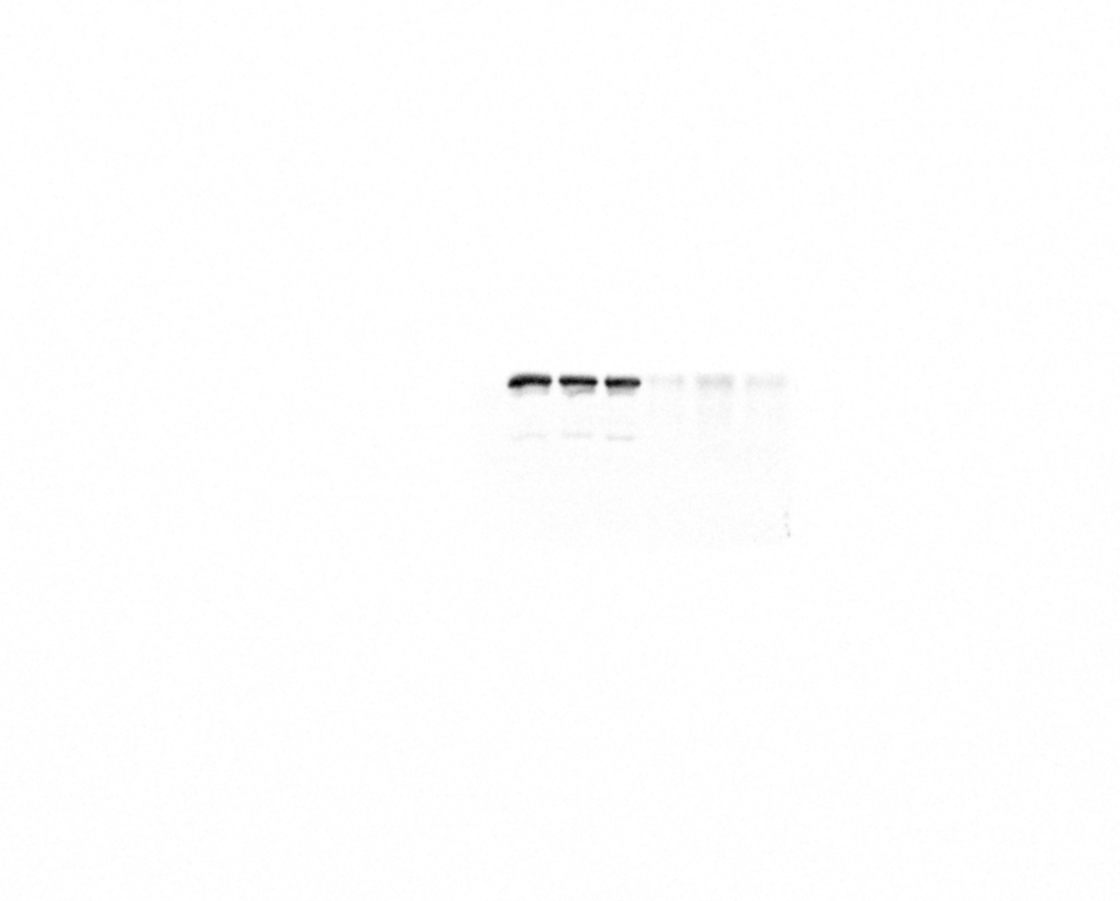

Supplement: Supplementary file 6 — Source data Fig. 5 [file 44318_2025_545_MOESM6_ESM.zip › Fig 5 SD/5E/Tubulin/25.04.02_10.24.31.tif]

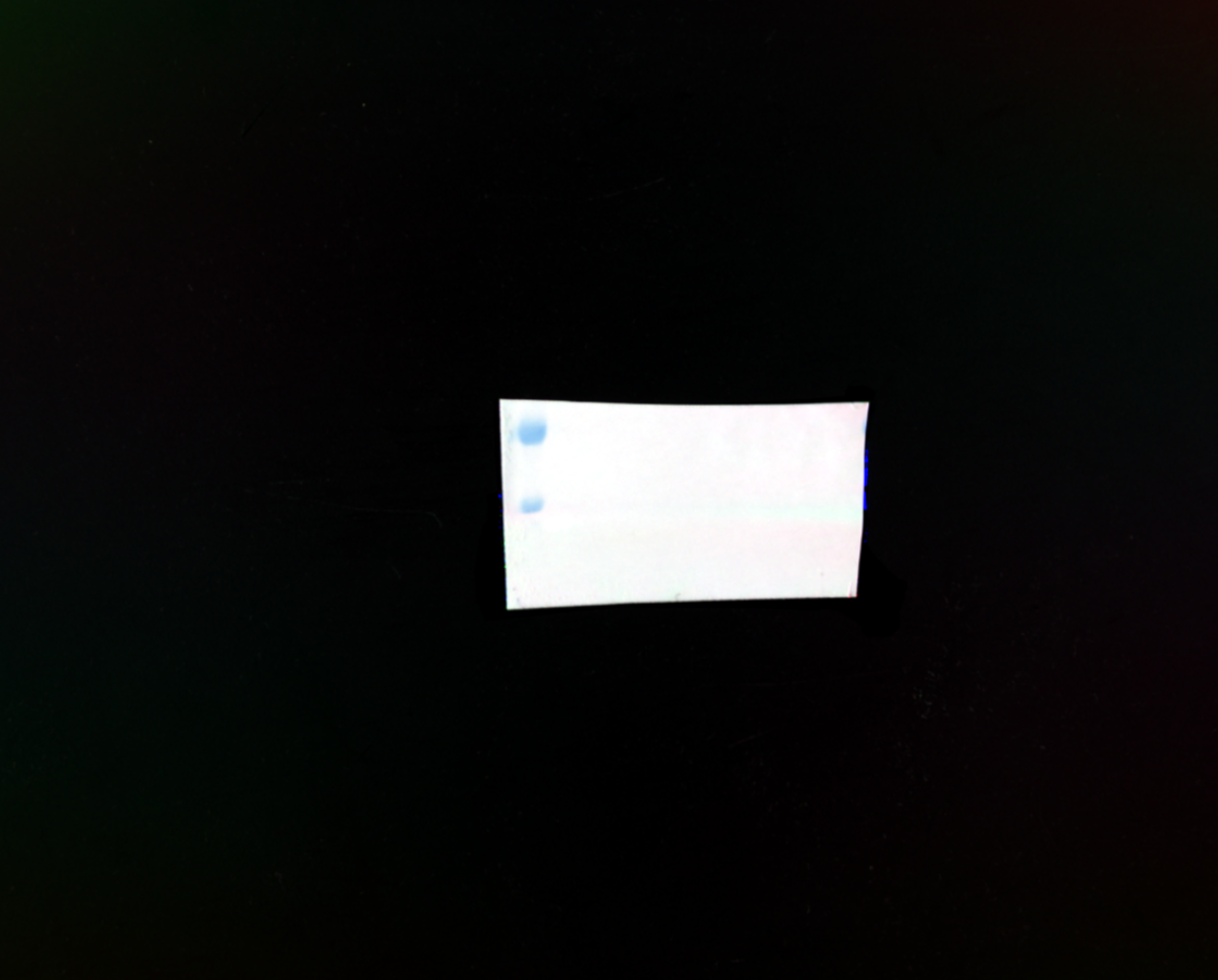

Supplement: Supplementary file 6 — Source data Fig. 5 [file 44318_2025_545_MOESM6_ESM.zip › Fig 5 SD/5E/Tubulin/25.04.02_10.24.31_marker_PUB_300.tif]

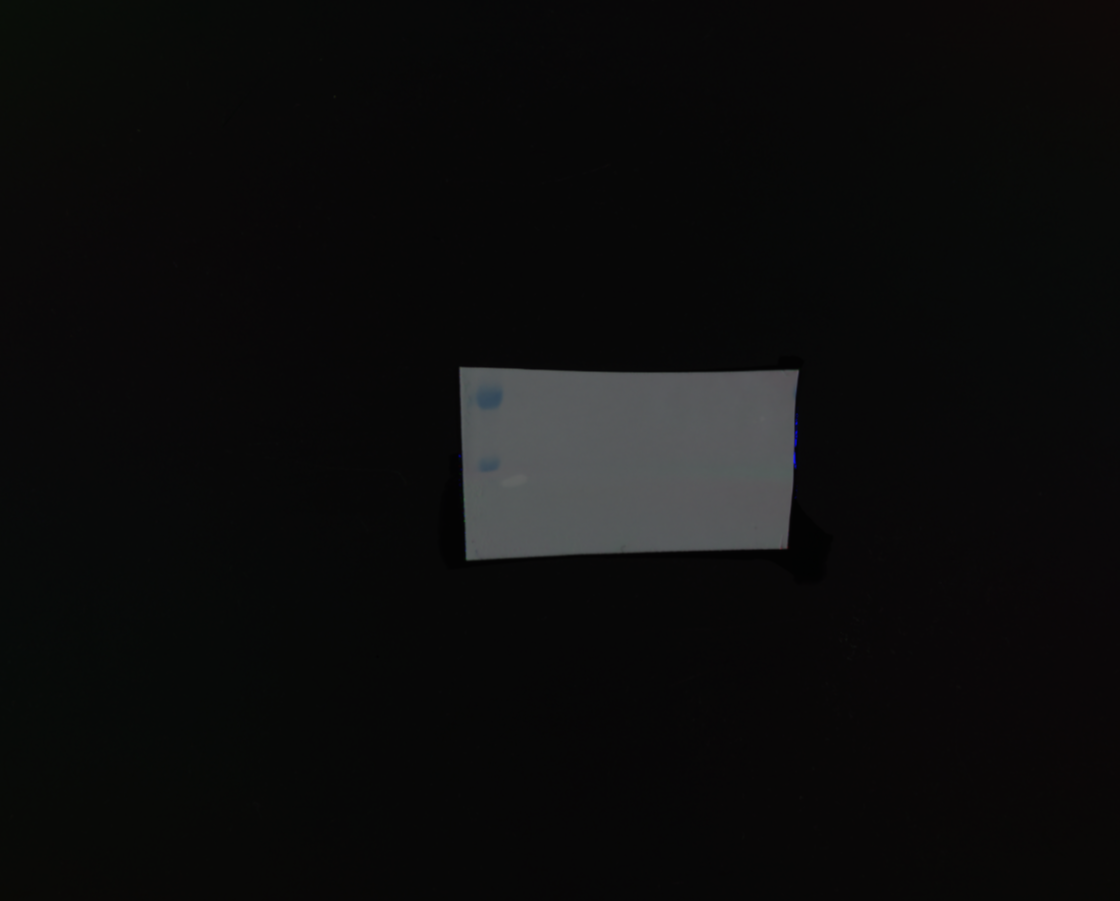

Supplement: Supplementary file 6 — Source data Fig. 5 [file 44318_2025_545_MOESM6_ESM.zip › Fig 5 SD/5E/Tubulin/25.04.02_10.24.31_marker.tif]

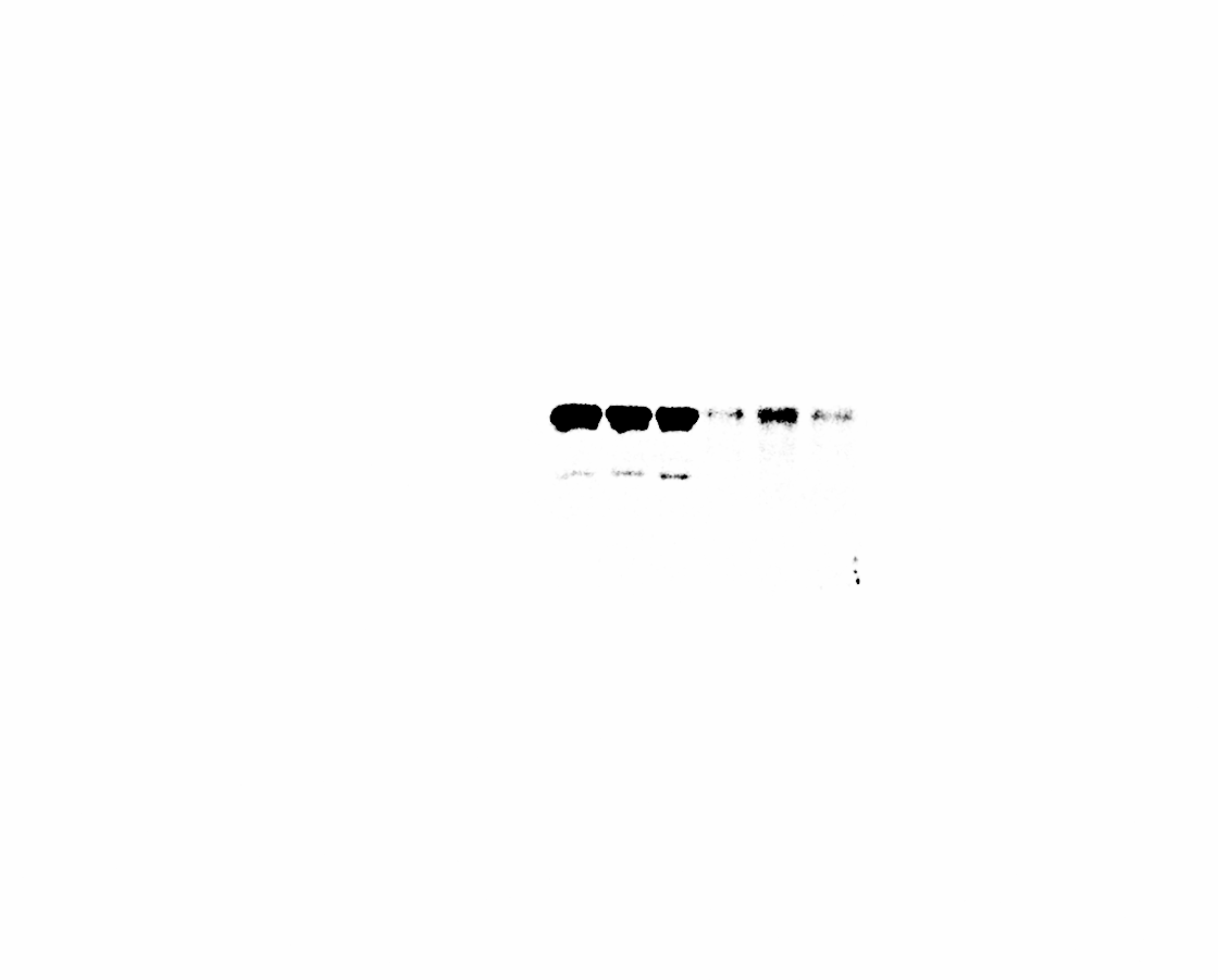

Supplement: Supplementary file 6 — Source data Fig. 5 [file 44318_2025_545_MOESM6_ESM.zip › Fig 5 SD/5E/Tubulin/25.04.02_10.24.31_PUB_300.tif]

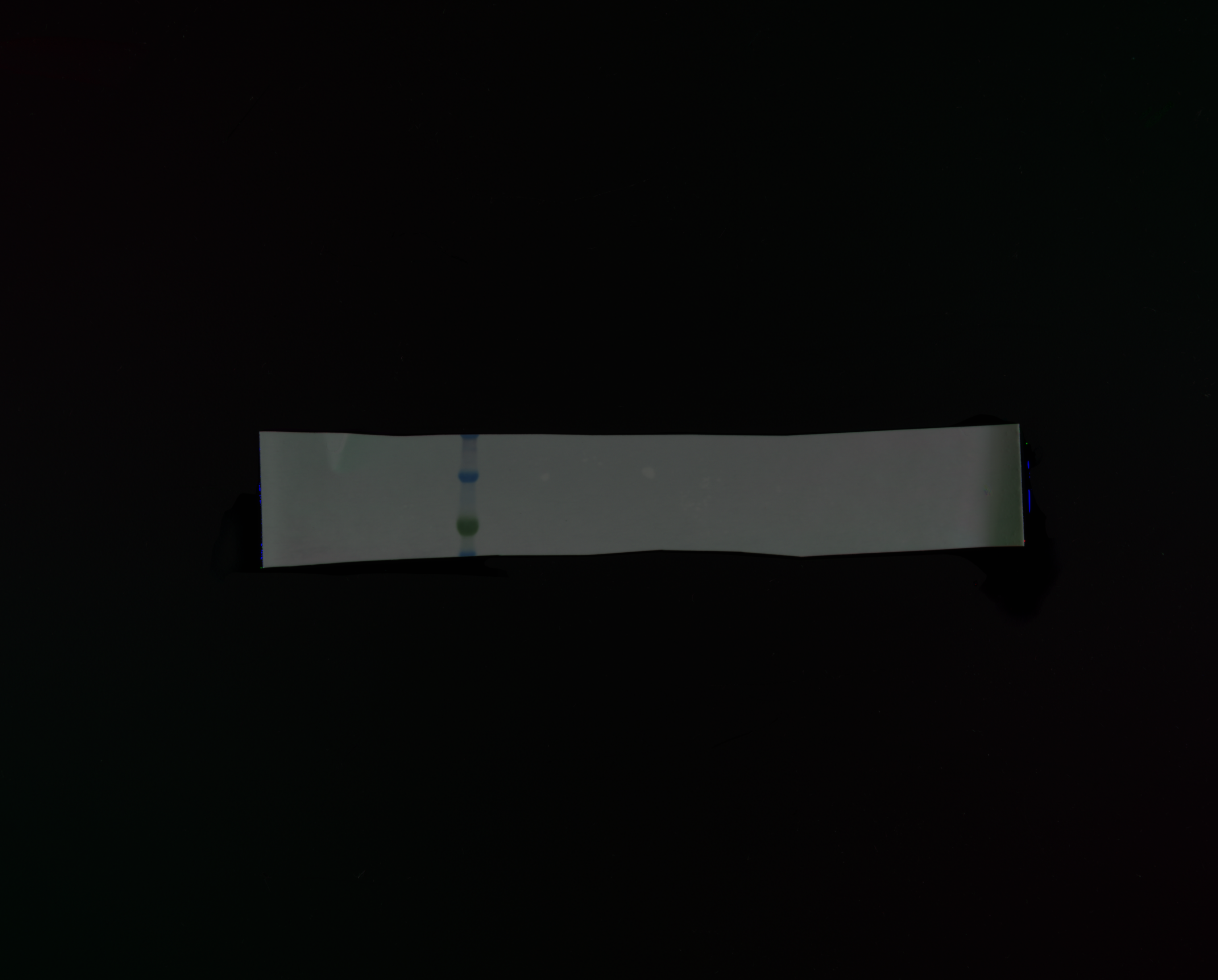

Supplement: Supplementary file 6 — Source data Fig. 5 [file 44318_2025_545_MOESM6_ESM.zip › Fig 5 SD/5E/GAPDH/25.04.17_14.51.04_S9_marker.tif]

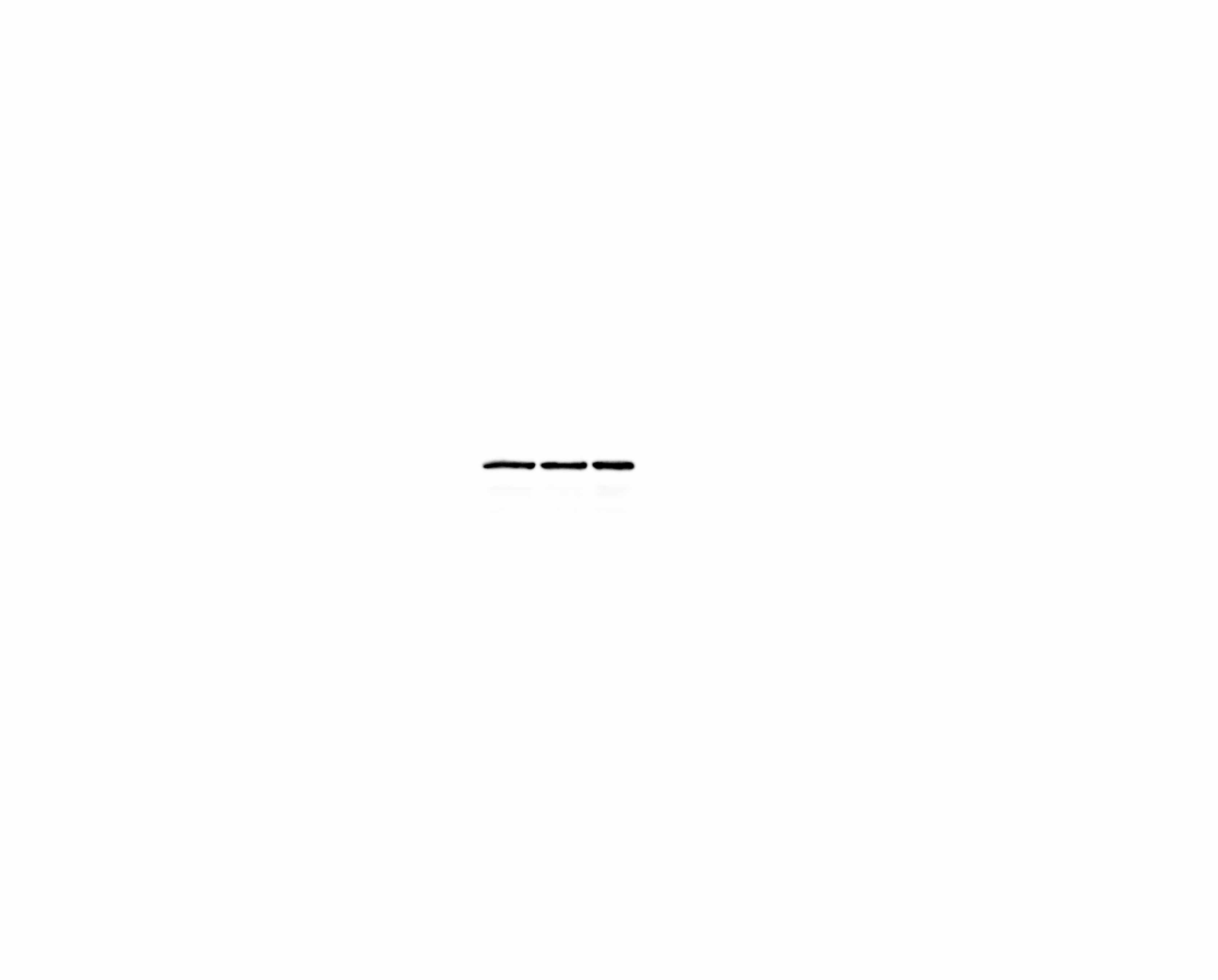

Supplement: Supplementary file 6 — Source data Fig. 5 [file 44318_2025_545_MOESM6_ESM.zip › Fig 5 SD/5E/GAPDH/25.04.17_14.51.04_S9_F01_PUB_300.tif]

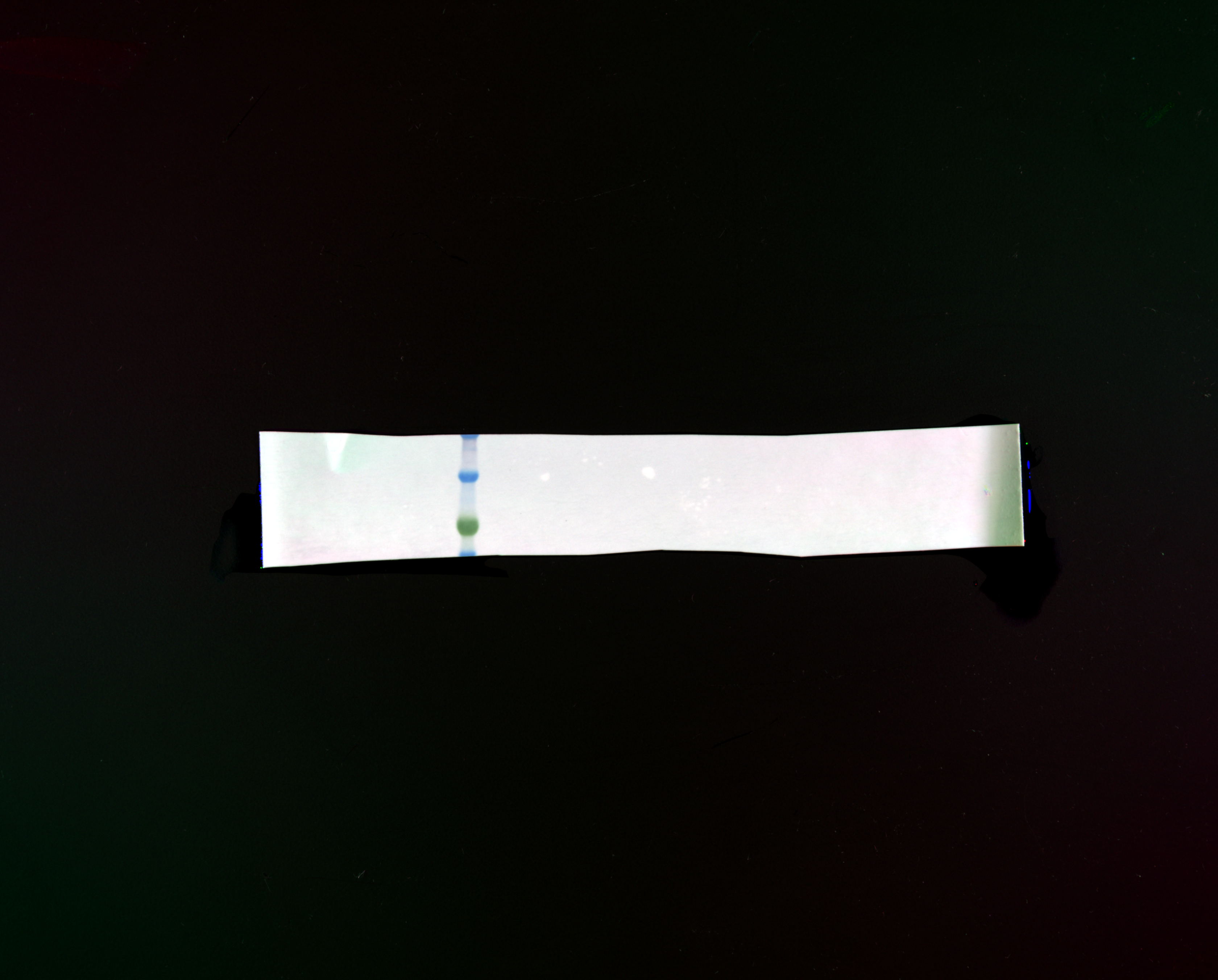

Supplement: Supplementary file 6 — Source data Fig. 5 [file 44318_2025_545_MOESM6_ESM.zip › Fig 5 SD/5E/GAPDH/25.04.17_14.51.04_S9_marker_PUB_300.tif]

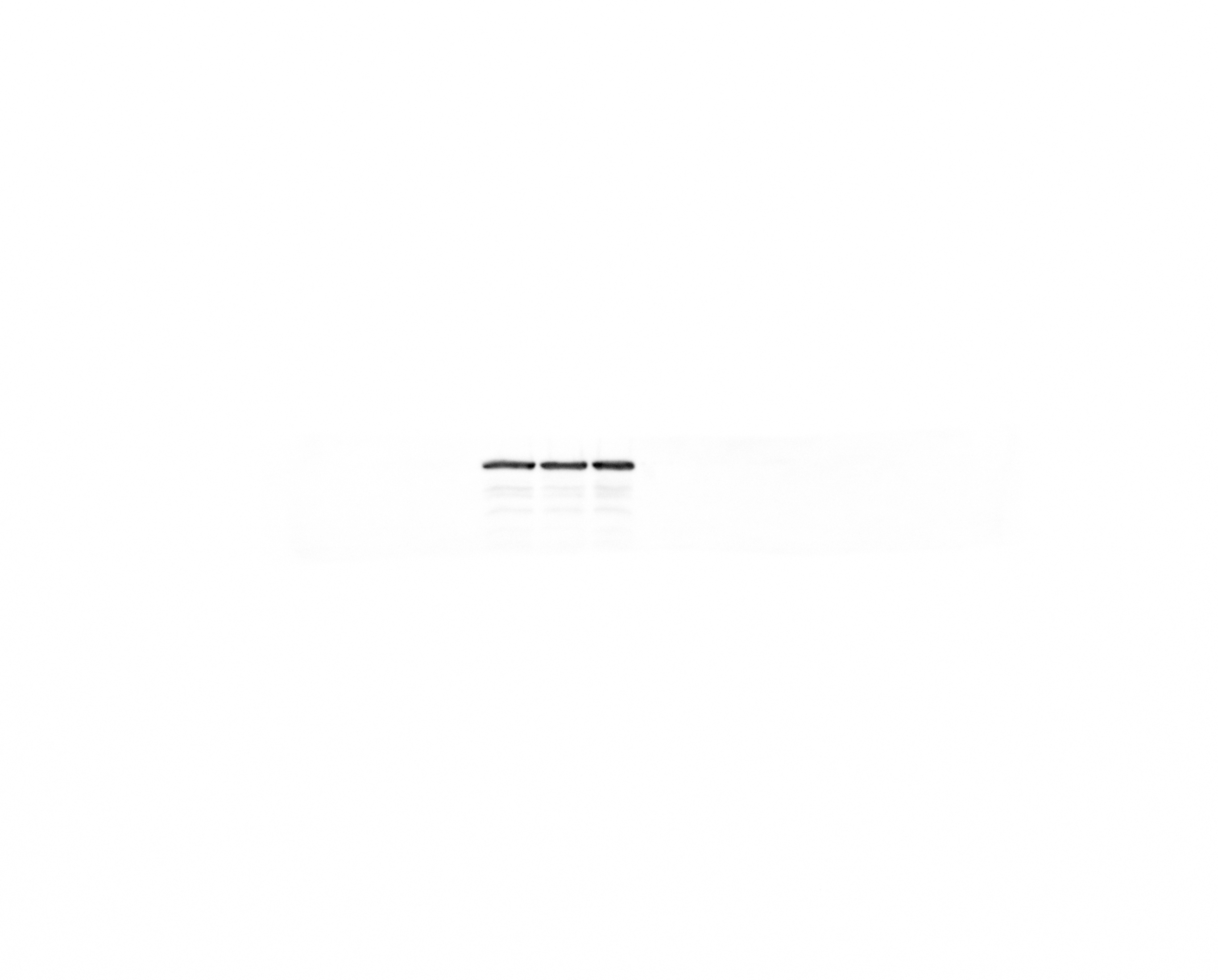

Supplement: Supplementary file 6 — Source data Fig. 5 [file 44318_2025_545_MOESM6_ESM.zip › Fig 5 SD/5E/GAPDH/25.04.17_14.51.04_S9_F01.tif]

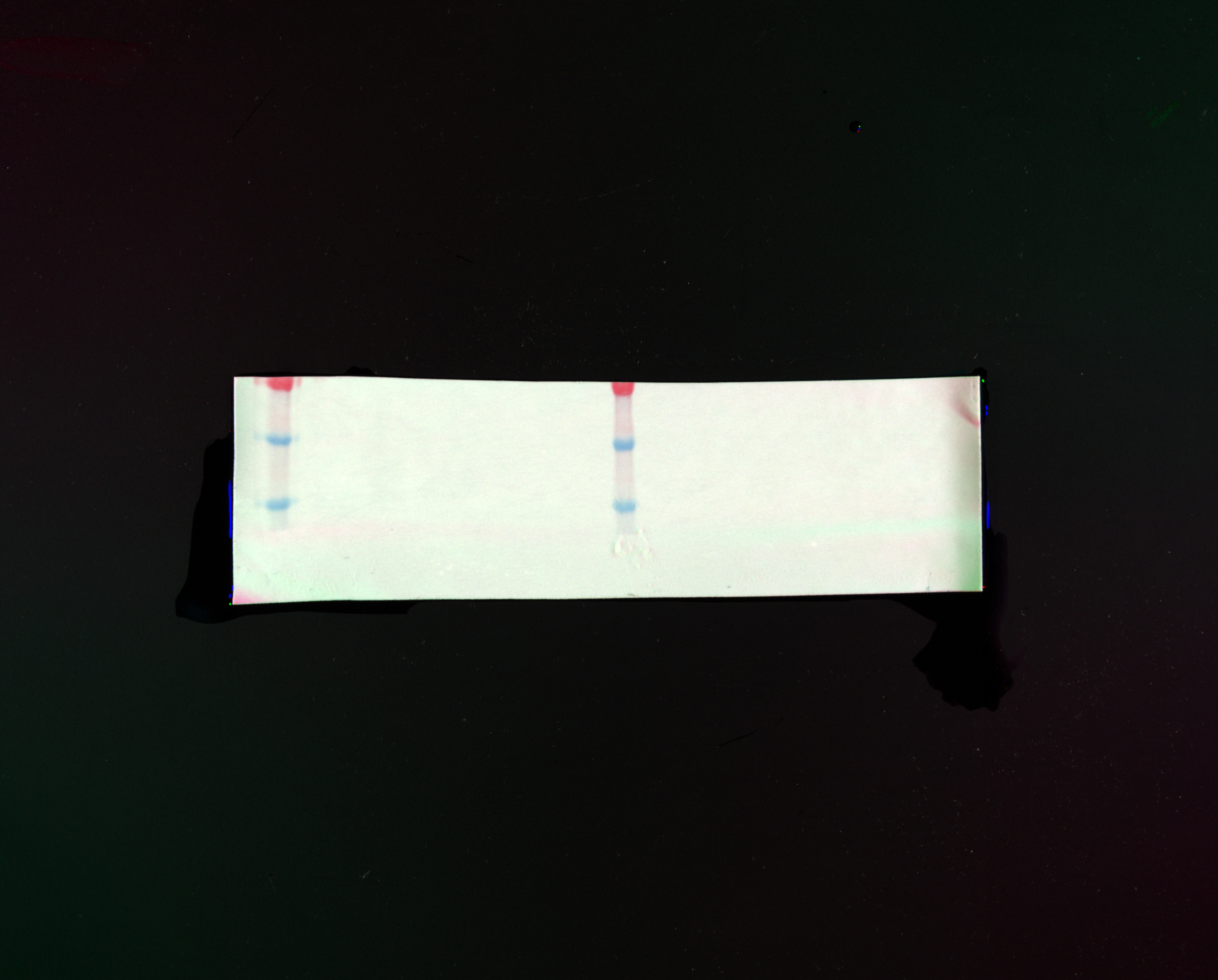

Supplement: Supplementary file 6 — Source data Fig. 5 [file 44318_2025_545_MOESM6_ESM.zip › Fig 5 SD/5E/C-FOS/25.04.17_14.55.16_S10_marker.tif]

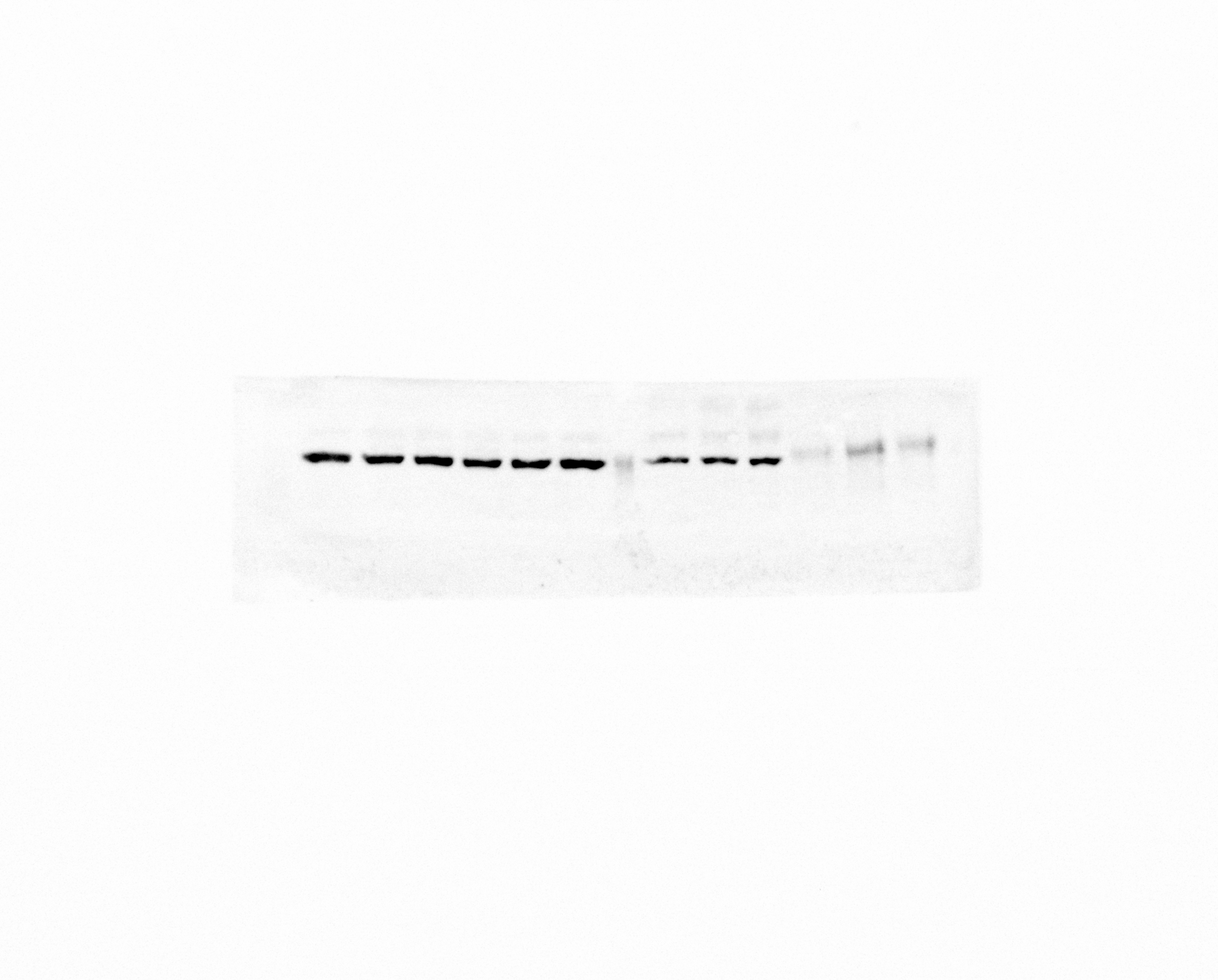

Supplement: Supplementary file 6 — Source data Fig. 5 [file 44318_2025_545_MOESM6_ESM.zip › Fig 5 SD/5E/C-FOS/25.04.17_14.55.16_S10_F01_PUB_300.tif]

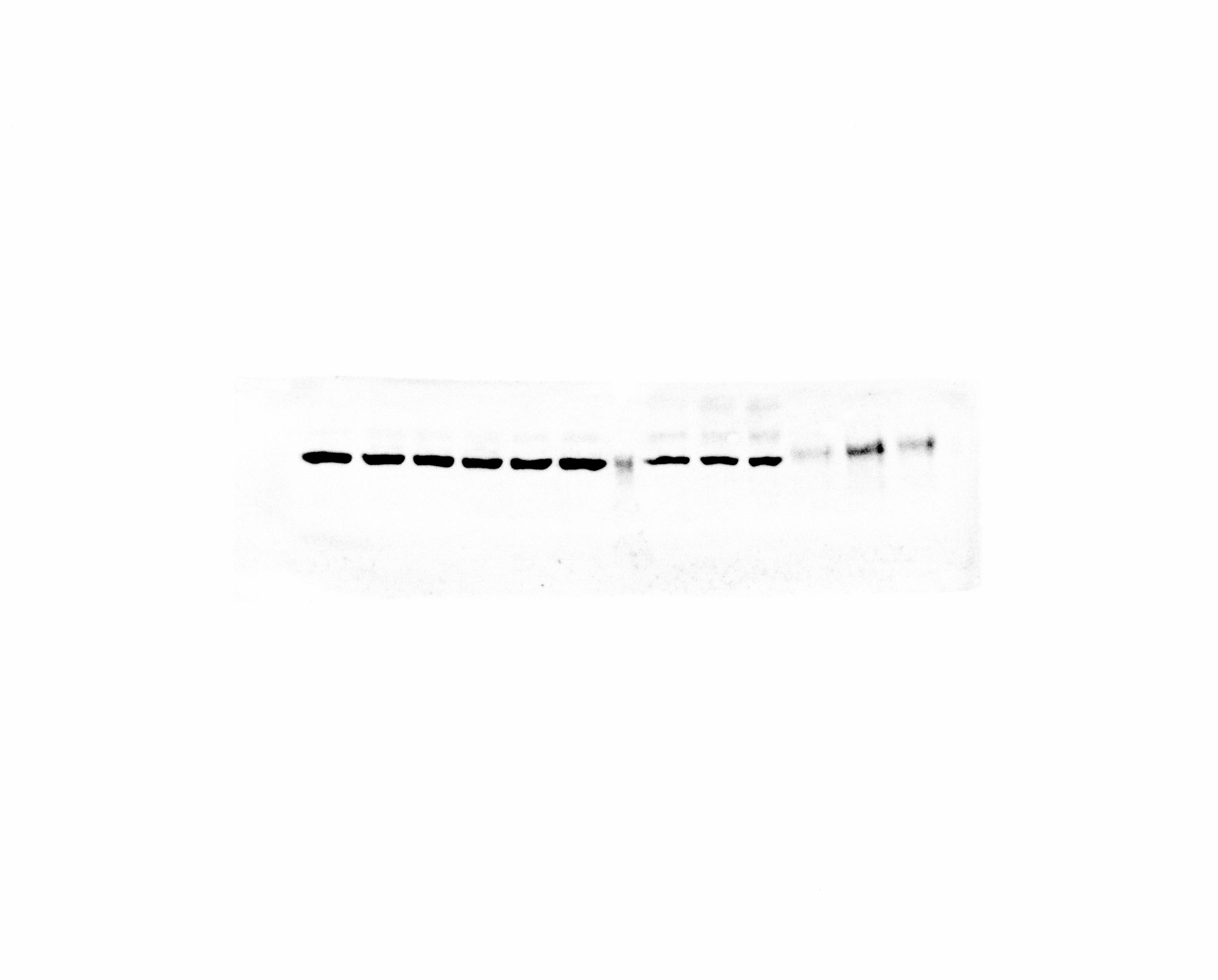

Supplement: Supplementary file 6 — Source data Fig. 5 [file 44318_2025_545_MOESM6_ESM.zip › Fig 5 SD/5E/C-FOS/25.04.17_14.55.16_S10_F02_PUB_300.tif]

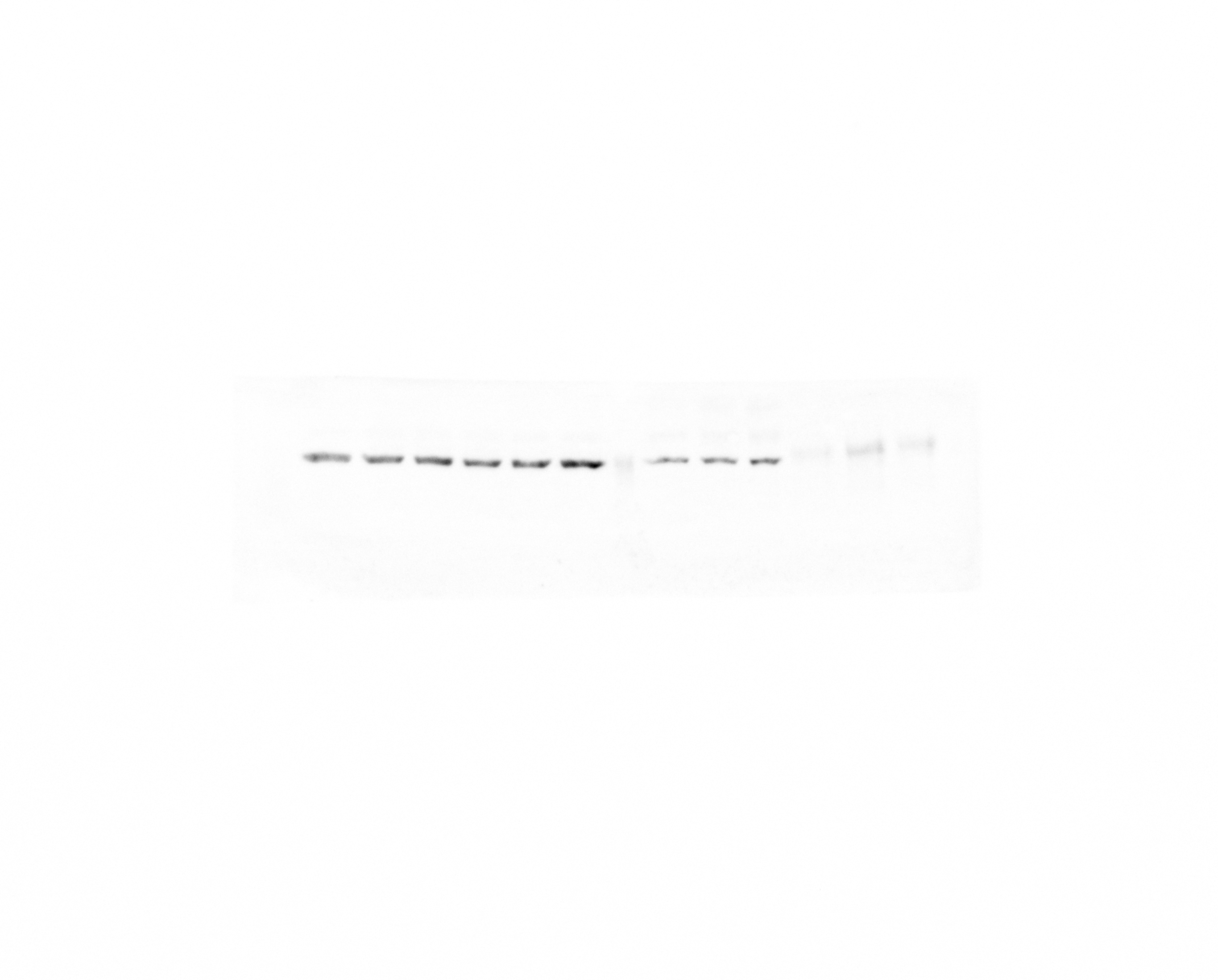

Supplement: Supplementary file 6 — Source data Fig. 5 [file 44318_2025_545_MOESM6_ESM.zip › Fig 5 SD/5E/C-FOS/25.04.17_14.55.16_S10_F01.tif]

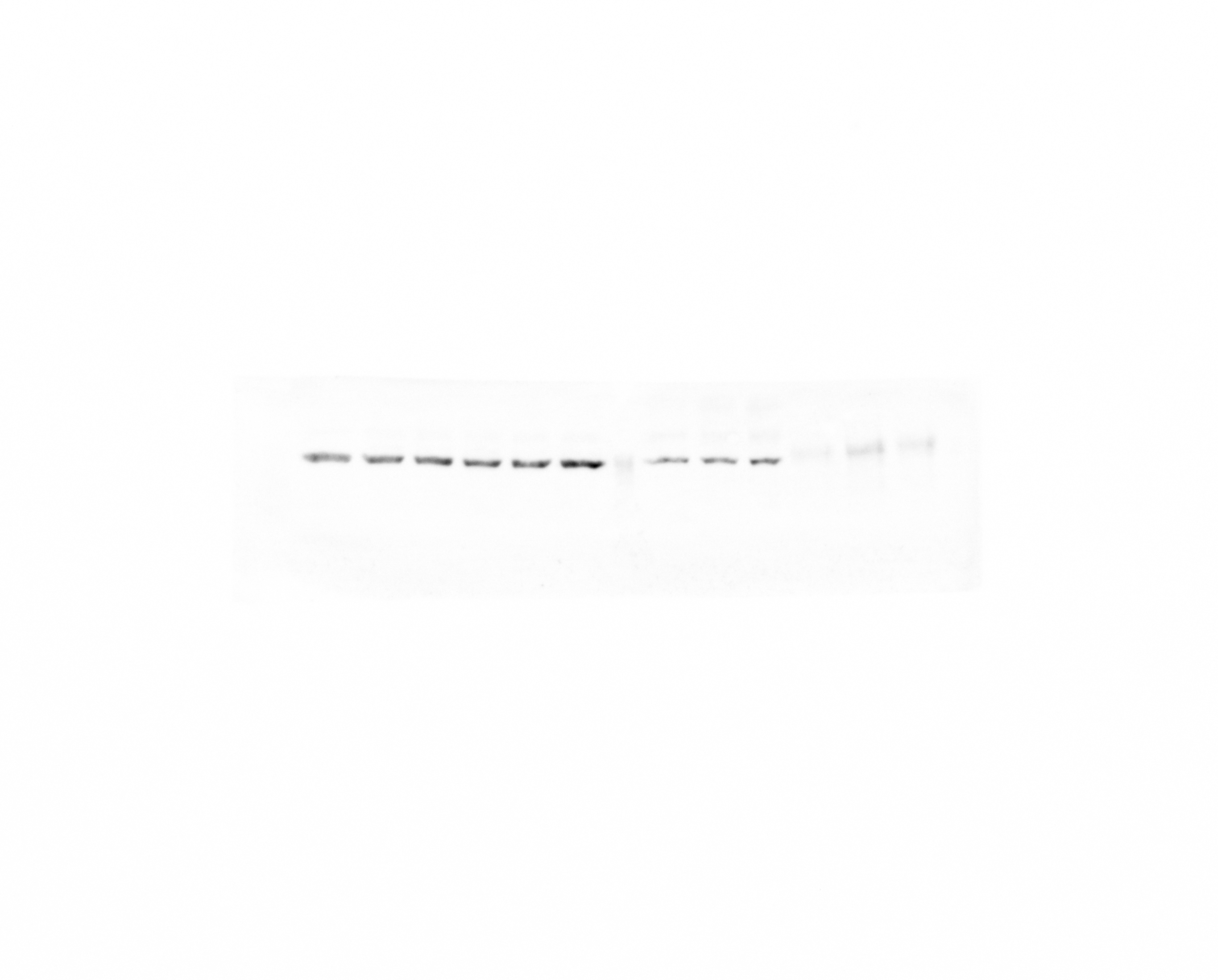

Supplement: Supplementary file 6 — Source data Fig. 5 [file 44318_2025_545_MOESM6_ESM.zip › Fig 5 SD/5E/C-FOS/25.04.17_14.55.16_S10_F02.tif]

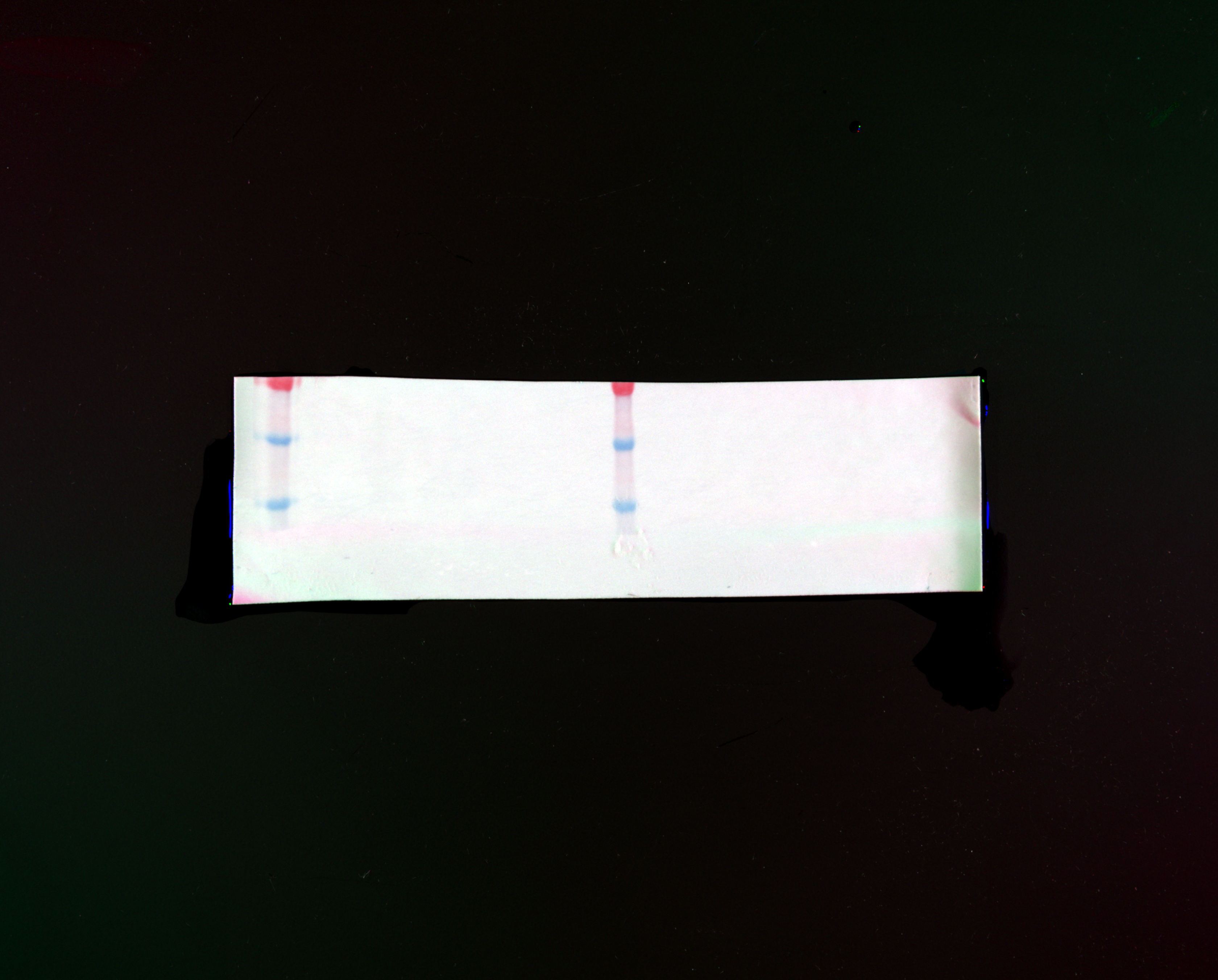

Supplement: Supplementary file 6 — Source data Fig. 5 [file 44318_2025_545_MOESM6_ESM.zip › Fig 5 SD/5E/C-FOS/25.04.17_14.55.16_S10_marker_PUB_300.tif]

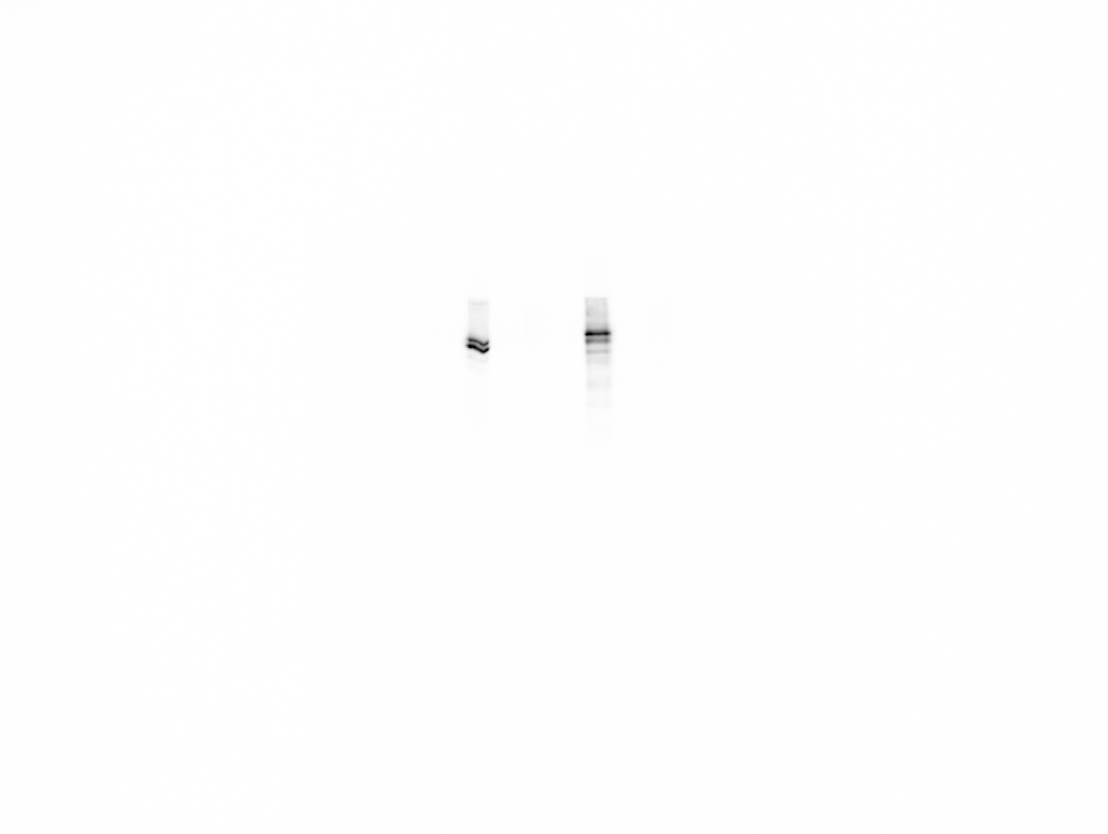

Supplement: Supplementary file 6 — Source data Fig. 5 [file 44318_2025_545_MOESM6_ESM.zip › Fig 5 SD/5B/ADPr/2024-0819-114447.tif]

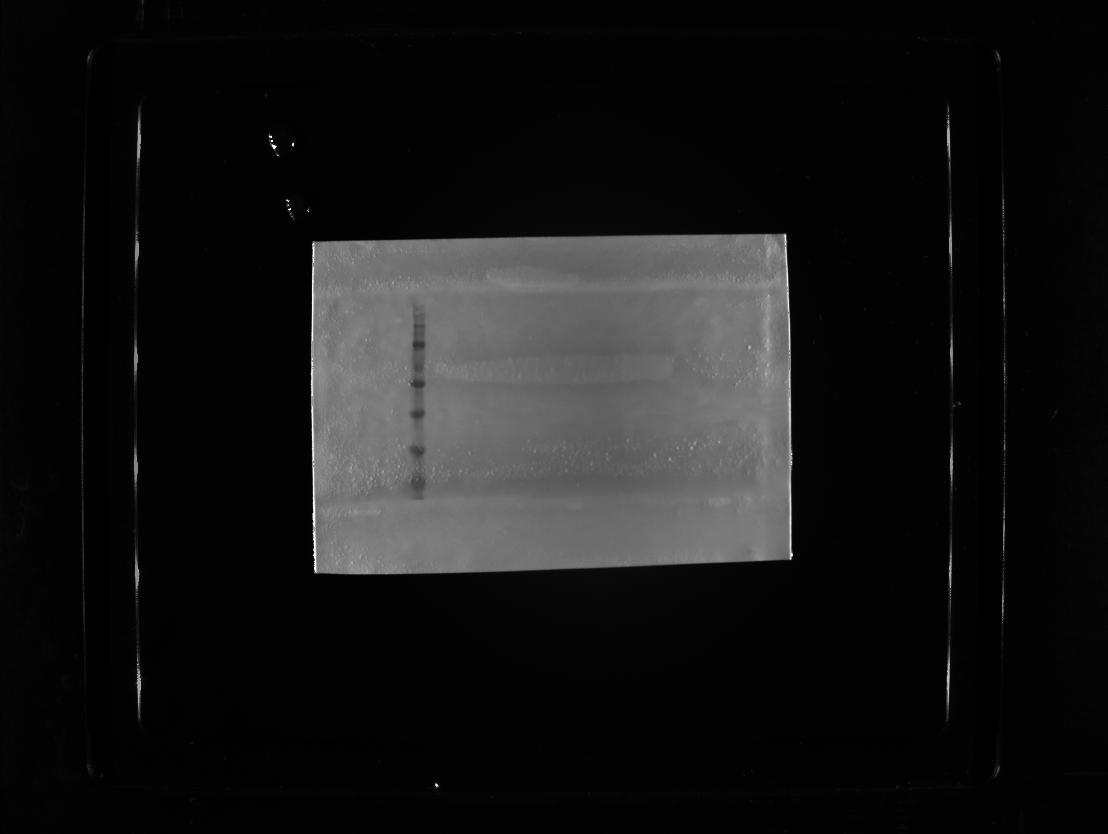

Supplement: Supplementary file 6 — Source data Fig. 5 [file 44318_2025_545_MOESM6_ESM.zip › Fig 5 SD/5B/ADPr/2024-0819-114446.tif]

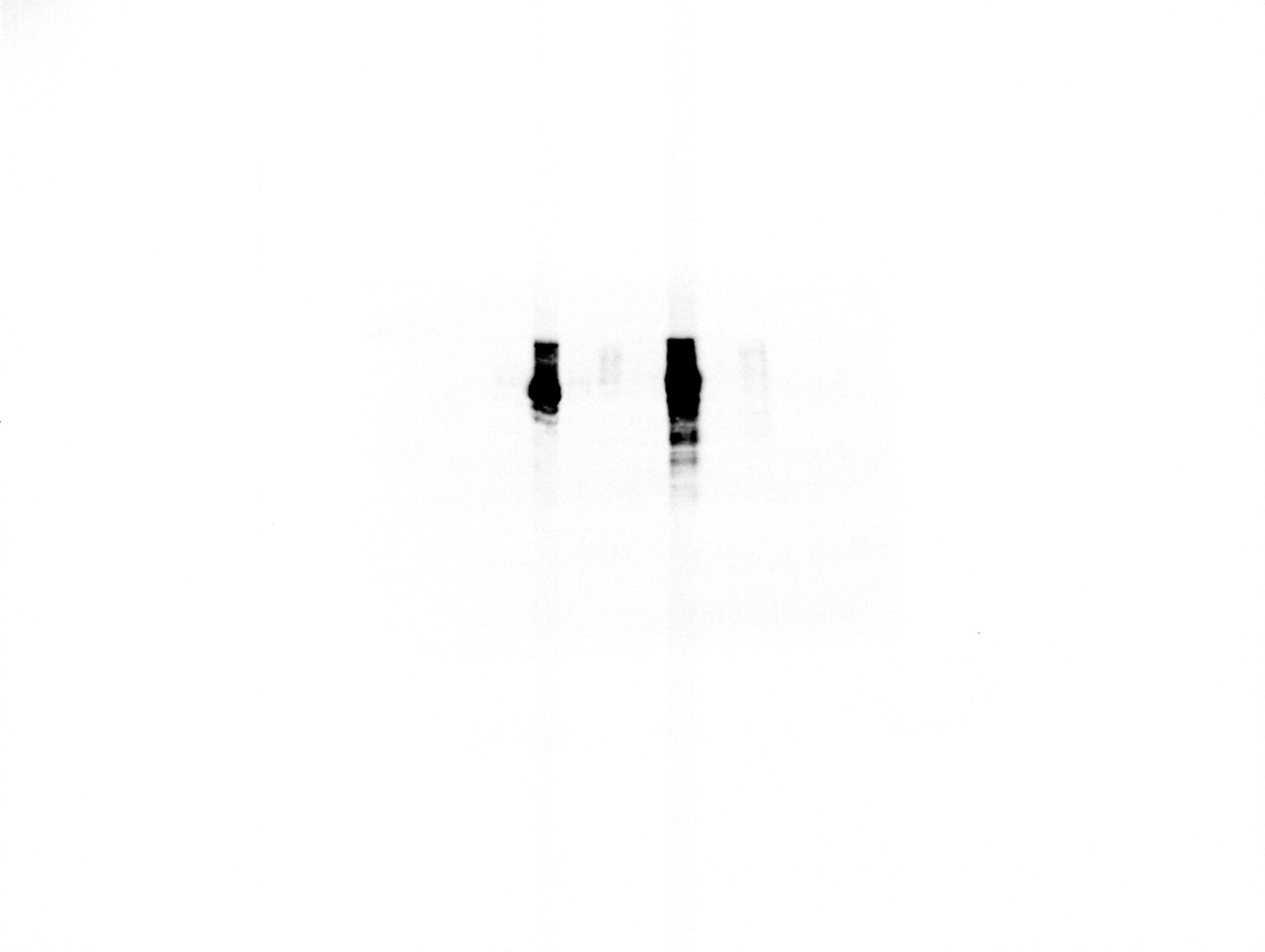

Supplement: Supplementary file 6 — Source data Fig. 5 [file 44318_2025_545_MOESM6_ESM.zip › Fig 5 SD/5B/ADPr/2024-0819-114447_pub.tif]

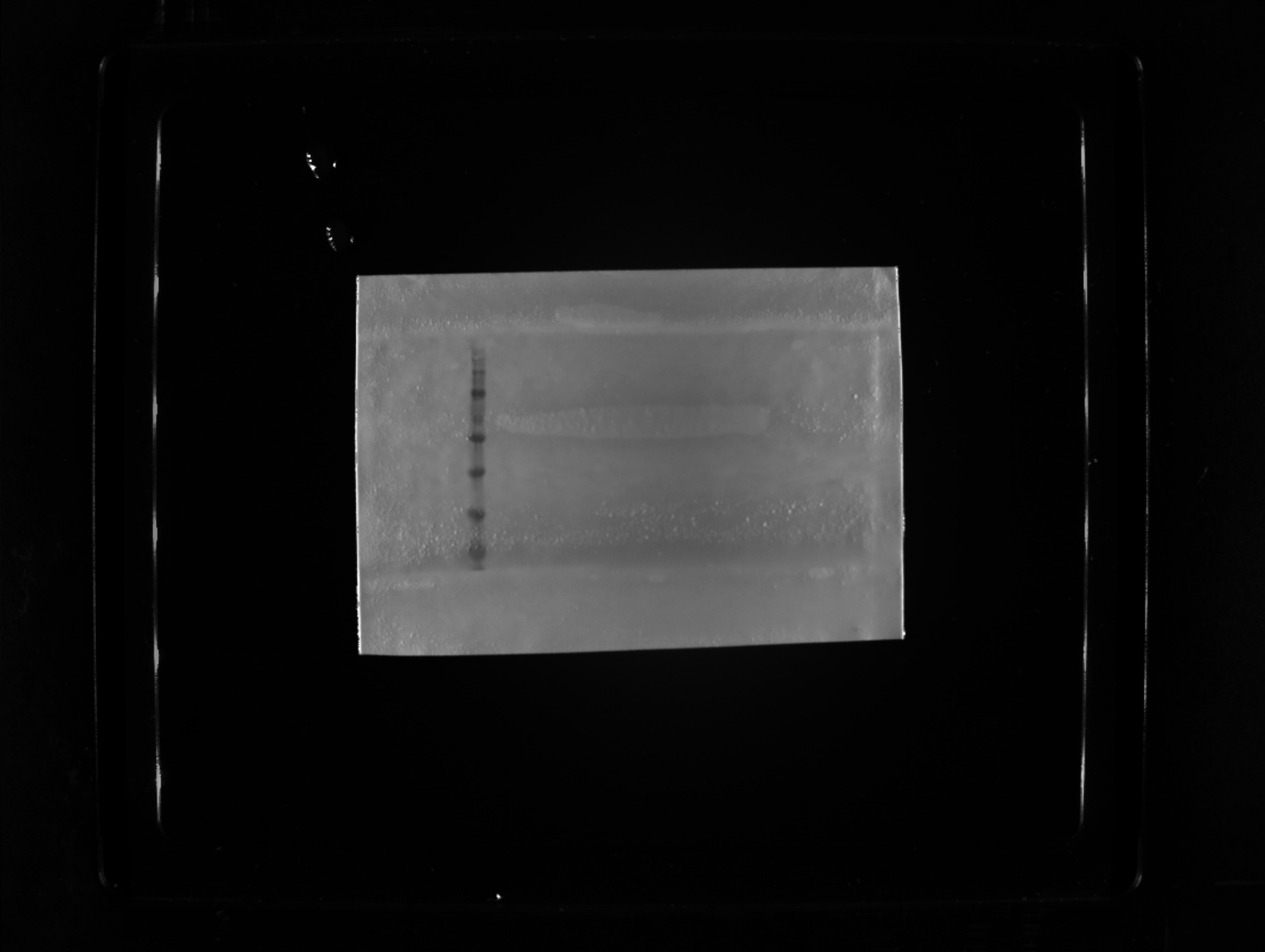

Supplement: Supplementary file 6 — Source data Fig. 5 [file 44318_2025_545_MOESM6_ESM.zip › Fig 5 SD/5B/ADPr/2024-0819-114446_pub.tif]

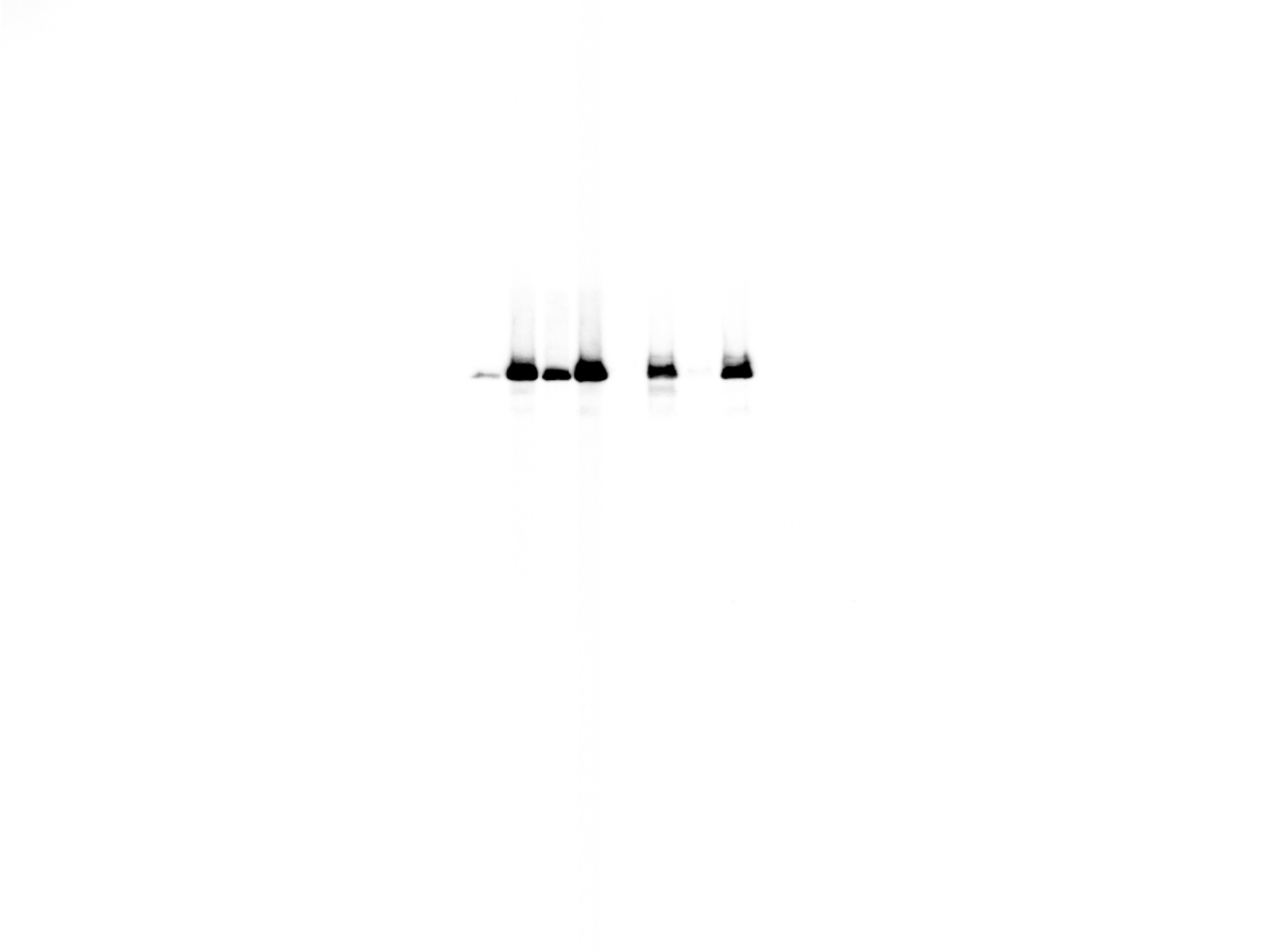

Supplement: Supplementary file 6 — Source data Fig. 5 [file 44318_2025_545_MOESM6_ESM.zip › Fig 5 SD/5B/GFP/2024-0819-114756_pub.tif]

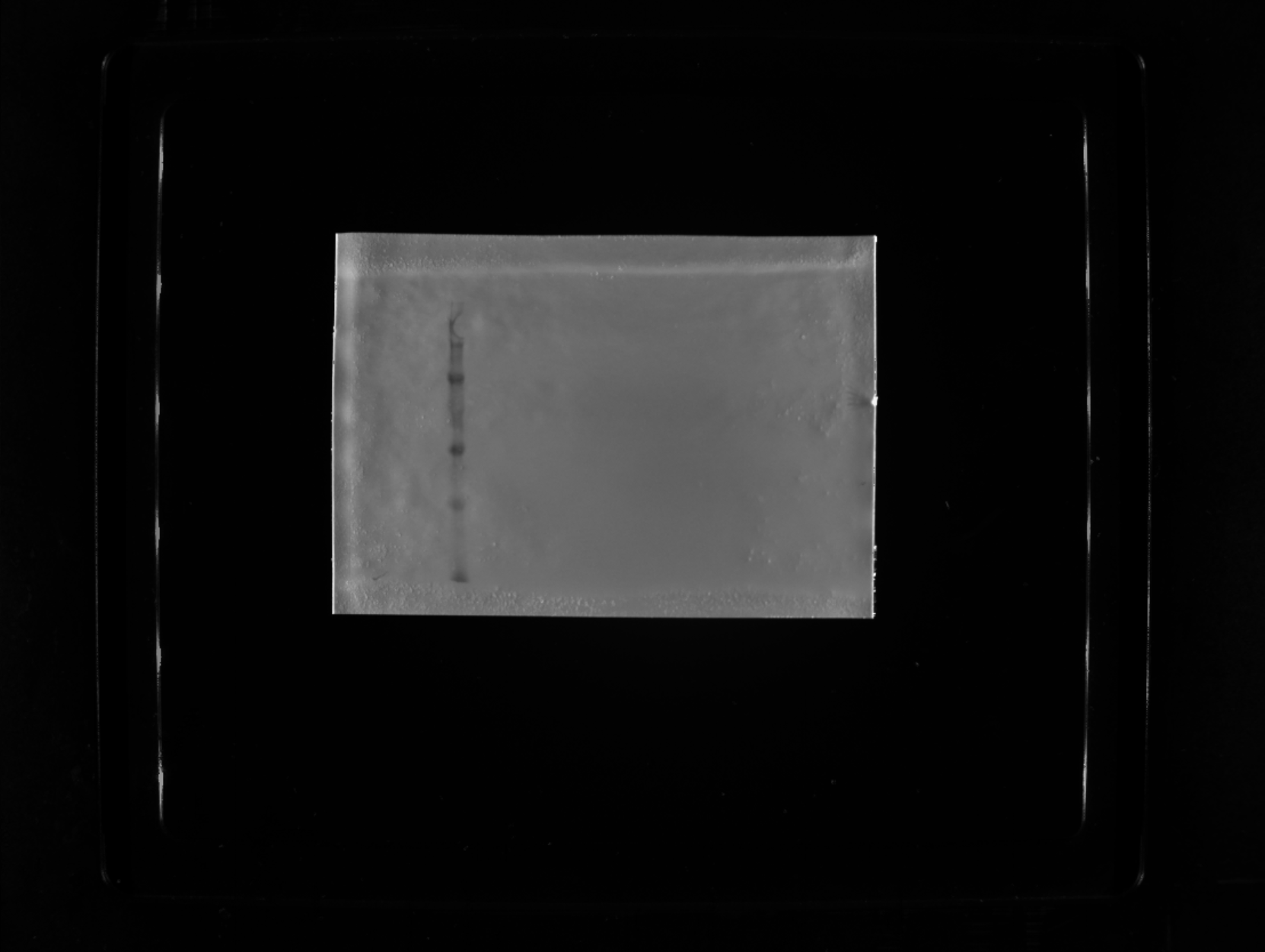

Supplement: Supplementary file 6 — Source data Fig. 5 [file 44318_2025_545_MOESM6_ESM.zip › Fig 5 SD/5B/GFP/2024-0819-114754_pub.tif]

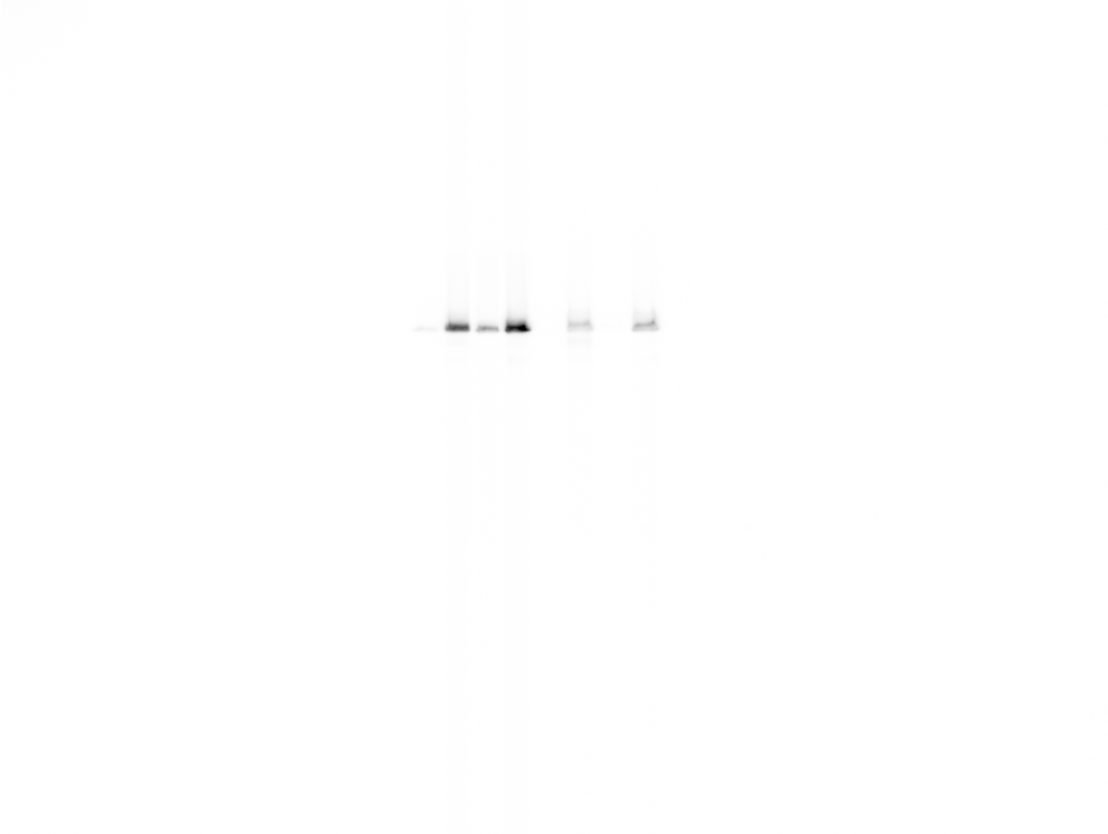

Supplement: Supplementary file 6 — Source data Fig. 5 [file 44318_2025_545_MOESM6_ESM.zip › Fig 5 SD/5B/GFP/2024-0819-114756.tif]

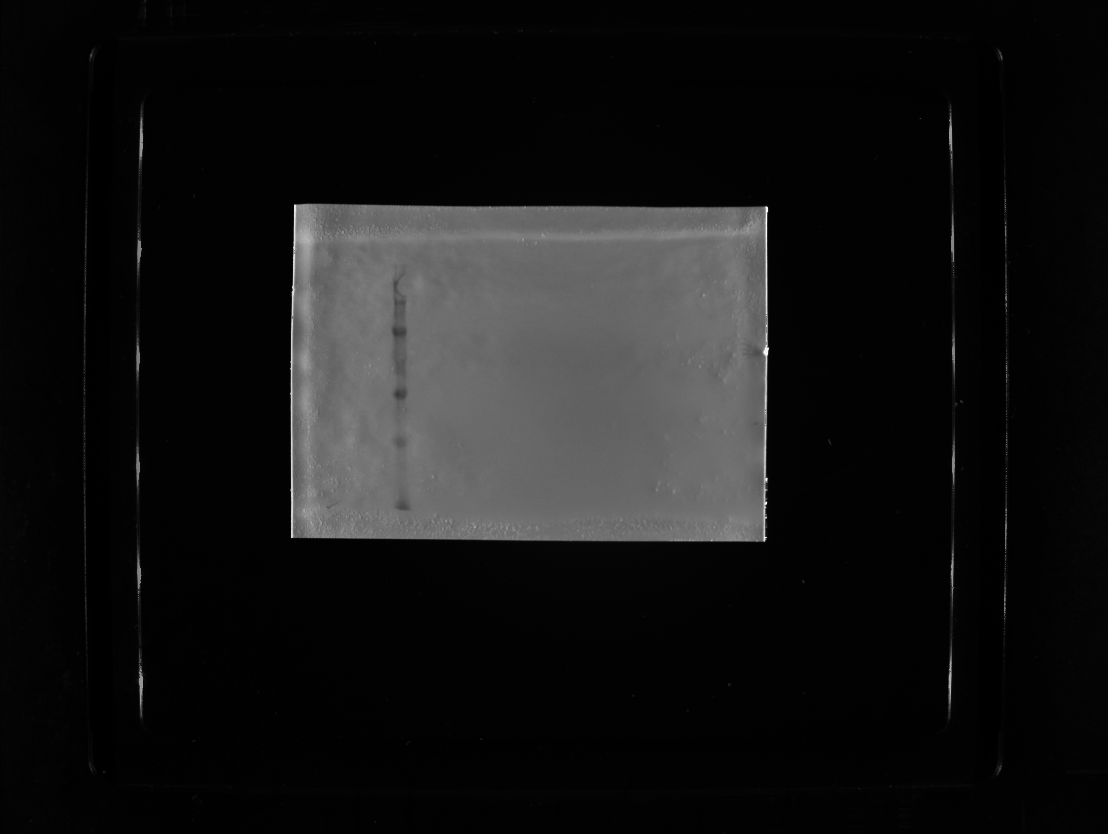

Supplement: Supplementary file 6 — Source data Fig. 5 [file 44318_2025_545_MOESM6_ESM.zip › Fig 5 SD/5B/GFP/2024-0819-114754.tif]
